# Metal-Catalyzed Enantioconvergent Transformations

**DOI:** 10.1021/acs.chemrev.3c00059

**Published:** 2023-10-04

**Authors:** Miguel Yus, Carmen Nájera, Francisco Foubelo, José M. Sansano

**Affiliations:** † Centro de Innovación en Química Avanzada (ORFEO−CINQA), Universidad de Alicante, Apdo. 99, E-03080 Alicante, Spain; ‡ Departamento de Química Orgánica and Instituto de Síntesis Orgánica (ISO), Universidad de Alicante, Apdo. 99, E-03080 Alicante, Spain

## Abstract

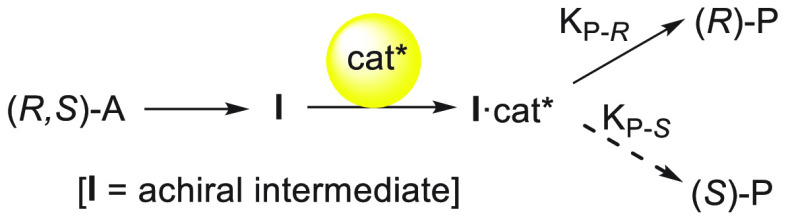

Enantioconvergent catalysis has expanded asymmetric synthesis to new methodologies able to convert racemic compounds into a single enantiomer. This review covers recent advances in transition-metal-catalyzed transformations, such as radical-based cross-coupling of racemic alkyl electrophiles with nucleophiles or racemic alkylmetals with electrophiles and reductive cross-coupling of two electrophiles mainly under Ni/bis(oxazoline) catalysis. C–H functionalization of racemic electrophiles or nucleophiles can be performed in an enantioconvergent manner. Hydroalkylation of alkenes, allenes, and acetylenes is an alternative to cross-coupling reactions. Hydrogen autotransfer has been applied to amination of racemic alcohols and C–C bond forming reactions (Guerbet reaction). Other metal-catalyzed reactions involve addition of racemic allylic systems to carbonyl compounds, propargylation of alcohols and phenols, amination of racemic 3-bromooxindoles, allenylation of carbonyl compounds with racemic allenolates or propargyl bromides, and hydroxylation of racemic 1,3-dicarbonyl compounds.

## 1 Introduction

**Figure 1:**
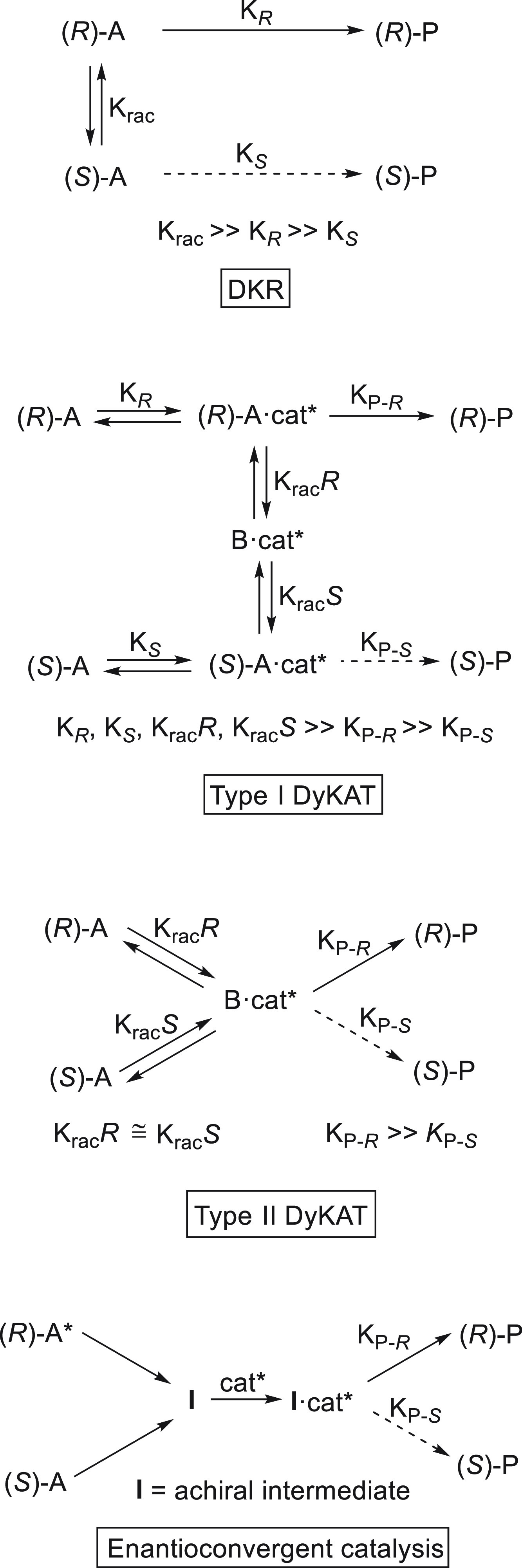
Conversion of racemates into a single enantiomer.

The preparation of enantiopure compounds is a major subject in chemistry. Enantiocatalytic methods are key methodologies nowadays to afford the synthesis of a single enantiomer. Desymmetrization reactions have been extensively applied to achiral or *meso*-compounds using metal-catalyzed, organocatalyzed, and enzymatic processes.^1−19^ Conversion of both enantiomers of a racemate into a single enantiomer has been carried out by dynamic kinetic resolutions (DKRs), dynamic kinetic asymmetric transformations (DyKATs), and enantioconvergent processes.^20−28^ In the case of DKR, the two enantiomers undergo a reversible racemization prior to the selective reaction of one enantiomer with the chiral catalyst, whereas in the DyKATs, the equilibration of both enantiomers is due to the chiral catalyst. In type I DyKATs, both enantiomers are bounded to the catalyst, and these intermediates undergo equilibration, whereas in type II DyKATs, the racemate loses the stereocenter by interaction with the chiral catalyst to form a prochiral intermediate B·cat*. In the case of enantioconvergent reactions, the equilibration is not necessary, and this deracemization methodology does not indicate to control kinetic factors, which are critical in DKRs and DyKATs processes. For enantioconvergent transformations, two enantiomers of the substrate are converted by a stereoablative reaction^20^ into a prochiral intermediate **I** that reacts with the chiral catalyst to give one enantiomeric product, for instance, (*R*)-P (Figure 1).

In this Review, we will focus on recent developments of enantioconvergent catalysis for asymmetric transformations. This strategy has become a booming methodology in the last 10 years in asymmetric catalysis mainly under transition metal C–C bond-forming reactions and also under organocatalysis for the preparation of enantioenriched compounds.

## 2 Enantioconvergent Cross-Couplings

In this Section, C–C bond-forming reactions of alkyl and aryl electrophiles with organometals will be considered. Bond-forming reactions by reaction of alkyl electrophiles with other nucleophiles have been performed in enantioconvergent processes. Reductive cross-electrophile couplings of Csp^2^–Csp^3^, Csp^3^–Csp^3^, and Csp^2^–Csp^2^ under Ni catalysis, as well as photoredox radical coupling, will be included. Cu catalysis under a radical mechanism gives enantioconvergent amination processes.

### 2.1 Racemic Alkyl Electrophiles with Organometals

Carbon–carbon bond formation under transition-metal-catalyzed cross-coupling reactions of acyl and vinyl electrophiles with organometals is an important methodology in organic synthesis.^29,30^ The development of cross-coupling reactions with alkyl electrophiles, especially secondary systems, was a challenging task because of β-hydride elimination processes^31^ and has been crucial for asymmetric transformations.^32,33^ Enantioconvergent cross-coupling reactions of alkyl electrophiles under Ni catalysis form radicals after the oxidative addition, which react with chiral Ni complexes to form the enantioenriched products.^21−25,34−36^ In this Section, enantioconvergent cross-coupling reactions of secondary alkyl electrophiles with organometals, such as Grignard reagents, organozinc, organoboron, organosilicon, organoindium, organozirconium, organoaluminum, and organotitanium organometallics, will be considered.

#### 2.1.1 Grignard Reagents

**Scheme 1:**
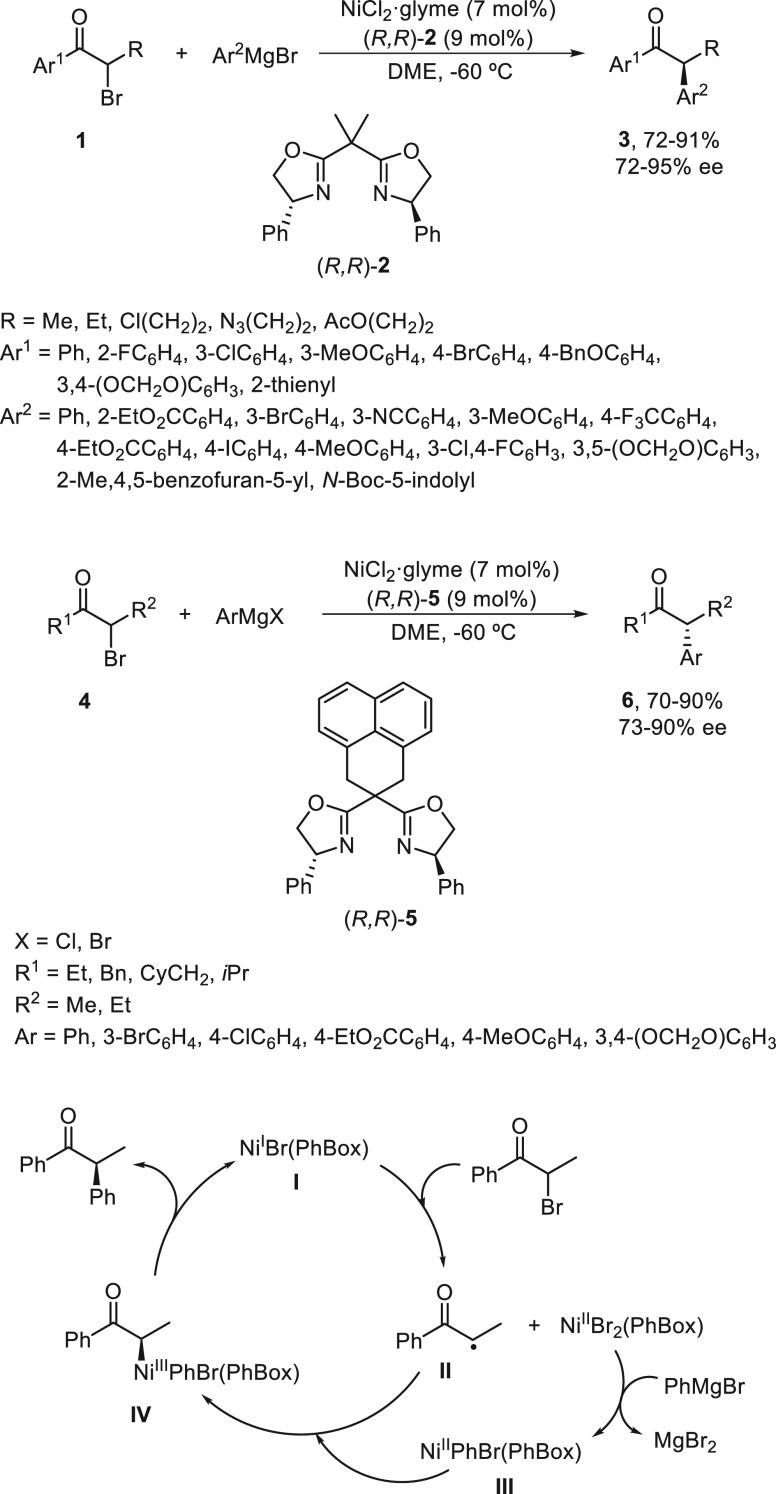
Enantioconvergent Ni-Catalyzed Kumada Reactions of α-Bromoketones **1** and **4** with Arylmagnesium Halides

Enantioconvergent Kumada reactions were described for the first time by Lou and Fu.^37^ Racemic α-bromoketones **1** were coupled with arylmagnesium reagents using chiral nickel/bis(oxazoline) catalyst **2** in dimethoxyethane (DME) at −60 °C (Scheme 1). In the case of alkyl aryl ketones, 7 mol % of NiCl_2_·glyme and 9 mol % of (*R,R*)-PhBox (**2**) were used as catalyst with different arylmagnesium bromides at −60 °C to give the corresponding enantioenriched ketones **3** in good yields (72–91%) and up to 95% ee. When the same reaction conditions were applied to dialkyl ketones **4**, ligand **5** gave the best results by working at −40 °C to provide compounds **6** in 70–90% yield and up to 90% ee. Recently, Yin and Fu^38^ performed mechanistic investigation for the reaction of α-bromo propiophenone with PhMgBr and NiBr_2_/PhBox as catalyst, thereby establishing that the C–C bond formation process works via a radical chain process. In the proposed catalytic cycle, a nickel radical **I** abstracts a halogen atom from the α-bromo ketone to generate the radical **II** and NiBr_2_(PhBox), which reacts with phenylmagnesium bromide to form intermediate **III**. Radical **II**, by reaction with **III**, gives an organonickel(III) intermediate **IV** through an out-of-cage pathway. Final reductive elimination of **IV** affords the coupling product and regenerates the chain-carrying radical **I**. According to DFT calculations, the coupling between intermediates **II** and **III** is the stereochemistry-determining step.

**Scheme 2:**
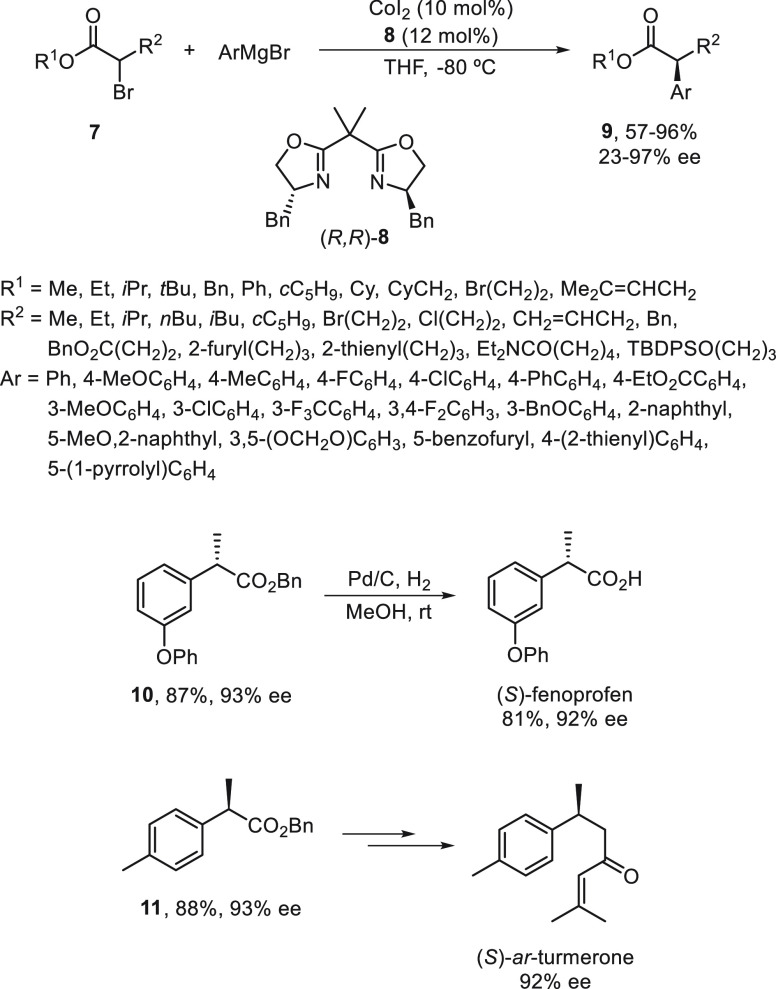
Enantioconvergent Co-Catalyzed Kumada Reactions of α-Bromo Esters **7** with Arylmagnesium Bromide

Zhong, Bian, and co-workers^39^ described the cobalt-catalyzed enantioconvergent Kumada reaction of α-bromo esters **7** using bis(oxazoline) **8** as chiral ligands (Scheme 2). A variety of chiral α-aryl alkanoic esters **9** were prepared using CoI_2_ (10 mol %) and ligand (*R,R*)-**8** (12 mol %) in THF at −80 °C with good yields (up to 96%) and enantioselectivities (up to 97% ee). This methodology was applied to the synthesis of the nonsteroidal anti-inflammatory drug (NSAID) (*S*)-fenoprofen by Kumada reaction to give ester **10** followed by debenzylation with hydrogen over Pd/C in 92% ee and 70% overall yield. For the total synthesis of (*S*)-*ar*-turmerone ester, (*R*)-**11**, which was obtained in 88% yield and 93% ee in gram scale, was further transformed in six steps into this sesquiterpene in 92% ee.

**Scheme 3:**
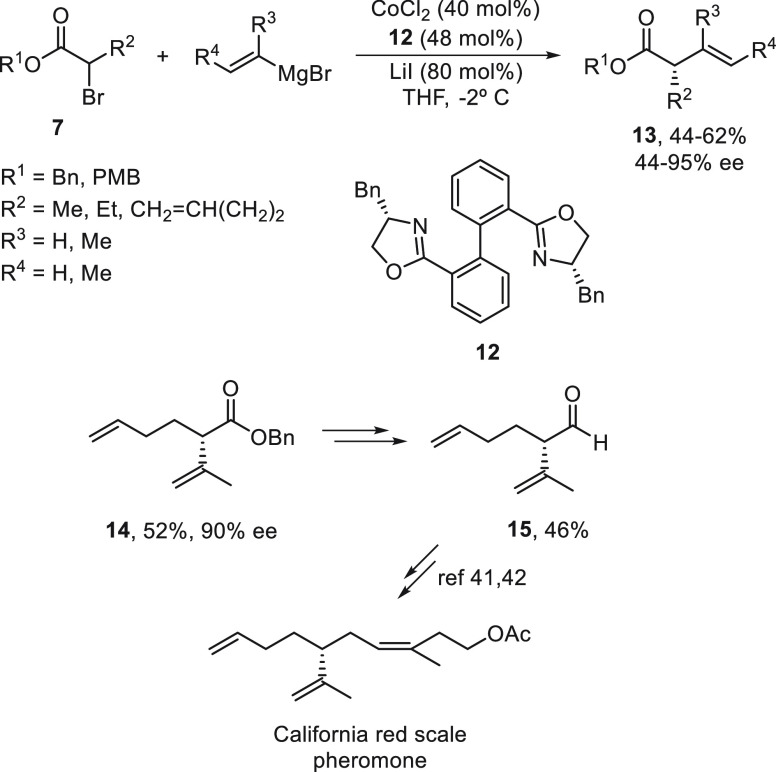
Enantioconvergent Co-Catalyzed Kumada Reaction of α-Bromo Esters **7** with Alkenyl Grignard Reagents

Recently, Zhong’s group^40^ achieved the Kumada reaction of α-bromo esters **7** with alkenyl Grignard reagents using a cobalt-bis(oxazoline) **12** catalysis to afford highly enantioenriched α-alkyl-β,γ-unsaturated esters **13** (Scheme 3). This enantioconvergent cross-coupling was applied to the formal synthesis of the California red scale pheromone isolated from female *Aonidiella aurantia* (Maskell). Ester **14** was obtained in 52% yield and 90% ee, and after reduction to the alcohol, oxidation to the aldehyde, and Wittig reaction, (*R*)-**15** was further transformed^41,42^ into this pheromone. From the radical clock experiments, it could be assumed that this cross-coupling took place via a radical intermediate.

**Scheme 4:**
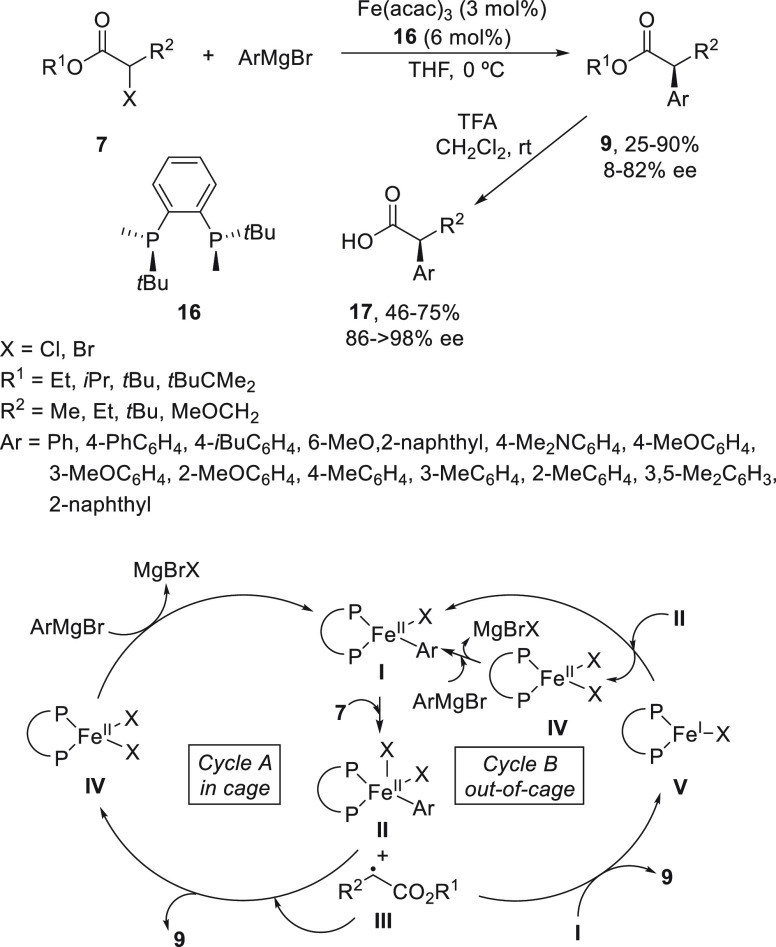
Enantioconvergent Fe-Catalyzed Kumada Reactions of α-Chloro and α-Bromo Esters **7** with Arylmagnesium Bromides

Iron-catalyzed enantioconvergent Kumada reaction of α-chloro and α-bromo alkanoates **7** has been described by Nakamura and co-workers.^43^ This cross-coupling is catalyzed by Fe(acac)_3_ and the chiral phosphine (*R,R*)-BenzP (**16**) working in THF at 0 °C to give esters **9** in up to 92% yield and 82% ee (Scheme 4). Compounds **9** were readily transformed into the corresponding α-aryl alkanoic acids **17** with up to >98% ee by simple deprotection/recrystallization. Radical probe experiments suggested a catalytic cycle in which the divalent iron species **I** was generated by partial reduction of Fe(acac)_3_ by the ligand. This species **I** abstracts the halogen from the substrate to give the iron species **II** and radical **III**. The arylation of intermediate **III** takes place by the aryl group of the iron species **II** in the solvent cage to provide the product **9** and the iron complex **IV**, which undergoes transmetalation with the Grignard reagent to regenerate species **I** (cycle A). A most favorable alternative process is depicted in cycle B on the basis of a bimetallic mechanism.^44^ In this out-of-cage mechanism, the radical **III** escapes from the solvent cage to react with the iron species **I** to form the coupling product **9** by forming the iron species **V**. Comproportionation of complexes **II** and **V** forms iron(II) species **I** and **IV**, or halogen abstraction of **V** from the α-halo ester **7** forms **IV** and radical **III**, which may participate in a chain reaction process.

Cross-couplings of α-bromo ketones can be enantioconvergently arylated under Ni/bis(oxazoline) catalysis. This arylation was also performed with α-halogenated esters using arylmagnesium bromides under Co/bis(oxazoline) and by Fe/diphosphine catalysis. Radical processes have been postulated in all these cases.

#### 2.1.2 Organozinc Reagents

**Scheme 5:**
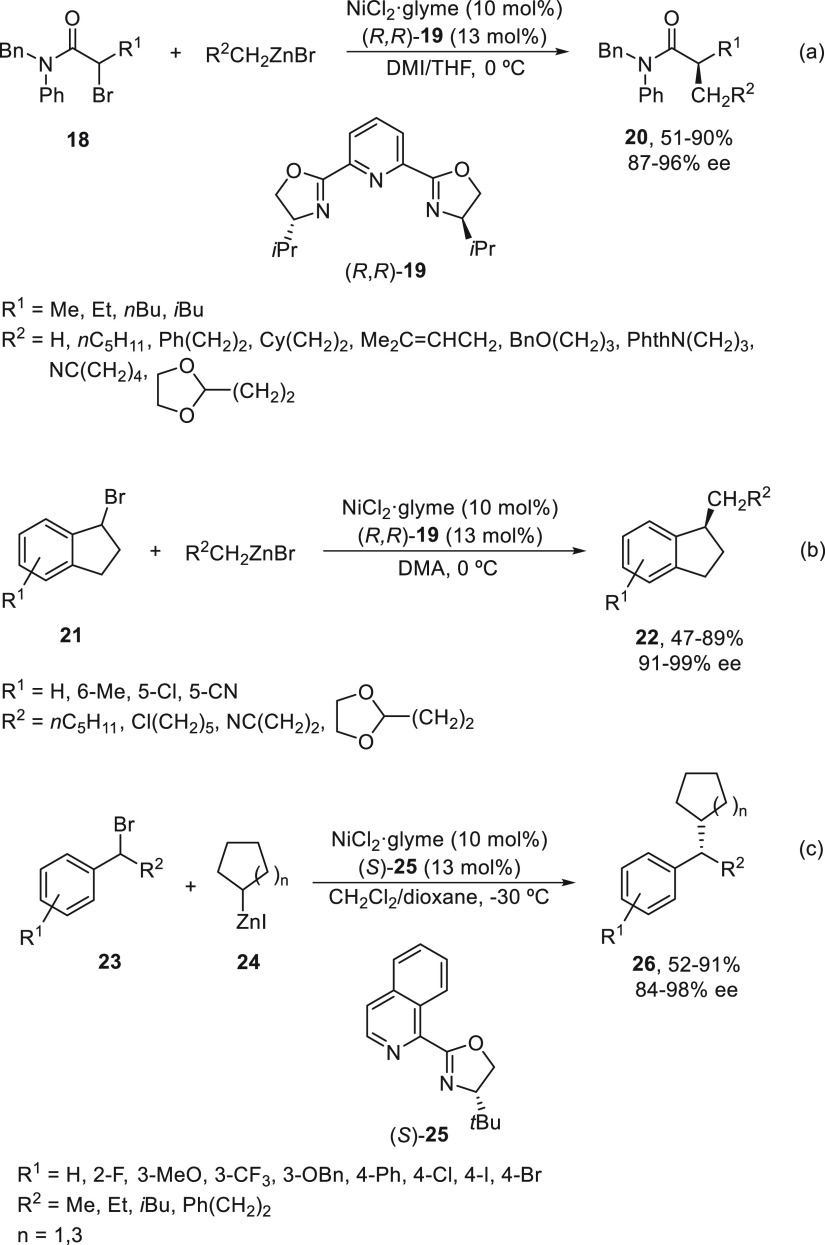
Enantioconvergent Ni-Catalyzed Negishi Reactions of α-Bromo Amides **18** and Benzylic Halides **21** and **23** with Alkylzinc Reagents

The first advantage in metal-catalyzed enantioconvergent cross-coupling of racemic alkyl electrophiles was performed with organozinc reagents by Fu and co-workers. Using NiCl_2_·glyme/(*R,R*)-*i*PrPyBox (**19**), α-bromo amides **18** underwent Negishi alkylation with alkylzinc bromides in 1,3-dimethyl-2-imidazolidinone (DMI)/THF at 0 °C to provide products **20** in up to 90% yield (Scheme 5a).^45^ Secondary benzylic halides, such as bromoindanes **21**, reacted with primary alkylzinc bromides to give the corresponding alkylated derivatives **22** using *N,N*-dimethylacetamide (DMA) as solvent at 0 °C and the same catalyst in up to 89% yield and 99% ee (Scheme 5b).^46^ Further studies about this sp^3^–sp^3^ cross coupling but using secondary alkylzinc iodides **24** showed that an isoquinoline-oxazoline ligand (*S*)-**25** gave the best results for secondary alkyl bromides **23** to afford products **26** in up to 91% yield and 98% ee (Scheme 5c).^47^

**Scheme 6:**
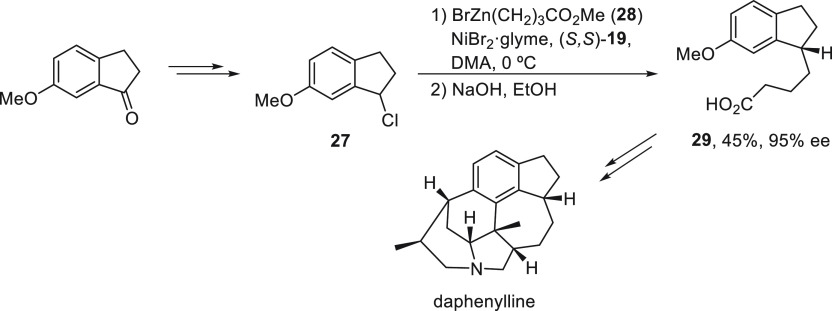
Enantioconvergent Ni-Catalyzed Negishi Reaction of Chloroindane **27** with Alkylzinc **28**

Yokoshima, Fukuyama, and co-workers^48^ employed this enantioconvergent Negishi reaction in the total synthesis of the alkaloid (−)-daphenylline isolated from the fruit *Daphniphyllum longeracemosum*.^49^ The chloroindane **27** reacted with the organozinc reagent **28** using NiBr_2_·glyme and (*S,S*)-*i*PrPyBox (**19**) as catalyst in DMA at 0 °C to furnish the corresponding acid **29** in 94% ee after hydrolysis and >98% ee after recrystallization of the salt formed with (*R*)-1-phenylethylamine (Scheme 6).

Mechanistic studies by DFT calculations were performed by Lin and co-workers^50^ for the Negishi reaction of bromoindoles **21**(46) to corroborate the Ni(I)–Ni(III) mechanism containing sequential oxidative addition–reductive elimination, which is more favorable than the Ni(0)–Ni(II) mechanism. In contrast to the calculations of Fu and co-workers^38^ for the Kumada reaction, in this case, it was suggested that the reductive elimination is the stereochemistry-determining step and not the coupling of the organonickel(II) complex with the organic radical (see Scheme 1).

**Scheme 7:**
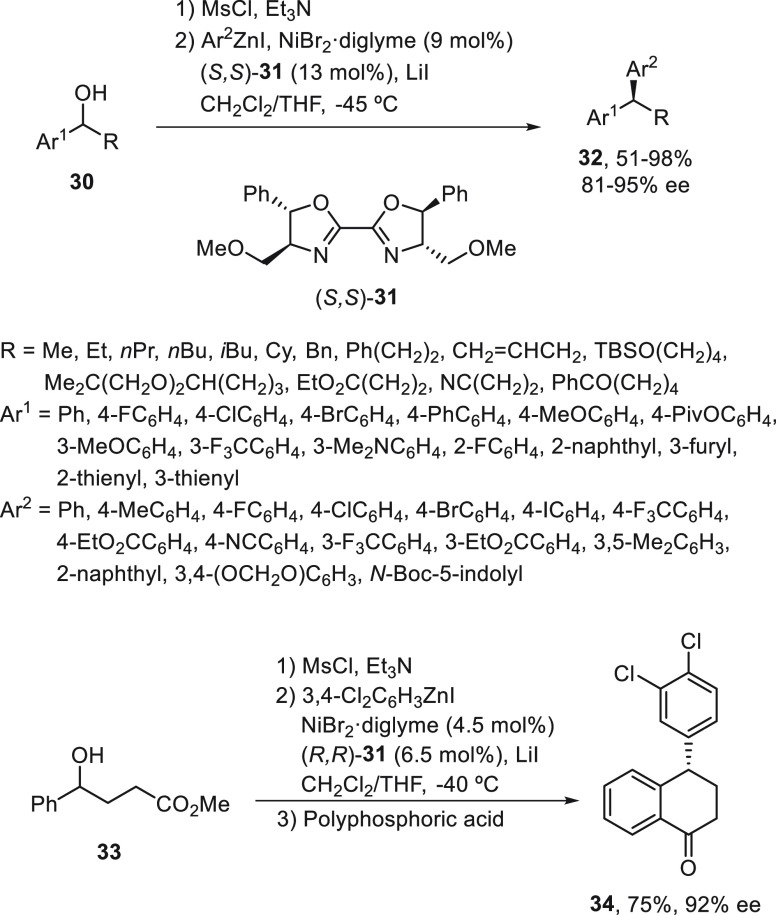
Enantioconvergent Ni-Catalyzed Negishi Reactions of Benzylic Mesylates with Arylzinc Reagents

Fu and co-workers^51^ obtained poor results when benzylic halides were allowed to react with arylzinc reagents. Instead, racemic benzylic mesylates were efficiently arylated using NiBr_2_·diglyme (9 mol %) and the bis(oxazoline) (*S,S*)-**31** (Scheme 7). Starting from benzylic alcohols **30** after mesylation, the subsequent Negishi reaction was carried out in a one-pot process to provide 1,1-diarylalkanes **32** up to 98% yield and 95% ee. This method was applied to a gram-scale synthesis of (*S*)-sertraline tetralone **34** from alcohol **33**, a precursor of sertraline hydrochloride (Zoloft, an antidepressant drug).

**Scheme 8:**
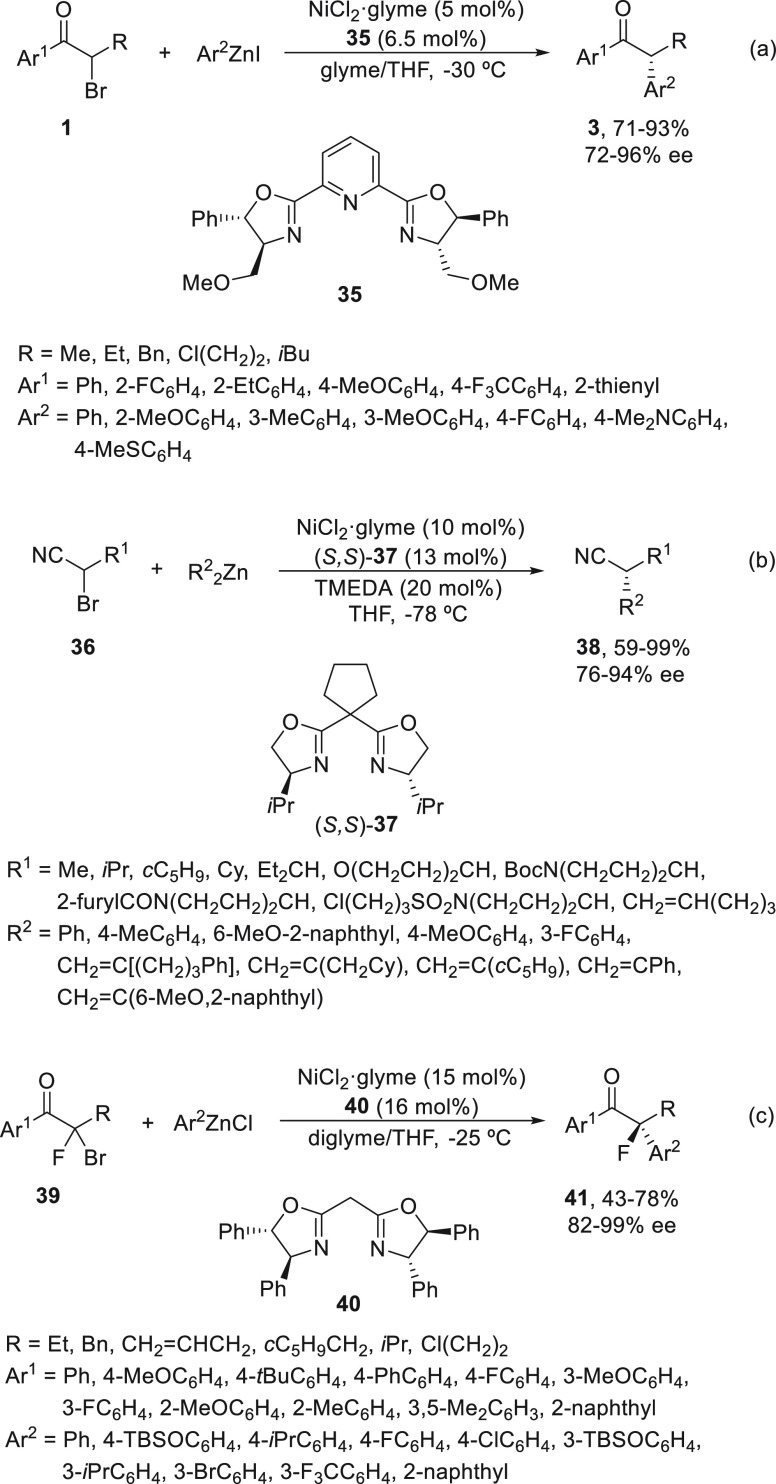
Enantioconvergent Ni-Catalyzed Negishi Reactions of α-Bromo Ketones **1**, α-Bromo Nitriles **36**, and α-Bromo-α-fluoro Ketones **39** with Aryl and Alkenylzinc Reagents

Racemic α-bromo ketones **1** have been arylated to ketones **3** with arylzinc iodides using NiCl_2_·glyme (5 mol %) and the PyBox ligand **35** with very good yields and enantioselectivities by Fu and co-workers^51^ (Scheme 8a). If the aryl group of the ketone was bulky, moderate enantioselectivity was observed. The reaction took place under mild reaction conditions with glyme/THF at −30 °C. α-Bromo nitriles **36** underwent enantioconvergent Negishi arylations and alkenylations^52^ using the PyBox ligand (*S,S*)-**37** in the presence of tetramethylenediamine (TMEDA) (20 mol %) in THF at −78 °C to provide nitriles **38** (Scheme 8b). In this case, substrates prone to undergo cyclization gave acyclic products, which suggests that instead of radical intermediates, this cross-coupling proceeds by conventional oxidative addition. Another case of activated electrophiles is the arylation of α-bromo-α-fluoro ketones **39** using a bis(oxazoline) **40** at −25 °C (Scheme 8c).^53^ The corresponding tertiary alkyl fluorides **41** were obtained in good yields and high enantioselectivity (up to 99%), which can be further transformed into a variety of organofluorine target molecules.

**Scheme 9:**
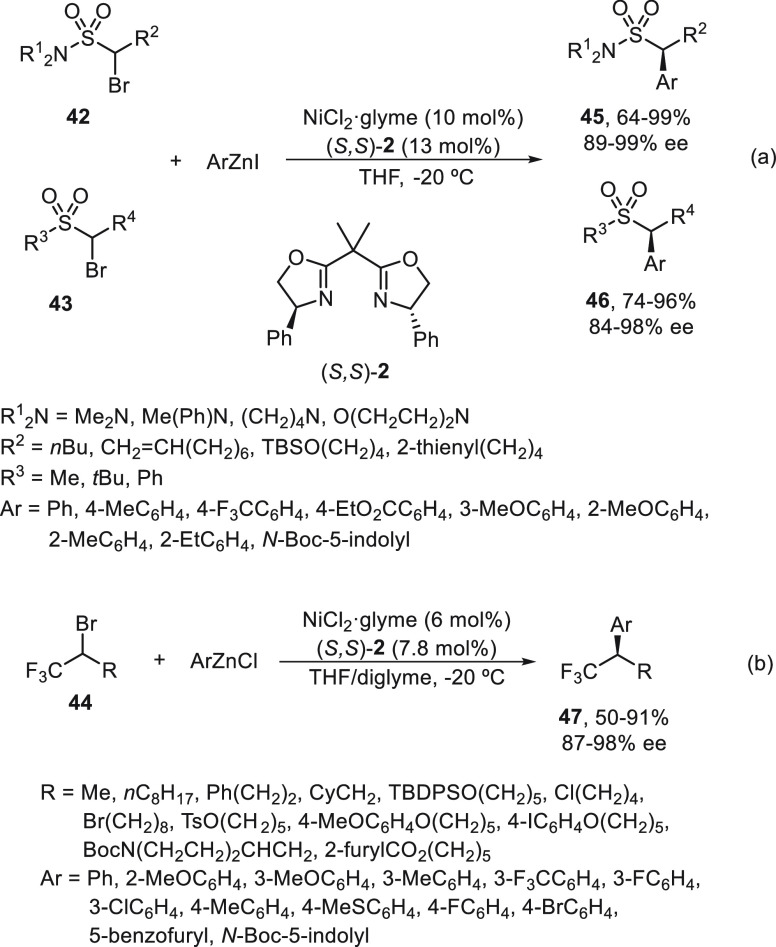
Enantioconvergent Ni-Catalyzed Negishi Reactions of α-Bromoalkyl Sulfonamides **42** and Sulfones **43**, as Well as CF_3_–Substituted Alkyl Bromides **44** with Arylzinc Reagents

Fu and co-workers^54,55^ studied the enantioconvergent Negishi reaction of unactivated alkyl electrophiles, such as α-bromo sulfonamides **42**(54) and sulfones **43**,^54^ as well as CF_3_-substituted alkyl bromides **44**(55) (Scheme 9). In all cases, NiCl_2_·glyme/(*S,S*)-PhBox (**2**) was used as catalyst at −20 °C with very good enantioselectivity. Sulfonamides **45** and sulfones **46** were obtained by arylation of the starting bromo derivatives **42** and **43**, respectively (Scheme 9a).^55^ Experimental mechanistic studies provided evidence for a radical intermediate that has a sufficient lifetime to escape from the solvent cage and to cyclize onto a pendant olefin. Trifluoromethyl-substituted secondary alkyl bromides **44** were transformed into compounds **47** by reaction with arylzinc chlorides in very good yields and enantioselectivities (Scheme 9b).^55^ It is noteworthy that the Ni catalyst was able to differentiate between a CF_3_ and an alkyl substituent in the asymmetric cross-coupling.

**Scheme 10:**
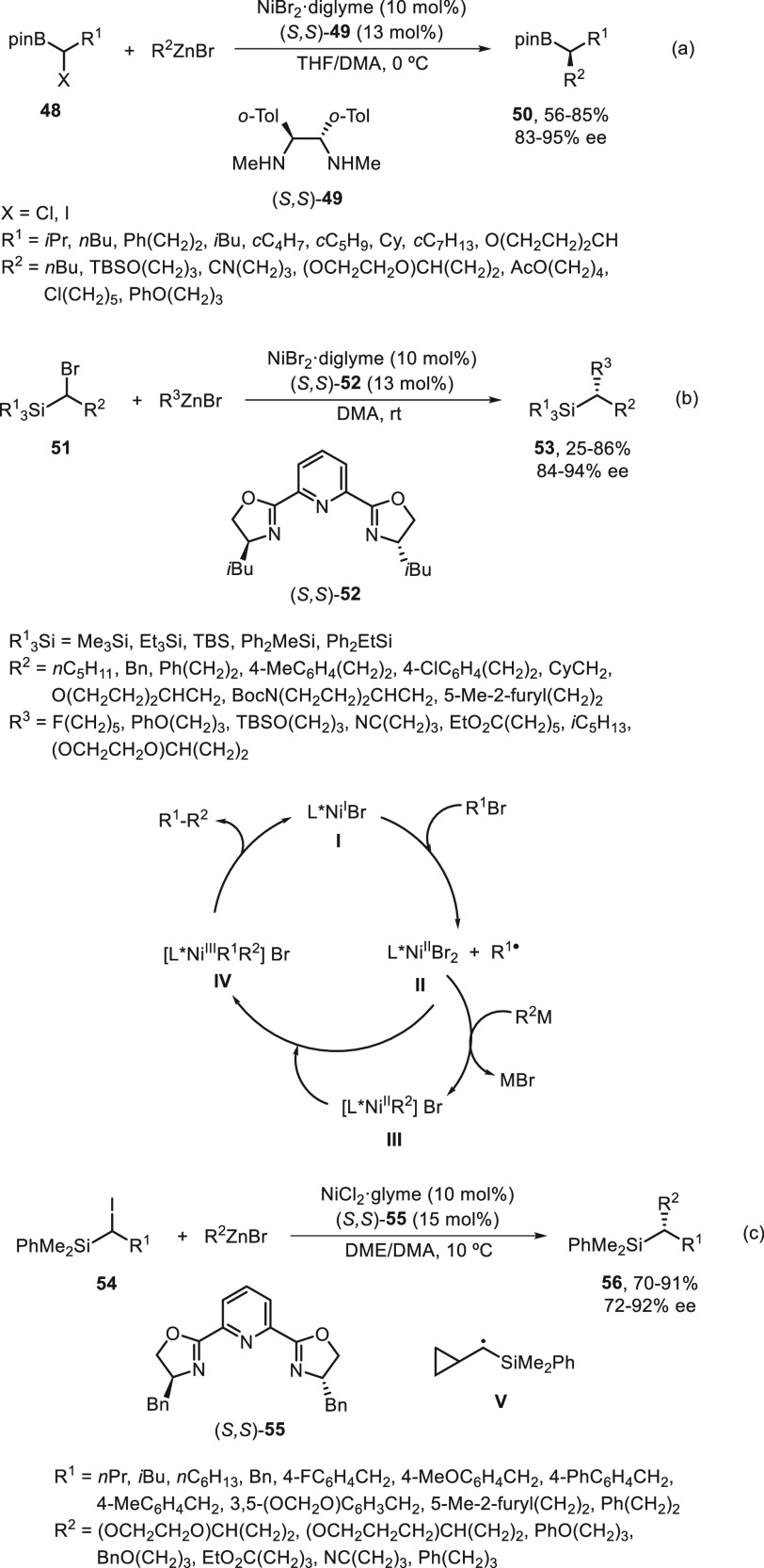
Enantioconvergent Ni-Catalyzed Negishi Reactions of α-Haloboronates **48** and α-Halosilanes **51** and **54** with Alkylzinc Bromides

Enantioconvergent substitution reactions of α-haloboronates^56^ and α-halosilanes^57^ with alkylzinc reagents catalyzed by nickel have been carried out by Fu and co-workers. Enantioenriched alkylboronate esters are powerful building blocks in synthesis because of the facile transformation of C–B bonds into C–heteroatom bonds in a stereospecific manner. Using racemic α-haloboronates **48**, enantioconvergent alkylation with alkylzinc reagents took place under NiBr_2_·diglyme/(*S,S*)-diamine **49** catalysis to furnish enantioenriched alkylboronates **50** (Scheme 10a).^56^ This alkyl–alkyl cross-coupling was carried out under mild reaction conditions THF/DMA at 0 °C and was compatible with different functional groups working with good yields and enantioselectivities. Several transformations to other families of enantioenriched compounds by C–C, C–N, C–halogen, and C–O bond formation were performed with little or no erosion in enantiomeric excess. Because of the synthetic interest of enantioenriched silanes, especially in medicinal chemistry,^58−60^ Matier, Fu, and Schwarzwalder^57^ studied the enantioconvergent cross-coupling of α-bromosilanes **51** with alkylzinc reagents in the presence of NiBr_2_·diglyme/bis(oxazoline) **52** (Scheme 10b). This alkylation proceeded at room temperature in DMA to give enantioenriched organosilanes **53** in moderate to good yields and up to 94% ee. Experiments with an enantioenriched α-bromosilane indicate that racemization occurred under the standard conditions, thereby confirming that the C–Br cleavage is reversible in discarding a DKR. In the proposed mechanism based on ESI-MS analysis and EPR spectrum, the Ni complex **III** is the predominant resting state during the catalytic cycle in which intermediates **I–IV** are involved. A consecutive publication of Oestreich and co-workers^61^ about the enantioconvergent alkylation of racemic α-iodosilanes **54** with alkylzinc bromides used similar reaction conditions (Scheme 10c). In this case, NiCl_2_/(*S,S*)-BnPyBox (**55**) was used as catalyst in a 5:2 mixture of DME/DMA at 10 °C to form the enantioenriched silanes **56** in high yield and slightly lower enantioselectivity. Control experiments with R^1^ = cyclopropyl revealed a radical clock mechanism supporting the intermediacy of a silicon-stabilized radical **V**.

**Scheme 11:**
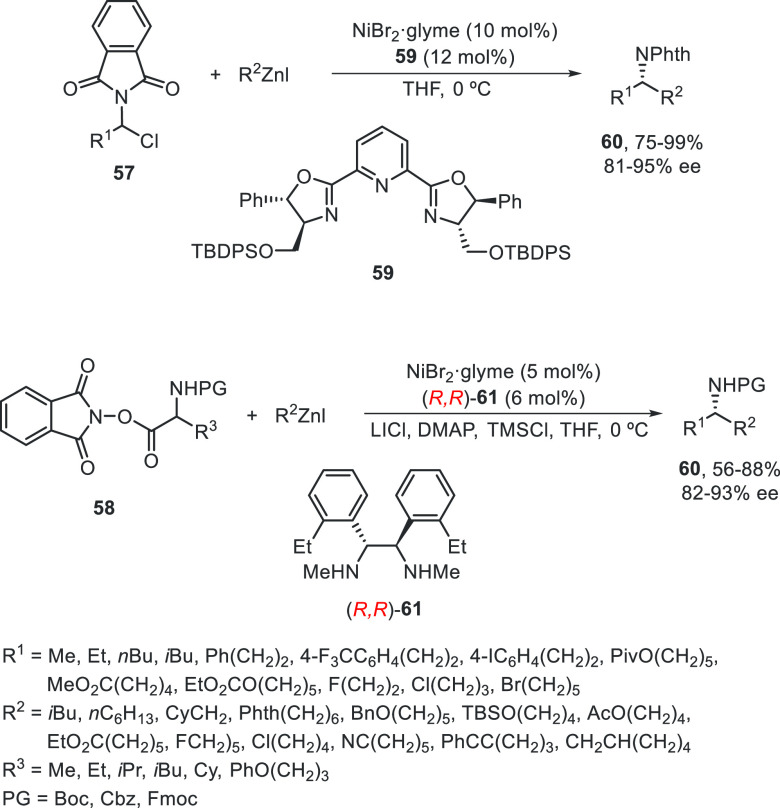
Enantioconvergent Ni-Catalyzed Negishi Reaction of α-Phthalimido Alkyl Chlorides **57** or *N*-Hydroxyphthalimide Esters **58** of Protected α-Amino Acids with Alkylzinc Iodides

Enantioconvergent synthesis of amines has been achieved by Fu and co-workers using α-phthalimido alkyl chlorides **57** or *N*-hydroxyphthalimide (NHP) esters **58** of protected α-amino acids as electrophiles.^62^ Primary amines protected as phthalimides **57** reacted with alkylzinc iodides using NiBr_2_/bis(oxazoline) **59** as catalyst to provide protected dialkyl carbinamines **60** in good yields and enantioselectivities (Scheme 11). In the case of redox-active NHP esters **58**, a decarboxylative coupling with alkylzinc iodides takes place in the presence of NiBr_2_/diamine **61** as catalyst to afford *N*-protected dialkyl carbinamines **60** in good yields and enantioselectivities.

**Scheme 12:**
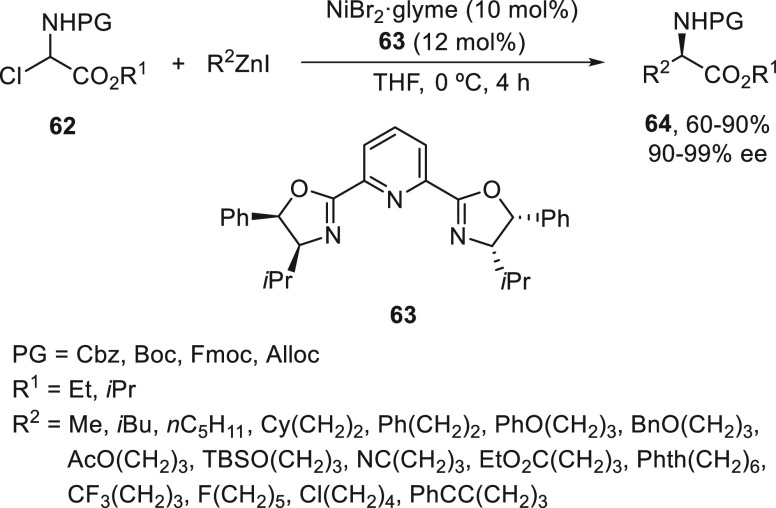
Enantioconvergent Ni-Catalyzed Negishi Reactions of α-Chloro Glycinates **62** with Alkylzinc Iodides

Recently, Fu and co-workers^63^ applied the Ni-catalyzed enantioconvergent Negishi reaction to the synthesis of α-amino acids. Starting from *N*-protected α-chloro glycinates **62**,^64^ the alkylation with alkylzinc iodides (1:1.1 molar ratio) using NiBr_2_·glyme/bis(oxazoline) **63** as catalyst in THF at 0 °C furnished protected α-amino acids **64** in good yields (60–90%) and excellent enantioselectivities (up to 99%) (Scheme 12). These couplings were achieved under mild reaction conditions and are tolerant of air, moisture, and a wide variety of functional groups, and have been applied to the synthesis of intermediates en route to bioactive compounds in gram scale.

**Scheme 13:**
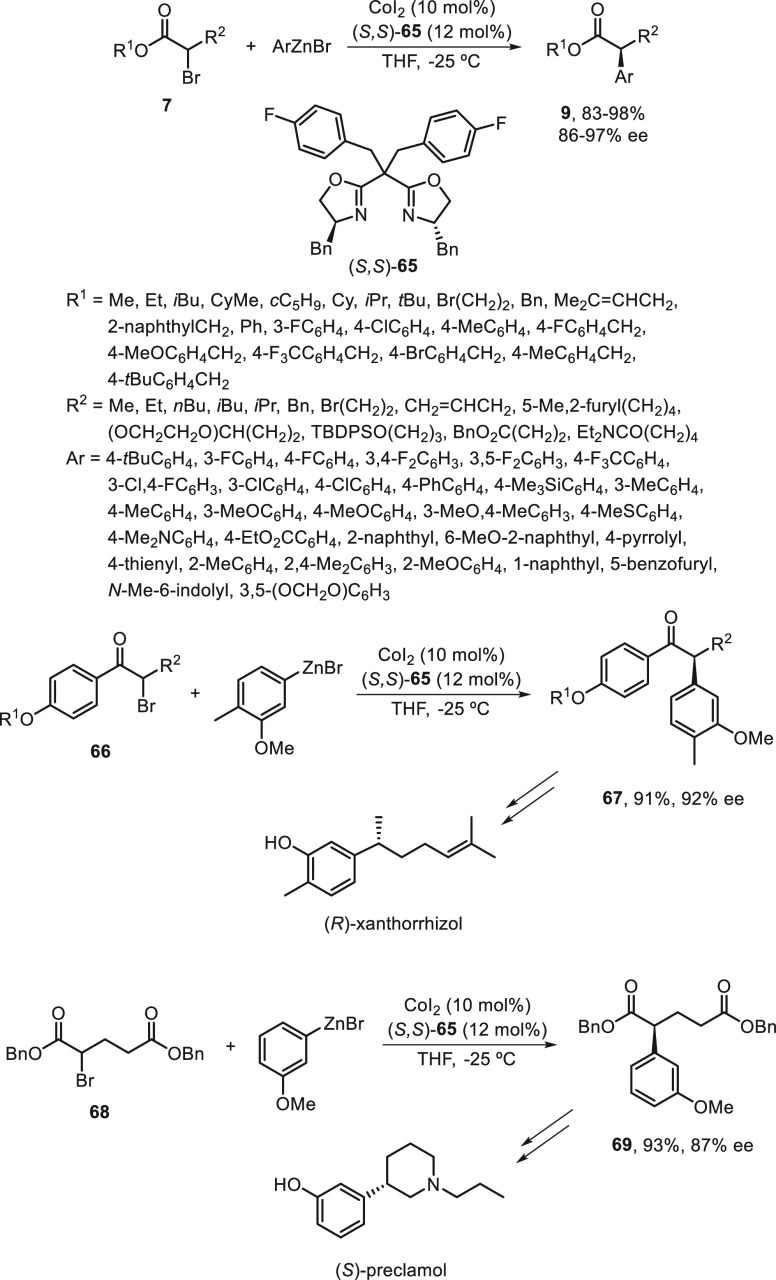
Enantioconvergent Co-Catalyzed Negishi Reactions of α-Bromo Esters **7** with Arylzinc Bromides

Cobalt-catalyzed enantioconvergent cross-couplings of racemic α-bromo esters **7** with arylzinc reagents, previously described with arylmagnesium reagents (Scheme 2),^38^ have been performed by Bian and co-workers.^65,66^ Differently substituted α-bromo esters **7** reacted with arylzinc bromides using CoI_2_/bis(oxazoline) (*S,S*)-**65** as chiral catalyst in THF at 25 °C to provide compounds **9** in very good yields and ee (Scheme 13). They used radical probes **7** bearing a cyclopropyl and 4-pent-en-1-yl substituents to demonstrate the radical pathway of this Co-catalyzed Negishi reaction. This process was applied in gram scale to the synthesis of the sesquiterpene (*R*)-xanthorrhizol isolated from *Curcuma xanthorrhiza* Roxb. rhizome, which has anti-inflammatory, antioxidant and antiestrogenic properties. In this case, the reaction of ester **66** with 4-methyl-3-methoxyphenylzinc bromide gave ester **67** with 91% yield and 92% ee, which is the key precursor of (*R*)-xanthorrizol. The same group has performed the enantioselective synthesis of (*S*)-predamol, a central dopamine receptor agonist via a Co-catalyzed enantioconvergent Negishi reaction (Scheme 13).^66^ The α-bromo ester **68** was allowed to react with 3-methoxyphenylzinc bromide under the previously described reaction conditions to provide **69**, a key precursor of (*S*)-preclamol.

**Scheme 14:**
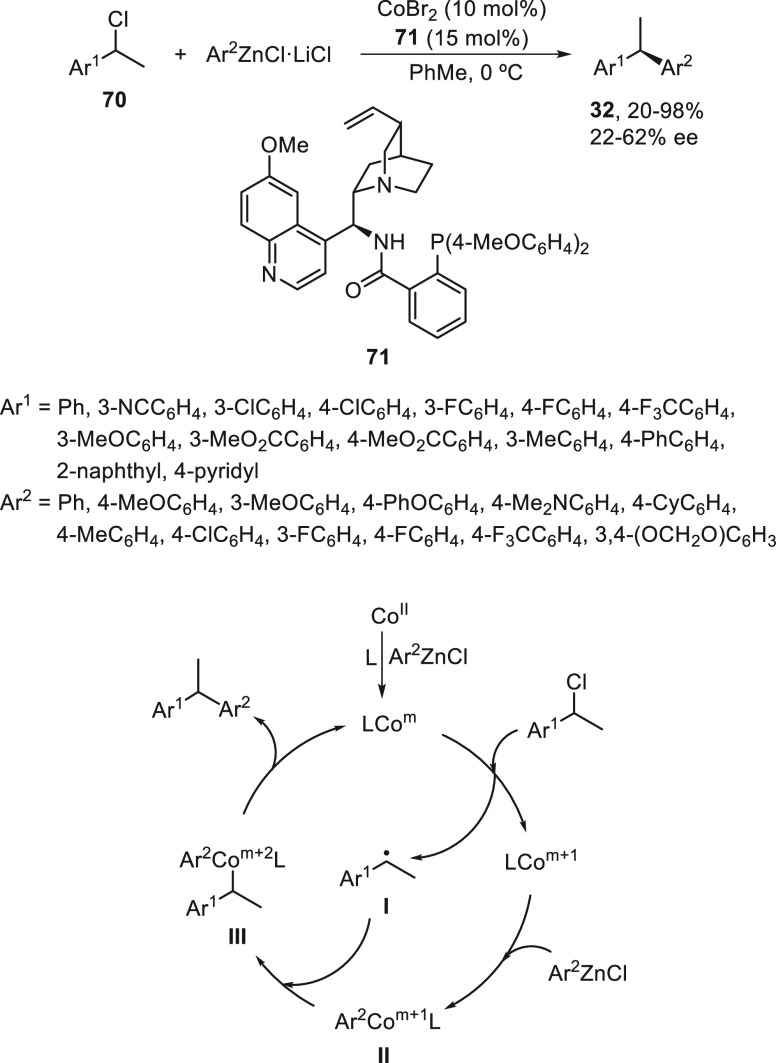
Enantioconvergent Co-Catalyzed Negishi Reactions of Benzyl Chlorides **70** with Arylzinc Reagents

A Negishi C(sp^3^)–C(sp^2^) cross-coupling of racemic benzyl chlorides **70** with arylzinc reagents has been carried out under Co catalysis by Gu, Liu, and co-workers.^67^ In this case, a chiral monodentate anionic ligand **71** and CoBr_2_ were used as catalysts in toluene at 0 °C to give 1,1-diarylethanes **32** in up to 98% yield and moderate ee (Scheme 14). According to experimental studies, a radical mechanism has been proposed. The Co^II^ is reduced by the arylzinc reagent to the active catalytic species LCo^III^, which undergoes an electron-transfer reaction with the benzyl chloride to generate a benzylic radical **I** and the LCo^*m*+1^ species. Subsequent transmetalation between the LCo^*m*+1^ species and the arylzinc reagent provides complex **II**, which recombines with the benzyl radical **I** to deliver complex **III**. Final reductive elimination of **III** furnished the coupling product and regenerated the catalyst.

As a summary of this Section 2.1.2, nickel-catalyzed enantioconvergent cross-coupling reactions allow the C–C bond formation between activated secondary alkyl electrophiles and alkyl or arylzinc reagents to form enantioenriched compounds mainly using mono- and bis(oxazolines) as chiral ligands. In some cases, this Negishi reaction can be also performed under cobalt catalysis. The most favorable mechanism involves the formation of an alkyl radical, which reacts with the chiral organonickel or cobalt intermediates through an out-of-cage pathway to afford the coupling product by final reductive elimination.

#### 2.1.3 Organoboron Reagents

**Scheme 15:**
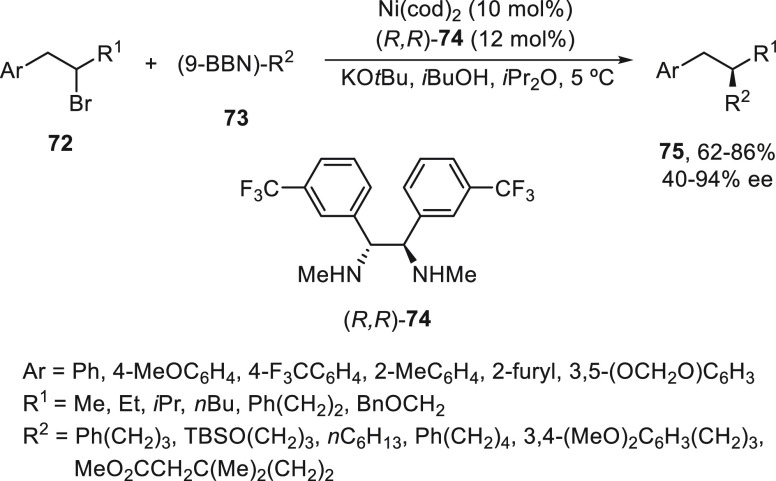
Enantioconvergent Ni-Catalyzed Suzuki Reactions of Homobenzylic Bromides **72** with Alkylboranes **73**

Nickel-catalyzed Suzuki cross-couplings of alkyl halides and alkylboronic acids were first described by Zhou and Fu.^68^ On the basis of the cross-coupling of unactivated alkyl halides with alkylboranes using *trans-N,N′*-dimethyl-1,2-cyclohexanediamine,^69^ Saito and Fu^70^ performed an enantioconvergent version of this alkyl–alkyl Suzuki reaction using a chiral diamine as ligand. Homobenzylic bromides **72** reacted with alkyl-(9-BBN) **73** using Ni(cod)_2_/(*R,R*)-**74** as catalyst to give products **75** in good yields (up to 86%) and ee (up to 94%) (Scheme 15). This method was applied to the arylation of racemic α-chloro- and α-bromo amides in which a modest kinetic resolution of the α-chloro amide was observed.^71^

**Scheme 16:**
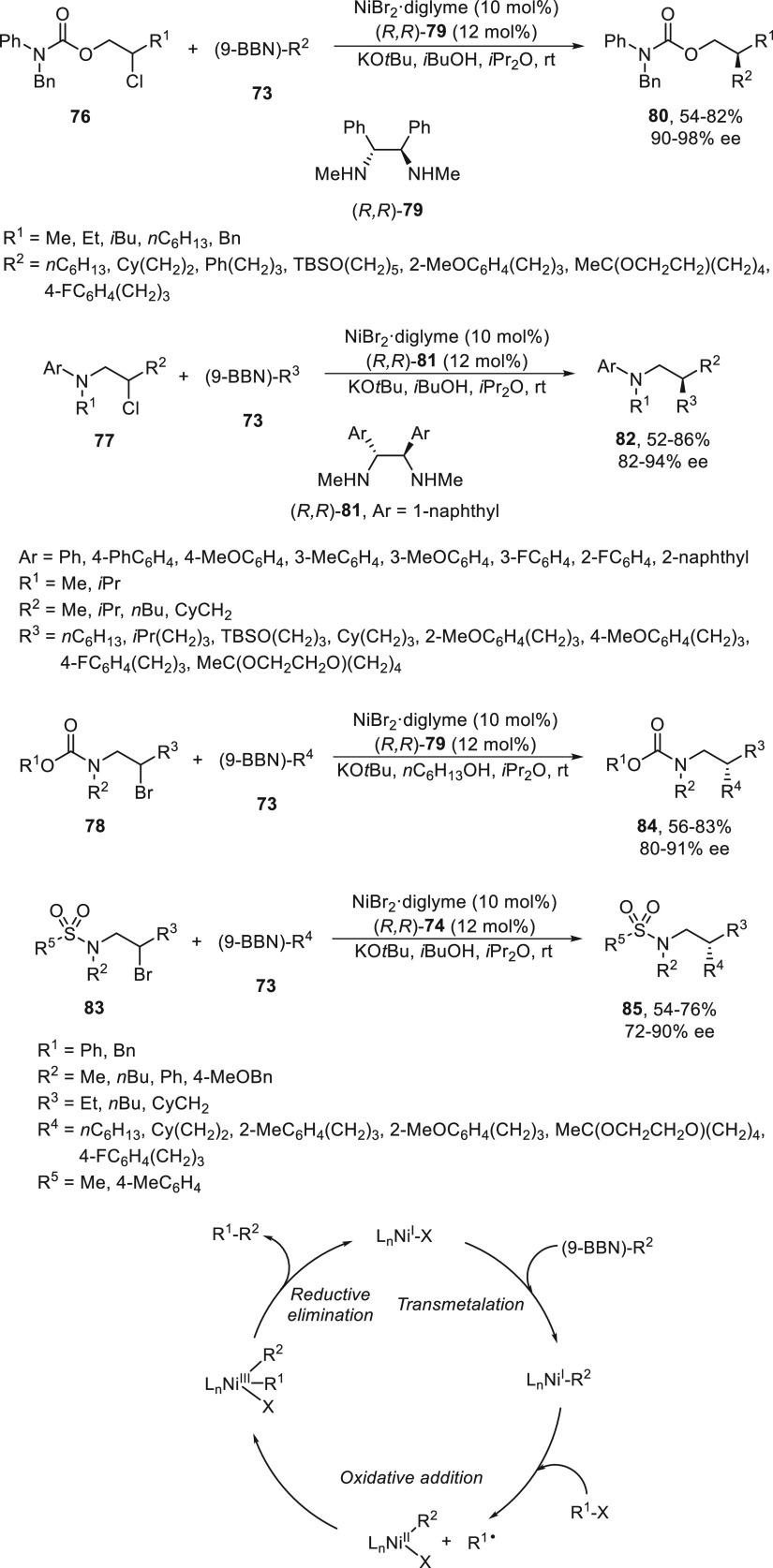
Enantioconvergent Ni-Catalyzed Suzuki Reactions of β-Halogenated Compounds: Reaction of Acylated Halohydrins **76**, β-Chloro Alkylanilines **77**, and *N*-Protected β-Bromoalkylamines **78** and **83** with Alkylboranes **73**

Subsequent studies on enantioconvergent alkyl–alkyl Suzuki reactions by Fu and co-workers were performed with acylated halohydrins **76**,^72^ β-halo alkylanilines **77**,^73^ and *N*-protected β-bromo alkylamines **78**(74) (Scheme 16). In all these cases, the presence of a directing group, which likely interacts with the chiral catalyst, is essential for the high enantioselectivity observed with these unactivated secondary alkyl halides. Acylated halohydrins **76** and alkylboranes **73** reacted in the presence of NiBr_2_/diamine (*R,R*)-**79** as catalyst, which provided products **80** in up to 82% yield and high enantioselectivity (90–98% ee).^72^ β-Chloro alkylanilines **77** reacted with boranes **73** using NiBr_2_/diamine **81** as catalyst to give enantioenriched β-alkylanilines **82** up to 86% yield and up to 94% ee.^73^ In the case of *N*-protected β-bromo alkylamines **78** or **83** bearing a carbonate or a sulfonamide group, respectively, the alkylation with boranes **73** was performed with NiBr_2_ and diamine **79** or **74** as chiral ligands to furnish products **84** or **85**, respectively. Experimental evidence showed that the alkyl group of the organoborane is transferred to the reaction product with retention of the configuration consistent with transmetalation with retention. Therefore, the structural integrity for the Ni–R^2^ bond is maintained during the catalytic cycle depicted in Scheme 16.

**Scheme 17:**
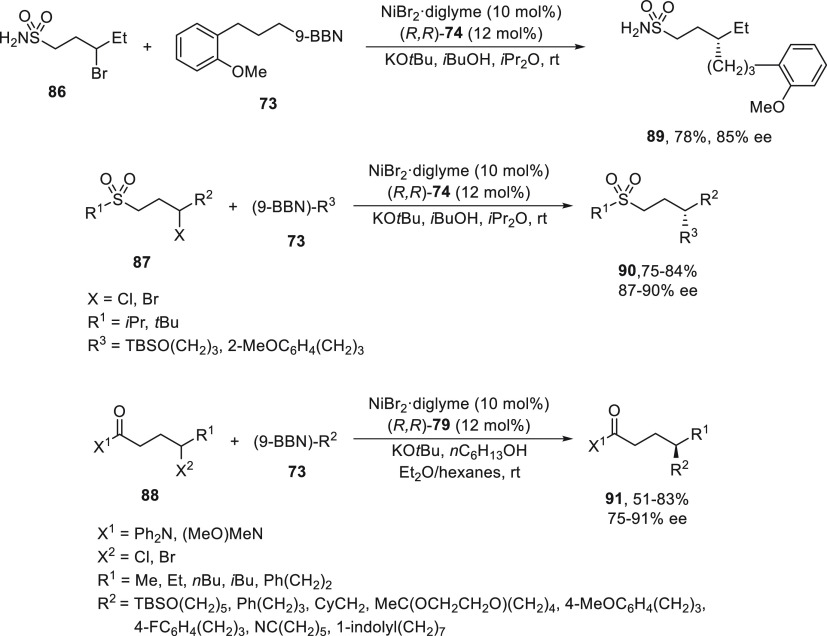
Enantioconvergent Ni-Catalyzed Suzuki Reactions of γ-Halogenated Sulfonamide **86**, Sulfones **87**, and Carboxamides **88** with Alkylboranes **73**

The sulfone group directed the Ni-catalyzed Suzuki reaction at the γ-position of sulfonamide **86** and sulfones **87** under the reaction conditions indicated for sulfonamides **83** (Scheme 16).^74^ This enantioconvergent γ-alkylation took place with γ-halo carboxamides **88** using NiBr_2_/diamine (*R,R*)-**79** as catalyst.^75^ In the case of sulfonamide **86**, product **89** was obtained in 78% yield and 85% ee, and sulfones **87** provided compounds **90** in 75–84% yields and 87–90% ee (Scheme 17).^74^ Carboxamides **88** bearing a bromo or chloro substituents at the γ-position afforded, by reaction with boranes **73**, the corresponding cross-coupling products **91** in good yields and enantioselectivities (Scheme 17).^75^

**Scheme 18:**
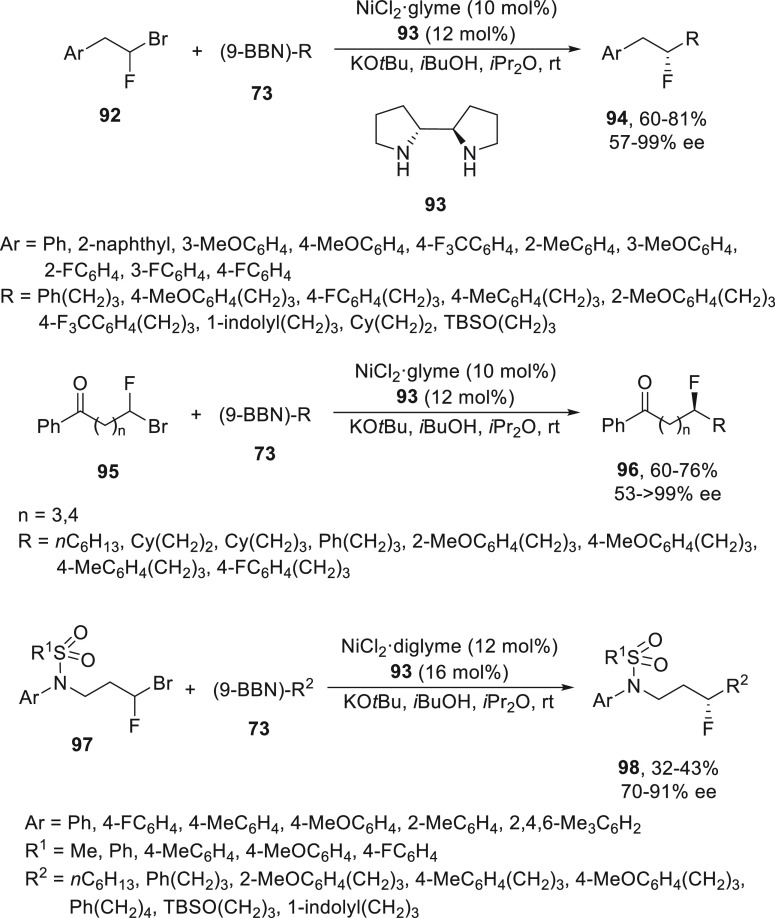
Enantioconvergent Ni-Catalyzed Suzuki Reactions of 1-Halo-1-fluoroalkanes **92**, **95**, and **97** with Alkylboranes **73**

Gandelman and co-workers^76,77^ described the enantioconvergent synthesis of secondary alkyl fluorides by Suzuki cross-coupling of 1-fluoro-1-haloalkanes with alkylboranes **73**. 1-Bromo-1-fluoro-2-arylethanes **92** were alkylated using NiCl_2_/diamine **93** as catalyst to give products **94** up to 81% yield and up to 99% ee (Scheme 18). Under these reaction conditions, fluorobromoalkanes bearing different directing groups, such as ketones **95**, provided chiral δ- and ε-fluoroalkanes **96** after alkylation. 1-Bromo-1-fluoroalkanes bearing a sulfonamide-directing group **97** gave γ-fluorosulfonamides **98** after enantioconvergent cross-coupling with alkylboranes **73** with modest yield and up to 91% ee.

**Scheme 19:**
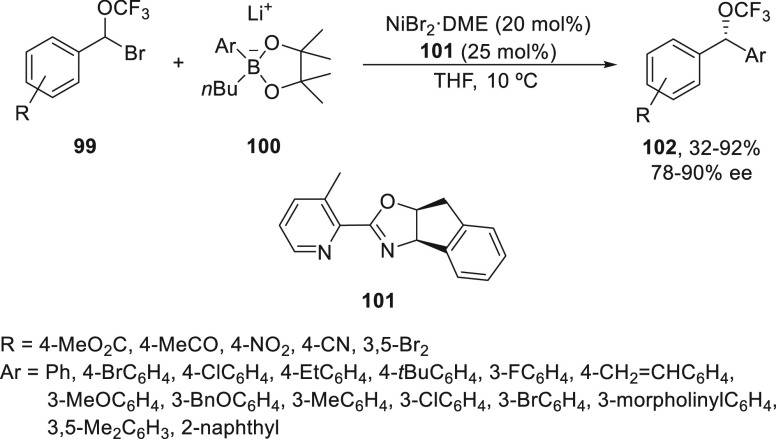
Enantioconvergent Ni-Catalyzed Suzuki Reactions of α-Bromobenzyl Trifluoromethyl Ethers **99** with Aryl Pinacol Boronates **100**

Starting from racemic α-bromobenzyl trifluoromethyl ethers **99**, Shen and co-workers^78^ performed the enantioconvergent cross-coupling with aryl pinacol boronates **100** to form α-trifluoromethoxy-substituted diaryl methanes **102** (Scheme 19). The reaction took place under mild reaction conditions using NiBr_2_/oxazoline **101** as catalyst to afford products **102** with good yields and up to 90% ee. However, other organometals, such as phenylmagnesium bromide or diphenylzinc, gave compounds **102** with lower yields due to the side reactions of these Lewis acidic organometals with products **102**. Several functional groups are tolerated under these reaction conditions, and the reaction can be easily scaled up to grams.

**Scheme 20:**
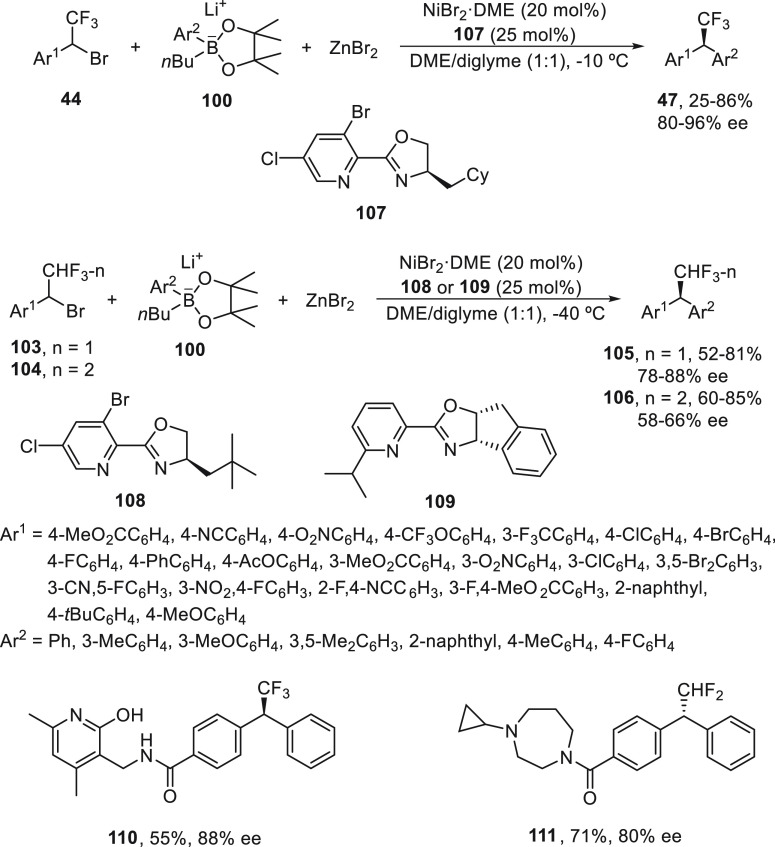
Enantioconvergent Ni-Catalyzed Arylation of Fluorinated Benzylic Bromides **44**, **103**, and **104** with Lithium Organoboronates **100** and ZnBr_2_

The same group^79^ has developed an enantioconvergent acylation of secondary α-bromobenzyl trifluoro-/difluoro-/monofluoromethanes **44**/**103**/**104** with dilithium aryl zincates, [Ar_2_ZnBr]Li, generated *in situ* from lithium organoborates **100** and ZnBr_2_ (Scheme 20). The use of lithium aryl zincates facilitates the transmetalation step of this nickel-catalyzed cross-coupling reaction, thereby allowing the synthesis of enantioenriched benzhydryl fluoroalkene derivatives **47**, **105**, and **106** using NiBr_2_·DME and a pyridine-oxazoline ligand **107** for trifluoromethyl substrates **44**, **108** for difluoromethyl compounds **103**, and ligand **109** for fluoromethyl reagents. This procedure was applied to the synthesis of **110**, a trifluoromethylated mimic of an inhibitor for the histone lysine methyltransferase enhancer of zeste homologue 2 (EZH2)^80^ in 55% overall yield and with 88% ee. A difluoromethylated compound **111**, which is a mimic of histamine H3 receptor,^81^ was prepared in 71% overall yield and with 80% ee.

**Scheme 21:**
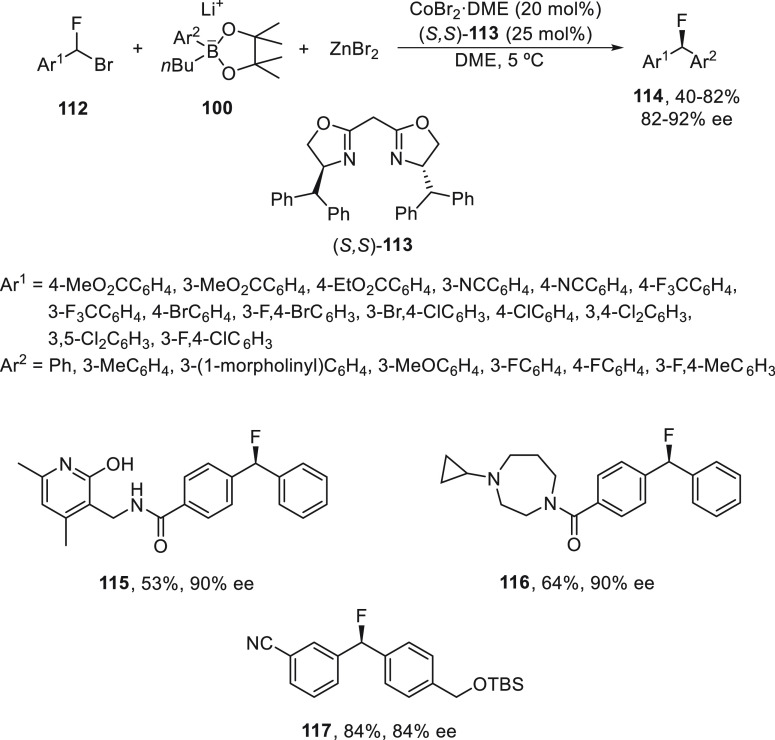
Enantioconvergent Co-Catalyzed Arylation of Fluorinated Secondary Benzyl Bromides **112** with Lithium Aryl *n*-Butyl Pinacol Boronates **100** and ZnBr_2_

For the cross-coupling of fluorinated secondary benzyl bromides **112**, Shen and co-workers^82^ also employed *in situ* generated lithium aryl zincates but under cobalt catalysis. In this case, CoBr_2_·DME and bis(oxazoline) (*S*,S)-**113** were used as chiral catalysts under mild reaction conditions to provide fluorinated diarylmethane derivatives **114** in up to 92% ee (Scheme 21). This methodology was applied to the synthesis of compounds **115** and **116**, which are fluorinated mimics of EZH2,^80^ respectively. Compound **117**, a key intermediate of a fluorine-substituted analogue of Lilly’s mGlu 2 receptor potentiators, which is a compound for the treatment of migraine headaches,^83^ was prepared in 84% yield with 84% ee.

**Scheme 22:**
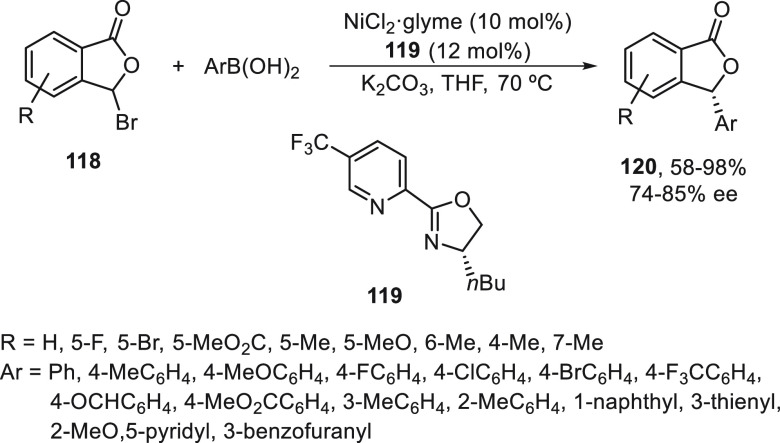
Enantioconvergent Ni-Catalyzed Suzuki Reaction of 2-Bromophthalides **118** with Arylboronic Acids

Enantioconvergent Suzuki reactions of 3-bromophthalides **118** with arylboronic acids was recently reported by Zhang, Feng, and co-workers.^84^ Cross-coupling with arylboronic acids took place using NiCl_2_/oxazoline **119** as catalyst and K_2_CO_3_ as base in THF at 70 °C to give chiral 3-arylphthalides **120** with good yields and up to 85% ee (Scheme 22).

**Scheme 23:**
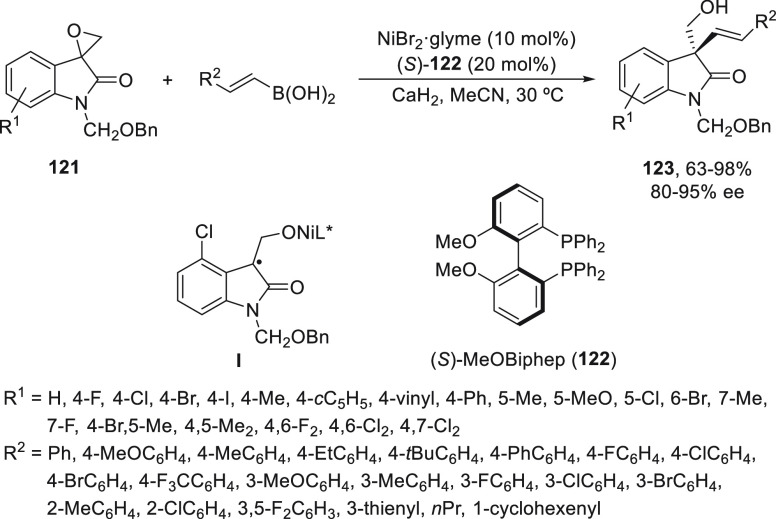
Enantioconvergent Ni-Catalyzed Suzuki Reactions of Spiroepoxyindoles **121** with Alkenylboronic Acids

In general, the alkyl electrophiles of these Suzuki reactions are secondary, which give rise to chiral tertiary carbon stereocenters. Recently, Zhang and co-workers^85^ reported a Ni-catalyzed enantioconvergent coupling of epoxides **121** with alkenylboronic acids. These racemic spiroepoxyindoles **121** afforded chiral oxindoles **123** bearing quaternary carbon stereocenters using NiBr_2_/(*S*)-MeOBiphen (**122**) as catalyst (Scheme 23). In this case, CaH_2_ was used as base, and MeCN was used as solvent at 30 °C to give products **123** with good yields and enantioselectivities. It has been proposed that the formation of a stabilized tertiary radical intermediate **I** by a single-electron transfer mechanism is formed during the oxidative addition step.

**Scheme 24:**
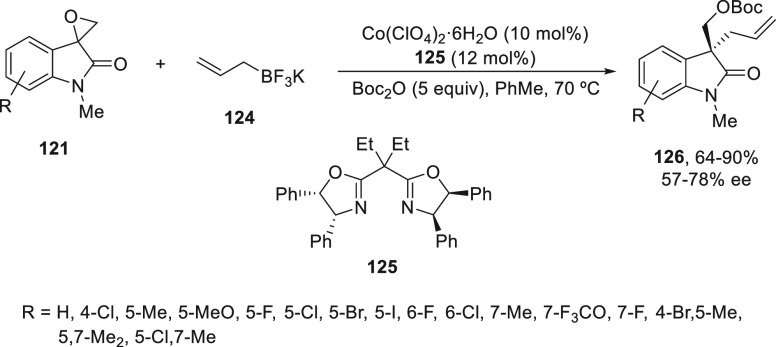
Enantioconvergent Co-Catalyzed Suzuki Reactions of Spiroepoxyoxindoles **121** with Potassium Allyltrifluoroborate **124**

The same group performed this ring-opening reaction of spiroepoxyindoles **121** with allylboron reagents under Co(II) catalysis.^86^ Potassium allyltrifluoroborate (**124**) reacted with epoxides **121** using Co(ClO_4_)_2_/bis(oxazoline) **125** in the presence of di-*tert*-butyl dicarbonate (Boc_2_O) in order to avoid the coordination of the alcohol from the ring-opening product with the chiral catalyst. Chiral oxindoles **126** were obtained with yields of 64–90% and up to 78% ee (Scheme 24).

**Scheme 25:**
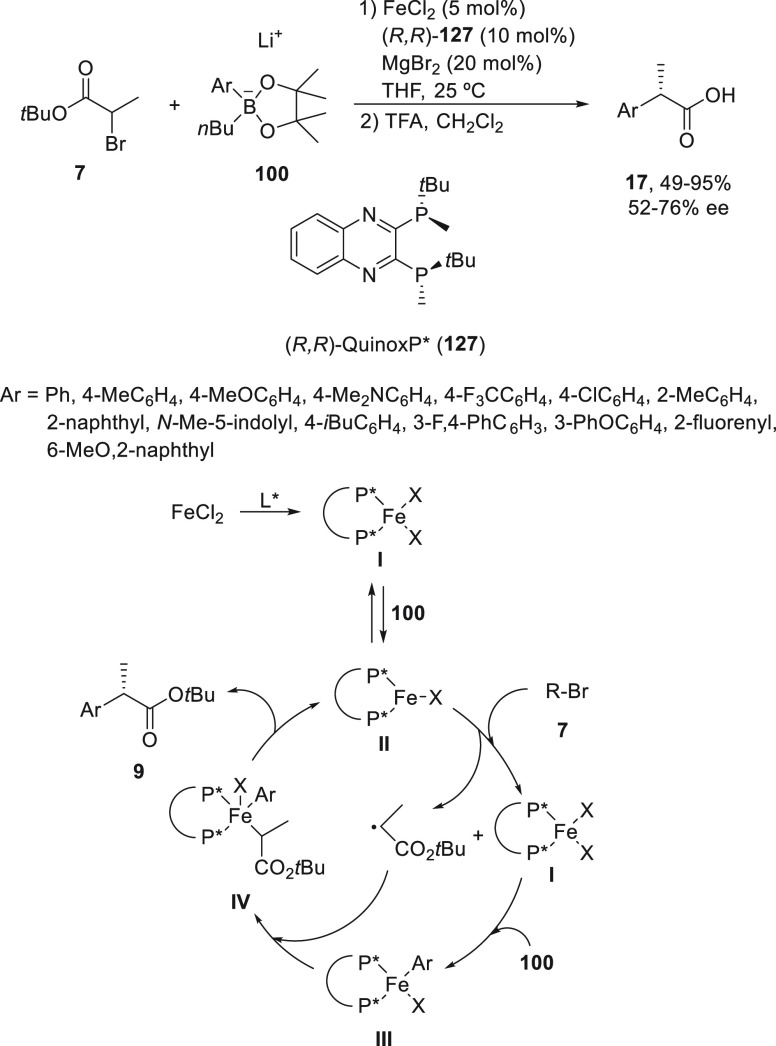
Enantioconvergent Fe-Catalyzed Suzuki Reaction of *t*-Butyl α-Bromopropionate **7** with Lithium Aryl Pinacol Boronates **100**

Nakamura and co-workers^87^ have described the enantioconvergent iron-catalyzed Suzuki reaction of *tert*-butyl α-bromopropionates **7**, which was previously described with Grignard reagents^43^ (Scheme 4). In this case, lithium aryl pinacol boronates **100** were used as coupling partners, and FeCl_2_/(*R,R*)-QuinoxP* (**127**) was used as catalyst to provide, after TFA hydrolysis of the corresponding esters **9**, enantioenriched α-arylpropionic acids **17** with good yields and moderate enantioselectivities (Scheme 25). As mentioned before, α-arylpropionic acids are well-known nonsteroidal anti-inflammatory drugs (NSAIDs). A plausible mechanism based on experimental and theoretical studies is depicted in Scheme 25. Transmetalation of complex **I** (LFeCl_2_) with the boron reagent and subsequent reductive elimination gave the active species **II**. This intermediate abstracts the bromine atom from compound **7** to generate the corresponding alkyl radical, which recombines with complex **III** generated by transmetalation of **I** with the boron reagent **100** to produce intermediate **IV**. Final reductive elimination of complex **IV** affords the expected product.

**Scheme 26:**
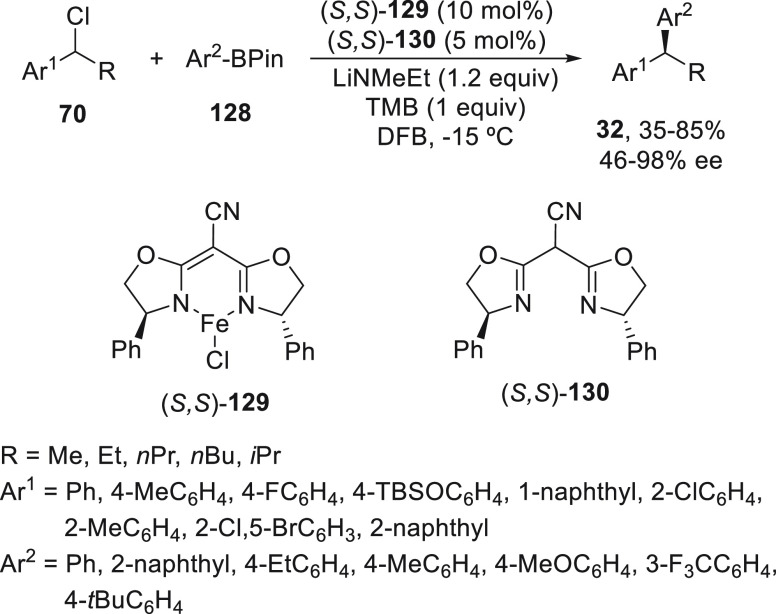
Enantioconvergent Iron-Catalyzed Suzuki Reactions of Benzylic Chlorides **70** with Arylboronic Pinacol Esters **128**

Enantioenriched 1,1-diarylalkanes **32** have been prepared by enantioconvergent Suzuki reaction of benzylic chlorides **70** with arylboronic pinacol esters **128** using cyano[bis(oxazoline)]iron(II) chloride complex **129** and the ligand **130** as catalyst (Scheme 26).^88^ This cross-coupling took place under mild reaction conditions in the presence of 1,3,5-trimethoxybenzene (TMB) as a stoichiometric additive, LiNMeEt as base, and 1,2-difluorobenzene (DFB) as solvent at −15 °C. Products **32** were obtained with moderate to good yields and enantioselectivities.

**Scheme 27:**
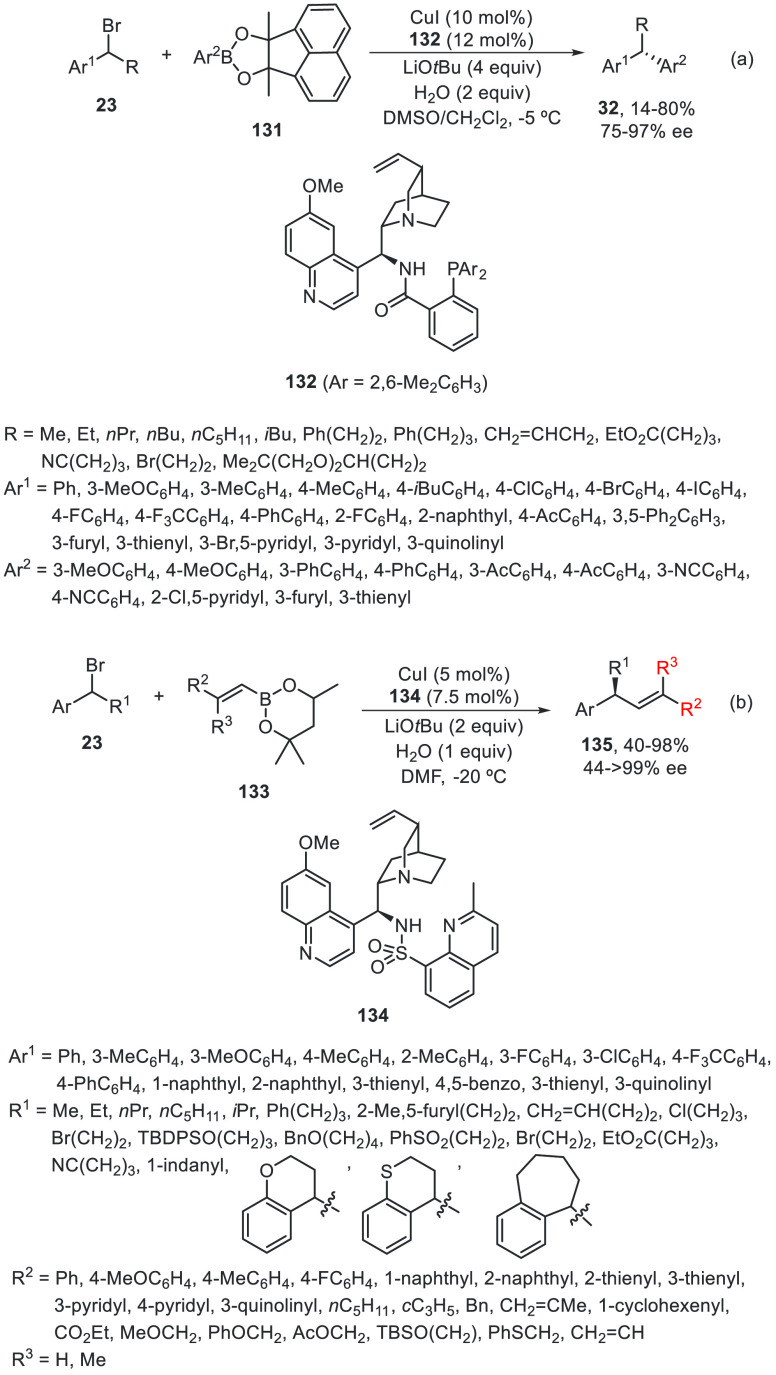
Enantioconvergent Cu-Catalyzed Suzuki Reactions of Benzylic Bromides **23** with Aryl and Alkenyl Boronates **131** and **133**, Respectively

Li, Liu, and co-workers^89,90^ have developed the Cu-catalyzed enantioconvergent radical Suzuki C(sp^3^)–C(sp^2^) cross-coupling. Benzylic bromides **23** have been allowed to react with cyclic B(mac)-derived boronate esters **131** using CuI/N,N,P-ligand **132** as the catalyst to provide enantioenriched 1,1-diarylalkanes **32** in up to 80% yield and 97% ee (Scheme 27a).^89^ When alkenyl methylpentanediol(mp)-derived boronate esters **133** were used as organometals for the Suzuki cross-coupling with benzylic bromides, CuI/N,N,N-ligand **134** was the best catalyst and gave products **135** in up to 98% yield and up to >99% ee (Scheme 27b).^90^ In this hemilabile N,N,N-ligand **134**, the presence of a methyl group at the *ortho* position of the sulfonamide quinoline moiety increases the enantioselectivity by steric hindrance probably by elongation of the Cu–N bond. These cross-couplings were also performed with propargyl bromides (see Section 4). Mechanistic studies revealed a radical process depicted in Scheme 27. The Cu^I^ complex undergoes a transmetalation process with the boronate ester to give intermediate **I**, which undergoes a single electron reduction with the benzylic bromide to deliver a radical **II** and the Cu^II^ complex **III**. Final reaction of these intermediates **II** and **III** forms the coupling product and regenerates the catalyst. In the case of alkenylboronates cross-coupling, DFT calculations supported tentatively the favorable transition state (TS) to explain the absolute configuration of the products.

**Scheme 28:**
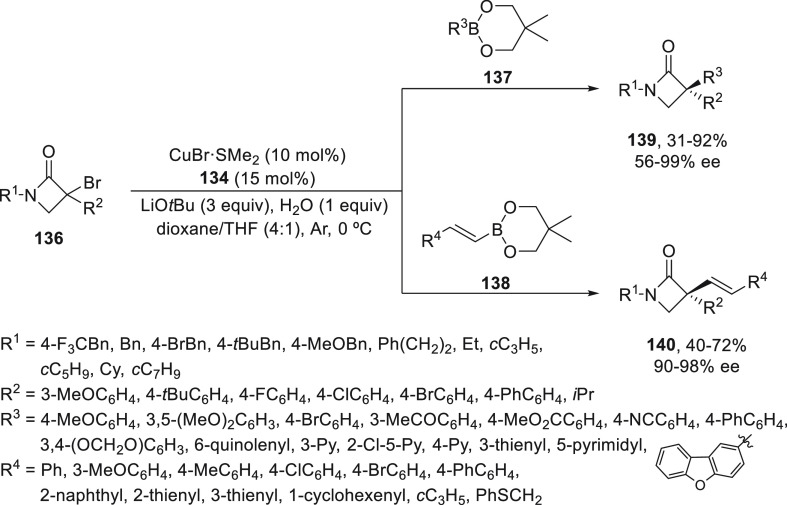
Enantioconvergent Cu-Catalyzed Suzuki Reaction of α-Bromo-β-lactams **136** with Aryl and Alkenyl Boronates **137** and **138**, Respectively

Recently, the same group^91^ performed a copper-catalyzed enantioconvergent radical C(sp^3^)–C(sp^2^) cross-coupling of α-bromo-β-lactams **136** with aryl and alkenyl organoboronate esters **137** and **138** (Scheme 28). In this case, the hemilabile N,N,N-ligand **134** gave products **139** and **140** with a quaternary stereocenter in up to 99% ee. The best results were obtained with neopentyl glycol (neop)-derived aryl-derived aryl and alkenyl boronate esters in the LiO*t*Bu (3 equiv)/H_2_O (1 equiv) system at 0 °C under an argon atmosphere and using a 4/1 mixture of 1,4-dioxane/THF. Experimental radical trap experiments corroborate the formation of an alkyl radical, which is formed because of the enhanced reducing capability of the Cu(I) catalyst bearing this type of electron-donating ligand.

Alkyl organoboranes reacted with unactivated secondary alkyl bromides and chlorides bearing a directing group at the α- to ε-position under Ni/chiral diamines catalysis. Boronates and boronic acids have been used for C(sp^3^)–C(sp^2^) bond-forming reactions with oxazolines or diphosphines as chiral catalysts. Cobalt, iron, and copper complexes catalyzed the cross-coupling of epoxides and activated bromides with boronates. In all cases, enantioconvergent transformations take place under mild reaction conditions by intermediacy of a radical derived from the electrophile.

#### 2.1.4 Organosilicon Reagents

**Scheme 29:**
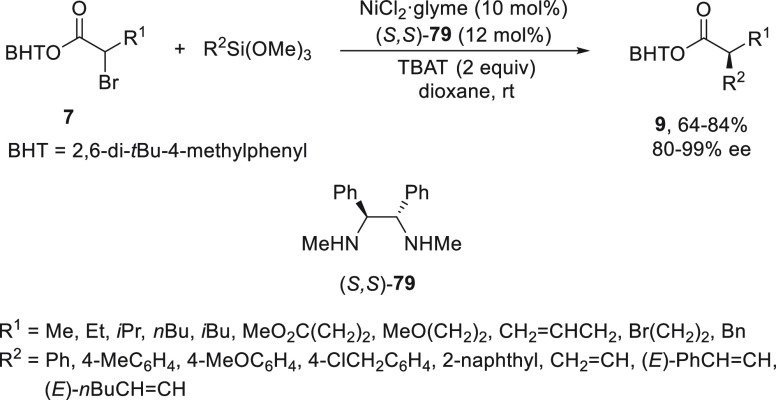
Enantioconvergent Ni-Catalyzed Hiyama Reactions of α-Bromo Esters **7** with Aryl or Alkenylsiloxanes

An enantioconvergent nickel-catalyzed Hiyama cross-coupling reaction can be performed with activated 2,6-di-*tert*-butyl-4-methylphenyl (BHT) α-bromo esters **7** and organosilanes. Fu and co-workers^92^ reported this C(sp^3^)–C(sp^2^) bond formation using NiCl_2_/diamine (*S,S*)-**79** as catalyst and aryl or alkenyltrimethoxysilanes to give esters **9** with good yields and up to 99% ee (Scheme 29). This Hiyama reaction took place at room temperature in dioxane promoted by tetrabutylammonium triphenyldifluorosilicate (TBAT) as fluoride activator.

**Scheme 30:**
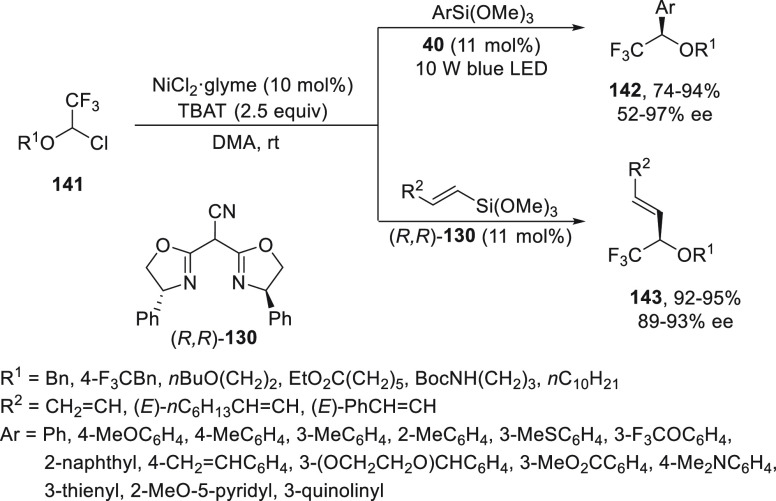
Enantioconvergent Ni-Catalyzed Hiyama Reactions of α-Chloro-α-trifluoromethyl Methyl Ethers **141** with Aryl or Alkenyl Siloxanes

Varenikov and Gandelman^93^ applied the enantioconvergent Hiyama reaction for the synthesis of enantioenriched α-trifluoromethyl ethers **142** and **143**, precursors of α-trifluoromethyl alcohols. Cross-couplings of different α-chloro-α-trifluoromethyl methyl ethers **141** with aryl siloxanes were performed under NiCl_2_/bis(oxazoline) **40** catalysis and irradiated in darkness with a white or blue LED lamp, which significantly accelerated the process, to provide compounds **142** with very good yields and up to 98% ee (Scheme 30). When alkenyl siloxanes were used as nucleophiles, bis(oxazoline) (*R,R*)-**130** gave compounds **143** in up to 93% ee. The authors presume that the photoinduction facilitates the oxidative addition of electrophile **141** to an excited Ni(I) catalytic species, which likely takes place by radical mechanism, as has been proposed by Fu and co-workers.^94^ In the case of alkyl ethers, the arylation needed longer reaction times. Presumably, the oxidative addition is the rate-limiting step, and the lower the LUMO of the electrophile, the faster the single-electron transfer (SET) from the catalyst occurs.

Enantioconvergent Hiyama reactions can be performed under Ni catalysis with diamines or bis(oxazolines) as chiral ligands and TBAT as fluoride activator. Activated alkyl bromides and chlorides underwent cross-coupling reaction with aryl or alkenyl siloxanes.

#### 2.1.5 Other Organometals

**Scheme 31:**
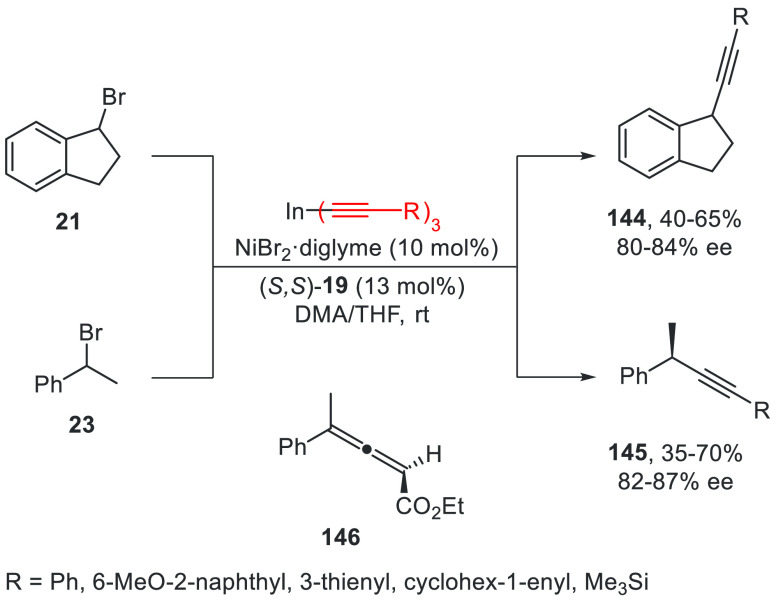
Enantioconvergent Ni-Catalyzed Cross-Coupling Reactions of Secondary Benzyl Bromides **21** and **23** with Alkynylindium Reagents

In 2008, Sarandeses and co-workers^95^ reported the enantioconvergent Ni-catalyzed cross-coupling reaction of trialkynylindium reagents with secondary benzylic bromides **21** and **23** using (*S,S*)-*i*Pr-Pybox (**19**) as a chiral ligand (Scheme 31). This alkynylation reaction took place in moderate to good yields and up to 87% ee working at room temperature during 140 h and in a 1:1 mixture of DMA/THF. In the case of products **144**, the absolute configuration was not determined. Compounds **145** were obtained without isomerization of the triple bond except in the case of ethyl propiolate, which afforded the allene **146** in 30% yield and 77% ee.

**Scheme 32:**
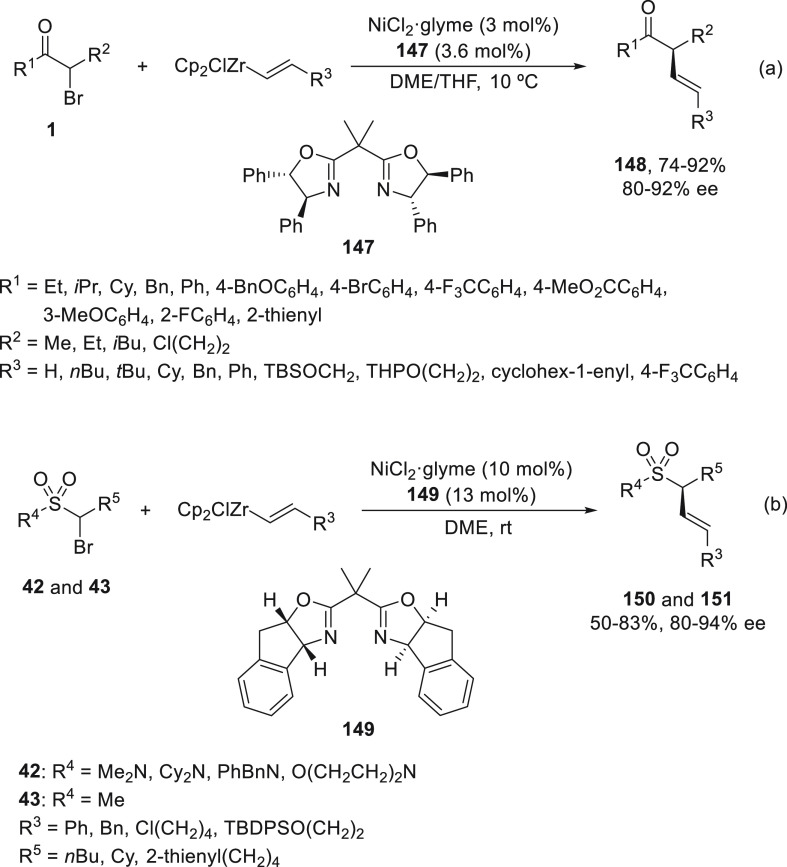
Enantioconvergent Ni-Catalyzed Cross-Coupling Reactions of α-Bromo Ketones **1** and α-Bromo Sulfonamides **42** and Sulfones **43** with Alkenylzirconium Reagents

Enantioconvergent and stereospecific Ni-catalyzed alkenylations with organozirconium reagents were described by the Fu group.^54,96,97^ Initial results were carried out with activated secondary alkyl bromides **1** using (−)-bis(oxazoline) **147** as a chiral ligand under smooth reaction conditions. α-Substituted β,γ-unsaturated ketones **148** were obtained in very good yields and ee with low catalyst loading to keep the (*E*)-configuration of the starting alkenylzirconium reagent (Scheme 32a). This alkenylation was applied to the cross-coupling of α-bromo sulfonamides **42** and sulfone **43** (R^4^ = Me; R^5^ = Cy) with alkenylzirconium reagents using, in this case, ligand **149** to furnish allylic sulfonamides **150** and sulfone **151**, respectively (Scheme 32b). In both cases, bis(oxazoline) **149** was the suitable chiral to give the corresponding products in good yields and enantioselectivities.

**Scheme 33:**
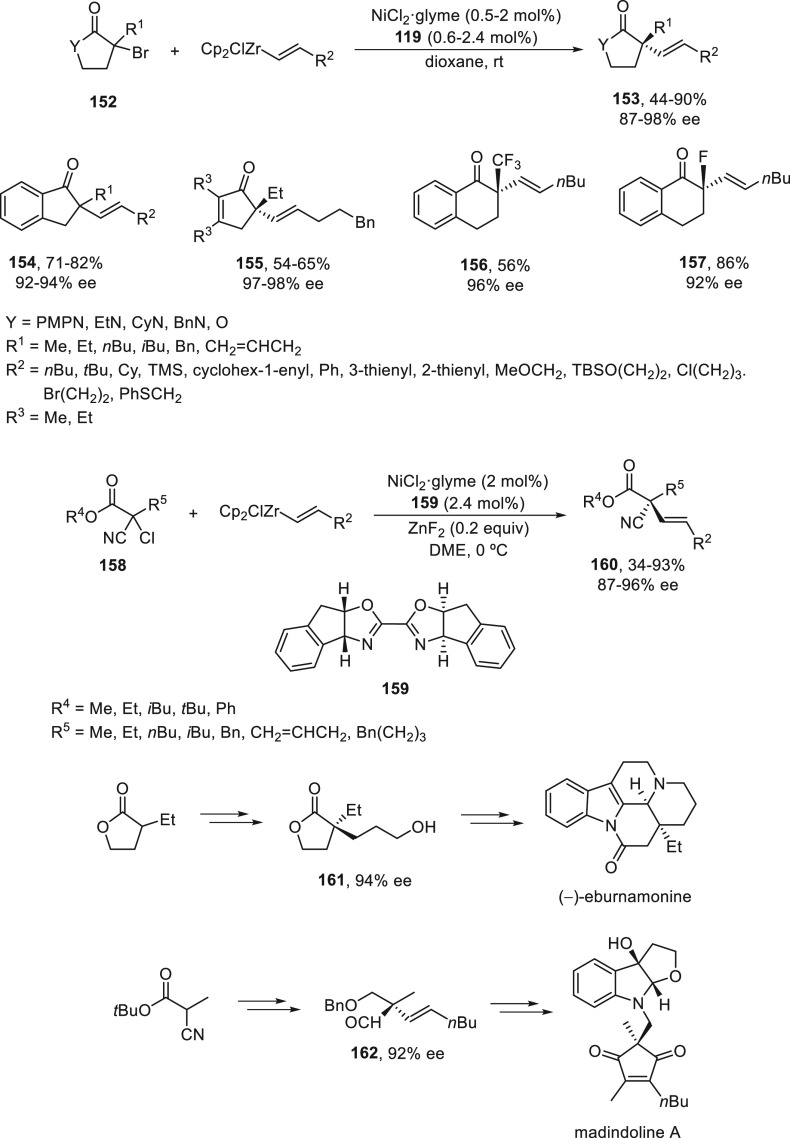
Enantioconvergent Ni-Catalyzed Cross-Coupling Reactions of Tertiary Alkyl Halides **152** and **158** with Alkenylzirconium Reagents

Recently, alkenylzirconium reagents have been used as appropriate nucleophiles for the enantioconvergent challenging alkenylation of activated tertiary alkyl halides.^97^ Cyclic and acyclic tertiary α-halo carbonyl compounds **152** and **158** reacted with alkenylzirconium reagents to afford enantioenriched α,α-disubstituted products **153**–**157** and **159**, respectively (Scheme 33). For cyclic systems **152**, NiCl_2_/oxazoline **119** was used as catalyst to provide α-alkenylated products **153**–**157** in good yields and enantioselectivities. In the case of α-chloro-α-cyano esters **158**, NiCl_2_/bis(oxazoline) **159** was the preferred catalyst, and ZnF_2_ (0.2 equiv) was used as additive to afford product **160**, also with good yields and enantioselectivities. As in the case of secondary halides, mechanistic experimental studies suggested the formation of radical intermediates. This method was applied to the formal total synthesis of bioactive natural products, such as (−)-eburnamonine and madindoline A, through the corresponding key intermediates **161** and **162**, respectively. Lactone **161** was prepared from racemic 3-ethyloxolan-2-one in four steps with 94% ee, which was previously transformed into the alkaloid (−)-eburnamonine.^98^ Madindoline A, an inhibitor of interleukin 6, has been previously prepared from aldehyde **162**,^99^ which was prepared in five steps in 92% ee starting from *tert*-butyl α-cyano propionate.

**Scheme 34:**
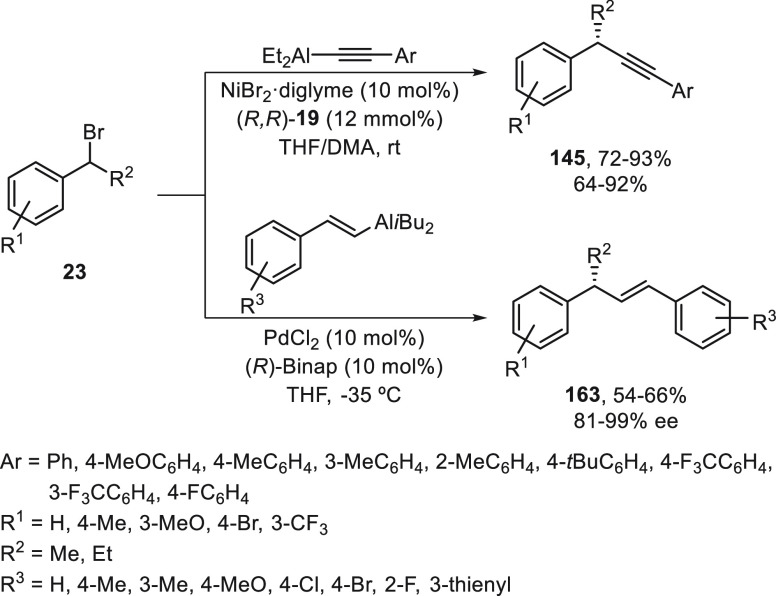
Enantioconvergent Ni- and Pd-Catalyzed Cross-Coupling of Secondary Benzylic Bromides **23** with Alkynyl and Alkenylaluminum Reagents, Respectively

Alkenyl and alkynylaluminum reagents have been employed as nucleophiles in enantioconvergent cross-coupling reactions with secondary benzylic bromides **23** by Zhou and co-workers.^100^ The alkynylation reaction took place using NiBr_2_/(*R,R*)-*i*Pr-Pybox (**19**) as catalyst in a 1:1 mixture of THF/DMA at room temperature to give products **145** with good yields and enantioselectivities (Scheme 34). However, the alkenylation reaction was carried out under Pd catalysis using (*R*)-Binap as chiral ligand in THF at −35 °C to provide enantioenriched aryl alkenes **163** with moderate yields and up to 99% ee.

**Scheme 35:**
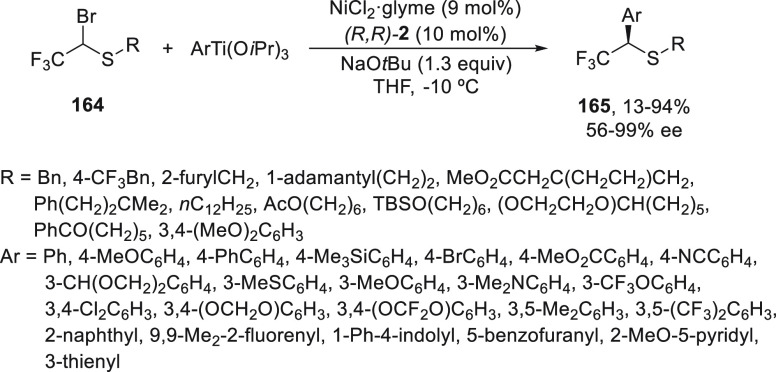
Enantioconvergent Ni-Catalyzed Cross-Coupling Reactions of α-Trifluoromethyl-α-bromomethyl Thioethers **164** with Aryltitanium(IV) Reagents

Organotitanium reagents possess a lower nucleophilicity than organomagnesium ones, which allows a greater functional group tolerance, and have higher transmetalation rates than organozinc compounds. Varenikov and Gandelman^101^ employed for the first time these organometals in asymmetric cross-coupling reactions devoted to the synthesis of enantioenriched α-trifluoromethyl thioethers **165**. Initial studies about the enantioconvergent Ni-catalyzed cross-coupling reaction of α-trifluoromethyl-α-bromomethyl thioethers **164** with aryltrimethoxysilanes gave poor results. However, aryltianium(IV) compounds, prepared by mixing Ti(O*i*Pr)_4_ with arylmagnesium bromides in the presence of NaO*t*Bu, formed titanate complexes able to react with thioethers **164** using NiCl_2_/PhBox [(*R,R*)-**2**] as catalyst in THF at −10 °C to provide a wide range of products **165** in moderate to good yields and high enantioselectivities (Scheme 35).

In summary of this Section 2.1 about enantioconvergent cross-coupling reactions of alkyl electrophiles with organometals, organozinc or organoboron reagents have been largely employed mainly under Ni catalysis and oxazolines or 1,2-diamines as chiral ligands, respectively. For alkenylation reactions, alkenylzirconium reagents using Ni/bis(oxazoline) as catalyst gave the best results, even with tertiary alkyl bromides. In the case of alkynylation reactions of benzylic bromides, alkynylindium and aluminum reagents under Ni catalysis have been successfully used.

### 2.2 Racemic Alkyl Electrophiles with Other Non-Metallic Nucleophiles

In this Section, enantioconvergent alkylation with alkyl halides of heteroatom-based nucleophiles, such as amines, other carbon nucleophiles, and borylations, mainly under Cu catalysis, will be considered.

#### 2.2.1 Nitrogen Nucleophiles

**Scheme 36:**
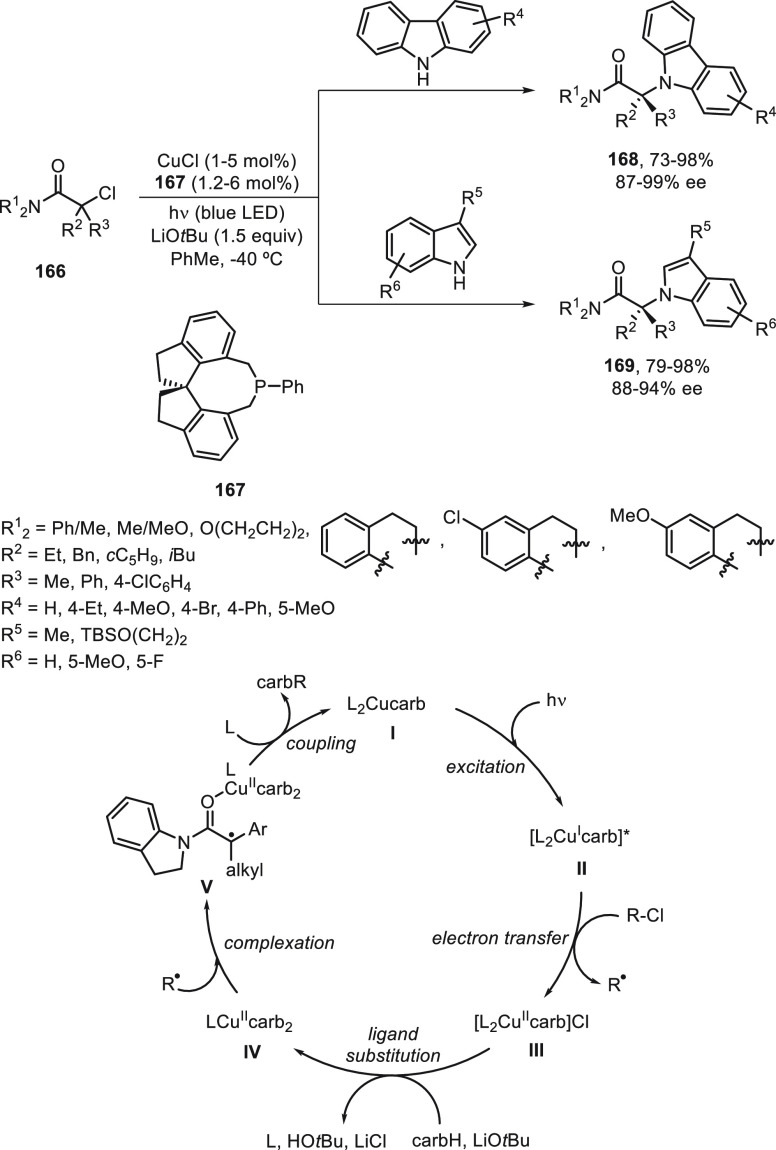
Enantioconvergent Photoreduced Cu-Catalyzed *N*-Alkylation of Carbazoles and Indoles with α-Chloro Amides **166**

Direct substitution reactions of alkyl electrophiles by nitrogen nucleophiles via S_N_1 or S_N_2 processes suffer from many limitations with regard to scope and/or stereoselectivity. Catalytic processes were developed for aryl electrophiles, such as the copper-catalyzed Ullmann reaction and the palladium-catalyzed Buchwald-Hartwig reaction, that achieved a broad scope for C–N formation. The coupling of alkyl halides with amines was achieved by Peters, Fu, and co-workers^102^ under the combined action of light and copper catalysis. Then, the same group^34,103,104^ developed challenging enantioconvergent *N*-alkylations by racemic secondary and tertiary alkyl halides in the presence of light and a chiral copper catalyst. Initial studies were performed with tertiary α-chloro amides **166** and carbazoles and indoles as nitrogen nucleophiles using CuCl/monodentate phosphine **167**, LiO*t*Bu as base, and visible light irradiation (blue LED) in toluene at −40 °C (Scheme 36).^103^ The resulting *N*-alkylated carbazoles and indoles **168** and **169** were obtained in good yields and enantioselectivities. Experimental and theoretical studies support the proposed catalytic cycle for this enantioconvergent *N-*alkylation.^104^ Two key intermediates, the copper(II) metaloradical **IV** and the tertiary α-amide organic radical R^•^, have been characterized by EPR and DFT calculations. These two radicals are combined to furnish the C–N coupling in 77% yield and 55% ee. DFT calculations reckon that the organic radical is resistant to radical–radical homocoupling and, therefore, accessible as a free radical in solution. In this detailed pathway, the previously characterized complex **I** serves as a photoreductant via excitation to **II**, which reacts with the electrophile to give the radical R^•^ and intermediate **III**. After ligand substitution by a second carbazolide ligand and loss of one ligand (L), the characterized intermediate **IV** is formed. Coordination of the radical R^•^ with **IV** gives intermediate **V**, which forms the product regenerating the complex **I** upon binding L.

**Scheme 37:**
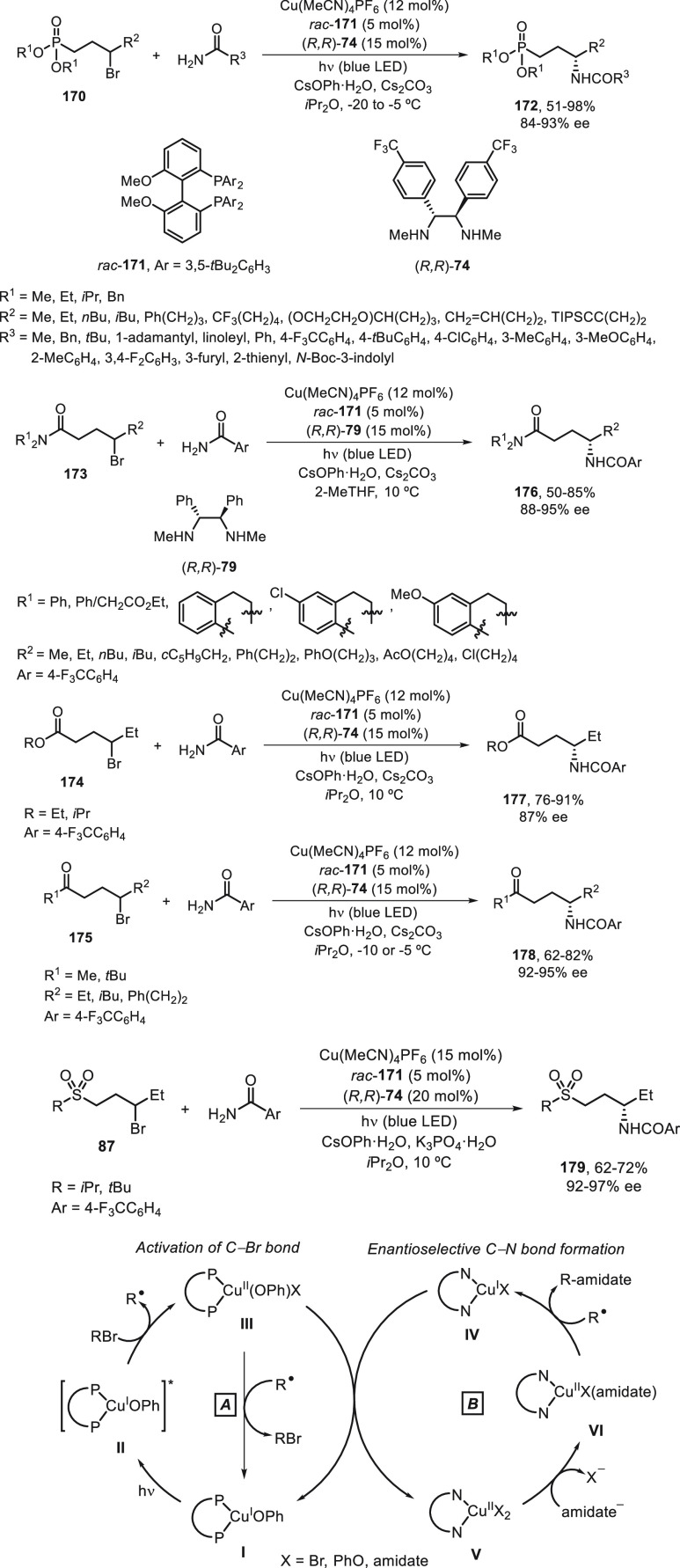
Enantioconvergent Photoinduced Cu-Catalyzed *N*-Alkylation of Carboxamides with γ-Bromo Phosphonates **170**, Amides **173**, Esters **174**, Ketones **175**, and Sulfones **87**

Photoinduced copper-catalyzed amidation of unactivated secondary alkyl halides was initially performed by Peters, Fu, and co-workers with carboxamides^105^ and carbamates.^94^ The same group performed an enantioconvergent amidation of racemic secondary alkyl bromides.^106^ They used a photoinduced copper-catalyzed asymmetric amidation via ligand cooperativity on the basis of three different ligands: a racemic bisphosphine **171**, cesium phenoxide, and a chiral diamine (*R,R*)-**79** or (*R,R*)-**74**. These ligands assemble *in situ* to form two distinct catalysts that act cooperatively: a copper/bisphosphine/phenoxide complex **I**, which serves as photocatalyst, and a chiral copper diamine complex that catalyzes enantioselective C–N bond formation. Alkyl bromides bearing a phosphonoyl group **170** gave γ-aminophosphonic acid derivatives **172** by reaction with carboxamides upon irradiation of the copper-based catalytic system with blue LED lamps in *i*Pr_2_O at −20 to −5 °C (Scheme 37). Other alkyl bromides **173**–**175** and **87** bearing Lewis basic functional groups, including γ-bromo amide **173**, ester **174**, ketone **175** and sulfone **87**, provided good enantioselectivity in the nucleophilic substitution reactions and led to products **176**–**179**. The proposed catalytic cycles ***A*** and ***B*** are based on experimental studies. In cycle ***A***, the photoredox catalyst **I** gives upon irradiation the excited state of **171**·Cu^I^OPh **II** with a sufficient lifetime to react with the electrophile by an inner-sphere electron-transfer pathway (halogen-atom transfer) to afford the radical R^•^ and intermediate **III**. Cycle ***A*** intersects with cycle ***B*** by reaction of intermediate **III** with the chiral copper complex **IV** to generate **I** and **V** by ligand exchange. Then, a nucleophilic substitution of complex **V** with the amidate anion leads to complex **VI**, which reacts with the organic radical R^•^ in an out-of-cage process via coordination of the directing group to Cu^II^ followed by C–N bond formation to furnish the product and complex **IV**.

**Scheme 38:**
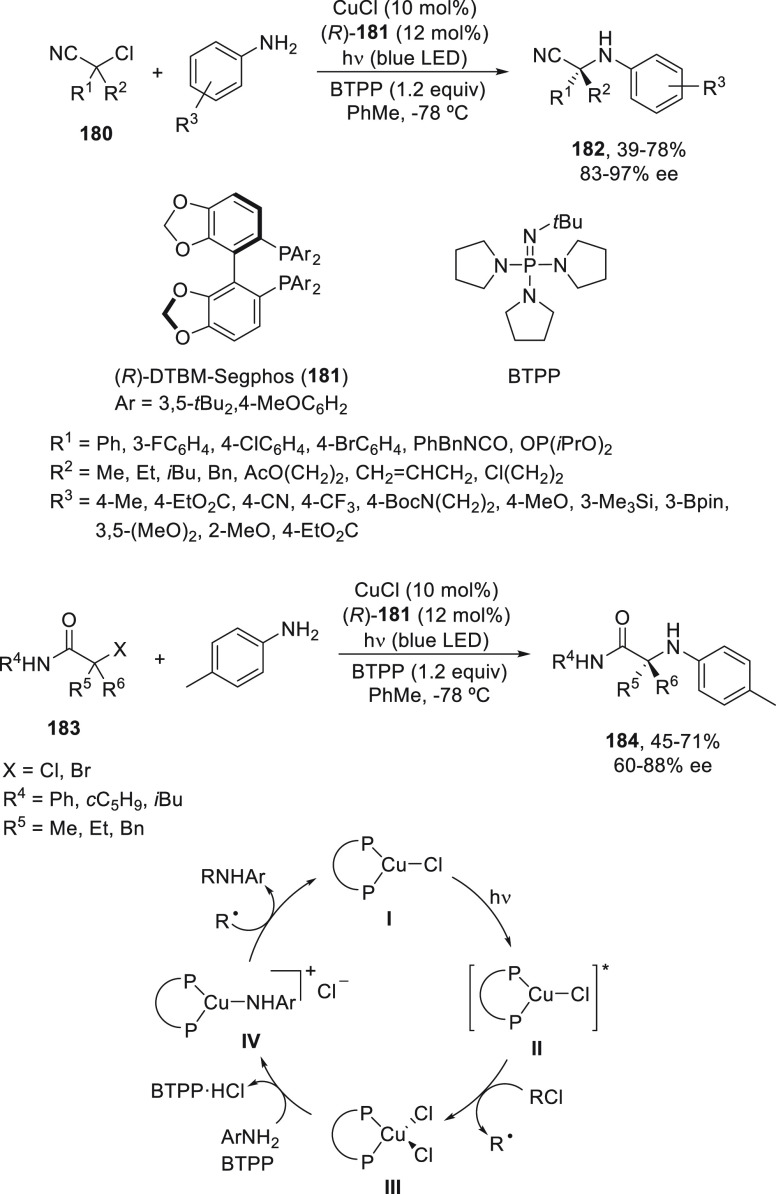
Enantioconvergent Photoinduced Cu-Catalyzed *N*-Alkylation of Anilines with α-Chloro Nitriles **180** and α-Halo Carboxamides **183**

Recently, the same group^107^ reported the photoinduced copper-catalyzed enantioconvergent alkylation of anilines by activated tertiary α-chloro nitriles **180** (Scheme 38). This substitution reaction took place using CuCl/(*R*)-DTBM-Segphos (**181**) as chiral photocatalyst and *tert*-butylimino-tri(pyrrolidino)phosphane (BTPP) as base in toluene at −78 °C to provide enantioenriched α-amino nitriles **182** with moderate to poor yields and up to 97% ee. Tertiary α-chloro or α-bromo amides **183** gave the corresponding α-amino amides **184** under the same reaction conditions with 45–71% yield and up to 88% ee. Experimental and theoretical studies led to identification of copper-based intermediates, such as the photoreductant L*CuCl (**I**) and [L*Cu(NHAr)]Cl (**IV**) as key intermediates. In the proposed catalytic cycle, catalyst **I** gave intermediate **II** upon radiation, which abstracts a chlorine atom from the alkyl chloride to form the radical R^•^ and intermediate **III**. By reaction with the anilido anion intermediate, **IV** is formed, which reacts with the radical to give the product and regenerates the catalytic complex **I**.

**Scheme 39:**
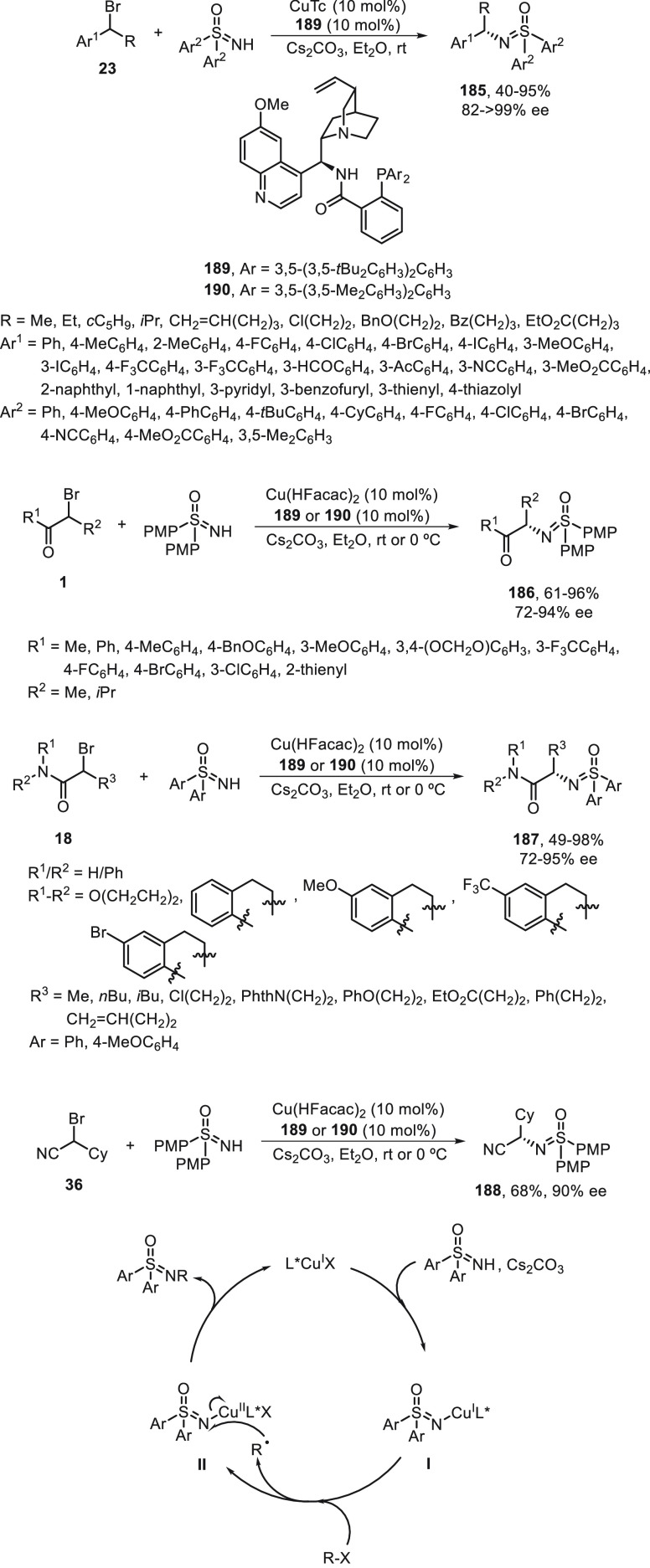
Enantioconvergent Cu-Catalyzed *N*-Alkylation of Secondary Benzylic Bromides **23**, α-Bromo Ketones **1**, α-Bromo Amides **18**, and α-Bromo Nitrile **36** with Sulfoximines

Liu and co-workers^108^ employed sulfoximines as ammonia surrogates to access α-chiral primary amines. This enantioconvergent Cu-catalyzed radical C–N coupling took place in the absence of light with secondary alkyl halides, such as benzylic bromides **23**, α-bromo ketones **1**, α-bromo amides **18**, and α-bromo nitrile **36**, to provide the corresponding *N*-alkyl sulfoximines **185**–**188** (Scheme 39). This procedure was carried out under mild thermal conditions using a Cu(I) salt and the bulky N,N,P-ligands **189** or **190** with Cs_2_CO_3_ as base in Et_2_O at room temperature or 0 °C. The authors proposed the formation of complex **I** able to reduce the alkyl bromide via a single-electron transfer process to generate the alkyl radical R^•^ by an outer-sphere radical substitution to give the product generating the Cu(I) catalyst. Products **185**–**188** (more than 60 examples) were isolated up to 99% yield and up to >99% ee and were transformed into enantioenriched primary amines by reduction with Mg or with sodium naphthalenide followed by acidic hydrolysis without remarkable losses of enantiopurity. This methodology was applied to the synthesis of commercial drugs, including cinacalcet, dapuxetine, and rivastigmine.

**Scheme 40:**
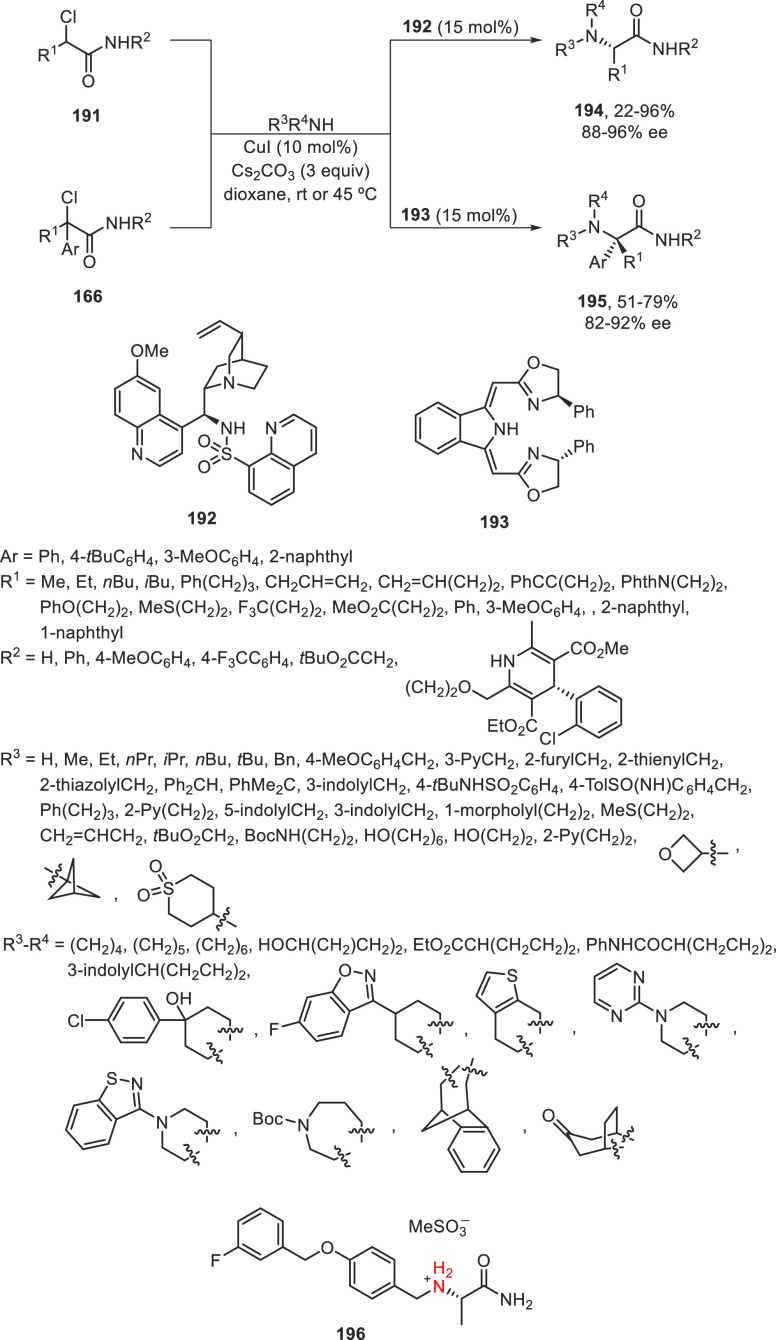
Enantioconvergent Cu-Catalyzed *N*-Alkylation of α-Chloro Amides **191** and **166** with Aliphatic Amines and Ammonia

Very recently, Liu and co-workers^109^ were able to develop the enantioconvergent Cu-catalyzed *N*-alkylation of aliphatic amines using chiral tridentate anionic ligands **192** and **193**. α-Chloro amides **191** and **166** reacted with a wide variety of primary and secondary aliphatic amines, even ammonia (more than 125 examples), to afford α-amino amides **194** and **195**, respectively, with 22–99% yields and 88–97% ee (Scheme 40). This procedure was applied to the synthesis of 12 drugs or bioactive molecules, including the anti-Parkinson drug XADAGO **196** with 62% yield and 94% ee. On the basis of experimental studies, the authors proposed the formation of a cuprate intermediate **I**, which undergoes intramolecular oxidative addition to give **II** and **III** in equilibrium. Subsequent outer-sphere amine attack to **III** delivers **IV**, which upon ligand exchange with the α-chloro amide forms the *N*-alkylated product.

Enantioconvergent cross-coupling amination and amidation of alkyl electrophiles has been mainly performed under photoinduced copper-catalyzed conditions and using phosphines as chiral ligands. In the absence of light, sulfoximines and aliphatic amines are able to perform the *N*-alkylation of activated alkyl bromides under Cu catalysis.

#### 2.2.2 Oxygen Nucleophiles

**Scheme 41:**
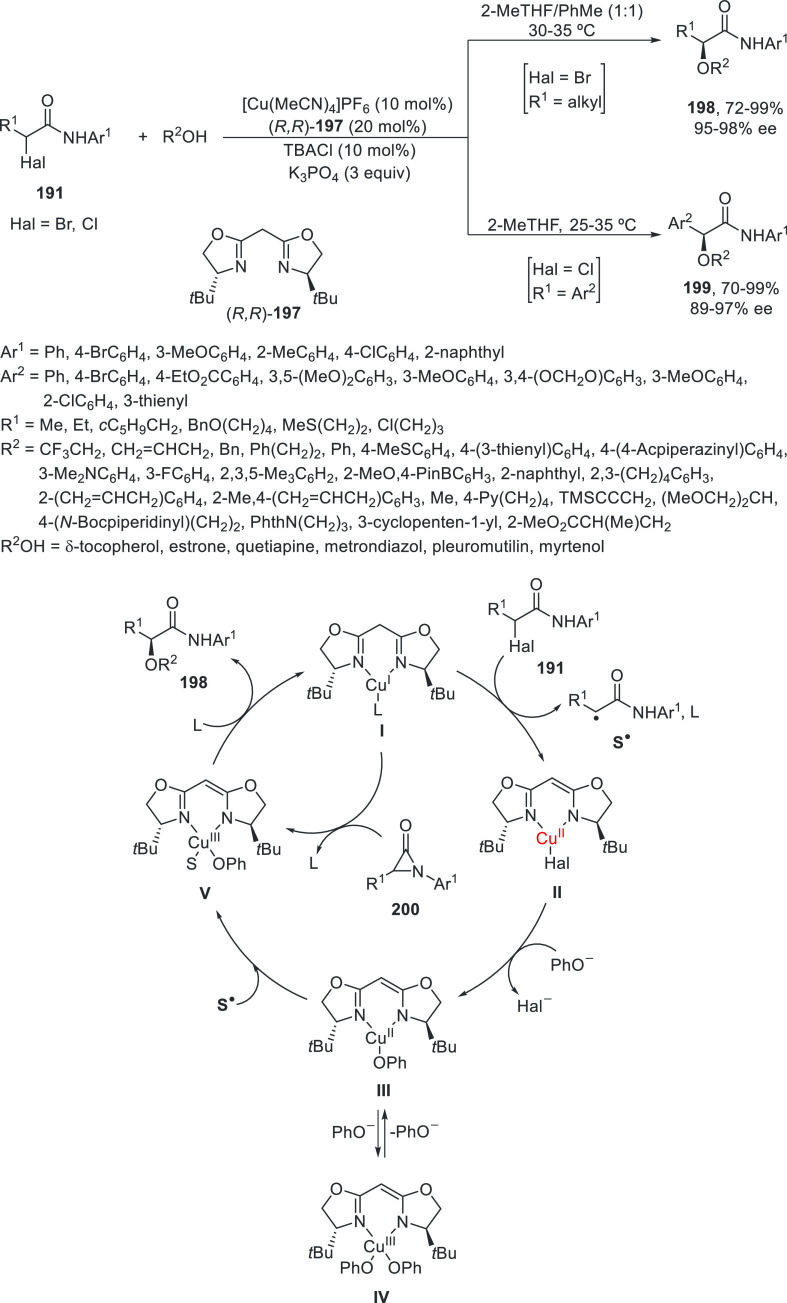
Enantioconvergent Cu-Catalyzed Cross-Coupling of α-Halo Amides **191** with Oxygen Nucleophiles

Cross-coupling reactions between α-bromo amides **191** and alcohols under Cu catalysis were reported by Kürti and co-workers in 2018.^110^ However, just recently, Chen and Fu^111^ achieved the enantioconvergent process using Cu(II) and a chiral bis(oxazoline) **197** (Scheme 41). The cross-coupling of α-bromo and α-chloro amides **191** took place with aliphatic alcohols and phenols to efficiently give α-alkoxy amides **198** and **199** in very good yields and with high enantioselectivity. These reaction conditions were also applied to the enantioconvergent alkylation of nitrogen nucleophiles, such as aliphatic primary amines and anilines. Experimental studies support that the process proceeds through a free radical pathway. Thus, Cu(I) complex **I** reacts with the α-halo amide to give the Cu(II) complex **II** and the alkyl radical **S**^•^. This complex **II** undergoes ligand substitution with the oxygen nucleophile to provide complexes **III** and **IV**. Then, the alkyl radical **S**^•^ reacts at copper to form organocopper(III) complex **V** via an out-of-cage pathway. This complex **V** can also be formed by oxidative addition of the aziridinone **200** to complex **I** along with complexation of phenol. Reductive elimination determines the stereochemistry and affords the product and complex **I**.

#### 2.2.3 Phosphorus Nucleophiles

**Scheme 42:**
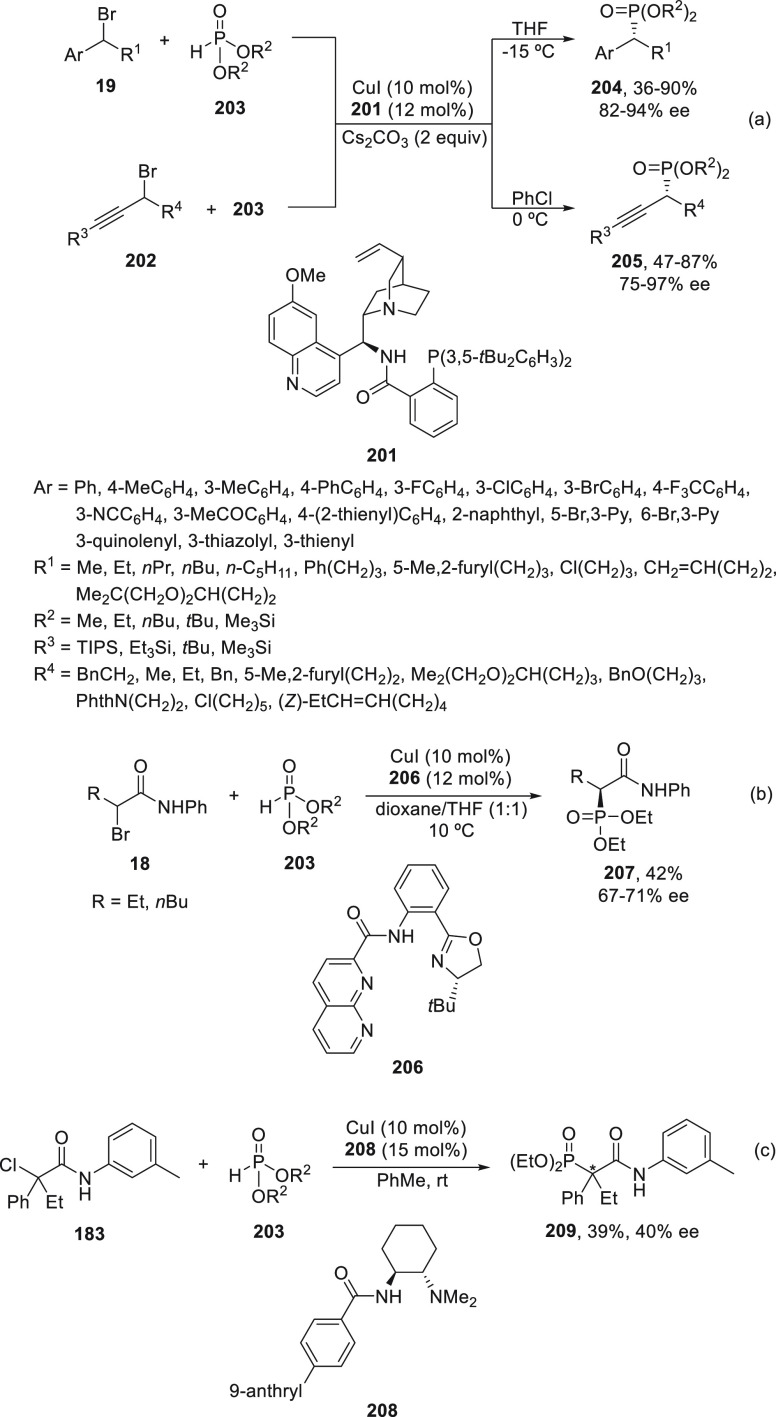
Enantioconvergent Cu-Catalyzed Michaelis–Becker Reaction of Alkyl Halides with H-Phosphonates

The Michaelis–Becker (M-B) reaction of H-phosphonates with alkyl halides is a direct method for the synthesis of C-phosphonates.^112^ Only recently, Liu and co-workers^113^ reported the enantioconvergent copper-catalyzed M-B-type C(sp^3^)–P cross-coupling reaction. By using the multidentate chiral anionic ligand **201**, benzylic bromides **23** and propargylic bromides **202** reacted with H-phosphonates **203** to furnish products **204** and **205**, respectively, with remarkable chemo- and enantioselectivity (Scheme 42a). In the case of α-halo carboxamides **18** and **183**, ligands **206** and **208** were used, respectively, to provide products **207** and **209** with moderate results (Schemes 42b,c). Concerning the reaction mechanism, a radical trap experiment with TEMPO supports a stereoselective radical pathway over a stereospecific S_N_2-type process.

#### 2.2.4 Other Carbon Nucleophiles

**Scheme 43:**
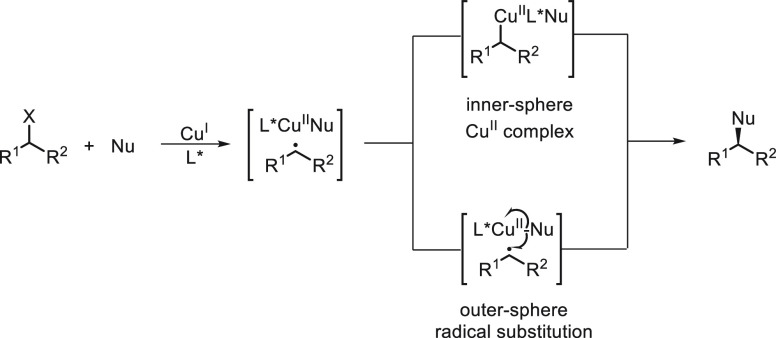
Enantioconvergent Strategy for Cu-Catalyzed Radical C(sp^3^)–C Cross-Coupling Reactions

Enantioconvergent cross-coupling of racemic alkyl electrophiles with carbon nucleophiles, such as cyanides, acetylides, and nitronates, by generation of achiral radicals via an inner-sphere single-electron transfer (SET) process with a chiral transition-metal catalyst will be considered. In addition, under photoredox catalysis an outer-sphere SET strategy produces alkyl radicals that by enantioselective radical coupling forms C(sp^3^)–C bonds. In Scheme 43, the Cu-catalyzed enantioconvergent radical C(sp^3^)–C cross-coupling reactions of alkyl electrophiles with nucleophiles are depicted.^114^

**Scheme 44:**
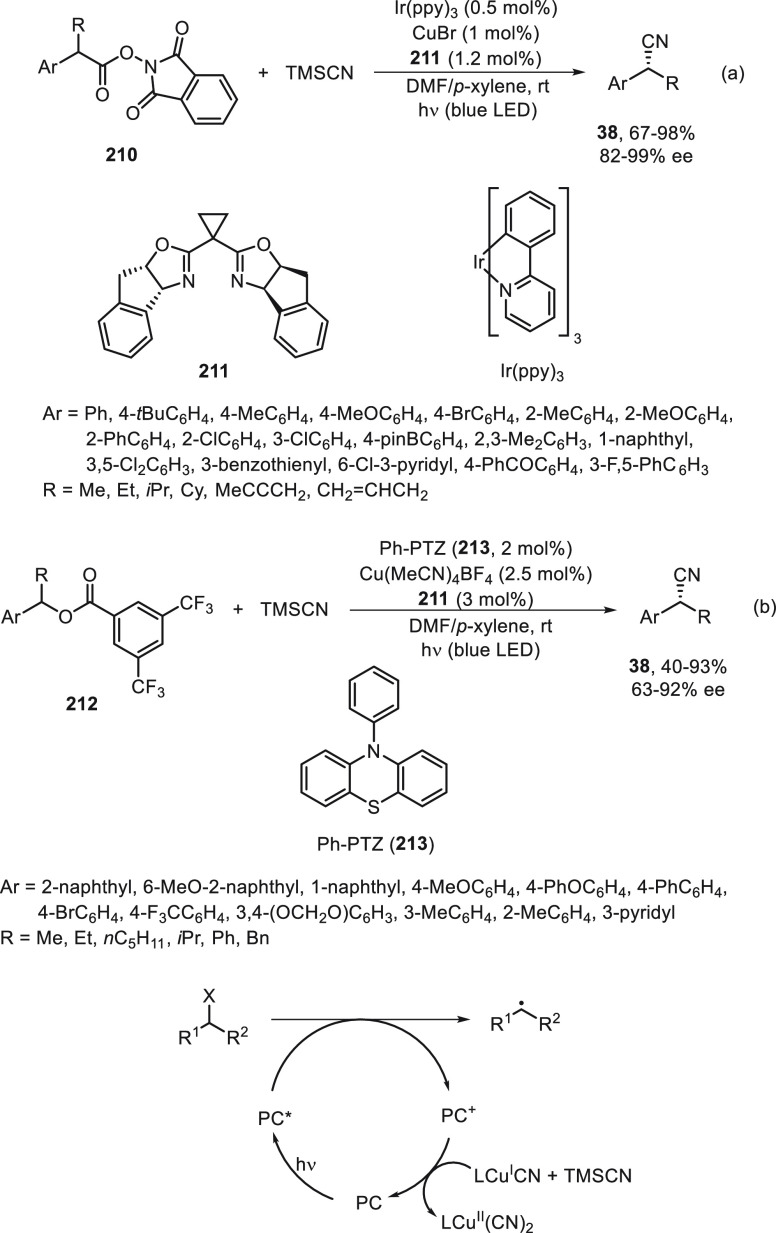
Enantioconvergent Photocatalyzed Cu-Catalyzed Cyanation of Esters **210** and **212** with TMSCN

Concerning cyanation reactions, for the synthesis of enantioenriched benzylic nitriles, two types of enantioconvergent photocatalyzed cross-couplings have been reported, either starting from carboxylic acid derivatives **210**(115) or benzylic alcohol esters **212**.^116^ Liu and co-workers^115^ performed decarboxylation of *N*-hydroxyphthalimide (NHP) esters **210** in the presence of trimethylsilyl cyanide (TMSCN) to give the corresponding benzylic nitriles **38** in up to 98% yield and 99% ee (Scheme 44a). This process was carried out using Ir(ppy)_3_ as photocatalyst under blue LED irradiation and CuBr/bis(oxazoline) **211** as chiral metal catalyst. Conversely, Xiao and co-workers^116^ started from 3,5-bis(trifluoromethyl)benzoyl esters **212**, which by reaction with TMSCN afforded the corresponding nitriles **38** in up to 93% yield and 92% ee (Scheme 44b). In this case, an organic photocatalyst Ph-PTZ (**213**) and Cu(MeCN)_4_BF_4_/bis(oxazoline) **211** as chiral metal catalyst were used. In a simplified catalytic cycle to explain the initiation of these reactions, starting compounds **210** and **212** gave benzylic radicals by photocatalytic decarboxylation and deoxygenation, respectively, by transfer of one electron of the excited photocatalyst (PC*). This PC* can oxidize L*Cu^I^CN to form L*Cu^II^CN, which reacts with TMSCN to form L*Cu^II^(CN)_2_. Subsequent combination of the benzylic radical with the active species L*Cu^II^(CN)_2_ by an outer-sphere radical substitution would provide the coupling product **38**.

**Scheme 45:**
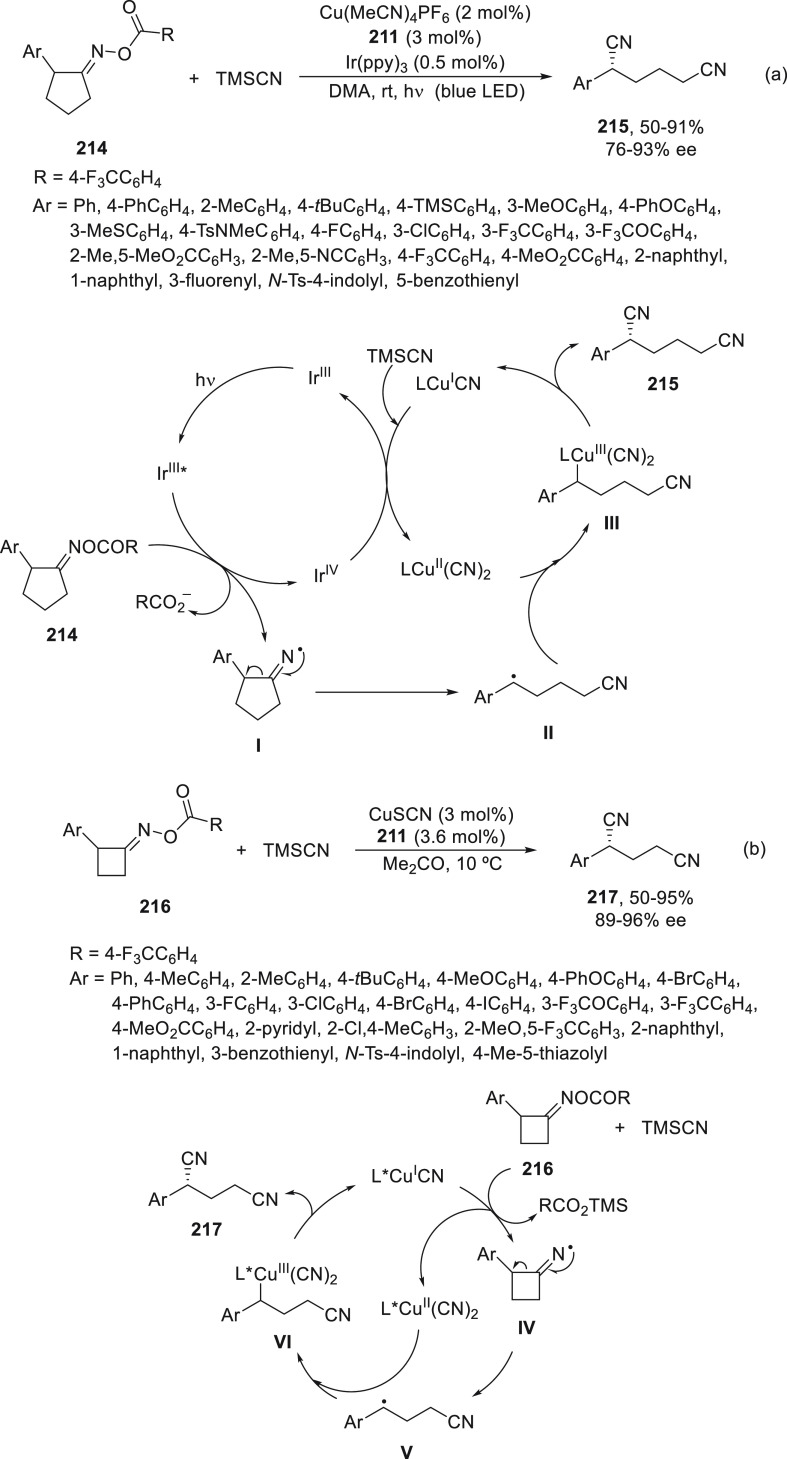
Enantioconvergent Cu-Catalyzed Cyanation of Cyclopentanone and Cyclobutanone Oxime Esters **214** and **216** with TMSCN to Give Dinitriles **215** and **217**

Wang and co-workers^117^ employed the dual photoredox/copper catalysis for the enantioconvergent ring-opening cyanation of cyclopentanone oxime esters **214** with TMSCN to access enantioenriched 1,6-dinitriles **215** in high yields and enantioselectivities (Scheme 45a). This process is based on the iminyl radical-mediated ring-opening of cyclic oxime derivatives by cleavage of the C–C single bond via β-scission reported by Zard and co-workers.^118^ Alternatively, cyclobutanone oxime esters **216** were transformed into enantioenriched 1,5-dinitriles **217** only under copper catalysis, presumably because of the higher strain release of four-membered ring than cyclopentanones (Scheme 45b).^119^ In both cases, bis(oxazoline) **211** was used as chiral ligand. In the proposed mechanism based on experimental studies, in the catalytic cycle for cyclopentanone oxime esters **214**, a SET process between the oxime and the excited state of photocatalyst Ir(III)* provided iminyl radical **I** and the Ir(IV) species, which oxidized L*Cu^I^CN to L*Cu^II^(CN)_2_ by reaction with TMSCN. Intermediate **I** generates by C–C bond cleavage the benzylic radical **II**, which is trapped by L*Cu^II^(CN)_2_ to deliver intermediate **III**. Final reductive elimination of **III** gives the desired product **215** and regenerates the catalyst. For the cyclobutanone oxime esters **216**, an initial SET process with L*Cu^I^CN in the presence of TMSCN affords L*Cu^II^(CN)_2_ and iminyl radical **IV**, which evolves to radical **V** by C–C bond cleavage. Radical **V** reacts with L*Cu^II^(CN)_2_ to give species **VI** followed by reductive elimination to provide dinitrile **217** and the catalyst L*Cu^I^CN.

Xiao, Chen, and co-workers^120^ independently reported the cyanation of cyclopentanone oxime esters **214** under dual photoredox and Cu catalysis using the reaction conditions depicted in Scheme 44b. In this case, they used a 2 × 3 W purple LED and DMA as solvent at 30 °C to provide dinitriles **215** with 75–99% yield and 81–94% ee.

**Scheme 46:**
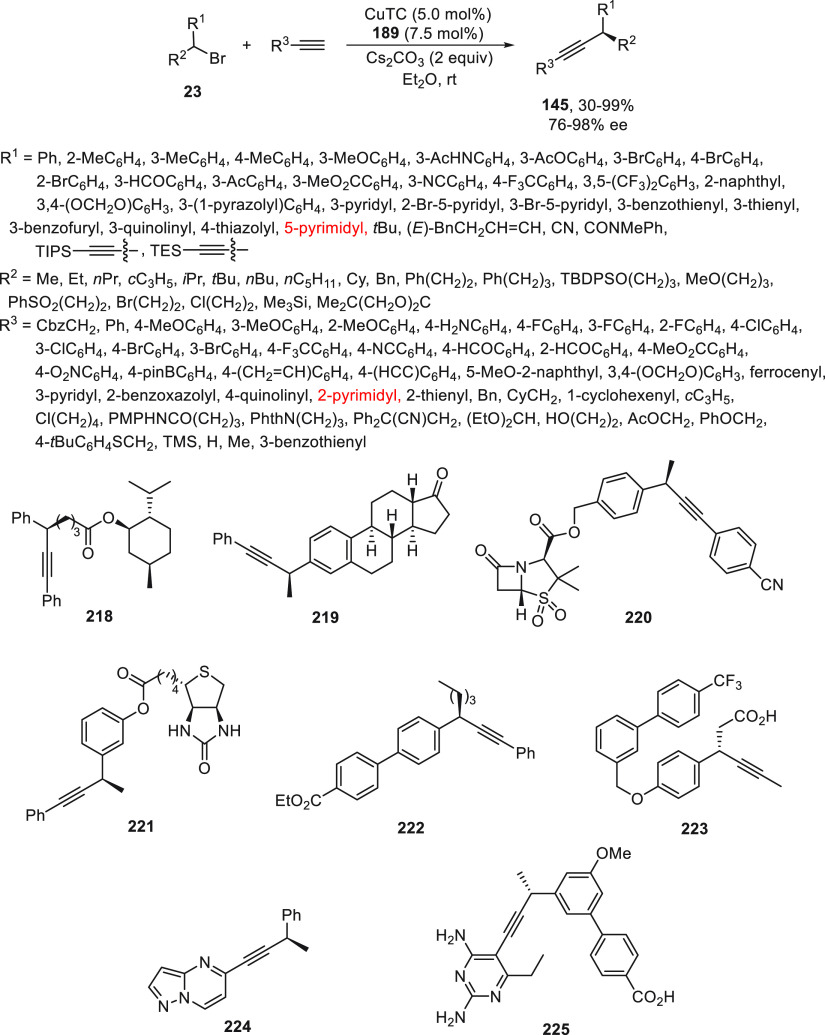
Enantioconvergent Cu-Catalyzed Reaction of Secondary Benzylic Bromides **23** with Acetylenes

Enantioconvergent C(sp^3^)–C(sp) cross couplings of secondary alkyl halides with alkynes have been developed by Liu and co-workers^121^ under CuTC (TC = thiophene-2-carboxylate) catalysis. This radical process needed the strong donating multidentate ligand developed by Dixon et al.^122^ (**189**) to enhance the reducing capability of the Cu catalyst, as well as to suppress the Glaser homocoupling.^114,121^ Benzylic bromides **23** reacted with terminal aromatic and aliphatic acetylenes, including acetylene, itself, using Cs_2_CO_3_ as base in ethyl ether at room temperature to provide products **145** (>120 examples) with good yields and enantioselectivities (Scheme 46). Synthetic applications of these transformations employed the core of several bioactive molecules, such as l-menthol, estrone, sulbactam, biotin, and a mesogenic compound **218**–**222**. In addition, they prepared chiral alkyne drug leads, such as **223** (AMG 837), a G-protein coupled receptor GPR40 agonist, and **224**, a patented mGluR modulator. They also prepared a dihydrolate reductase (DHFR) inhibitor UCP1172 **225** for drug-resistant bacteria treatment and other bioactive molecules. The reaction possibly proceeds by formation of the alkynylcopper(I) complex **I**, which undergoes a SET process with the racemic alkyl bromide to afford the radical species, and the Cu^II^ intermediate **II**. Subsequent coupling of both species delivers the coupling product and releases the catalyst.

**Figure 2:**
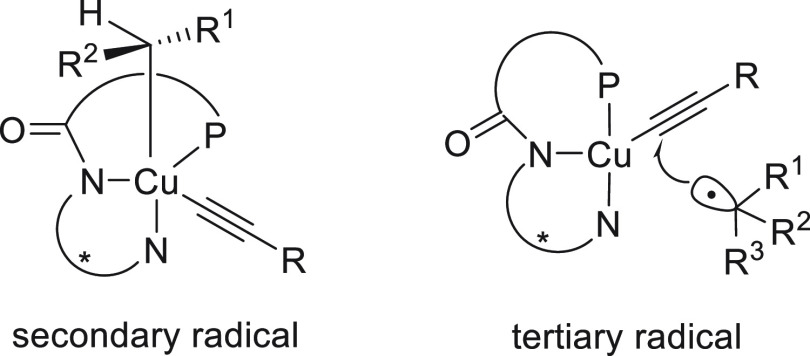
TSs for the Cu-catalyzed cross-coupling of secondary and tertiary radicals with acetylenes.

For the Cu-catalyzed enantioconvergent C(sp^3^)–C(sp) cross-coupling of tertiary electrophiles with alkynes, Liu and co-workers^123^ have developed tailor-made N,N,N-ligands on the basis of mechanistic studies. DFT calculations revealed that the coupling of the tertiary alkyl radical and the alkynyl group proceeded via an outer-sphere radical substitution-type C–C bond-formation pathway (Figure 2). However, the secondary alkyl radical is involved in the reductive elimination from an inner-sphere Cu(III) intermediate formed upon radical trapping^121^ (Figure 2). The enantiodetermining transition states in the outer-sphere C–C bond-formation mechanism are less organized, and therefore, an appropriate ligand should favor the accommodation of the sterically bulky tertiary radical.

**Scheme 47:**
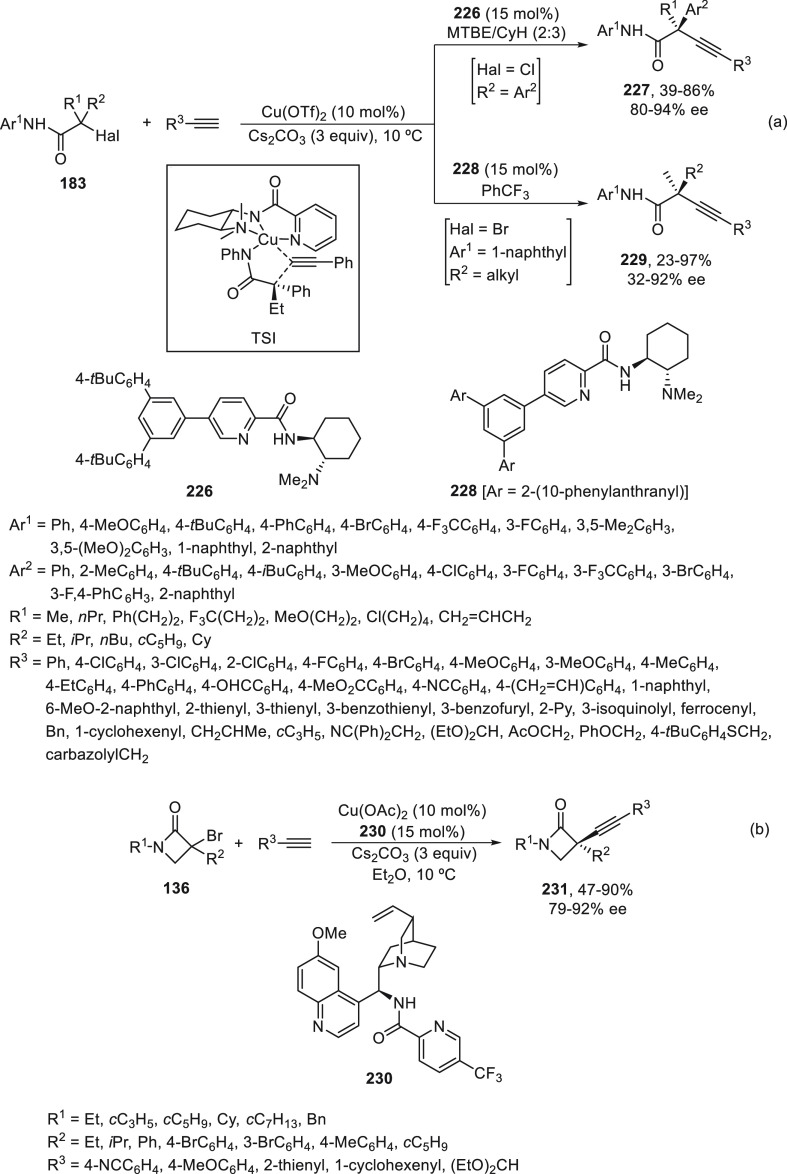
Enantioconvergent Cu-Catalyzed Sonogashira–Hagihara Reaction of Tertiary α-Halo Amides **183** and **136** with Acetylenes

In the case of the cross-coupling of α-chloro amides **183** bearing an aryl group at the α-position, ligand **226** was the most efficient and afforded products **227** in 39–86% yields with 80–94% ee (Scheme 47a).^123^ α-Bromo amides **183** with two alkyl groups at the α-position were cross-coupled with terminal acetylenes in the presence of ligand **228** to provide products **229** in 23–67% yields with 32–92% ee (Scheme 47a). Cross-coupling of α-bromo-β-lactams **136** with terminal acetylenes was achieved using the chiral ligand **230** to furnish products **231** in good yields (47–90%) with 79–92% ee (Scheme 47b). DFT calculations explained the efficient enantiodiscrimination on the basis of the enantiodetermining outer-sphere radical group transfer pathway (see, TS).

**Scheme 48:**
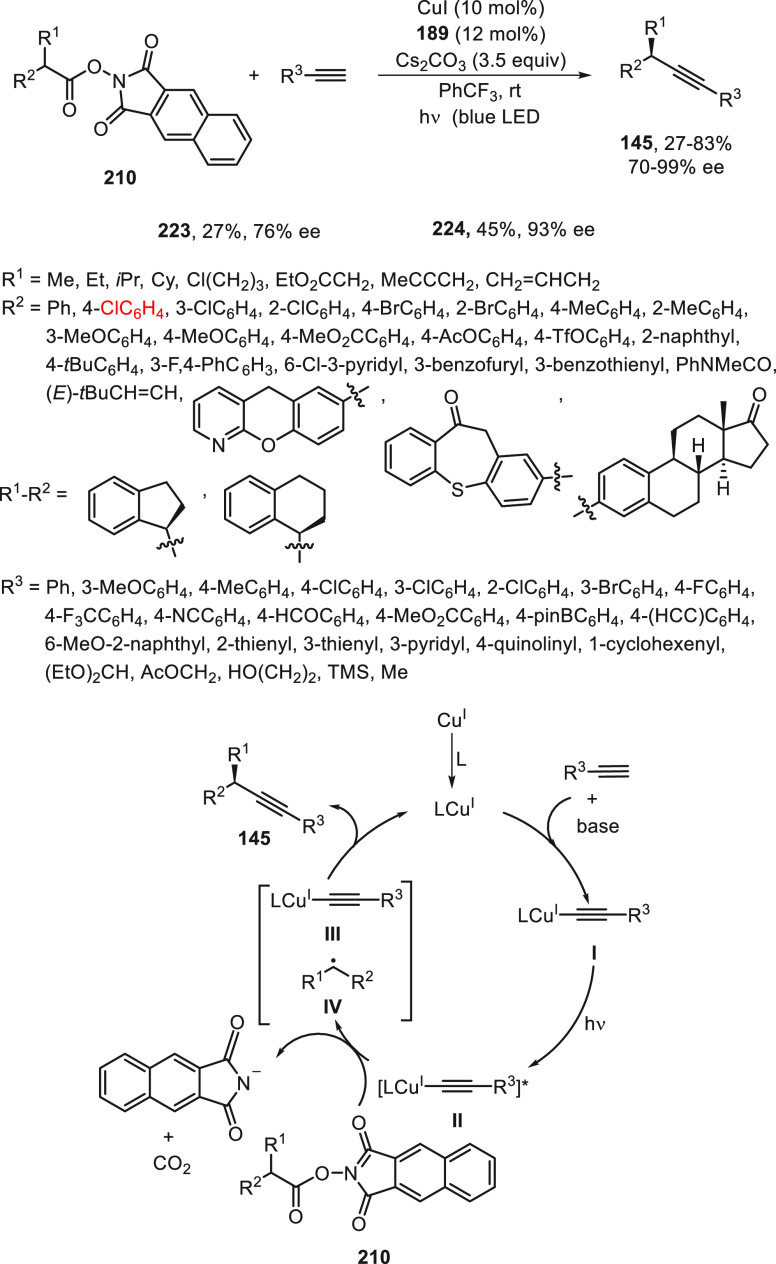
Enantioconvergent Photocatalytic and Copper-Catalyzed Decarboxylative Alkynylation of Esters **210**

The same group^124^ performed the enantioconvergent radical decarboxylative C(sp^3^)–C(sp) cross-coupling by reaction of NHP-esters **210** with terminal alkynes. This photoinduced copper-catalyzed alkynylation of esters **210** as radical precursors used the anionic chiral multidentate N,N,P-ligand **189** for the enantiocontrol over prochiral radical intermediates to avoid their homodimerization. Because of the use of stable and easily available NHP-type esters **210**, a broader substrate scope compared with their alkyl halide counterparts **23** was observed, which gave products **145** in moderate to good yields and excellent enantioselectivities (Scheme 48). In addition, a tandem one-pot procedure was developed starting from carboxylic acids, which were esterified and then, without purification, submitted to the standard asymmetric radical decarboxylative alkynylation. On the basis of experimental studies, a possible catalytic cycle was proposed. Thus, intermediate **I** was excited to give complex **II**, which transfers one electron to NHP-type ester **210** to deliver the Cu^II^-complex **III**. The formed anionic radical of ester **210** undergoes a radical decarboxylation to generate the radical intermediate **IV**. Finally, C(sp^3^)–C(sp) bond formation with **III** provides the final product **145** and regenerates the catalyst L*Cu^I^ complex.

**Scheme 49:**
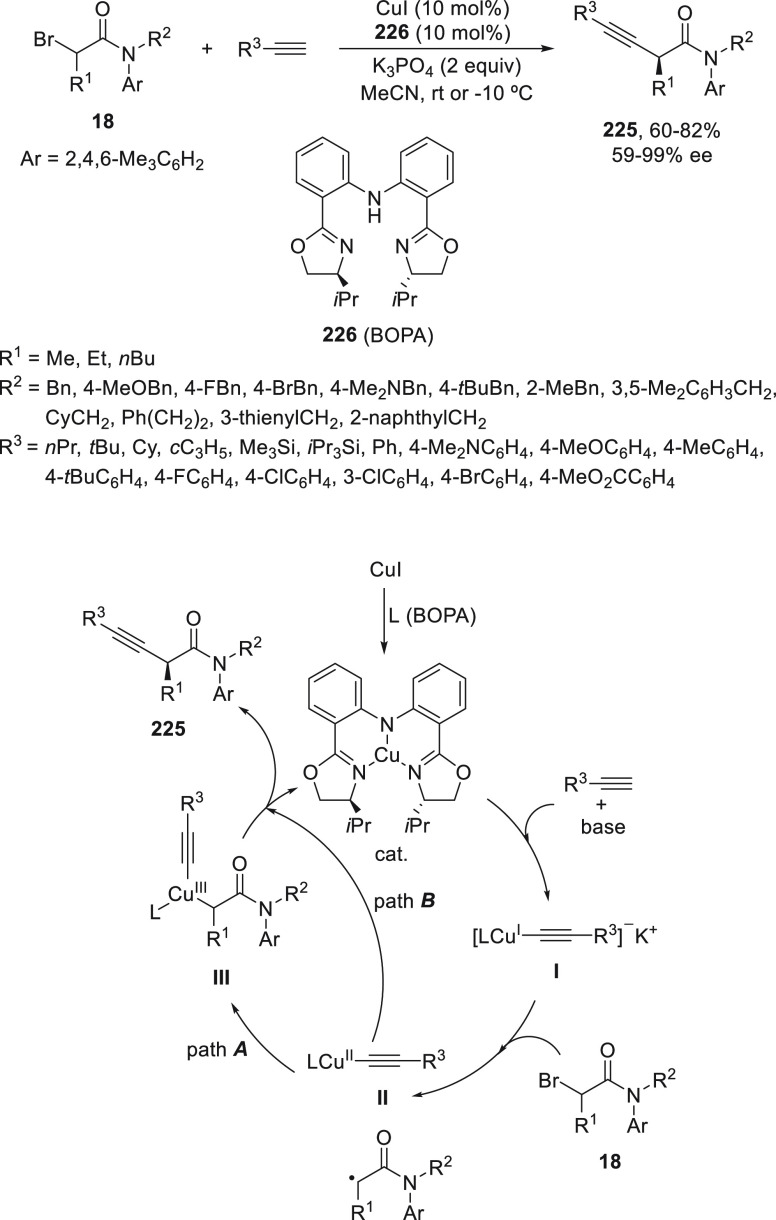
Enantioconvergent Cu-Catalyzed Sonogashira–Hagihara Reaction of α-Bromo Amides **18** with Acetylenes

Zhang and co-workers^125^ reported an enantioconvergent Cu-catalyzed alkynylation reaction of α-bromo amides **18** with terminal alkynes to provide β,γ-alkynyl amides **225** (Scheme 49). They used as anionic chiral ligand^114^ a bis(oxazoline) diphenylanaline **226** (BOPA) containing a central anionic nitrogen σ-donor and two lone pair donors from the oxazoline units reported by Nakada et al.,^126^ whereas simple chiral bis(oxazoline) ligands are not effective in this cross-coupling reaction. Racemic α-bromo amides bearing a 2,4,6-trimethyphenyl group at the nitrogen are critical for good stereocontrol to give products **225** with good yields and high enantioselectivities. The authors proposed two possible pathways involving either an inner-sphere Cu^III^ complex **III**, which undergoes reductive elimination to generate the product (path ***A***), or the radical undergoes direct out-of-cage bond formation with the Cu^II^ species **II** to furnish the product (path ***B***).

**Scheme 50:**
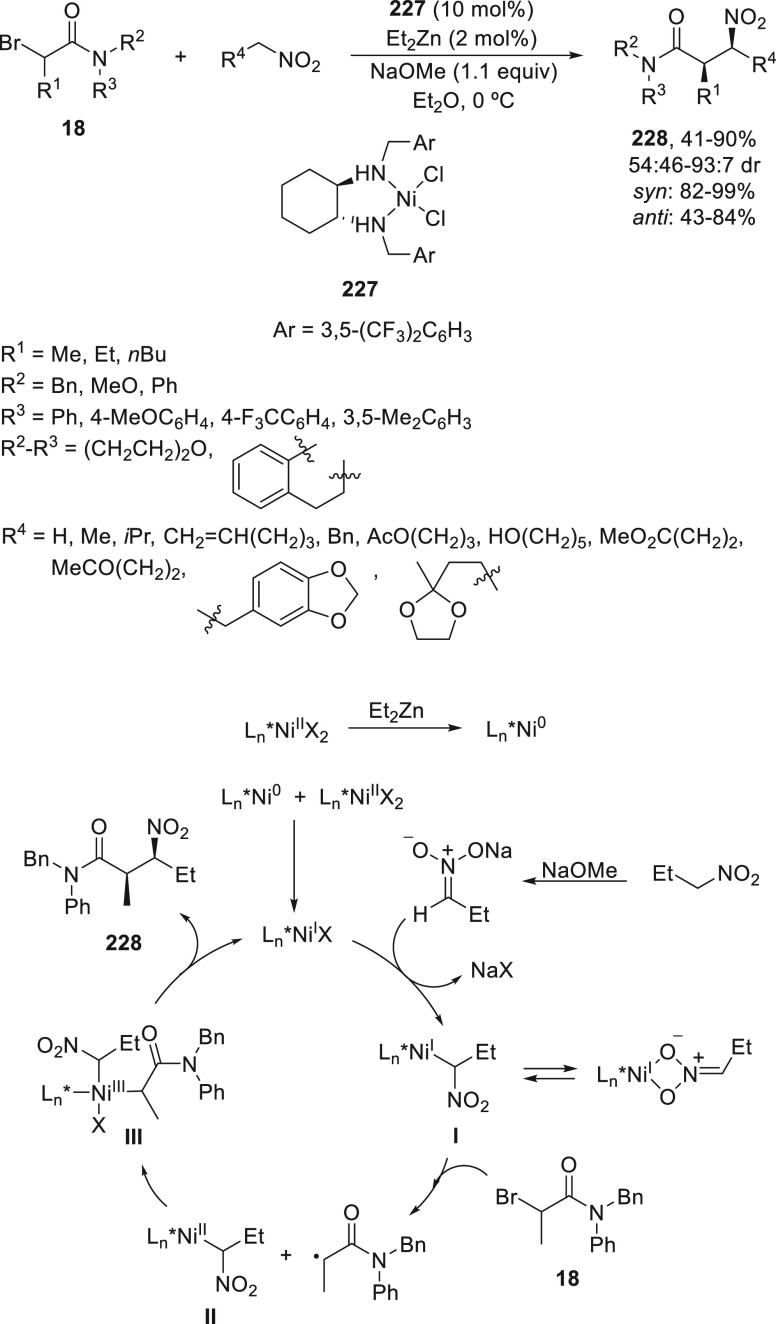
Enantioconvergent Ni-Catalyzed Reaction of α-Bromo Amides **18** with Nitroalkanes

Enantioconvergent alkylation of nitroalkanes with racemic α-bromo amides **18** has been carried out under asymmetric Ni catalysis by Watson and co-workers.^127^ They employed the Ni-precatalyst **227** (10 mol %), Et_2_Zn (2 mol %) as *in situ* reductant, and NaOMe (1.1 equiv) as base in ethyl ether at 0 °C to provide β-nitro amides **228** mainly as *syn* diastereomers (Scheme 50). In the proposed mechanism, initial reduction of Ni^II^ to Ni^0^ followed by comproportionation with the excess of Ni^II^ complex **227** results in a Ni^I^ catalyst. Simultaneous deprotonation of the nitroalkane by NaOMe gives a nitronate anion, which undergoes anion exchange with the Ni^I^ complex to result in intermediate **I**. This Ni nitronate reacts with the α-bromo amide via a stepwise oxidative addition to form the Ni^II^ intermediate **II** and subsequently with the radical to form the Ni^III^ species **III**. Reductive elimination of **III** provides the product and regenerates the catalyst.

For the enantioconvergent decarboxylative cyanation of esters, a cooperative photoredox and copper/bis(oxazoline) catalysis produces enantioenriched benzylic nitriles. Iminyl-radical-triggered C–C bond cleavage of cyclohexanone oxime esters under Cu/bis(oxazoline) catalysis gave chiral 1,6- and 1,5-dinitriles. In the case of alkynylation reactions of benzylic bromides or NHP esters under Cu catalysis, the presence of a chiral multidentate N,N,P-ligand was crucial. Alkynylation reaction under Cu catalysis of α-bromo amides also needs a multidentate anionic N,N,N-ligand. Alkylation of nitroalkanes with α-bromo amides has been performed with NiCl_2_ and Et_2_Zn as reductant using a chiral 1,2-diamine ligand. In all these processes, radical intermediates are involved.

#### 2.2.5 Boron Reagents

**Scheme 51:**
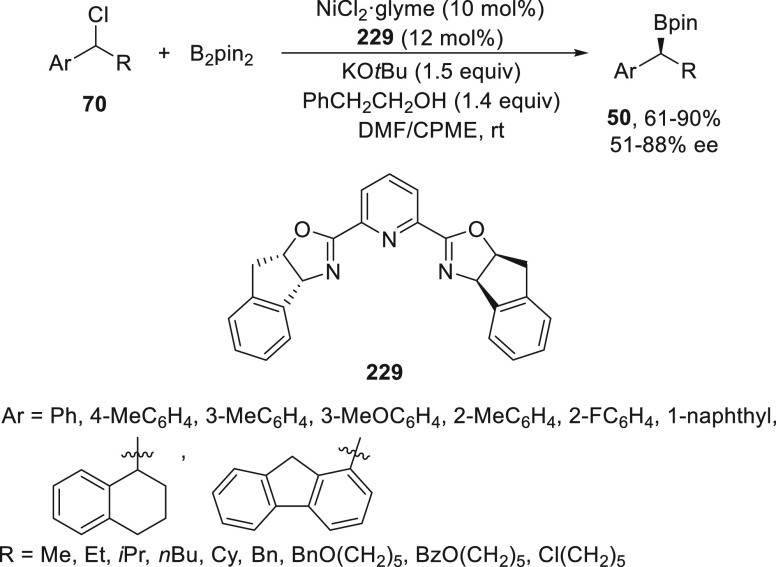
Enantioconvergent Ni-Catalyzed Miyaura Borylation of Benzylic Chlorides **70**

Concerning borylation of alkyl halides under metal catalysis, Miyaura borylation provided C–B bond formation to give alkylboranes. Dunik and Fu^128^ reported the Ni-catalyzed cross-coupling reaction of primary, secondary, and tertiary alkyl halides with diboron reagents. The resulting boronic esters can be aminated, hydroxymethylated, and arylated. Enantioenriched alkylboron compounds were converted with high retention of the configuration.^129^ Enantioconvergent Ni-catalyzed borylation of racemic secondary benzylic chlorides **70** was described by Fu and co-workers.^130^ The corresponding benzylic boronic esters **50** were obtained with good yields and enantioselectivities using NiCl_2_/bis(oxazoline) **229** as catalyst and B_2_pin_2_ as borylating reagent (Scheme 51). The authors demonstrated that enantioenriched benzylic chlorides do not undergo racemization under these reaction conditions. In the proposed mechanism, a radical pathway^128^ analogous to that described for the Kumada and Negishi reactions (see Section 2.1) was suggested.

**Scheme 52:**
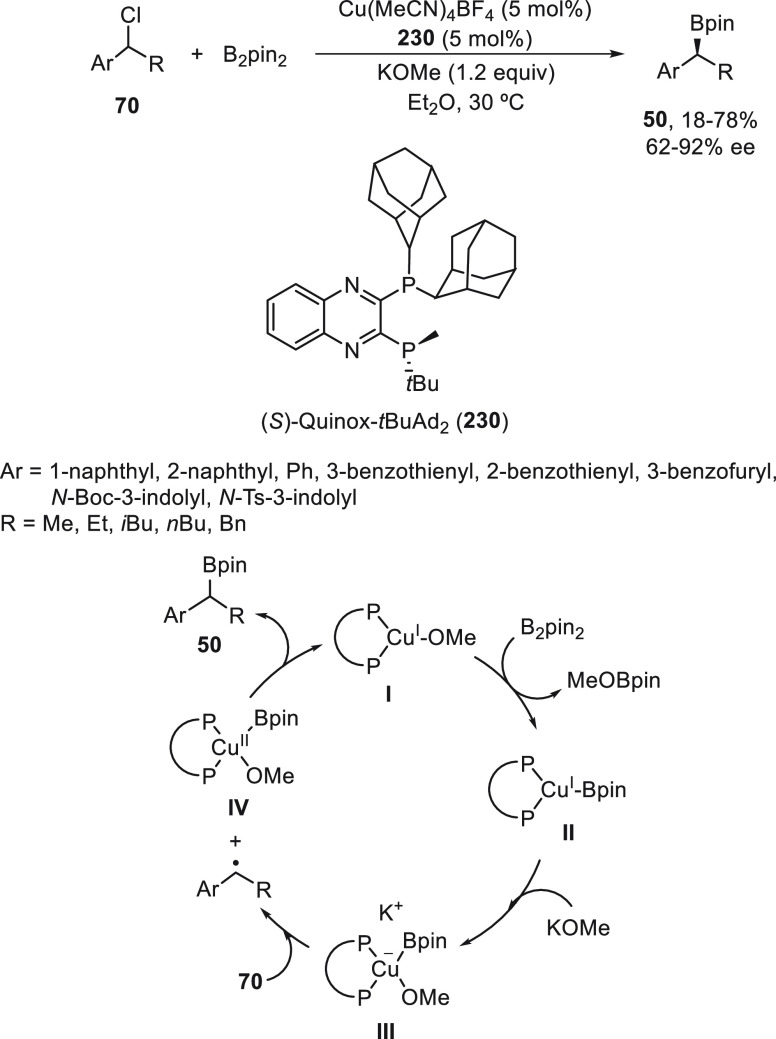
Enantioconvergent Cu-Catalyzed Miyaura Borylation of Benzylic Chlorides **70**

The copper(I)-catalyzed enantioconvergent borylation of racemic benzylic chlorides **70** with B_2_pin_2_ has been reported by Ito and co-workers.^131^ Boronic esters **50** resulted in moderate to good yields and enantioselectivities using Cu(MeCN)_4_BF_4_/bisphosphine (*S*)-quinox-*t*BuAd_2_**230** as chiral catalyst (Scheme 52). Mechanistic studies on copper(I)-catalyzed borylation reactions have led to a plausible catalytic cycle that involves a radical intermediate.^131,132^ Catalyst **I** reacts with B_2_pin_2_ to give the borylcopper(I) species **II**, which reacts with KOMe to provide cuprate **III**. SET from **III** to benzylic chloride generates the benzylic radical and the Cu(II) species **IV**. Subsequent enantioselective borylation of the radical by intermediate **IV** furnishes the product **50** and regenerates the catalyst **I**.

### 2.3 Racemic Alkylmetals with Electrophiles

**Scheme 53:**
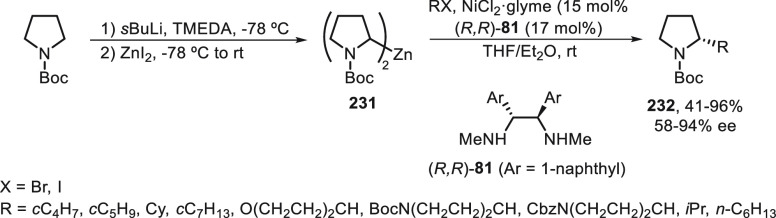
Enantioconvergent Ni-Catalyzed Negishi Reactions of Racemic α-Zincated *N*-Boc-pyrrolidine 231 with Alkyl Halides

Enantioconvergent cross-coupling reactions for C(sp^3^)–C bond formation can be also performed through an umpoled strategy using racemic secondary alkyl metals. The first reverse polarity process was described by Kumada and co-workers^133^ using a racemic benzylic Grignard reagent (PhCHMeMgCl) and vinyl bromide as reaction partners and Ni complexes of chiral (aminoalkylferrocenyl)phosphines to generate enantioenriched 3-methylallylbenzene. Fu and co-workers^134^ performed the enantioconvergent Negishi reaction of α-zincated *N*-Boc-pyrrolidine **231** with alkyl halides under NiCl_2_/diamine **81** catalysis (Scheme 53). The resulting enantioenriched α-alkyl-*N*-Boc-pyrrolidines **232** were obtained with good yields when alkyl iodides were employed as electrophiles (50–96%), with lower yields with alkyl bromides (41–80%), and with good enantioselectivities, in general.

**Scheme 54:**
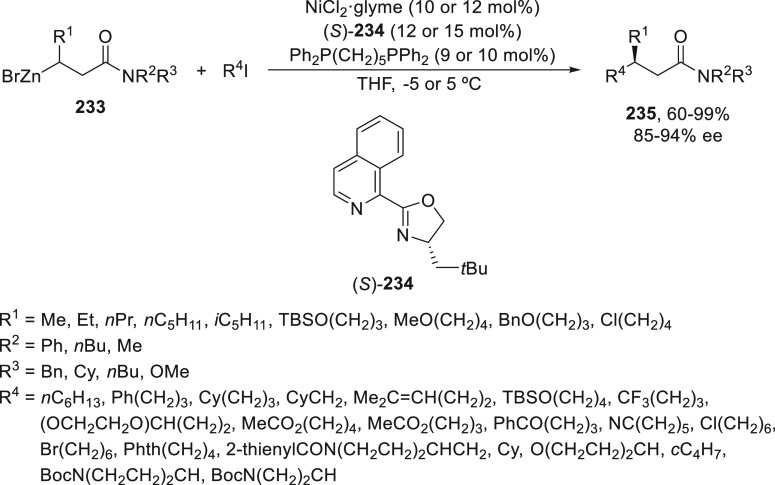
Enantioconvergent Ni-Catalyzed Negishi Reaction of Racemic β-Zincated Amides **233** with Alkyl Iodides

The same group recently reported^135^ this type of cross-coupling reaction using β-zincated amides **233** and a broad range of alkyl iodides (Scheme 54). The chiral catalyst NiCl_2_/isoquinoline-oxazoline **234** gave products **235** with good yield and ee. The reaction with primary alkyl groups was performed with 10 mol % of NiCl_2_ and 12 mol % of ligand at −5 °C, whereas secondary alkyl iodides needed a higher loading, 12 mol % NiCl_2_, and 15 mol % ligand at 5 °C.

**Scheme 55:**
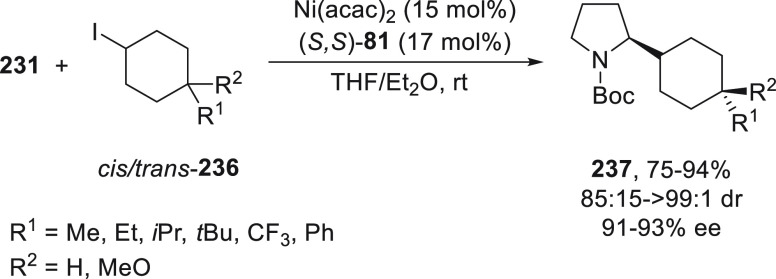
Doubly Enantioconvergent Ni-Catalyzed Negishi Reaction of α-Zincated *N*-Boc-pyrrolidine **231** with Racemic Alkyl Electrophiles **236**

Doubly enantioconvergent cross-coupling of racemic alkyl nucleophiles and electrophiles was also described by Fu and co-workers.^136^ Vicinal stereocenters are generated with very good stereoselectivity when α-zincated *N*-Boc-pyrrolidine **231** was allowed to react with 4-substituted cyclohexyl iodides **236** under Ni catalysis (Scheme 55). This Negishi reaction proceeds with good yields to give products **237** with good ee and diastereoselectivity, and it also proceeds with 4,4-disubstituted and 3,5-disubstituted cyclic iodides. With respect to the new C–C bond, the chiral catalyst controls the stereochemistry of the stereocenter in the nucleophile, and the substrate controls the stereochemistry of the stereocenter generated in the electrophile.

**Scheme 56:**
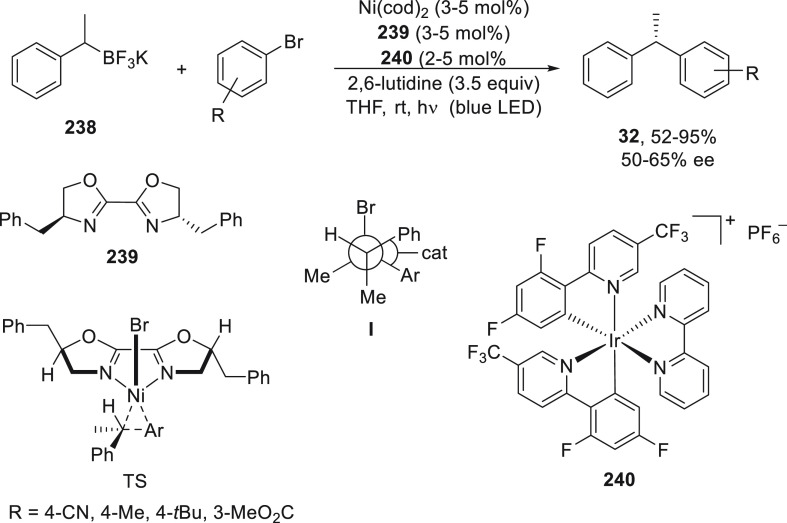
Enantioconvergent Photocatalytic and Ni-Catalyzed Suzuki Reactions of Potassium Alkyltrifluoroborate **238** with Aryl Bromides

The first enantioconvergent Suzuki reaction of racemic alkylboron reagents with aryl halides was described by Molander and co-workers.^137−139^ Because of the slow rate of transmetalation of inactivated alkylboron, a photocatalyst **240** and nickel/bis(oxazoline) **239** as dual catalysts enable the cross-coupling of potassium alkyltrifluoroborate **238** with aryl bromides to afford enantioenriched diaryl ethanes **32** in moderate enantioselectivity (Scheme 56). On the basis of DFT calculations, it was proposed to be a DKR of a Ni(III) intermediate wherein the stereodetermining step is the reductive elimination.^139^ In the lower energy diastereomeric TS, the gauche-like interactions (**I**) along the forming C–C bond are avoided by rotation of the α-methylbenzyl group.

In comparison with enantioconvergent cross-coupling reactions of racemic alkyl halides with nucleophiles, only secondary racemic alkylzinc reagents react with electrophiles in an enantioconvergent manner under Ni catalysis. However, other racemic organometals, such as organoboron reagents, equilibrate under the reaction conditions. Recently, another strategy has involved Ni-catalyzed double enantioconvergent cross-coupling of racemic secondary alkylzincs with racemic secondary alkyl electrophiles to generate two stereocenters.

### 2.4 Reductive Cross-Couplings

An alternative strategy for enantioconvergent C–C bond formation between one electrophile and one organometallic partner is the cross-electrophile coupling reaction.^140,141^ This reductive cross-coupling (RCC) reaction has been carried out mainly under Ni catalysis between C(sp^3^) and C(sp^2^) electrophiles in the presence of a terminal reductant and alternatively under photoredox catalysis. Enantioconvergent related processes, such as decarboxylative cross-coupling reactions and acyl cross-coupling reactions, will be also considered.

**Figure 3:**
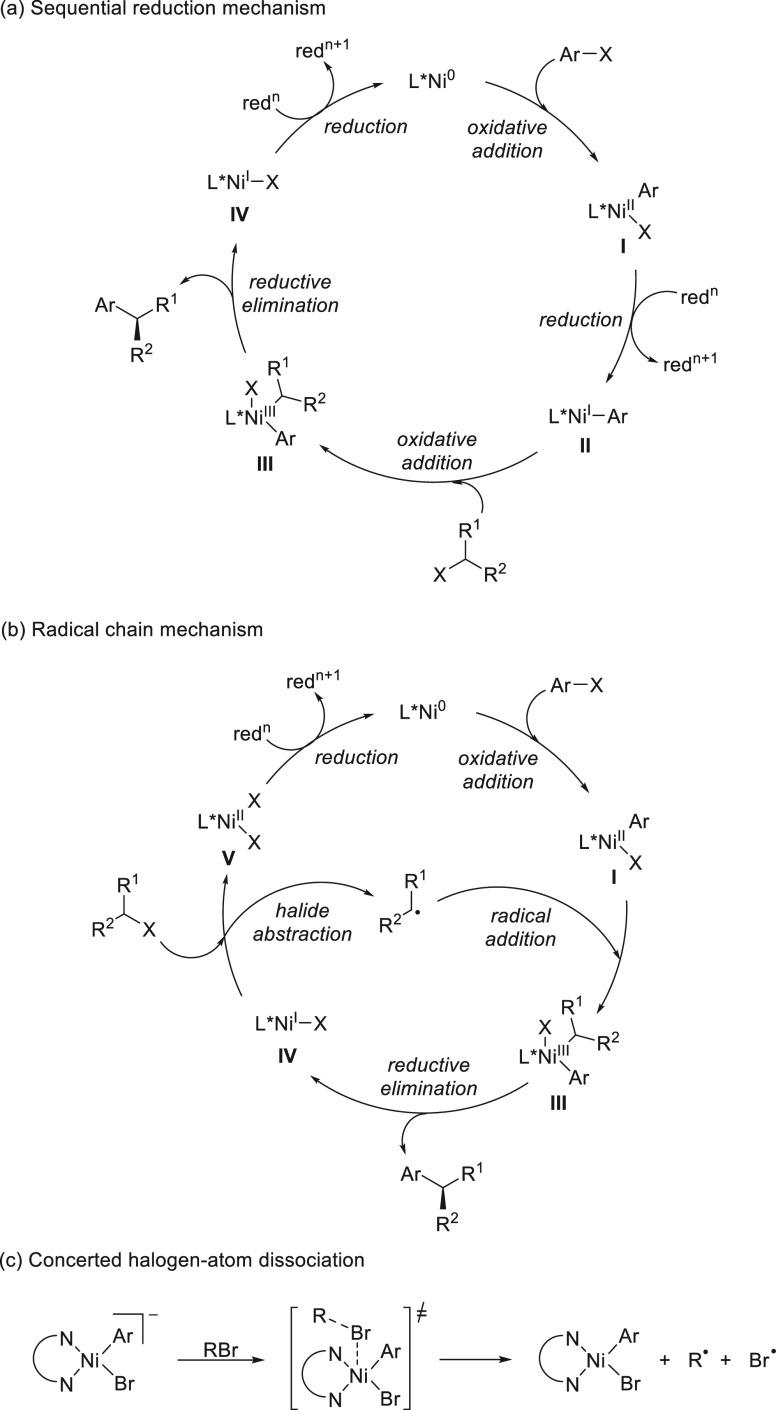
Proposed mechanisms for conventional reductive cross-coupling reactions.

Initial studies on a possible mechanism for conventional RCC reactions are based on a sequential reduction mechanism (Figure 3a) and on a radical chain mechanism (Figure 3b).^142,143^ In the sequential reduction mechanism, the C(sp^2^) electrophile undergoes oxidative addition to Ni(0) preferentially to give the Ni(II) complex **I**, which is then reduced by a metal reductant to Ni(I) intermediate **II**. This complex **II** reacts with the C(sp^3^) electrophile to give the Ni(III) intermediate **III**, which after reductive elimination provides the enantioenriched product and the Ni(I) complex **IV** that, after reduction, regenerates the Ni(0) catalyst. In the radical mechanism, intermediate **I** is formed similarly, which reacts with the alkyl radical to give the Ni(III) complex **III**, precursor of the final product. Subsequent reductive elimination of **III** also generates intermediate **IV**, which undergoes halide abstraction from the alkyl halide to generate the radical and intermediate **V**. Final reduction of **V** regenerates the Ni(0) catalyst.

Diao and co-workers^144^ recently reported electroanalytical and theoretical studies to elucidate the Ni-mediated radical formation in cross-electrophile coupling reactions. Cyclic voltammetry studies on (bpy)Ni(Mes)Br revealed that instead of outer-sphere electron transfer or two-electron oxidative addition pathways, by using (bpy)Ni catalyst proposed for the halogen-atom abstraction pathway, the inner-sphere electron transfer concerted with halogen-atom dissociation (Figure 3c).

**Scheme 57:**
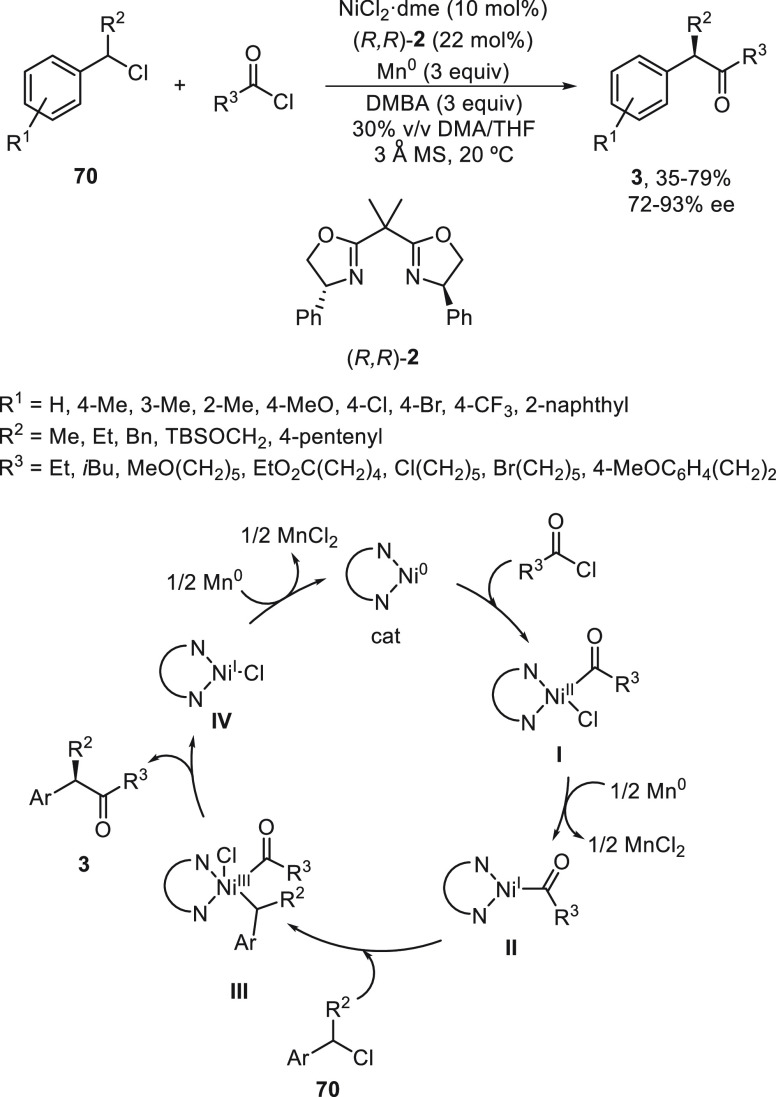
Enantioconvergent Ni-Catalyzed Reductive Acyl Cross-Coupling of Benzylic Chlorides **70**

Reisman and co-workers^145^ reported in 2013 the enantioconvergent acyl cross-coupling of benzylic chlorides **70** with acyl chlorides using NiCl_2_/bis(oxazoline) Ph-Box (*R,R*)-**2** as catalysts and Mn(0) as the terminal reductant (Scheme 57). The corresponding enantioenriched α-substituted ketones **3** were obtained with moderate to good yields and enantioselectivities in a mixture of THF/DMA and in the presence of dimethylbenzoic acid (DMBA) as additive in order to suppress homocoupling of the benzylic chloride. In the proposed mechanism, intermediate **I** results from the oxidative addition of the acid chloride, which could be reduced by Mn(0) to give the Ni(I)-acyl species **II**. Subsequent oxidative addition of a benzyl chloride **70** by a radical process generates the Ni(III) complex **III**, which undergoes reductive elimination to give intermediate **IV** and the ketone.

**Scheme 58:**
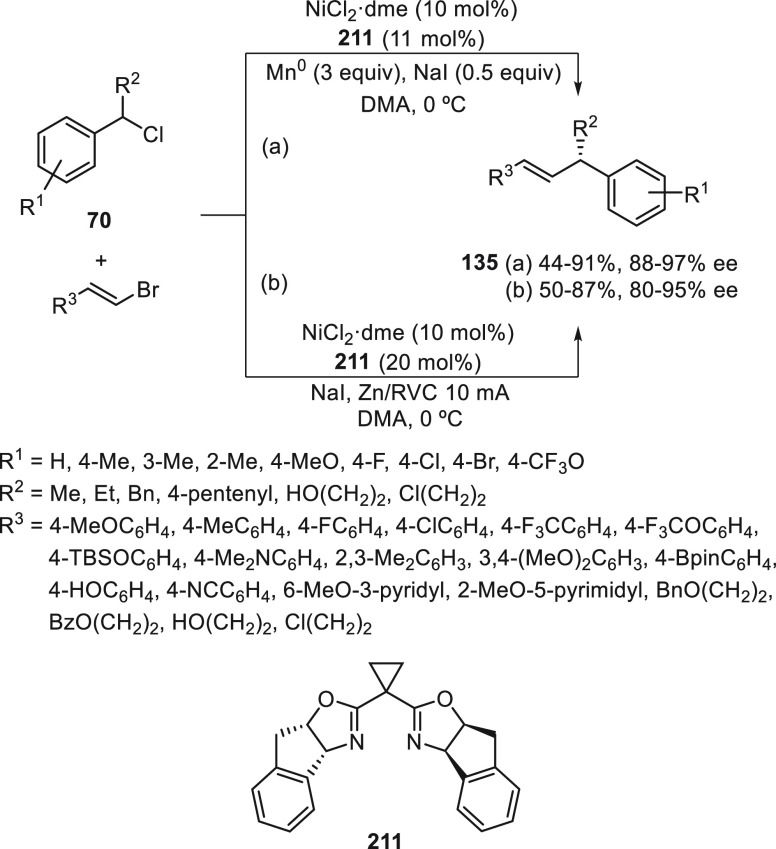
Enantioconvergent Ni-Catalyzed Reductive Cross-Coupling of Benzylic Chlorides **70** with Alkenyl Bromides

The same group reported the enantioconvergent RCC of benzylic chlorides **70** with alkenyl bromides under NiCl_2_/bis(oxazoline) **211** and Mn(0) as terminal reductant (Scheme 58a).^146^ In this case, NaI was an important additive improving the yield of products **135** and decreasing the formation of the dibenzyl homodimer. NaI has been suggested to accelerate the electron-transfer between Mn(0) and Ni or by *in situ* formation of iodide electrophiles.^147^ Alkenes **135** were obtained with good yields and enantioselectivities. This process can be driven electrochemically to avoid the use of metal powder as reducing agent.^148,149^ The corresponding products **135** resulted in up to 87% yield and up to 95% ee (Scheme 58b). Reticulated vitreous carbon (RVC) was used as the cathode, and Zn was used as the sacrificial anode in an undivided cell.^148^

**Scheme 59:**
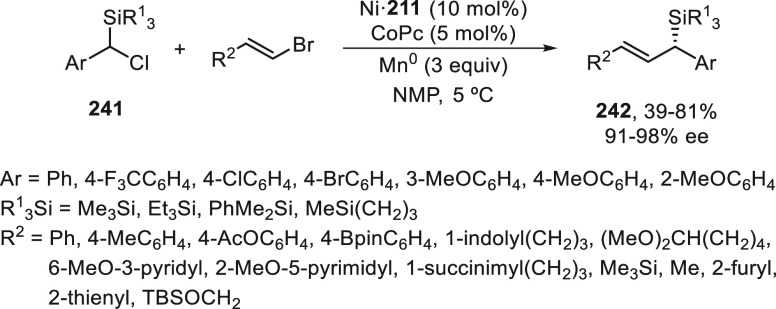
Enantioconvergent Ni-Catalyzed RCC of Chloro(arylmethyl)silanes **241** with Alkenyl Bromides

By Ni-catalyzed RCC, Reisman and co-workers^150^ performed the synthesis of enantioenriched allylic silanes **242** from chloro(arylmethyl)silanes **241** and alkenyl bromides (Scheme 59). In this case, a cobalt phthalocyanine (CoPc) was required for efficient coupling of these bulky benzylic silanes, presumably to favor radical formation.^151^ This RCC took place in the presence of NiCl_2_/bis(oxazoline) **211** and Mn(0) as terminal reductant in NMP at 5 °C to provide allylic silanes **242** in moderate to good yields and high enantioselectivities. Stereospecific transformations of these products were applied to the synthesis of (+)-tashiromine.

**Scheme 60:**
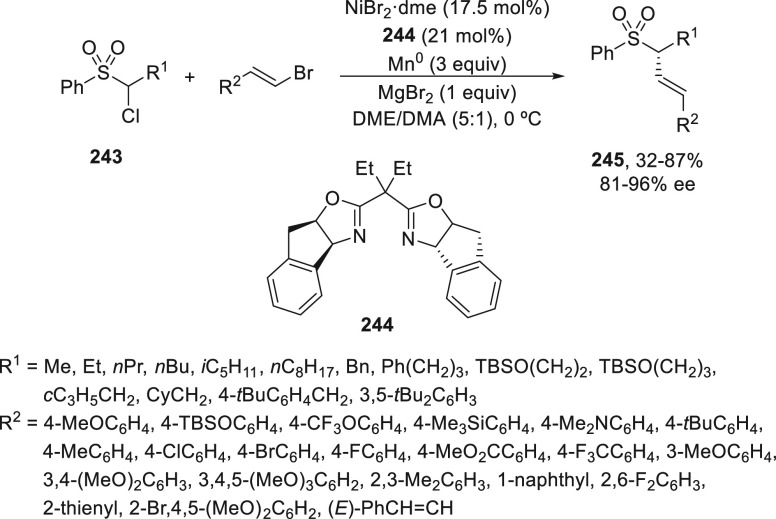
Enantioconvergent Ni-Catalyzed RCC of α-Chloro Sulfones **243** with Alkenyl Bromides

Recently, Sun, Wu, and co-workers^152^ reported the enantioconvergent reductive alkenylation of α-chloro sulfones **243** under NiBr_2_/bis(oxazoline) **244** catalysis, Mn as reductant, and MgBr_2_ as additive (Scheme 60). The resulting enantioenriched allylic sulfones **245** were isolated in up to 87% yield and up to 96% ee and involved radical intermediates.

**Scheme 61:**
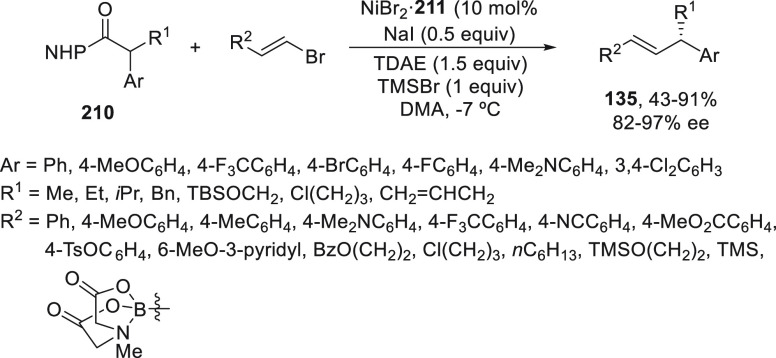
Enantioconvergent Ni-Catalyzed Reductive Decarboxylative Cross-Coupling of *N*-Hydroxyphthalimide Esters **210** with Alkenyl Bromides

Enantioconvergent reductive alkenylation of *N*-hydroxyphthalimide (NHP) esters **210** with alkenyl bromides have been carried out by Reisman and co-workers (Scheme 61).^153^ These esters **210** underwent decarboxylation to generate the corresponding benzylic radicals, which by cross-coupling with alkenyl bromides and using the complex NiBr_2_/bis(oxazoline) **211** as catalyst furnished enantioenriched alkenes **135** in up to 91% yield and up to 97% ee. The reaction uses tetrakis(*N,N*-dimethylamino)ethylene (TDAE) as a terminal organic reductant instead of a large excess of metal(0), TMSBr, and NaI as additives. This procedure is an alternative to the use of benzylic chlorides,^146^ which could be difficult to prepare or unstable, but still gives similar enantioselectivities. According to experimental data, the corresponding mechanism proceeds through a cage-escaped radical.

**Scheme 62:**
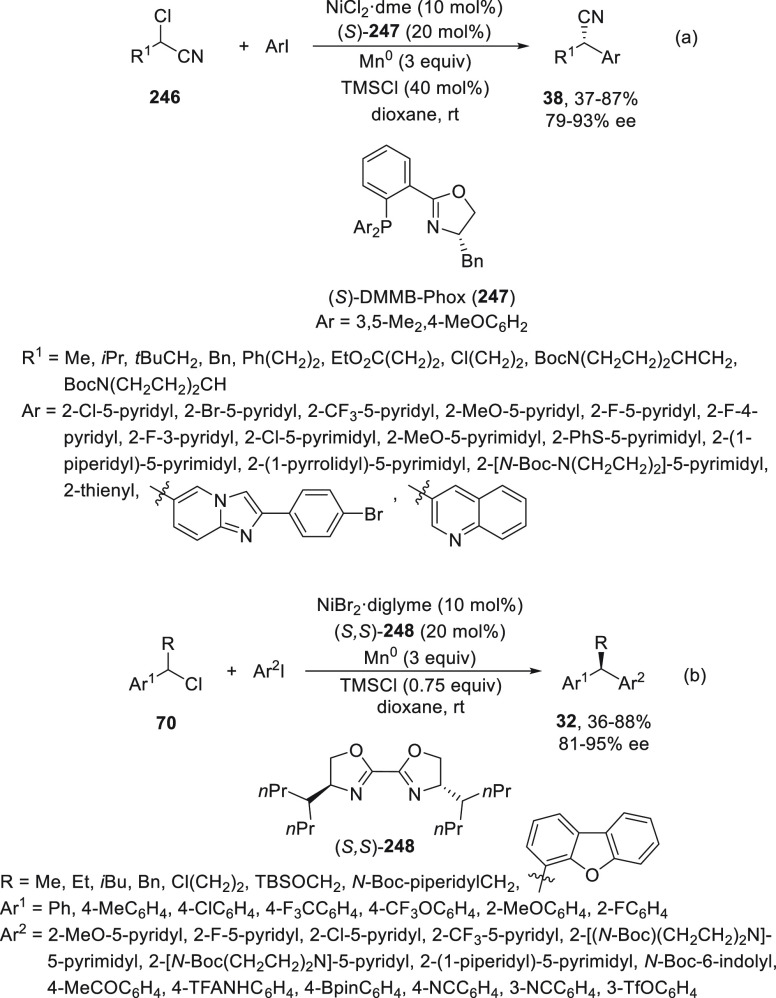
Enantioconvergent Ni-Catalyzed Reductive Cross-Coupling of α-Chloro Nitriles **246** and Benzylic Chlorides **70** with Aryl Iodides

Preliminary enantioconvergent RCC of α-chloroethylbenzene with 4-acetylbromobenzene was described by Weix and co-workers^151^ using NiBr_2_/bis(oxazoline) as chiral catalyst and CoPc as cocatalyst to afford the corresponding diarylethane in 41% yield and 43% ee. In 2015, Kadunce and Reisman^154^ reported the Ni-catalyzed RCC of α-chloro nitriles **246** and aryl iodides (Scheme 62a). This enantioconvergent RCC was performed using NiCl_2_/phosphinoxazoline (*S*)-**247**, Mn(0) as reductant, and TMSCl as additive to provide nitriles **38** in good yields and enantioselectivities. To access 1,1-diarylalkanes **32** from benzylic bromides **70**, a different ligand (*S*)-**248** was used by Reisman and co-workers (Scheme 62b).^155^ Products **32** were obtained under similar reaction conditions as nitriles **38** with moderate to good yields and in up to 95% ee.

**Scheme 63:**
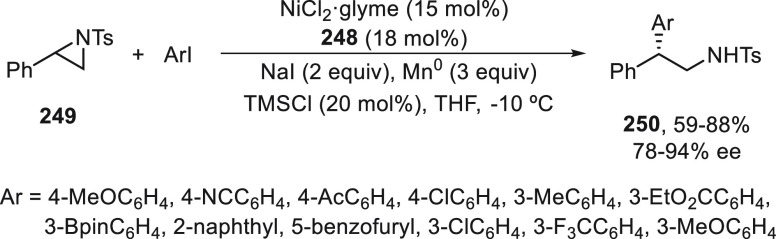
Enantioconvergent Ni-Catalyzed Reductive Cross-Coupling of Styryl Aziridine **249** with Aryl Iodides

Doyle, Sigman, and co-workers^156^ reported the enantioconvergent RCC of racemic styryl aziridine **249** with aryl iodides (Scheme 63). This enantioconvergent C(sp^3^)–C(sp^2^) cross-coupling was carried out under NiCl_2_/bis(oxazoline) **248** catalysis using Mn(0) as a terminal reductant and in the presence of NaI and TMSCl as additives to provide 2,2-diarylethylamines **250** in good yields and enantioselectivities (up to 94%).

**Scheme 64:**
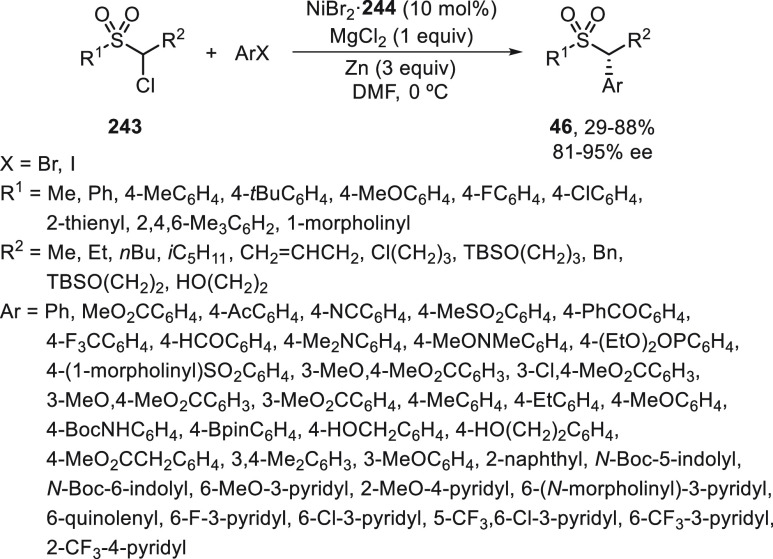
Enantioconvergent Ni-Catalyzed Reductive Cross-Coupling of α-Chloro Sulfones **243** with Aryl Halides

Asymmetric α-sulfonyl arylation of α-chloro sulfones **243** was performed by Lei, Gong, and co-workers.^157^ They used similar reaction conditions to those previously described for the alkenylation of α-chloro sulfones^151^ (see Scheme 60). Enantioconvergent Ni-catalyzed RCC of compounds **243** with aryl bromides and iodides yielded enantioenriched α-arylated sulfones **46** in modest to good yields and good enantioselectivities (Scheme 64). In this case, Zn(0) and, in some cases, Mn(0) were used as terminal reductants in DMF as solvent.

**Scheme 65:**
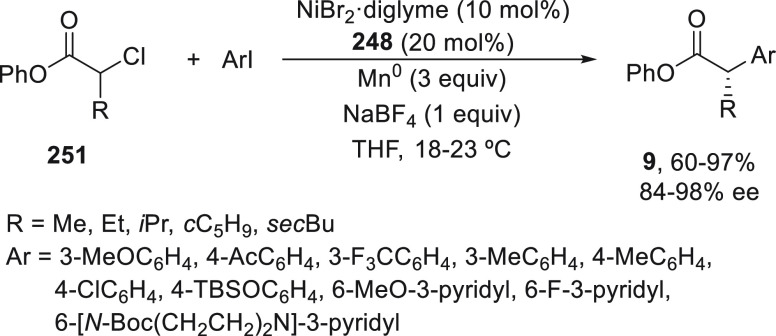
Enantioconvergent Ni-Catalyzed Reductive Cross-Coupling of α-Chloro Esters **251** with Aryl Iodides

For the enantioconvergent RCC of α-chloro esters **251** with aryl iodides (1.5 equiv), Reisman and co-workers^158^ employed NiBr_2_/bis(oxazoline) **248** as catalyst, Mn(0) as terminal reductant, and NaBF_4_ as additive to provide esters **9** in good yields and enantioselectivities (Scheme 65). Under these reaction conditions, even β-branched esters (e.g., R = *i*Pr, *sec*Bu) gave the corresponding α-aryl esters with good yield and high ee. Experimental studies exclude the participation of a manganese enolate generated *in situ* from the ester. This procedure was applied to the synthesis of naproxen by reaction of phenyl α-chloro propionate with 6-methoxy-2-naphthyl iodide to give the corresponding ester with 93% yield and 84% ee. DFT calculations using multivariate linear regression model quantitatively relate the cooperative influence of the α-chloro ester and ligand steric profiles on enantioselectivity.

**Scheme 66:**
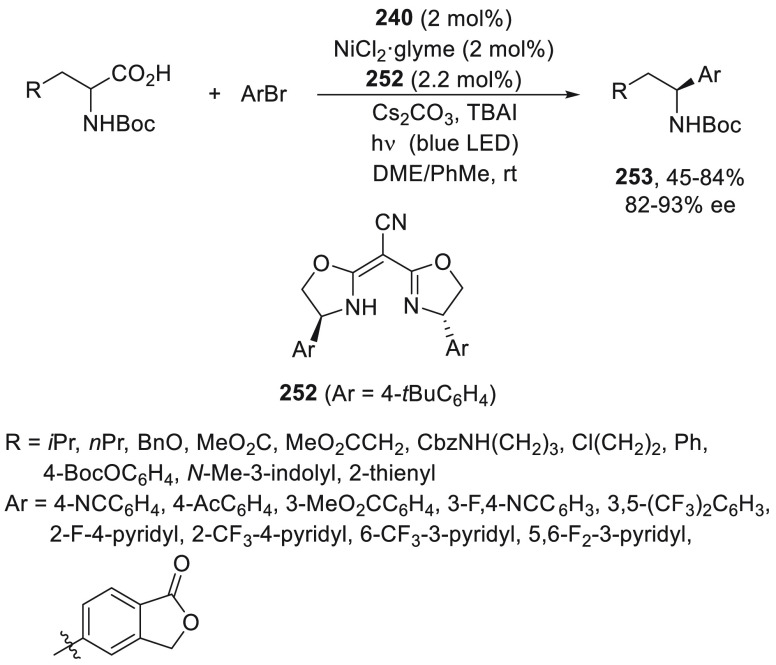
Enantioconvergent Photoredox and Ni-Catalyzed Decarboxylative Cross-Coupling of α-Amino Acids and Aryl Bromides

Dual-nickel/photoredox catalysis^159−161^ is an alternative strategy for challenging cross-electrophile coupling reactions to promote C(sp^3^)–C(sp^2^) bond formation. MacMillan, Doyle, and co-workers^162^ used carboxylic acid as radical precursors for decarboxylative photocatalyzed/Ni-catalyzed cross-coupling to form C(sp^3^)–C(sp^2^) bonds. Enantioconvergent photoredox decarboxylative arylation was carried out by MacMillan, Fu, and co-workers^163^ starting from racemic α-amino acids and aryl bromides (Scheme 66). Using low loadings of the Ir photocatalyst **240** (2 mol %) and NiCl_2_/bis(oxazoline) **252** (2–2.2 mol %) as catalyst, the corresponding *N*-Boc-benzylamines **253** were obtained in up to 84% yield and up to 93% ee. The stereochemical outcome was determined by the configuration of the ligand. This protocol was applied to the synthesis of pharmacophores present in bioactive compounds.

**Scheme 67:**
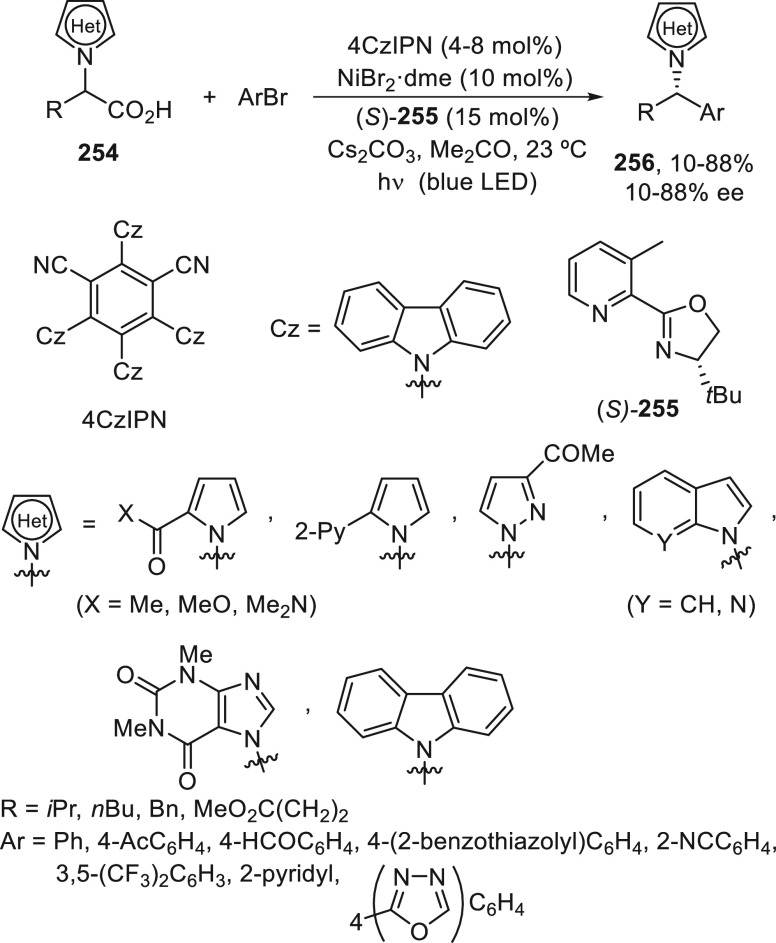
Enantioconvergent Photoredox and Ni-Catalyzed Decarboxylative Cross-Coupling of α-Heterocyclic Carboxylic Acids **254**

In a similar decarboxylative arylation under photoredox/Ni dual catalysis, Davison and co-workers^164^ reported an enantioconvergent synthesis of *N*-benzyl heterocycles **256** from α-heterocyclic carboxylic acids **254** with aryl bromide (Scheme 67). They used an organic photocatalyst 4CzIPN and NiBr_2_/pyridine-oxazoline (*S*)-**255** as dual catalysts for the C(sp^3^)–C(sp^2^) cross-coupling with aryl and hetaryl bromides. The presence of a directing group at the C2 position of the nitrogenated heterocycle increased stereoselectivity in the final product, which was obtained in modest to good yields and up to 88% ee.

**Scheme 68:**
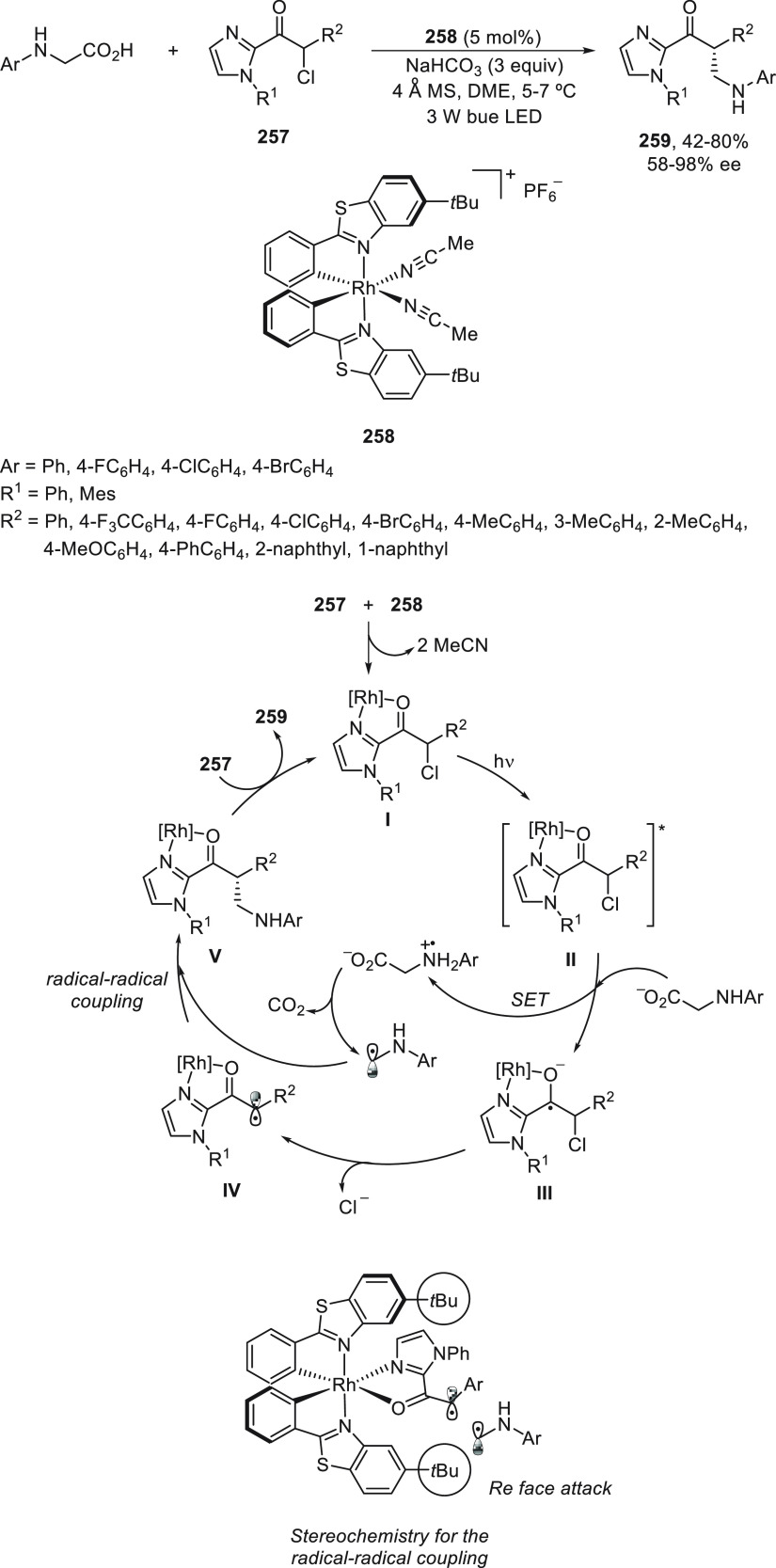
Enantioconvergent Rh-Photocatalyzed Decarboxylative Cross-Coupling of *N*-Aryl Glycines and α-Chloro Imidazol-2-yl Ketones **257**

Racemic α-chloro imidazol-2-yl ketones **257** reacted with *N*-aryl glycines under photoredox/rhodium catalysis to form C(sp^3^)–C(sp^3^) bonds.^165^ This enantioconvergent decarboxylative cross-coupling was performed with a chiral rhodium complex **258**, which serves as chiral Lewis acid and as photoredox active species upon substrate binding under blue LED irradiation. The resulting products **259** were isolated in up to 80% yield and up to 98% ee. On the basis of extensive studies on Ir and Rh catalysts by Meggers’s group,^166,167^ the proposed mechanism is depicted on Scheme 68. By coordination of the ketone **257** with the Rh complex, chelate **I** is formed, which is the photoactive species. Upon irradiation to photoexcited state, intermediate **II** facilitates a SET from *N*-aryl glycinates to provide an amine radical cation and an Rh ketyl **III**. The glycinate radical releases CO_2_ to furnish an α-aminoalkyl radical, which is coupled with a radical intermediate **IV** formed by release of chloride from intermediate **III**, thereby providing the coupling intermediate **V**. Final release of product **259** and coordination with substrate **257** generates the complex **I**. The steric model of the Rh-bound radical intermediate **IV** explains the attack of the α-aminoalkyl radical at the *Re* face.

**Scheme 69:**
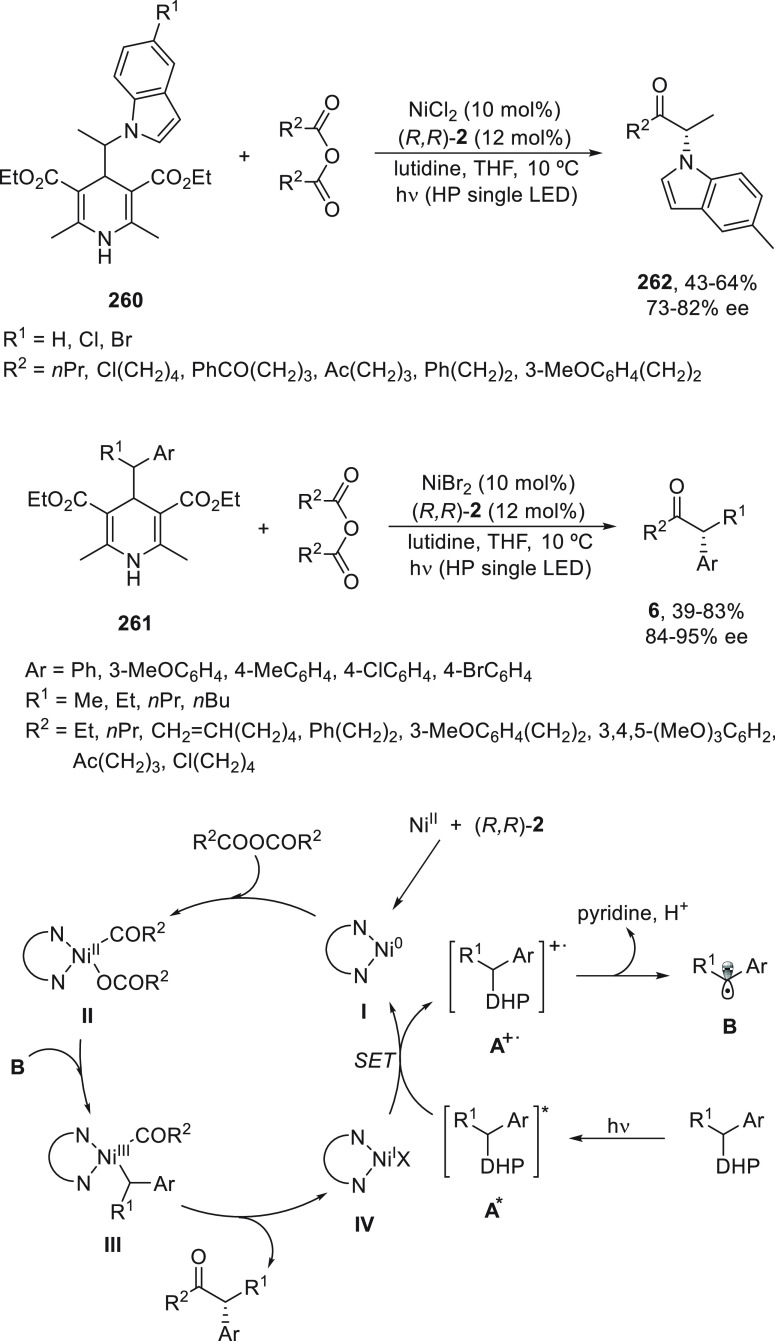
Enantioconvergent Photoredox/Ni-Catalyzed Acyl–Akyl Cross-Coupling of 4-Alkyl-1,4-dihydropyridines **260** and **261** with Anhydrides

Melchiorre and co-workers^168^ used 1,4-dihydropyridines (1,4-DHPs) as photoreductants in the enantioconvergent photoredox acyl cross-coupling of 4-alkyl-1,4-DHPs **260** and **261** with anhydrides (Scheme 69). This process does not require a photocatalyst and was carried out using NiCl_2_/bis(oxazoline) (*R,R*)-**2** as catalyst with a single high-power (HP) LED (λ_max_ = 405 nm) to provide ketones **262** and **6** in up to 83% yield and up to 95% ee. Direct excitation of DHPs at 405 nm gave the excited-state intermediate A*, which participates in two sequential SETs with the Ni catalyst to give the Ni(0) intermediate **I** and the radical cation **A**^**+•**^ [*E*(A^+**•**^/A* = 1.6 V and *E*(Ni^II^/Ni^0^) = −1.2 V]. This unstable **A**^+**•**^ undergoes homolytic cleavage to generate a radical **B**. Oxidative addition into the acyl anhydride would provide the acylNi(II) complex **II**, which by radical trapping of **B** affords the acyl–Ni(III) complex **III**. Subsequent reductive elimination furnishes the ketone and the Ni(I) complex **IV**, which by a SET process from A* regenerates the Ni(0) species **I**.

**Scheme 70:**
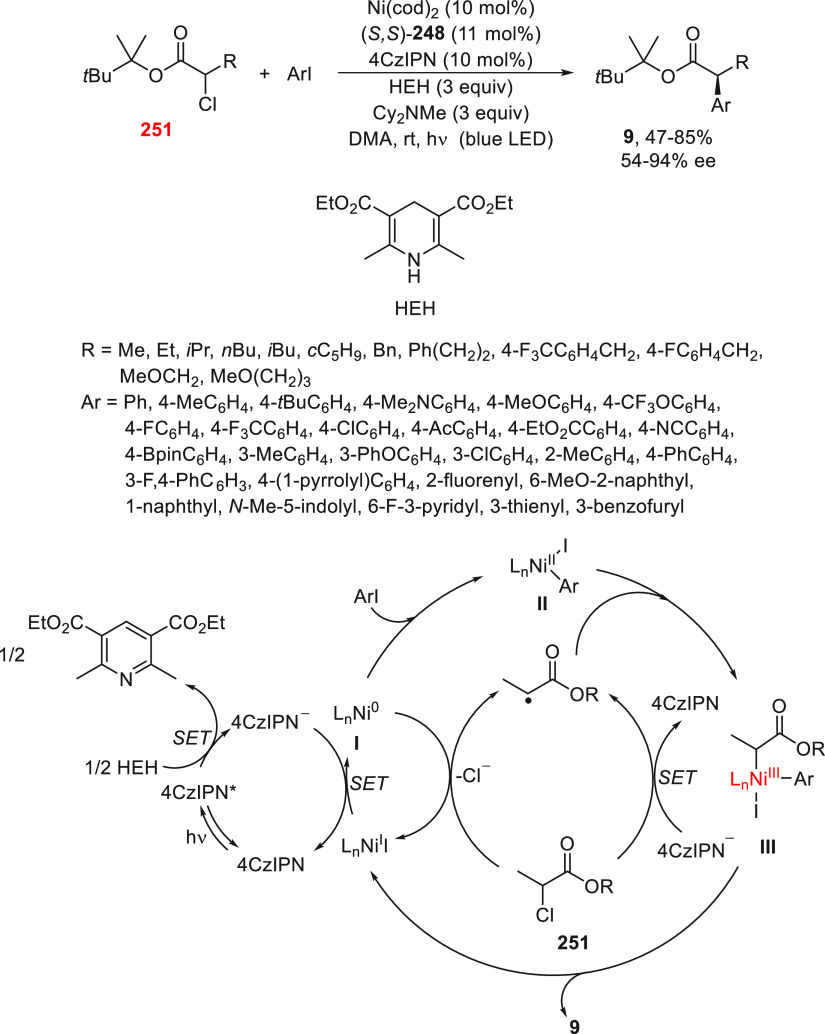
Enantioconvergent Photoredox/Ni-Catalyzed Reductive Cross-Coupling of α-Chloro Esters **251** with Aryl Iodides

An enantioconvergent Ni/photoredox-catalyzed reductive cross-coupling of racemic α-chloro esters **251** with aryl iodides was described by Mao, Walsh, and co-workers.^169^ They employed the organic dye 4CzIPN (see Scheme 67) as photocatalyst and a Hantzsch ester (HEH) as organic reductant instead of metals, such as Mn and Zn (Scheme 70). As in the case of the enantioconvergent arylation of α-chloro esters **251** (Scheme 65), bis(oxazoline) (*S,S*)-**248** was used as ligand to furnish the corresponding α-aryl esters **9** with good yields and ee. A dual-catalytic mechanism has been proposed to explain this process. The aryl iodide undergoes oxidative addition of the Ni(0) species **I** to give complex **II**. Next, the α-chloro ester is reduced by SET to the α-carbonyl radical by the reduced photocatalyst 4CzIPN^–^. This radical is trapped by intermediate **II** to generate the Ni(III) species **III**, which undergoes rapid reductive elimination to form the α-aryl ester. In the left catalytic cycle, 4CzIPN gives by blue light excitation the long-lived photoexcited-state 4CzIPN*, which can be reduced by HEH to give 4CzIPN^–^. The L_*n*_Ni(0) species **I** could be regenerated by a SET from 4CzIPN^–^.

**Scheme 71:**
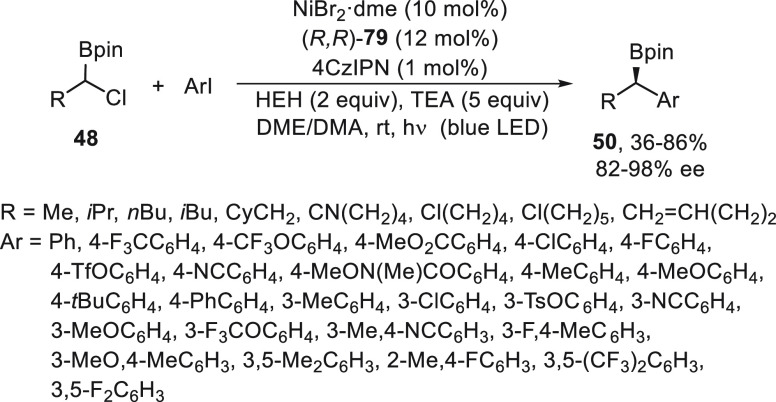
Enantioconvergent Photoredox Ni-Catalyzed Reductive Cross-Coupling of α-Chloroboranes **48** with Aryl Iodides

Asymmetric borylation reactions of benzylic chlorides with B_2_pin_2_ have been carried out under Ni and Cu catalysis by the Fu^130^ (Scheme 51) and Ito^131^ (Scheme 52) groups, respectively. Recently, Xu and co-workers^170^ reported the photoredox/Ni-catalyzed reductive cross-coupling of aryl iodides and α-chloroboranes **48** to furnish benzylic boronic esters **50** with excellent enantioselectivities (Scheme 71). In this case, NiBr_2_/diamine (*R,R*)-**79** as chiral catalyst, 4CzIPN as photocatalyst, and HEH as reductant were used. A similar mechanism as depicted in Scheme 70 has been proposed.

**Scheme 72:**
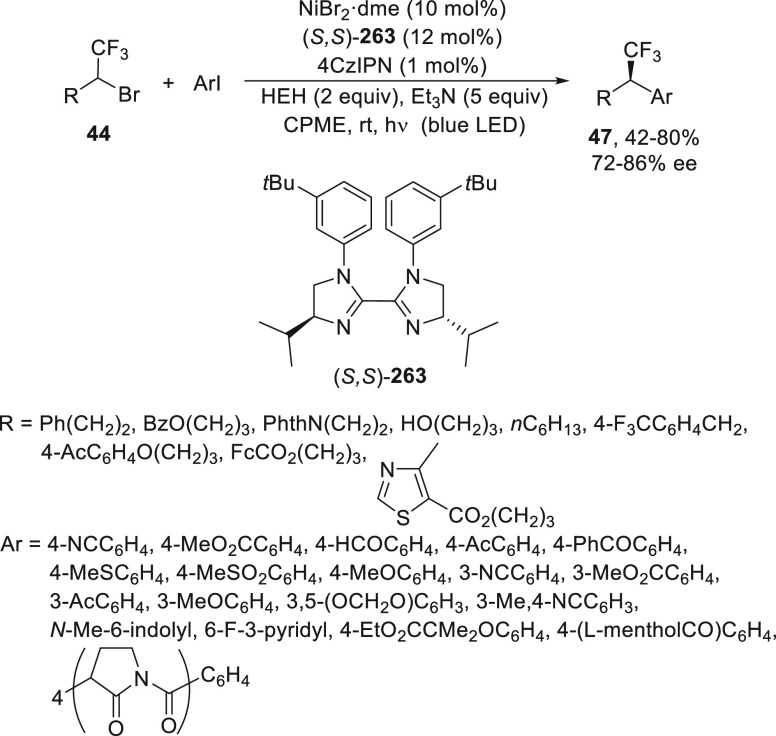
Enantioconvergent Photoredox/Ni-Catalyzed Reductive Cross-Coupling of α-CF_3_-Substituted Alkyl Bromides **44** with Aryl Iodides

The same group applied this Ni/photoredox methodology to the synthesis of enantioenriched trifluoromethylated alkanes **47** (Scheme 72).^171^ Reductive cross-coupling of aryl iodides with racemic α-CF_3_-substituted alkyl bromides **44** was carried out in the presence of the chiral bis(imidazoline) (*S,S*)-**263**^172^ to provide products **47** in good yields and enantioselectivities under mild conditions. Aryl bromides can be also used to give the products with lower yields. Aryl iodides derived from drugs, such as clofibrate and aniracetam, were transformed into CF_3_-containing derivatives.

**Scheme 73:**
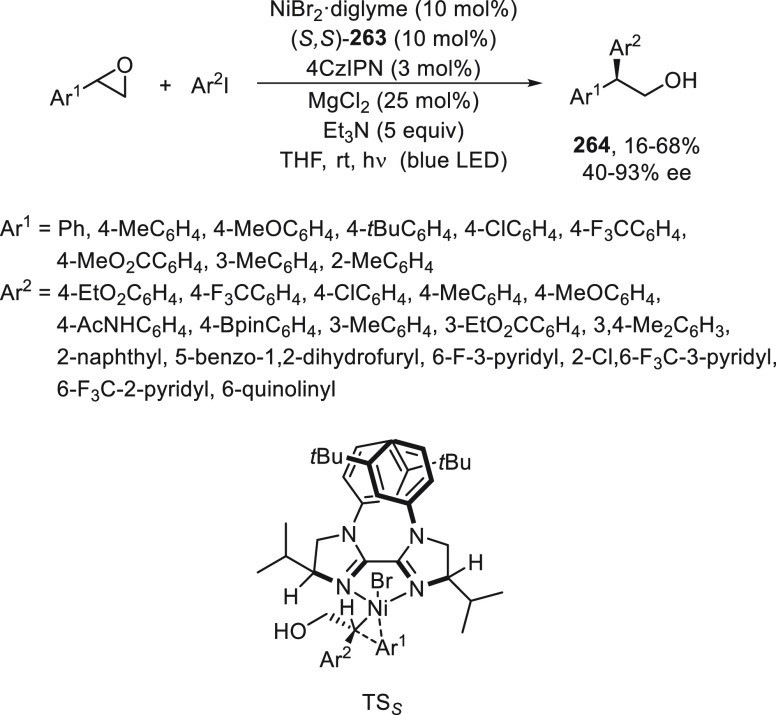
Enantioconvergent Photoredox/Ni-Catalyzed Reductive Cross-Coupling of Styrene Oxides with Aryl Iodides

Doyle and co-workers^173,174^ reported a Ni/photoredox-catalyzed enantioconvergent RCC of styrene oxides with aryl iodides. Initial studies^173^ were carried out with different epoxides using NiBr_2_/Cp_2_TiCl_2_ and 4CzIPN as catalysts. In the enantioconvergent version,^174^ NiBr_2_/bis(imidazoline) (*S,S*)-**263** and 4CzIPN were used as catalysts, and MgBr_2_ was used as Lewis acid (Scheme 73). Enantioenriched 2,2-diarylethanols **264** resulted with high enantioselectivity in correlation with electronic properties of the assayed ligands. Experimental and theoretical mechanistic studies supported that the reductive elimination step is enantiodetermining and that TS_*S*_ is 1.7 kcal/mol less in energy compared with TS_*R*_. One example with 4-ethoxycarbonylphenyl bromide gave the corresponding alcohol **264** with 60% yield and 88% ee. In addition, *N*-tosyl styrene aziridine **249** reacted with 4-acetylphenyl iodide to furnish the β,β-diaryl *N*-tosylethanamine **250** with 48% yield and 83% ee.

**Scheme 74:**
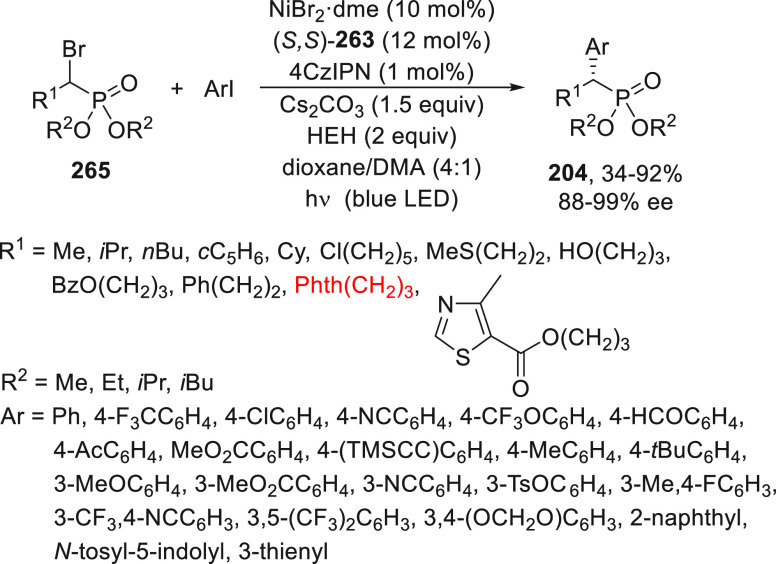
Enantioconvergent Photoredox Ni-Catalyzed Reductive Cross-Coupling of α-Bromophosphonates **265** with Aryl Iodides

Recently, XU, Li and co-workers^175^ applied the previously described photoredox/Ni-catalyzed conditions^170^ to the synthesis of enantioenriched α-aryl phosphonates **204** by arylation of α-bromophosphonates **265**. A wide range of substrates were transformed into products **204** in good to excellent yields and enantioselectivities using NiBr_2_/bis(imidazoline) (*S,S*)-**263** with HEH as an organic reductant (Scheme 74). From DFT calculations it was found that the oxidative addition of the alkyl radical to the LNi(II) species, and not the reductive elimination, was the enantiodetermining step.

Conventional Ni-catalyzed enantioconvergent reductive cross-coupling reaction using mainly bis(oxazolines) as chiral ligands allowed the C(sp^3^)–C(sp^2^) bond formation of two electrophiles (alkyl with aryl or vinyl halides) in the presence of a terminal reductant. This methodology avoids the use of organometallic reagents and has been used for the formation of stabilized intermediate alkyl radicals, such as benzylic and α-substituted ones, bearing a silyl, sulfonyl or a cyano group. The combination of visible-light photoredox and Ni as dual catalysts is a novel strategy for these reductive cross-coupling reactions, which avoids the use of an excess of metal as terminal reductant. In this case, stabilized radicals are generated with α-amino, α-alkoxycarbonyl, α-boronates, α-trifluoromethyl, and α-phosphoryl groups. The use of bis(oxazolines) as chiral ligand allowed a high control of the enantioselectivity in the arylation of racemic alkyl electrophiles. Organic photocatalysis and reductants have been successfully used for these photoredox Ni-catalyzed transformations.

## 3 Enantioconvergent Allylic Cross-Couplings

**Scheme 75:**
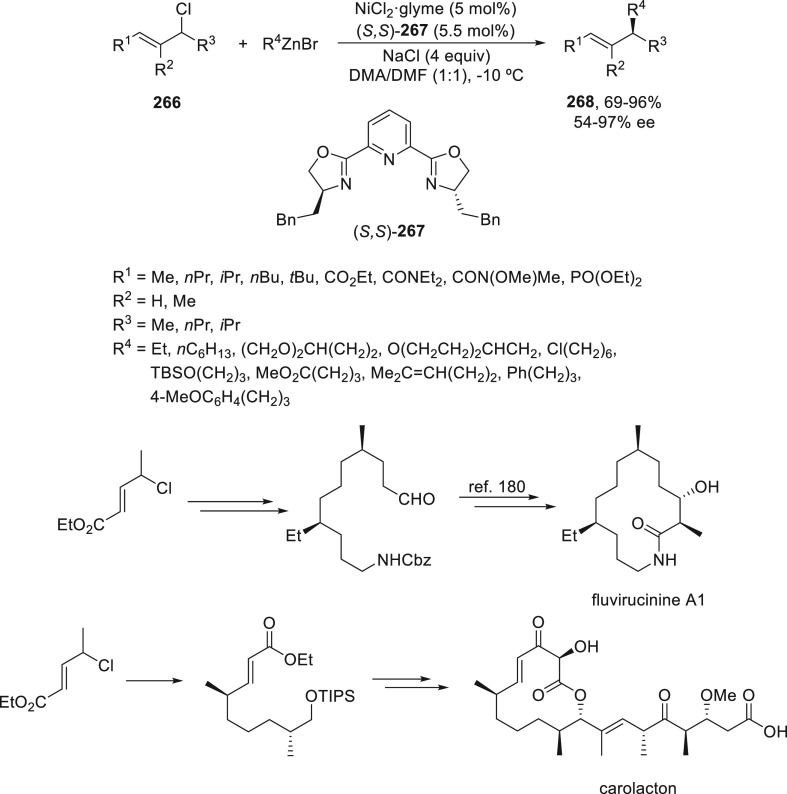
Enantioconvergent Ni-Catalyzed Negishi Reaction of Allylic Chlorides **266** with Alkylzinc Reagents

Allylic systems undergo asymmetric allylic substitution reactions mainly under palladium catalysis through DyKAT processes.^20−22,24,176−178^ Allylic electrophiles are challenging substrates because they face several kinds of selectivity issues, such as chemo-, regio-, *Z/E*-stereo-, diastereo-, and enantioselectivity. An enantioconvergent cross-coupling reaction of racemic secondary allylic chlorides **266** with alkylzincs was described by Son and Fu in 2008.^179^ This Negishi reaction was carried out under NiCl_2_/bis(oxazoline) (*S,S*)-**267** catalysis at −10 °C to provide products **268** in good yields and selectivities (Scheme 75). The enantioselectivity depends strongly on the substituent at the α-position of the chloride. Good ee values are obtained when symmetrical allylic chlorides have sterically low-demand substituents. Unsymmetrical allylic chlorides with R^3^ = Me and R^1^ = *n*Bu gave regioselectivity favoring the reaction proximal to the methyl substituent in a ratio 1.9:1. This method was applied to the synthesis of a precursor of the macrocycle fluvirucinine A_1_.^180^ Schmidt and Kirschning^181^ used the same allylic chloride **266** (R^1^ = CO_2_Et, R^3^ = Me) for the synthesis of carolacton, which reduces the number of viable cells in biofilms at nanomolar concentration.

**Scheme 76:**
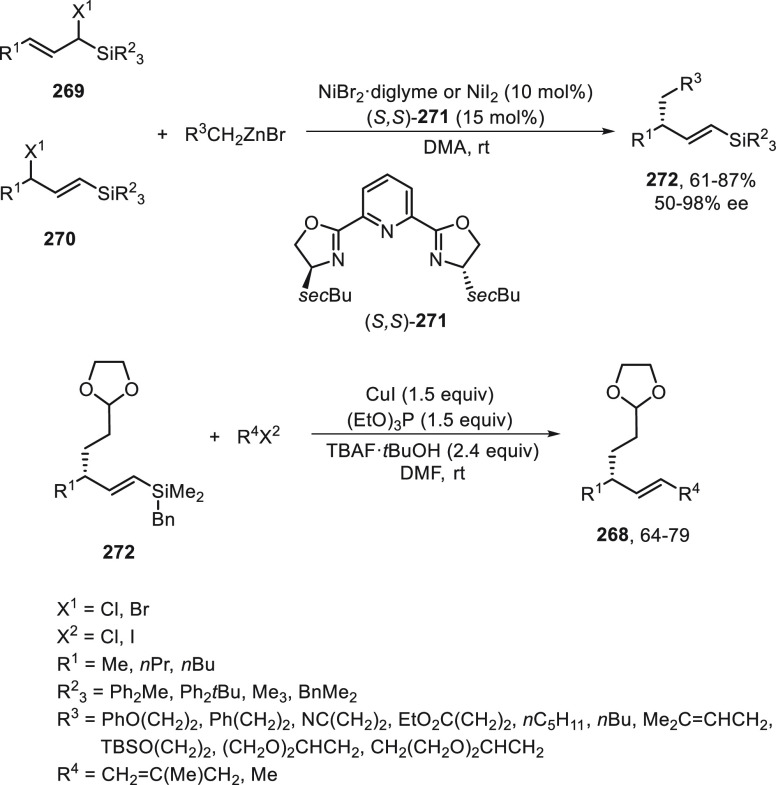
Enantioconvergent Ni-Catalyzed Negishi Reaction of Silylated Allylic Halides **269** and **270** with Alkylzinc Bromides

Enantioconvergent Negishi cross-coupling of regioisomeric mixtures of silylated allylic halides **269** and **270** (*E/Z* > 98:2) to provide enantioenriched vinylsilanes **272** has been described by Oestreich and co-workers (Scheme 76).^182^ The reaction was performed with alkylzinc bromides NiBr_2_ or NiI_2_/Pybox (*S,S*)-**271** as catalysts in DMA at room temperature to give products **272** with good yields and enantioselectivities. The controlling element for the regioconvergence was the silyl group. Following the protocol of Tsubouchi and co-workers^183^ the cross-coupling of the obtained vinylsilanes **272** with a BnMe_2_Si substituent with alkyl electrophiles was carried out with alkyl halides to give products **268** with retention of the configuration in the stereocenter and in the C–C double bond. This two-step process is an alternative to the previously described Negishi reaction by Fu and co-workers^179^ for the synthesis of enantioenriched 1,3-dialkyl-substituted acyclic allylic systems **268**.

**Scheme 77:**
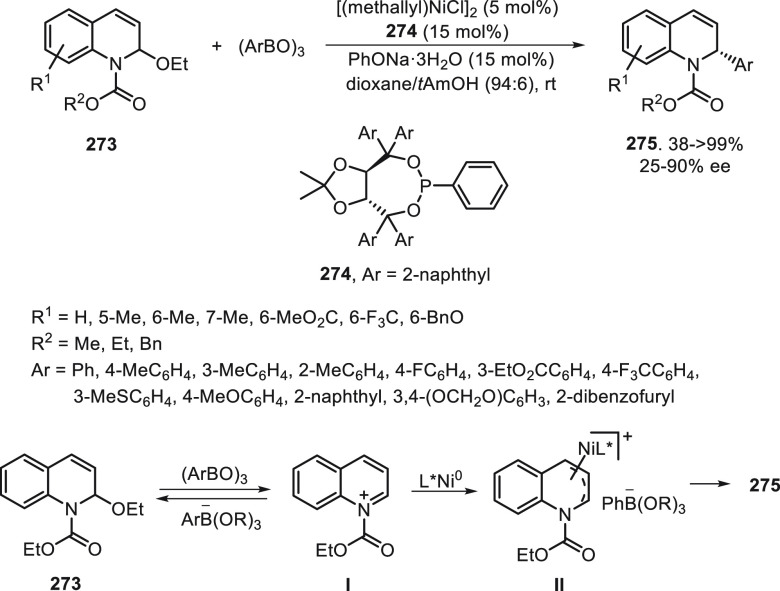
Enantioconvergent Ni-Catalyzed Suzuki Cross-Coupling of 2-Ethoxy-1,2-dihydroquinolines **273** with Arylboroxines

Doyle and co-workers^184^ have reported the Ni-catalyzed enantioconvergent Suzuki cross-coupling of 2-ethoxy-1-ethoxycarbonyl-1,2-dihydroquinolines **273** with arylboroxines. This arylation of quinolinium intermediates was carried out in the presence of α,α,α′,α′-tetraaryl-2,2-disubstituted 1,3-dioxolane-4,5-dimethanol (TADDOL)-derived phosphonate **274** as chiral ligand to provide 2-substituted dihydroquinoline derivatives **275** in moderate to high yields and enantioselectivities (Scheme 77). According to previously developed mechanistic studies,^185^ initial formation of a quinolinium intermediate **I** is facilitated by Lewis acid assistance from the arylboroxine via an S_N_1-like mechanism, followed by an unusual ionic oxidative addition of the Ni(0) complex to afford the Ni(II) complex **II**. Final reaction of **II** with ArB(OR)_3_^–^ gives rise to products **275**.

## 4 Enantioconvergent Propargylic Alkylations

**Scheme 78:**
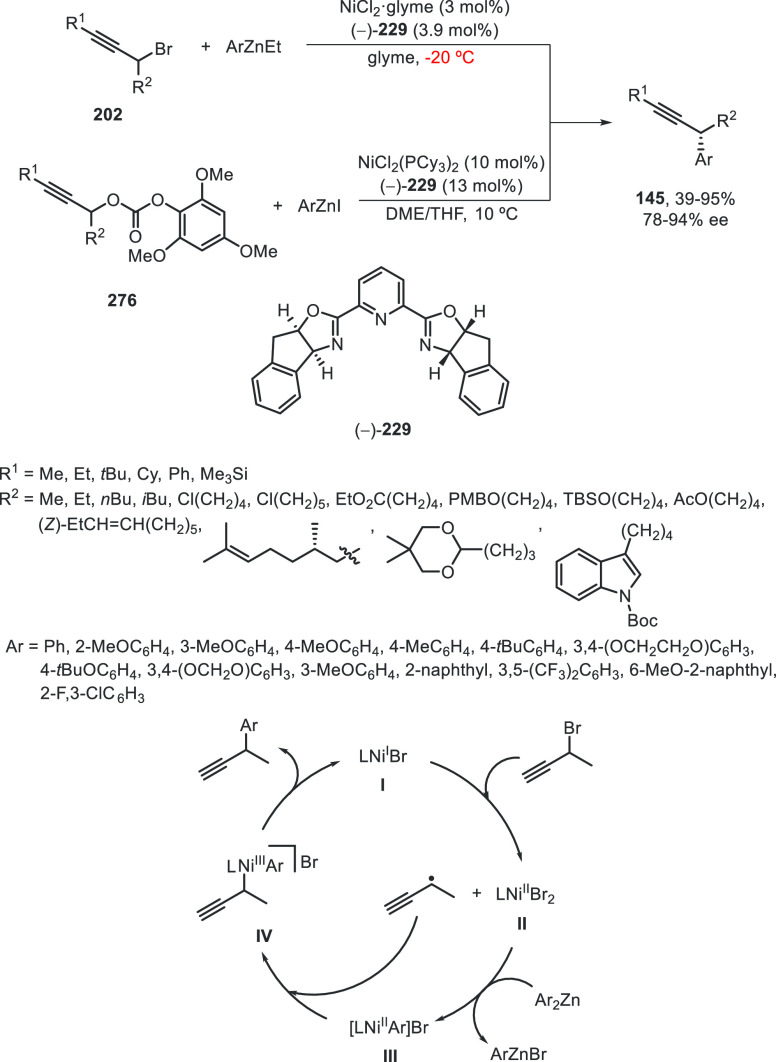
Enantioconvergent Ni-Catalyzed Negishi Reactions of Propargylic Bromides **202** and Carbonates **276** with Arylzinc Reagents

Smith and Fu reported in 2008 the first enantioconvergent Negishi cross-coupling of secondary propargylic bromides **202** with arylzinc reagents ArZnEt under NiCl_2_/bis(oxazoline) (−)-**229** catalysis.^186^ The reaction took place with 5 mol % of NiCl_2_ in glyme at −20 °C to provide alkynes **145** with up to 93% yield and 94% ee (Scheme 78). The same group^187^ extended this Negishi reaction of propargylic carbonates **276** with ArZnI using 10 mol % of NiCl_2_(PCy_3_)_2_ and 13 mol % of Pybox (−)-**229** as chiral ligand in a 1:1 mixture of DME/THF at 10 °C. The resulting alkynes **145** were obtained in up to 95% yield and 93% ee (Scheme 78). This method was also applied to the cross-coupling of racemic TMS-protected propargylic bromides and chlorides.^187^ Mechanistic studies on the enantioconvergent cross-coupling of propargyl bromides with arylzinc reagents revealed the formation of propargyl radical through an inner-sphere electron transfer reaction with a Ni(I) complex **I**.^188^ The resulting LNiBr_2_ complex **II** reacts with diarylzinc to provide complex **III**, which reacts with the propargyl radical to give intermediate **IV**. Final reductive elimination furnishes the product and regenerates complex **I** (Scheme 78). In the case of propargylic carbonates, for which a direct S_N_2 reaction is not viable, the authors suggested that the Ni(I) complex **I** adds to the carbonyl group to generate a (nickel)ketyl, which then fragments to form the propargyl radical.

**Scheme 79:**
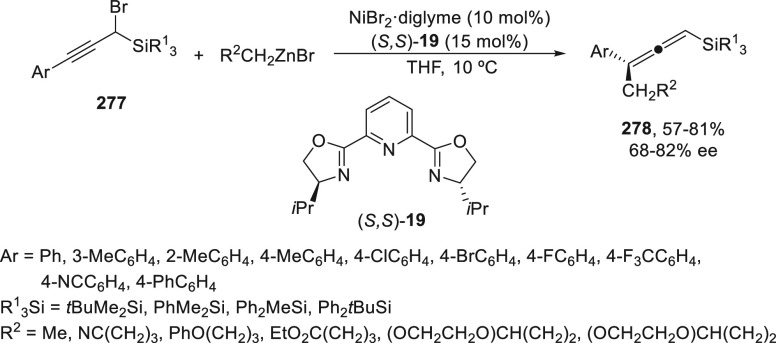
Enantioconvergent Ni-Catalyzed Negishi Reaction of α-Silylated Propargylic Bromides **277** with Primary Alkylzinc Reagents

When propargylic bromides **277** have a silyl moiety at the α-position, an enantioconvergent Ni-catalyzed Negishi reaction results in enantioenriched allenylsilanes **278** (Scheme 79).^189^ In this case, NiBr_2_/Pybox (*S,S*)-**19** was used as catalyst for the cross-coupling with primary alkylzinc reagents to regioselectively form allenylsilanes **278** with good yields and moderate enantioselectivities. The high regioselectivity is because of the bulky silyl group directing the cross-coupling at the γ-position of the propargylic system.

**Scheme 80:**
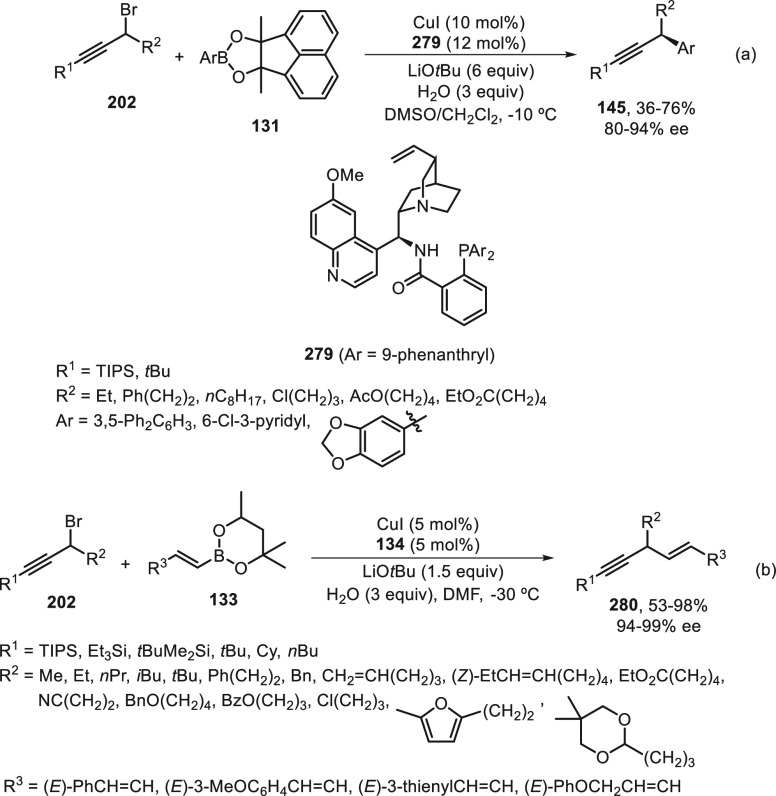
Enantioconvergent Cu-Catalyzed Suzuki Reactions of Propargylic Bromides **202** with Aryl and Alkenyl Boronates **131** and **133**, Respectively

As it was mentioned in Section 2.1.3, Li, Liu, and co-workers^89,90^ reported the enantioconvergent Cu-catalyzed Suzuki reactions of benzylic bromides **19** with aryl and alkenyl boronates **131**(89) and **133**(90) (Scheme 27). These authors also reported the arylation of propargylic bromides **202** with aryl or heteroaryl B(mac)-derived boronate esters **131** using CuI/N,N,P-ligand **279** (Ar = 9-phenantryl) as catalyst (Scheme 80a).^89^ Enantioenriched alkynes **145** were obtained in up to 76% yield and 94% ee. Less reactive propargyl chloride of type **202** [R^1^ = TIPS, R^2^ = Ph(CH_2_)_2_] reacted with 3,5-diphenylB(mac) to give the corresponding alkyne with 42% yield and 95% ee. Alkenyl methylpentanediol (mp)-derived boronate esters **133** were allowed to react with propargylic bromides under the same reaction conditions as benzylic bromides but with ligand **134** (Scheme 24b) to provide enynes **280** in up to 98% yield and 99% ee (Scheme 80b).^90^

**Scheme 81:**
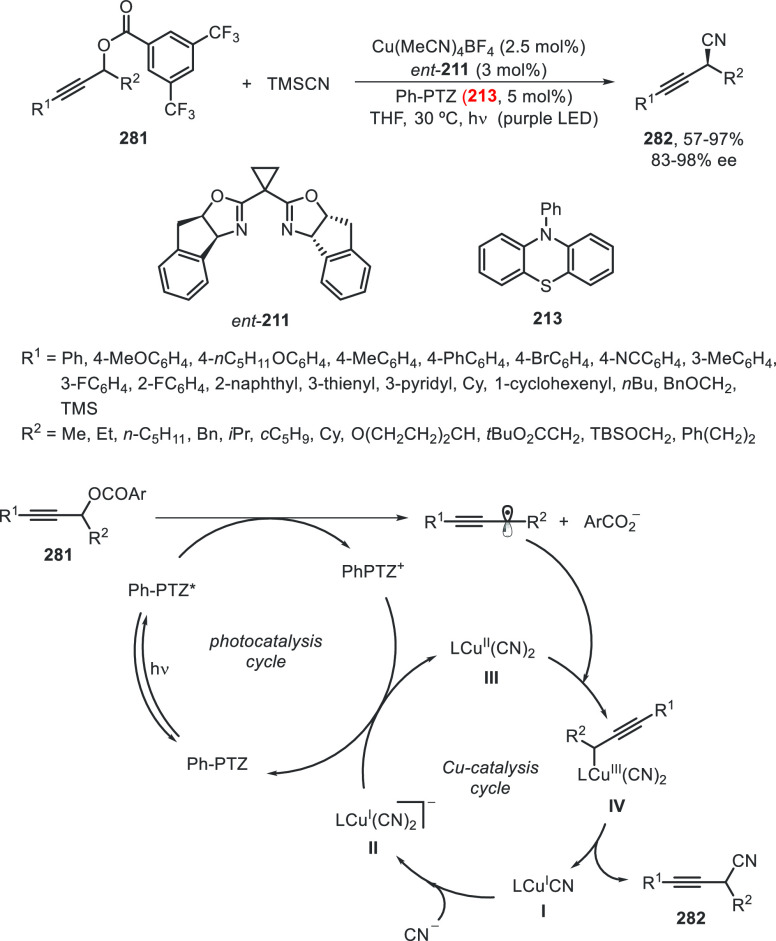
Enantioconvergent Photoredox Cu-Catalyzed Cyanation of Propargylic Esters **281**

Lu, Lan, Xiao, and co-workers^190^ have developed an enantioconvergent propargylic radical cyanation via a synergetic photoredox/copper catalysis strategy. They employed an organophotocatalyst Ph-PTZ (**213**) to generate propargyl radicals and oxidize Cu(I) species to Cu(II) ones. Propargyl esters **281** were allowed to react with TMSCN using Cu(MeCN)_4_BF_4_/bis(oxazoline) *ent*-**211** as chiral catalyst in THF at 30 °C under the irradiation of 2 × 3 W purple LED to give enantioenriched propargylic cyanides **282** in up to 97% yield and 98% ee (Scheme 81). Mechanistic studies based on experiments and DFT calculations suggested that the propargylic ester accepts a single electron from the excited state of Ph-PTZ* to generate propargyl radical and a carboxylate anion. This radical can be captured by LCu^II^(CN)_2_ (**III**) to form the Cu(III) **IV**. Reductive elimination of intermediate **IV** would deliver the propargylic cyanide and regenerate the chiral Cu(I) catalyst **I**. A cyanide anion can be released from TMSCN by the Cu(I) catalyst to form LCu^I^(CN)_2_^–^ (**II**) species.

## 5 Enantioconvergent C–H Functionalization

Regioselective C–H functionalization processes are important strategies in the direct enantioselective reaction of organic molecules, mainly under transition metal catalysis and especially by means of C–H activation.^191−198^ This functionalization represents an atom- and step-economic procedure for the generation of structural complexity. In this Section, enantioconvergent C(sp^2^)–H, C(sp^3^)–H, and C–H allylic functionalizations will be considered.

### 5.1 C(sp^2^)–H Functionalization

**Scheme 82:**
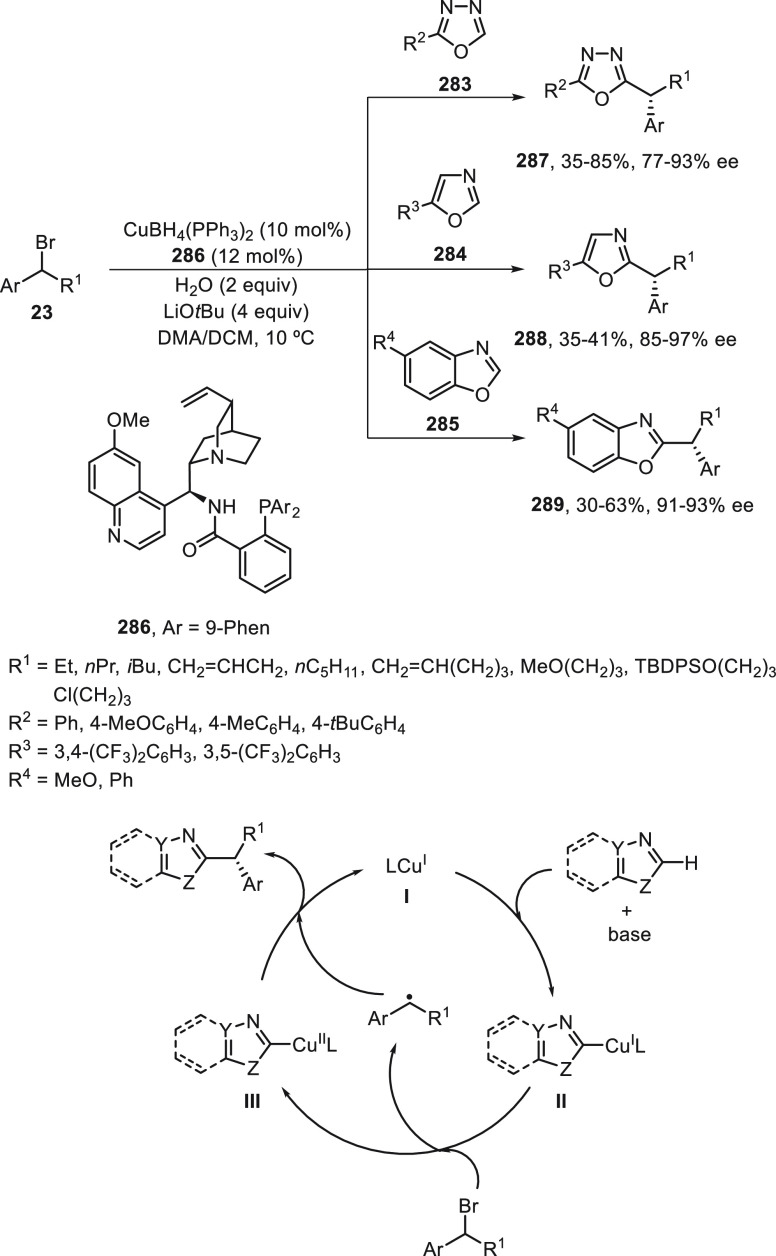
Enantioconvergent Cu-Catalyzed Cross-Coupling of Benzylic Bromides **23** with Azoles **283**–**285**

Very recently, enantioconvergent cross-coupling of racemic alkyl bromides with azole C(sp^2^)–H bonds has been disclosed.^197^ This copper-catalyzed heteroarylation of benzylic bromides **23** has been carried out with azoles, such as 1,3,4-oxadiazoles **283**, oxazoles **284**, and benzo[*d*]oxazoles **285** (Scheme 82). CuBH_4_(PPh_3_)_2_/*Cinchona* alkaloid-derived N,N,P-ligand **286** was the appropriate catalyst in the presence of LiO*t*Bu as base and H_2_O at 10 °C in DMA/DCM. The resulting enantioenriched azoles **287**–**289** were obtained in moderate to good yields and enantioselectivities and used for drug discovery. From experimental essays, a tentative mechanism has been proposed in which the LCu(I) complex **I** reacts with the azole to give intermediate **II**. This intermediate provides the alkyl radical and the Cu(II) complex **III**. Finally, after a C(sp^3^)–C(sp^2^) coupling via a Cu(III) intermediate and its reductive elimination affords the product.

**Scheme 83:**
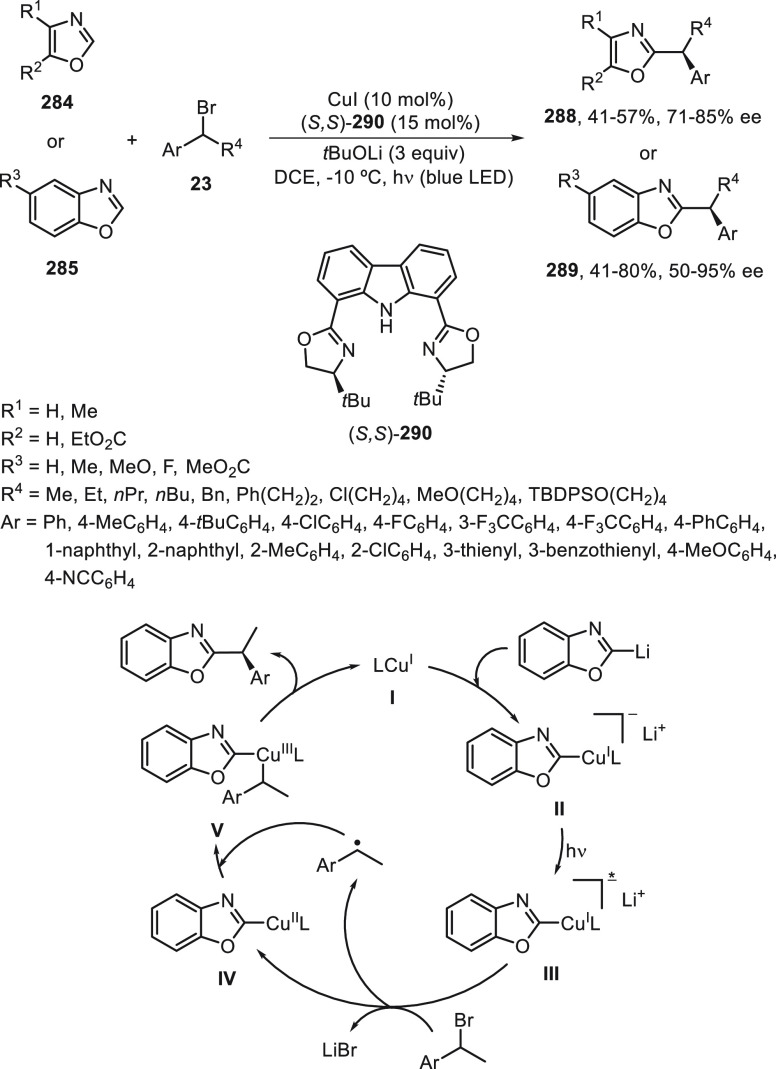
Enantioconvergent Photo- and Cu-Catalyzed Cross-Coupling of Benzylic Bromides **23** with Oxazoles **284** and **285**

Independently, Li, Chen, Zhang, and co-workers^198^ reported a similar enantioconvergent alkylation of azoles under blue-light-promoted reaction conditions. Oxazoles **284** and benzoxazoles **285** reacted with secondary benzylic bromides **23** using CuI/bis(oxazoline) (*S,S*)-**290** as a photo- and chiral catalyst and *t*BuOLi as base in DCE at −10 °C under blue LED irradiation (Scheme 83). The resulting C–H functionalized oxazoles **288** and benzoxazoles **289** were obtained with moderate to good yields and enantioselectivities. In the proposed mechanism, complex **I** is formed *in situ* and undergoes transmetalation with Li-azole to generate intermediate **II**. After photoexcitation, species **III** is formed, which delivers by electron transfer LiBr and complex **IV**. Subsequent transformation of complex **IV** into intermediate **V** via enantioselective radical trapping followed by reductive elimination from the Cu(III) center leads to product. Alternatively, intermediate **IV** can give the product through a direct SET process.

**Scheme 84:**
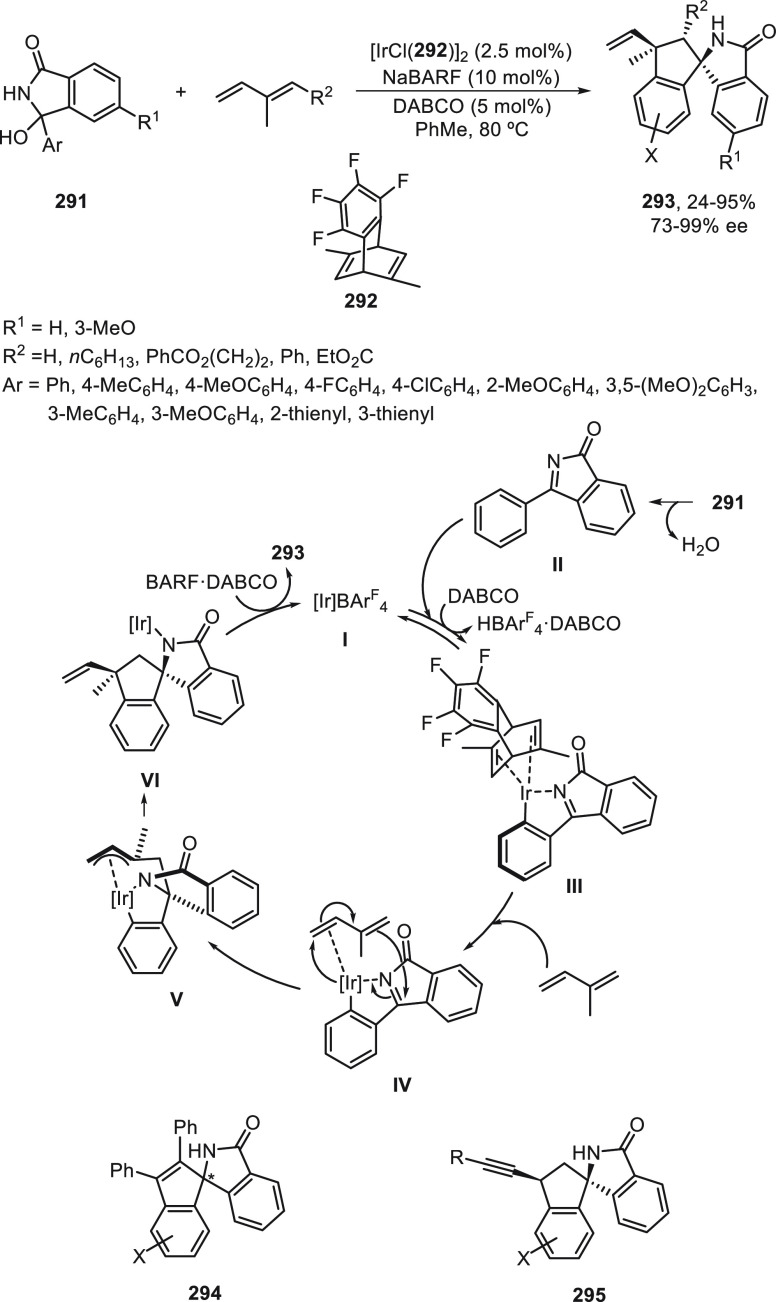
Enantioconvergent Ir-Catalyzed Annulation of 3-Aryl-3-hydroxyisoindolin-1-ones **291** with 1,3-Dienes

Enantioconvergent [3 + 2] annulation between 1,3-dienes and *N*-acyl ketimines, generated *in situ* from 3-aryl-3-hydroxyisoindolin-1-ones **291**, proceeded via C(sp^2^)–H activation under an Ir/chiral diene **292** complex as catalyst (Scheme 84).^199^ This annulation gave spiroaminoindane derivatives **293** in high yields with high regio- and enantioselectivities. The catalytic cycle is postulated by C–H activation at the *ortho* position of ketimine **II** by oxidative addition of the C–H bond to Ir and deprotonation by 1,4-diazabicyclo[2.2.2]octane (DABCO) to provide arylindium(I) species **III**. The diene, e.g., isoprene, approaches the Ir center from the *Re* face of the imine to form intermediate **IV**. Oxidative cyclization in intermediate **IV** gives the π-allyliridium(III) complex **V**, and reductive elimination provides **VI**, which is followed by subsequent protonolysis to form the product and regenerate the cationic iridium catalyst **I**. This type of asymmetric [3 + 2] annulation has been further performed by the same group with alkynes to give products **294**(200) and with 1,3-enynes to provide compounds **295**.^201^

**Scheme 85:**
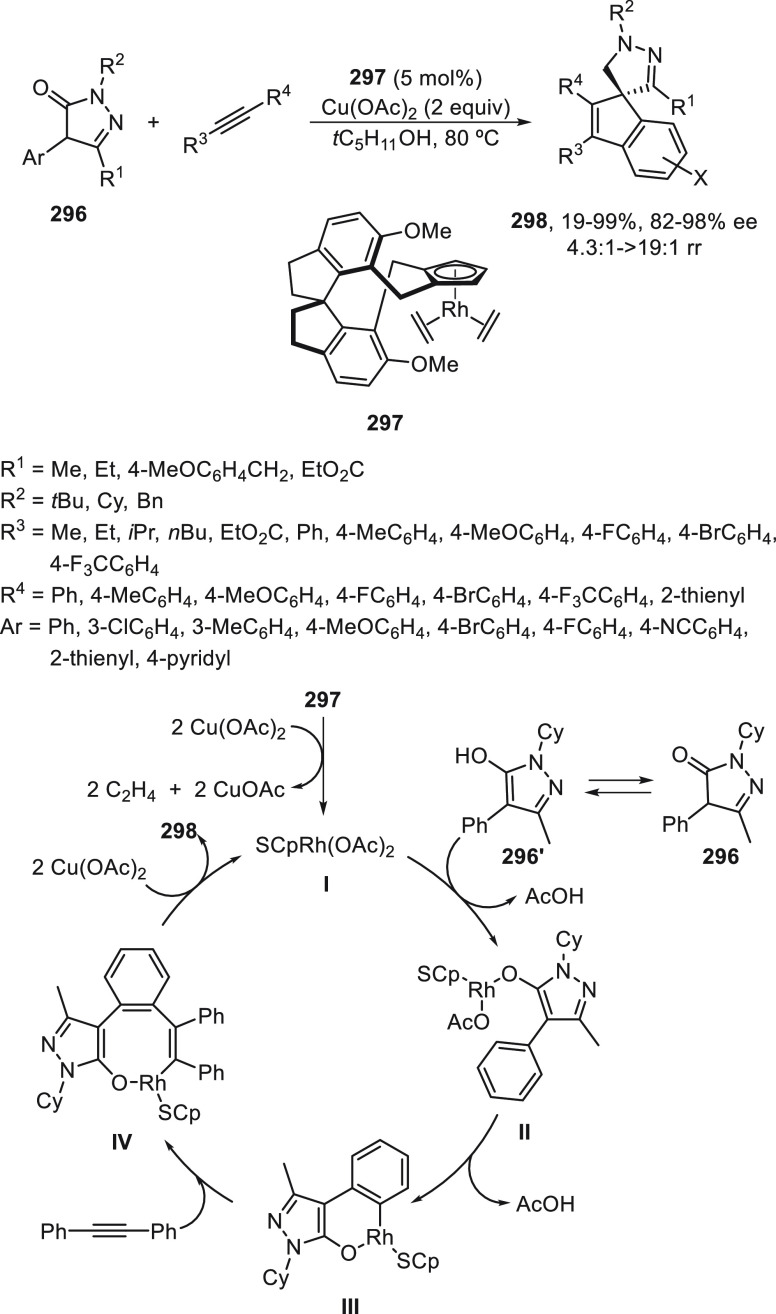
Enantioconvergent Rh-Catalyzed Annulation of 4-Aryl-5-pyrazolones **296** with Alkynes

You and co-workers^202^ have described a Rh-catalyzed C(sp^2^)–H functionalization reaction of 4-aryl-5-pyrazolones **296** followed by [3 + 2] annulation reactions with alkynes to furnish highly enantioenriched 4-spiro-5-pyrazolones **298** in up to 99% yield and 98% ee (Scheme 85). These processes were catalyzed by a Rh complex **297** bearing a chiral 1,1′-spirobiindane scaffold SCpRh(C_2_H_4_)_2_. In the proposed catalytic cycle, once the pyrazolone **296** (R^1^ = Me, R^2^ = Cy, Ar = Ph) tautomerizes into **296′**, the Rh catalyst **I** deprotonates the hydroxy group of **296′** to form intermediate **II**. This intermediate **II** undergoes C–H activation to give rhodacycle **III**, which forms the eight-membered rhodacycle **IV** by alkyne coordination and migratory insertion. Final reductive elimination provides product **298** and releases the Rh(I) species, which is oxidized by Cu(OAc)_2_ to the active Rh(III) catalyst **I**.

### 5.2 C(sp^3^)–H Functionalization

In this Section, enantioconvergent functionalization of racemic compounds at the C(sp^3^)–H located at the α-, β-, and γ-positions of the functional group will be considered.

#### 5.2.1 α-Functionalization

**Scheme 86:**
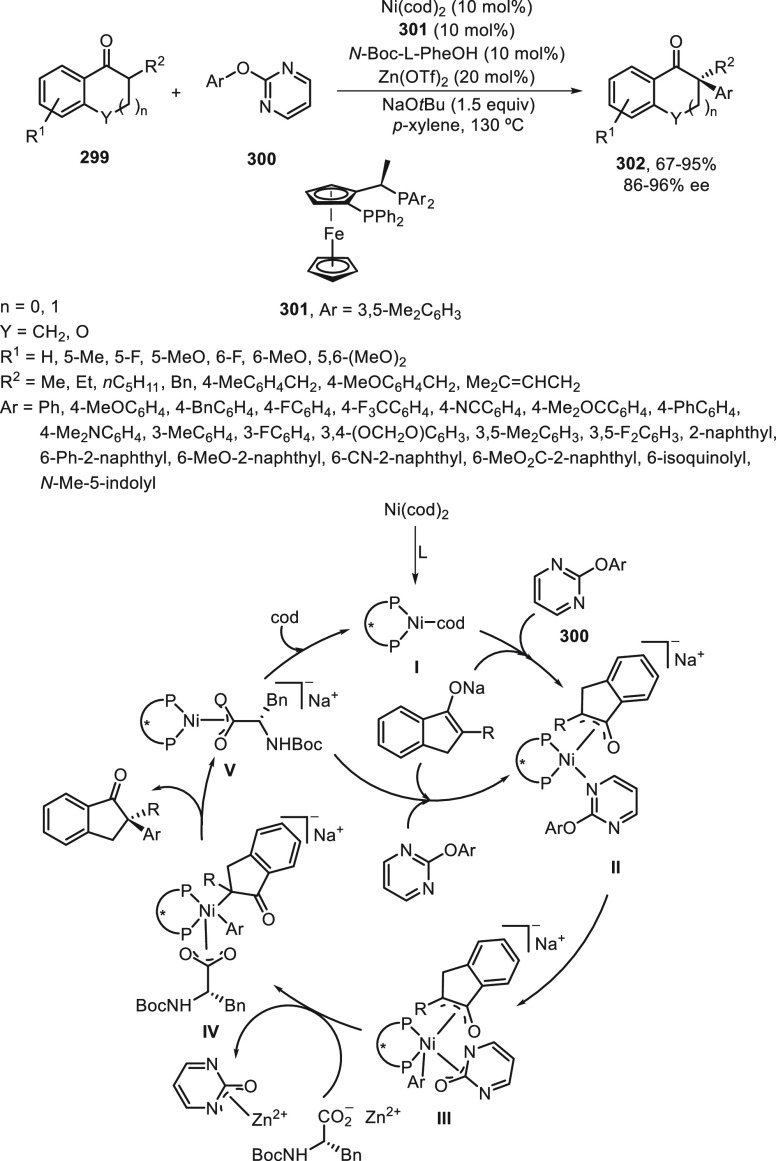
Enantioconvergent Ni-Catalyzed α-Arylation of Cyclic Ketones **299** with Aryl Pyrimidyl Ethers **300**

Racemic α-substituted carbonyl compounds and related systems are transformed into enantioenriched ketones with a quaternary carbon at the α-position, generally by enantioselective metal-catalyzed arylation reactions. This chemistry has been recently covered by Zhou, Yu, and co-workers.^203^ For the α-arylation of α-carbonyl enolates, aryl bromides, chlorides, and triflates have been used under Pd and chiral phosphines catalysis described by Buchwald and co-workers.^204^ In the pioneering work of Ma and co-workers,^205^ the arylation of 2-methylacetoacetates was performed with 2-iodotrifluoroacetanilides under CuI/*trans-*4-hydroxy-l-proline catalysis. The α-arylation of ketones with chloroarenes was carried out by Ge and Hartwig^206^ under Ni(cod)_2_/chiral diphosphines catalysis. Martin and co-workers^207^ described for the first time the asymmetric α-arylation of cyclic ketones with aryl pivaloyl esters under Ni(cod)_2_/tol-Binap catalysis. Recently, Li and Wang^208^ reported the same transformation using cyclic ketones **299** and aryl pyrimidyl ethers **300** under Ni(cod)_2_/Josiphos **301** catalysis (Scheme 86). The corresponding α-arylated ketones **302** were obtained with good yields and enantioselectivities in the presence of *N*-Boc-l-phenylalanine and Zn(OTf)_2_ in *p*-xylene at 130 °C. According to experimental studies, a plausible mechanism was proposed starting with formation of the chiral Ni complex **I**, which undergoes ligand exchange with the enolate anion of **299** and the aryl pyrimidyl ethers **300** to provide the anionic Ni(0) intermediate **II**. This intermediate **II** evolves to intermediate **III** by oxidative addition, which after ligand exchange with the chiral amino acid and Zn(OTf)_2_ provides intermediate **IV**. Subsequent reductive elimination of the Ni(II) complex **IV** gives the arylated ketone **302** and complex **V** to regenerate the catalyst by ligand exchange with 1,5-cyclooctadiene (cod) or with the enolate anion to form intermediates **I** and **II**, respectively.

Enantioconvergent α-arylation of amides to give enantioenriched oxindoles was first described by Lee and Hartwig^209^ using Pd(dba)_2_ and a chiral *N*-heterocyclic carbene (NHC) as catalyst. Later, Buchwald and co-workers^210^ developed enantioconvergent α-arylation of 3-alkyl oxindoles with aryl bromides under Pd/chiral biaryl monophosphine ligand catalysis. α-Substituted γ-butyrolactones were enantioconvergently α-arylated with aryl chlorides and bromides in 2002 by Spielvogel and Buchwald^211^ using Ni(cod)_2_/Binap as catalyst in the presence of ZnBr_2_. Stolz, Morgan, and co-workers^212^ described the enantioconvergent α-arylation of α-alkyl γ-lactams with aryl iodides and bromides using Pd(0)/chiral diphosphines as catalyst. The first Pd-catalyzed enantioconvergent α-arylation of alkylnitriles with aryl bromides to provide enantioenriched α-aryl-α-alkyl nitriles was reported in 2016 by Zhou and co-workers^213^ using PdCl_2_/chiral phosphoramidite as catalyst.

**Scheme 87:**
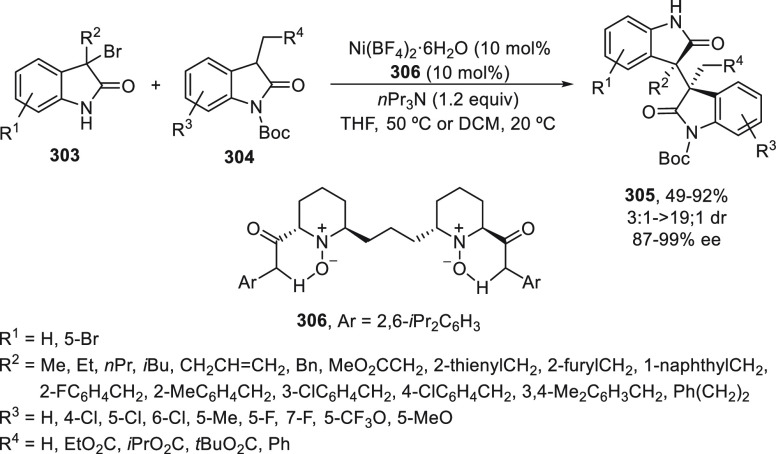
Enantioconvergent Ni-Catalyzed Reaction of 3-Bromooxindoles **303** with 3-Substituted *N*-Boc Oxindoles **304**

Feng and co-workers^214^ reported the asymmetric C(sp^3^)–C(sp^3^) cross-coupling of racemic 3-substituted *N*-Boc oxindoles **303** with racemic 3-bromo oxindoles **304** for the enantioconvergent synthesis of 3,3′-bisoxindoles **305** (Scheme 87). This reaction took place with high yields and good diastereo- and enantioselectivities under mild reaction conditions catalyzed by chiral Ni(BF_4_)_2_/*N,N′*-dioxide **306** complex as a chiral Lewis acid. The corresponding products **305** were transformed into diverse hexahydropyrroloindole alkaloids, such as (+)-chimonanthidine, (+)-calycanthidine, and related compounds, with potential antiparasitic and anticancer properties.

**Scheme 88:**
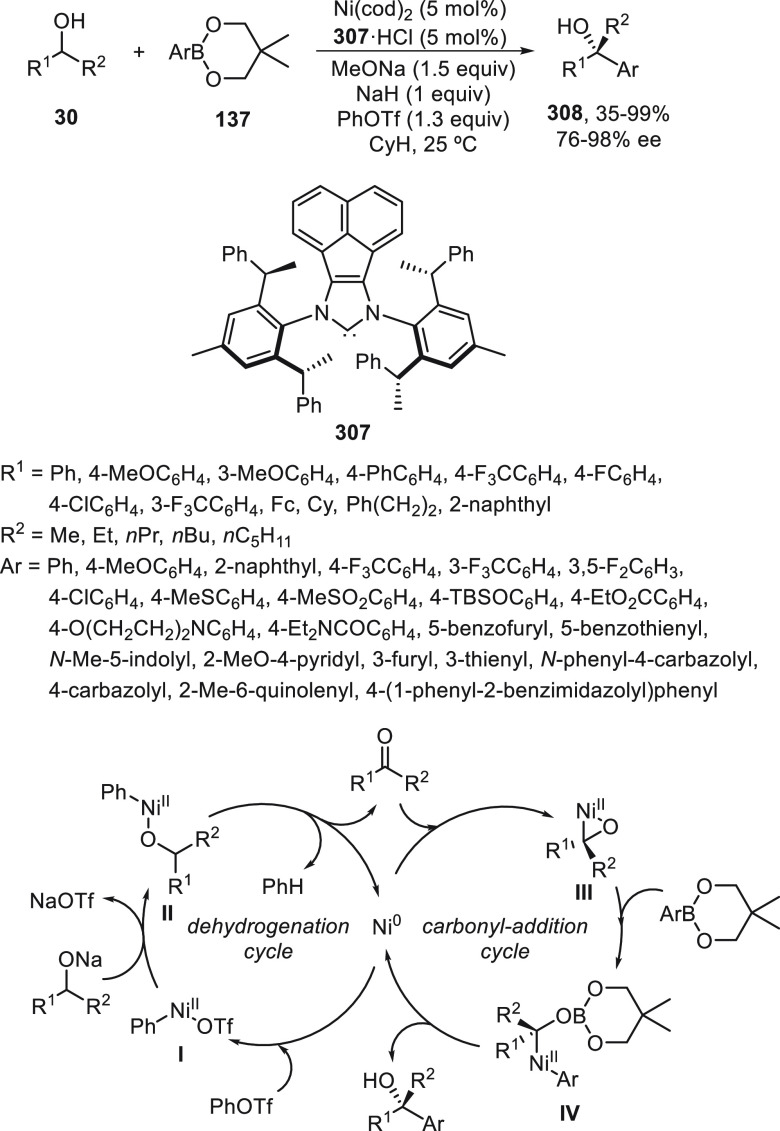
Enantioconvergent Ni-Catalyzed α-Arylation of Secondary Benzylic Alcohols **30** with Arylboronates **137**

Recently, Cai and Shi^215^ reported an enantioconvergent formal α-arylation of racemic secondary benzylic alcohols **30** to enantioenriched tertiary alcohols **308** by means of a Ni/NHC **307** (ANIPE) catalyst (Scheme 88). This transformation takes place via a dehydrogenation of the secondary alcohol by phenyl triflate followed by addition of arylboronic esters **137** to the intermediate ketones. In the proposed dehydrogenative cycle, phenyl triflate undergoes oxidative addition to Ni(0) to give complex **I**, which reacts with the secondary alcohol to provide intermediate **II**. Subsequent β-hydride elimination of **II** gives benzene and the ketone regenerating Ni(0). In the carbonyl addition cycle, the ketone experiments a Ni-catalyzed enantioselective coupling by oxidative cyclization to form intermediate **III** followed by transmetalation with the arylboronic ester to provide intermediate **IV**. Final reductive elimination of complex **IV** forms the chiral tertiary alcohol and regenerates the Ni(0) catalyst.

#### 5.2.2 β-Functionalization

**Scheme 89:**
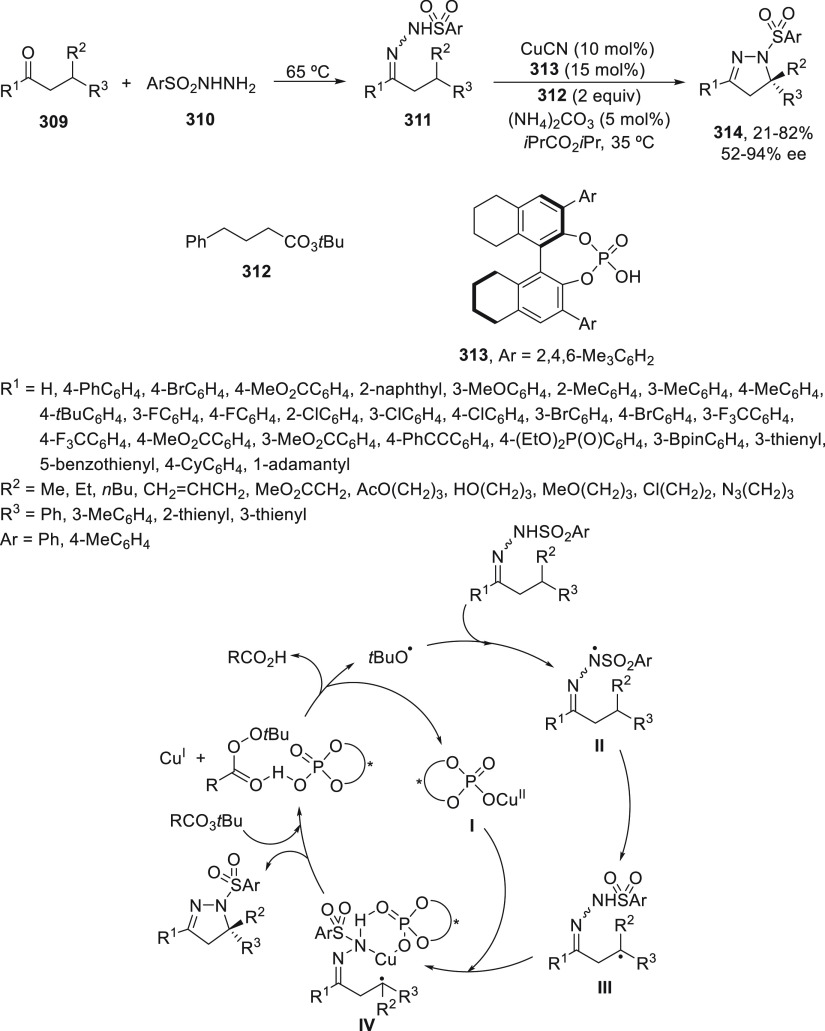
Enantioconvergent Cu/CPA-Catalyzed β-Amination of Hydrazones **311** Derived from Ketones **309**

Recently, metal-catalyzed processes have been used for enantioselective transformations by functionalization of prochiral β-C(sp^3^)–H bonds.^216^ Liu and co-workers^217^ have reported a radical enantioconvergent Cu(I)/chiral phosphoric acid (CPA) **313** dual catalytic protocol for the amination of racemic ketones bearing tertiary C(sp^3^)–H bonds **309** at the β-position (Scheme 89). By reaction of ketones **309** with arylsulfonylhydrazides **310**, the corresponding hydrazones **311** were formed, which were treated with CuCN/CPA **313**, perester **312** as oxidant, and ammonium carbonate as additive to provide enantioenriched dihydropyrazoles **314** with moderate to good yields and enantioselectivities. Mechanistic investigations suggest that initially Cu(I) reacts with **312**-activated peroxide via a SET process to afford a *tert-*butoxy radical and the chiral Cu(II) phosphate complex **I**. Intermolecular hydrogen abstraction of the NH bond in the hydrazone by the *t*BuO radical provides radical **II**. A subsequent intramolecular 1,5-hydrogen atom abstraction step forms the tertiary radical **III**, which associates with complex **I** to form intermediate **IV** and promote the enantioselective C–N bond formation.

#### 5.2.3 γ-Functionalization

**Scheme 90:**
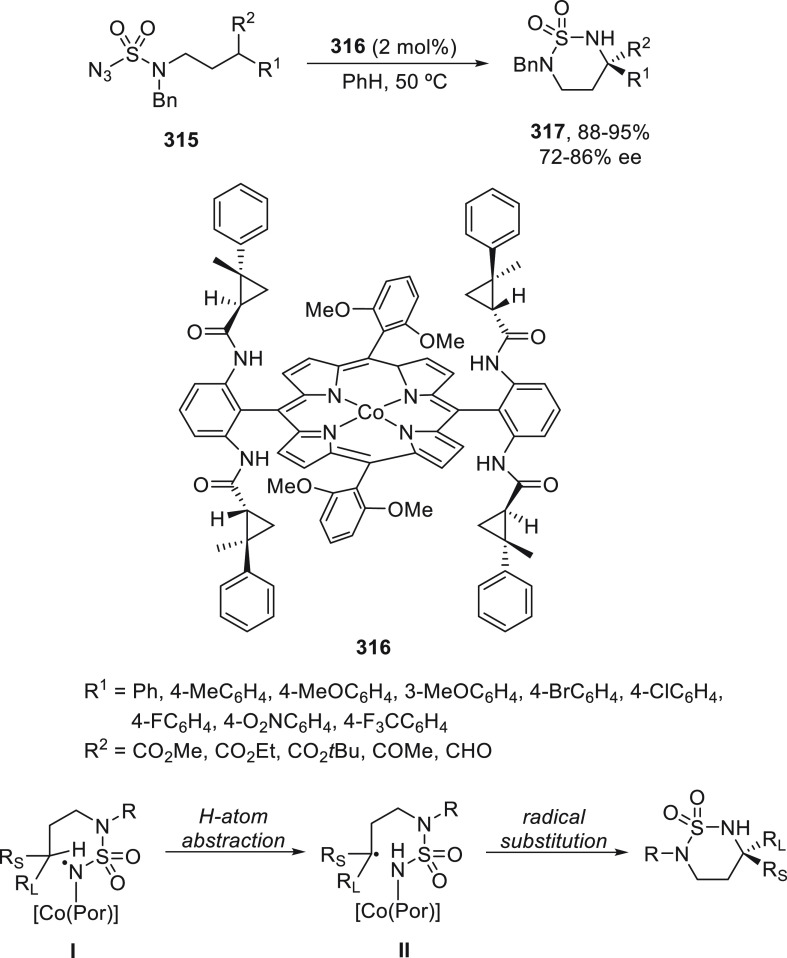
Enantioconvergent Co-Catalyzed γ-Amination of Sulfamoyl Azides **315**

Enantioconvergent amination of racemic tertiary C(sp^3^)–H bonds has been achieved by Zhang and co-workers^218^ through an intramolecular radical process via Co(II)-based metalloradical catalysis. This enantioconvergent 1,6-C(sp^3^)–H amination of sulfamoyl azides **315** was carried out using a Co(II) complex of porphyrin 2,6-DiMeO-QuingPhyrin **316** in benzene at 50 °C to form six-membered cyclic sulfamides **317** in up to 95% yield and 86% ee (Scheme 90). In the proposed mechanism, the efficient H atom abstraction of the tertiary C–H bond occurs by formation of α-Co(III)-aminyl radical **I**, which gives the carbon-centered radical **II**. Subsequent radical substitution of intermediate **II** provides the cyclic product **317**. These products have been applied to the stereoselective construction of bicyclic N-heterocycles as sulfamide-fused piperazinone, imidazolone, and tetrahydroquinazoline.

### 5.3 Allylic Functionalizations

**Scheme 91:**
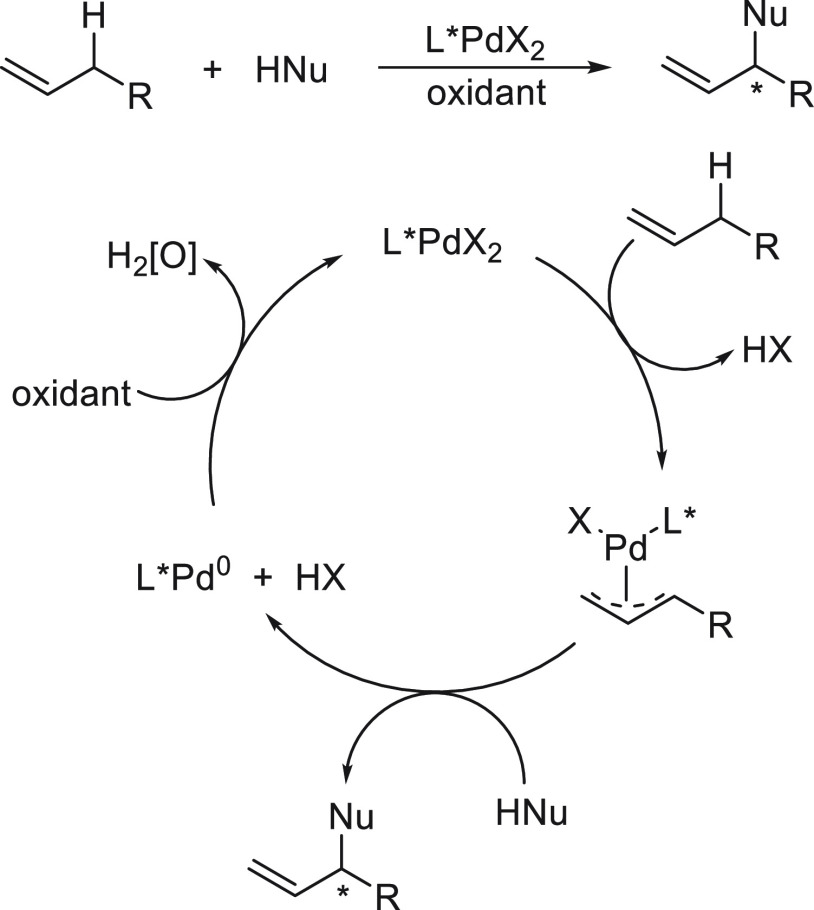
Pd-Catalyzed Allylic C–H Functionalization

Asymmetric allylic C–H functionalization under Pd catalysis with unfunctionalized alkenes using an allylic hydrogen atom as the leaving group needs stoichiometric amounts of an oxidant.^219^ The cleavage of the allylic C–H bond gives the corresponding π-allylpalladium intermediate, which by nucleophilic attack accelerated by a phosphorus-based ligand releases Pd(0) and the product. By means of a stoichiometric amount of an oxidant, the Pd(II) catalyst is regenerated (Scheme 91).

**Figure 4:**
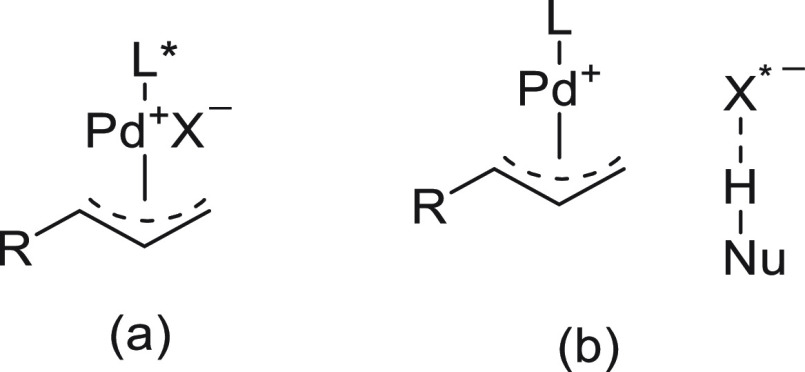
Asymmetric strategies in the Pd-catalyzed allylic C–H functionalization.

For the stereoselectivity control, two main strategies have been employed: (a) the use of a chiral ligand compatible with the oxidant and (b) a chiral counterion able to form hydrogen-bonding interactions with the nucleophile and also with a chiral ligand (Figure 4).

**Scheme 92:**
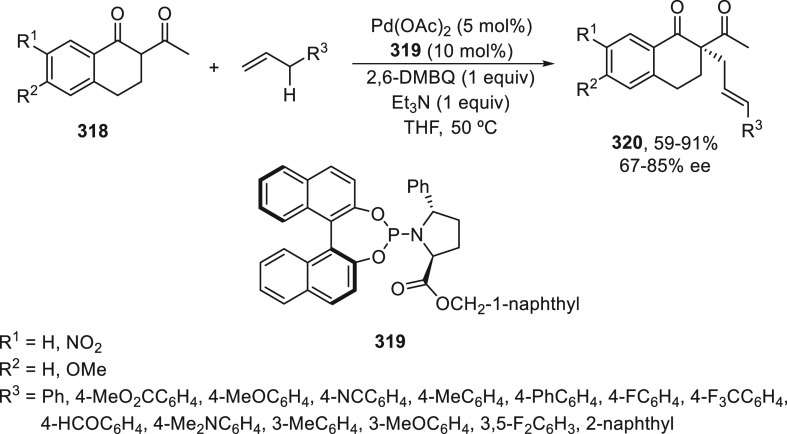
Enantioconvergent Pd-Catalyzed Allylic Alkylation of 1,3-Diketones **318** with Allylbenzenes

These processes can be enantioconvergent when the nucleophile attacking the cationic π-allylpalladium intermediate is an enolate derived from a racemic compound. The first example was described by Trost and co-workers^220,221^ using 1,3-diketones **318** as nucleophiles, allylbenzenes, phosphoramidite **319** as chiral ligand, 2,6-dimethylbenzoquinone (2,6-DMBQ) as oxidant, and Et_3_N as base (Scheme 92), The resulting allylated 1,3-diketones **320** were obtained in up to 91% yield and 85% ee, which is lower than the traditional asymmetric allylic alkylation (AAA) with allylic acetates.

**Scheme 93:**
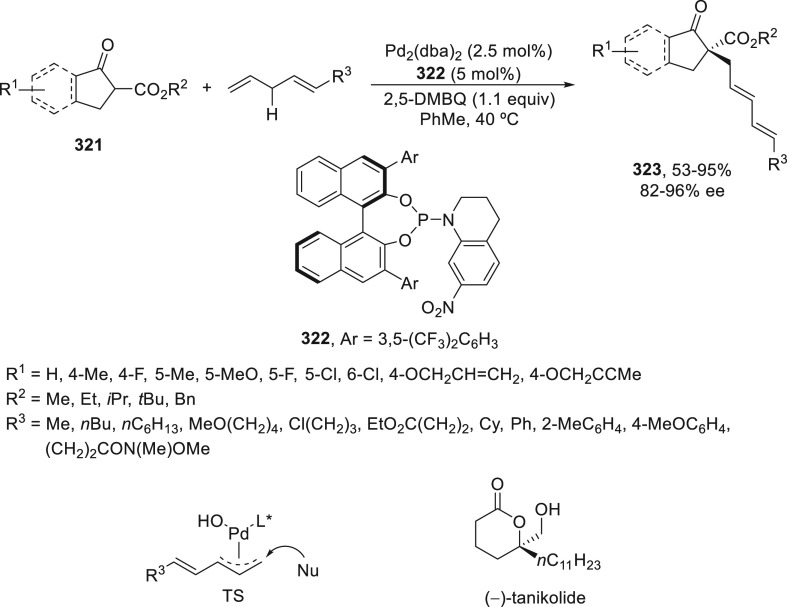
Enantioconvergent Pd-Catalyzed Allylic Alkylation of 1,4-Dienes with Cyclic β-Keto Esters **321**

Gong and co-workers^219,222^ reported the allylation of cyclic β-keto esters **321** with 1,4-dienes in the presence of phosphoramidite **322** to provide enantioenriched α,α-disubstituted β-keto esters **323** with up to 96% ee (Scheme 93). The authors proposed a linear outer-sphere TS to explain the regioselective formation of the linear dienyl products **323**. This procedure was applied to the formal synthesis of tanikolide, a brine-shrimp toxin and antifungal marine natural product isolated from the lipid extract of the cyanobacterium.^223^

**Scheme 94:**
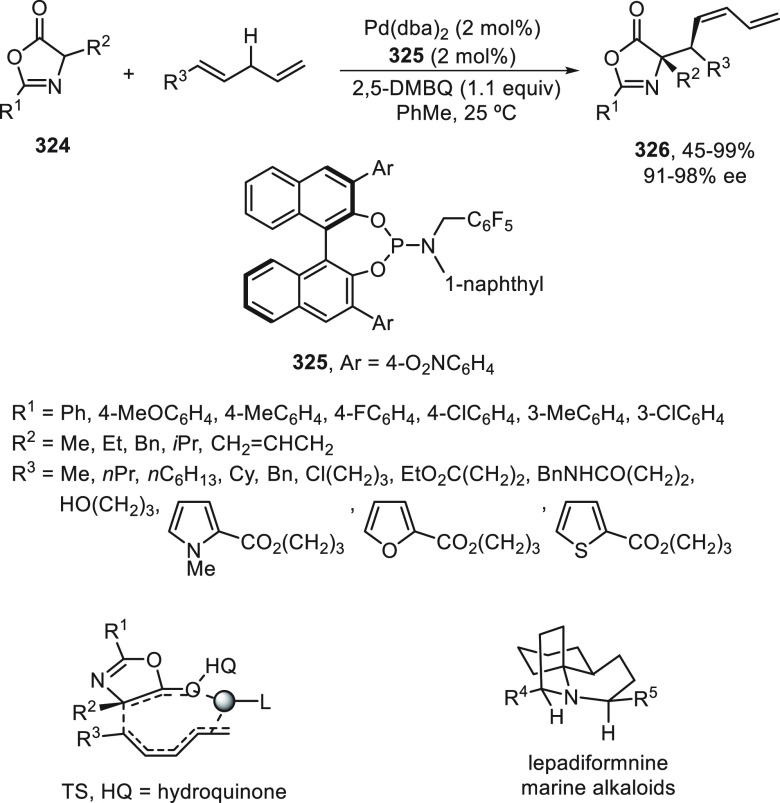
Enantioconvergent Pd-Catalyzed Allylic Alkylation of Azlactones **324** with 1,4-Dienes

However, the use of azlactones **324** as nucleophiles resulted in the corresponding branched products **326** (Scheme 94).^219,224^ Under Pd(dba)_2_ and phosphoramidite **325** catalysis with the use of 2,5-DMBQ as external oxidant, the allylic C–H alkylation of 1,4-dienes gave α,α-disubstituted α-amino acid surrogates **326** with high yields and excellent levels of *Z* diastereoselectivities and enantioselectivities. Experimental and computational studies suggest that through the TS the stereo and regioselectivity are governed by the geometry and coordination pattern of the nucleophile. This methodology has been applied to the synthesis of a key intermediate for the synthesis of lepadiformine marine alkaloids described by Rychnovsky and co-workers.^225^

**Scheme 95:**
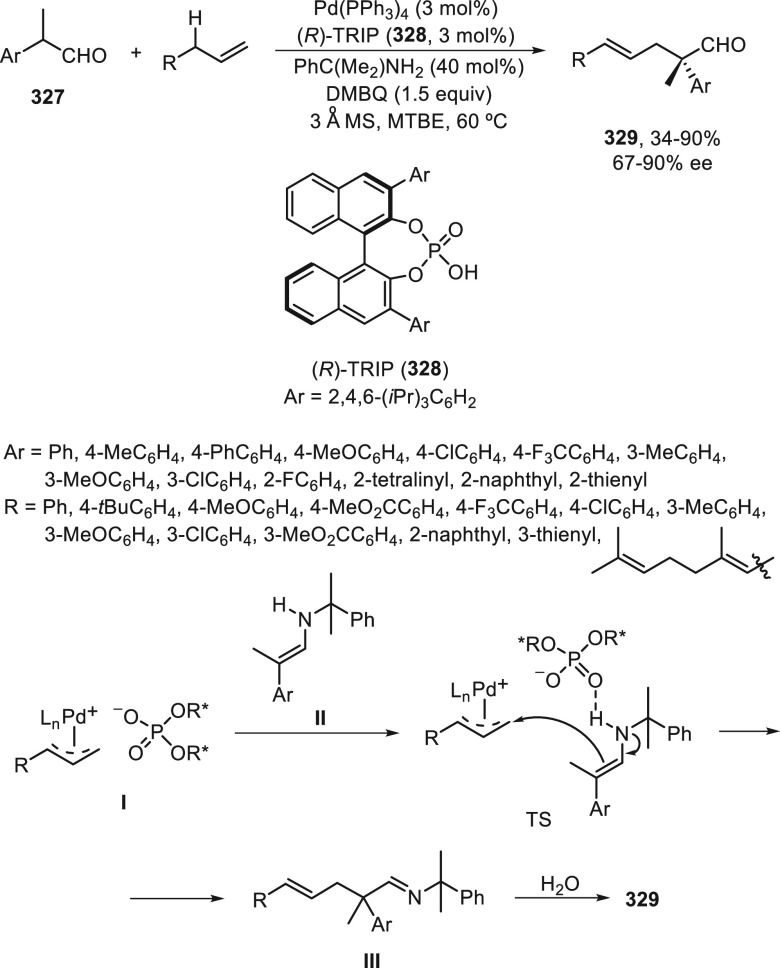
Enantioconvergent Pd- and CPA-Catalyzed Allylic Alkylation of Aldehydes **327** with Terminal Alkenes

Enantioconvergent α-allylation of racemic enolizable aldehydes **327** with terminal alkenes was reported by Gong and co-workers^219,226^ by using the chiral counteranion strategy. This asymmetric cooperative catalysis was carried out under Pd(PPh_3_)_4_, cumylamine, and a CPA (*R*)-TRIP (**328**) catalysis with 2,5-BMBQ as oxidant and 3 Å molecular sieve (MS) as additive in methyl *tert-*butyl ether (MTBE) at 60 °C (Scheme 95). The resulting allylated aldehydes **329** were obtained with good yields and enantioselectivities. In the proposed mechanism, a π-allylpalladium phosphate complex **I** reacts with enamine **II** via TS, as demonstrated by Mukherjee and List,^227^ to give imine **III**, which generates the aldehyde **329** by hydrolysis. This process was also performed with 1,4-dienes under similar reaction conditions to provide the linear (*E,E*)-dienyl aldehydes in up to 82% yield, up to 94% ee, and >20:1 *E/Z* stereoselectivity.^219,228^

**Scheme 96:**
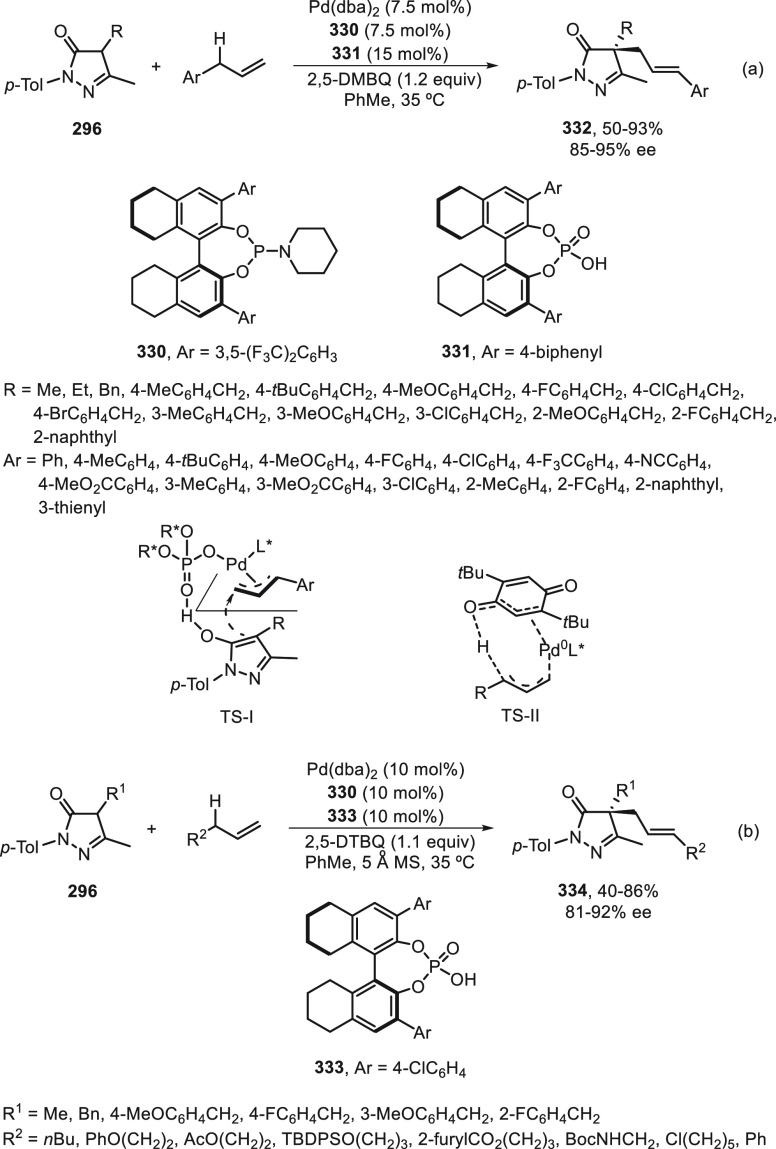
Enantioconvergent Pd- and CPA-Catalyzed Allylic Alkylation of Pyrazol-5-ones **296** with Terminal Alkenes

Pyrazol-5-ones **296** are considered soft nucleophiles and have been allylated with allyl arenes by the cooperative catalysis of a chiral palladium complex and a chiral Brønsted acid by Gong and co-workers.^219,229^ This allylic C–H alkylation was carried out with Pd(dba)_2_/phosphoramidite **330** and CPA **331** as catalysts and 2,5-DMBQ as oxidant in toluene at 35 °C to furnish compounds **332** with good yields and enantioselectivities (Scheme 96a). In the proposed catalytic cycle, the π-allylpalladium complex reacts with the tautomer of pyrazole-5-one to form the corresponding TS-I, in which the CPA is bonded to the OH group and also to the Pd. When unactivated terminal alkenes were used, another CPA **333** and the bulkier oxidant 2,5-di-*tert*-butylbenzoquinone (2,5-DTBQ) were used to provide the corresponding allylated pyrazole-5-ones **334** with good yields and enantioselectivities (Scheme 96b).^218,230^ For this alkylation with inert allylic C–H bonds, TS-II has been proposed in which a concerted proton and a two-electron transfer process facilitates the allylic C–H cleavage.^219,224^

**Scheme 97:**
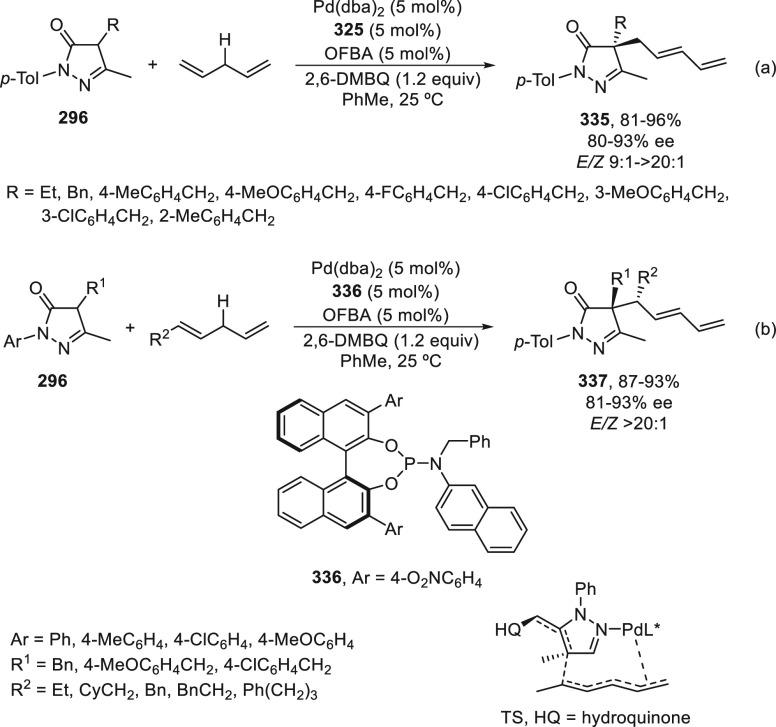
Enantioconvergent Pd- and Achiral Phosphoric Acid-Catalyzed Allylic Alkylation of Pyrazol-5-ones **296** with 1,4-Dienes

For the enantioselective allylic C–H alkylation of 1,4-pentadienes with pyrazole-5-ones **296**, Pd(dba)_2_/phosphoramidite **325** and achiral 2-fluorobenzoic acid (OFBA) as cocatalyst gave the corresponding linear products **335** with high yields and enantioselectivities. With regard to diastereoselectivity, the *E/Z* ratio was 9:1 to >20:1 (Scheme 97a).^219,229^ However, substituted 1,4-pentadienes, phosphoramidite **336**, and OFBA gave the best results by affording C5-branched and *E*-dienyl products **337** with up to 93% yield and up to 93% ee (Scheme 97b).^219,229^ According to DFT calculations, the TS explains the attack of pyrazole-5-one preferentially at the vinyl position via an inner-sphere mechanism in which the nucleophile prefers nitrogen coordination with Pd to afford the C5-branched regioselectively.

**Scheme 98:**
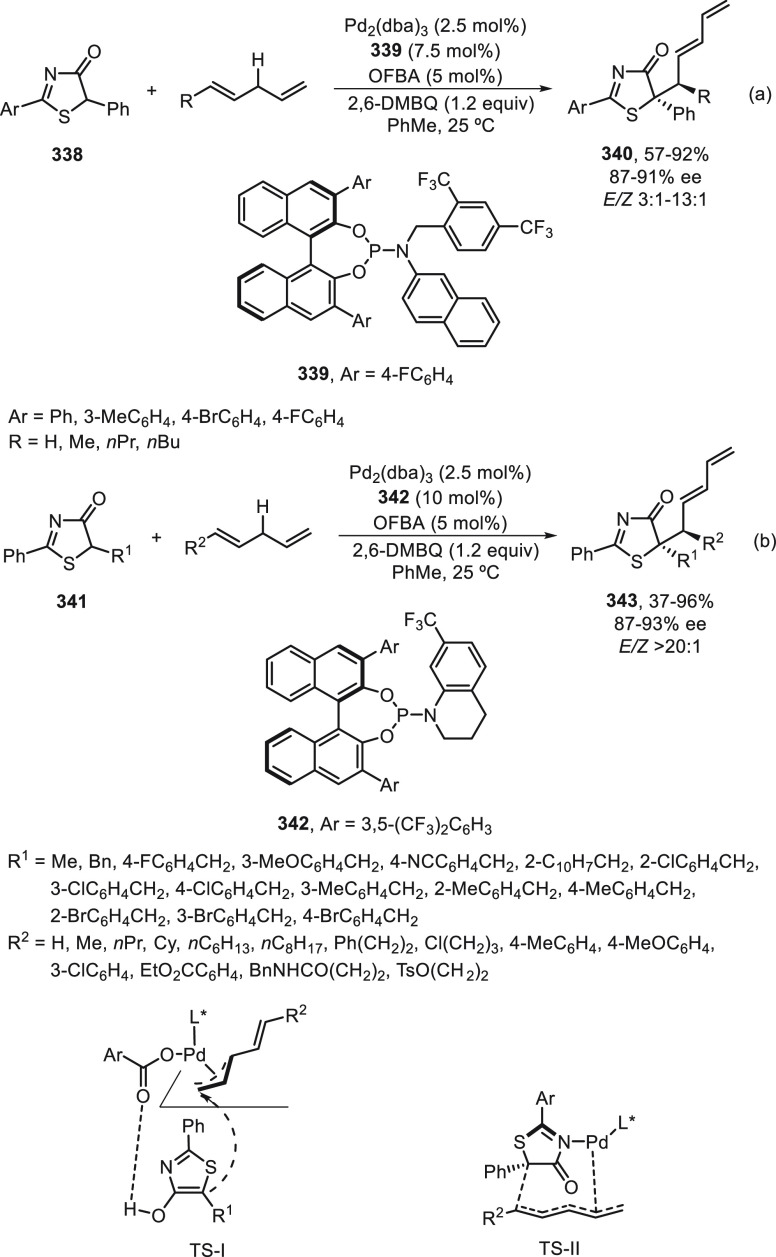
Enantioconvergent Pd- and Achiral Phosphoric Acid-Catalyzed Allylic Alkylation of 5*H*-Thiazol-4-ones **338** and **341** with 1,4-Dienes

The C5-branched regioselectivity was also observed in the case of 2,5-diarylthiazol-4(5*H*)-ones **338** using as chiral phosphoramidite **339** and the achiral phosphoric acid OFBA (Scheme 98a).^219,231^ Under this cooperative catalysis, a broad range of α,α-disubstituted 5*H*-thiazol-4-ones **340** were isolated in up to 92% yield and up to 91% ee. However, when 5-alkylthiazol-4(5*H*)-ones **341** were treated with 1,4-dienes using phosphoramidite **342** as chiral ligand and OFBA as Brønsted acid, linear products **343** were mainly formed in up to 96% yield and up to 93% ee (Scheme 98b). This nucleophile-dependent regioselectivity was explained by the difference in the acidity and also by the steric hindrance of the 5-substituted thiazolones. In the case of 5-alkylthiazolones, which have lower acidity and bulkiness than 5-arylthiazolones, the attack on the vinyl π-allylPd intermediate occurs through an outer-sphere mechanism (TS-I) where hydrogen bonding interactions between the counteranion and thiazolone help to form the linear products. For the formation of the C5-branched product, an inner-sphere mechanism (TS-II) was proposed.

Copper-catalyzed enantioconvergent cross-coupling of azoles with benzylic bromides through C(sp^2^)–H functionalization takes place under alkyl radical formation. For enantioconvergent annulation reactions of 3-hydroxyindolinones with dienes and pyrazolones with alkynes, Ir and Rh catalysts are used, respectively. With respect to the C(sp^3^)–H functionalization of the α-position of carbonyl compounds, Pd, Cu, and Ni catalysts allow their enantioconvergent arylation. In the case of benzylic alcohols under Ni catalysis, the enantioconvergent α-arylation can be carried out with acylboronates. For the enantioconvergent intramolecular β- and γ-amination of hydrazones and sulfamoyl azides, Cu and Co catalysts have been employed, respectively. The allylic C–H enantioconvergent functionalization of terminal alkenes and 1,4-dienes is performed under Pd catalysis and stoichiometric amounts of an oxidant, generally benzoquinones using chiral phosphoric acids. 1,3-Diketones, azlactones, aldehydes via enamines, pyrazolones, and thiazolones have been used as nucleophiles.

## 6 Enantioconvergent Hydrofunctionalization of Unsaturated Hydrocarbons

In this Section, an alternative to metal-catalyzed enantioconvergent alkyl–alkyl cross-coupling will be considered on the basis of reductive hydroalkylation of racemic alkyl halides with unsaturated hydrocarbons, such as olefins, allenes, and alkynes.

**Scheme 99:**
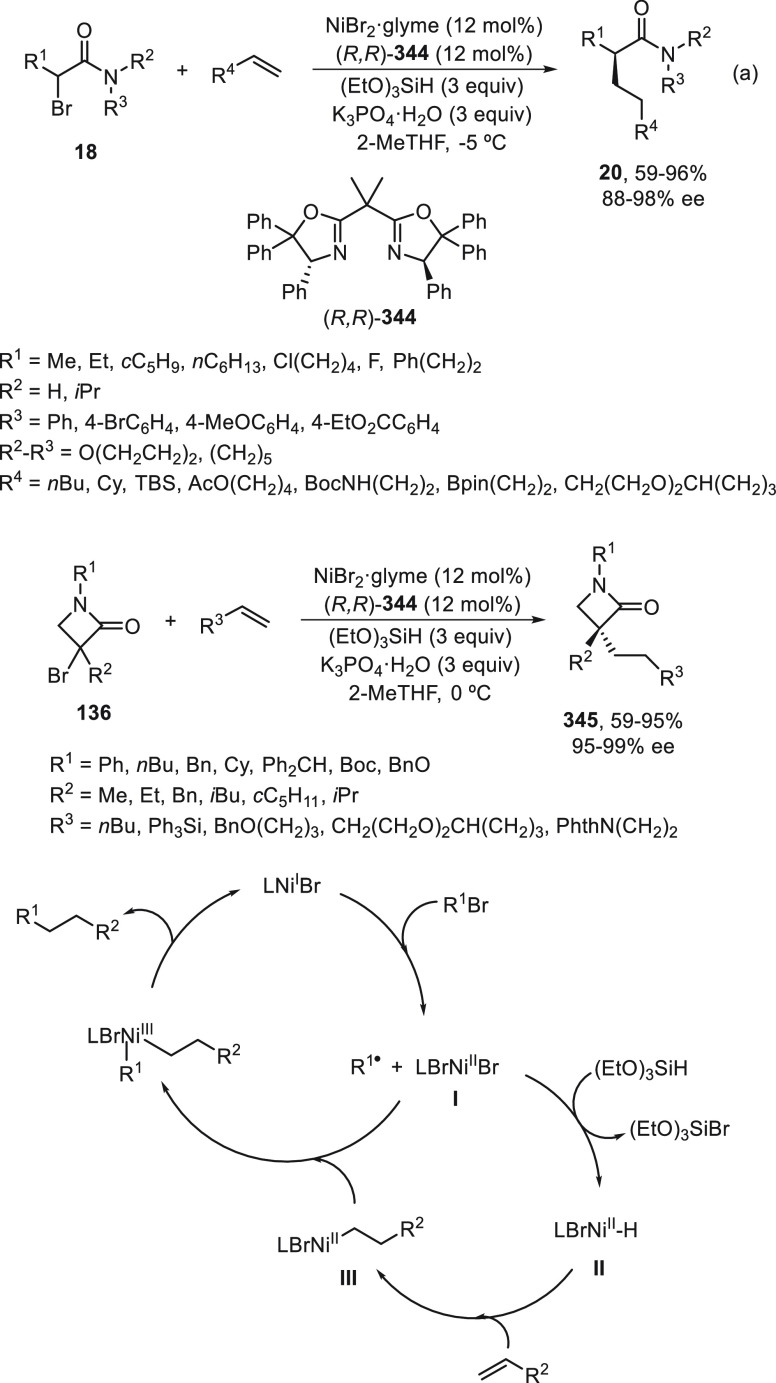
Enantioconvergent Ni-Catalyzed Hydroalkylation of Olefins with α-Bromo Amides **18** and **136**

Fu and co-workers^232^ recently reported the enantioconvergent hydroalkylation of olefins with α-bromo amides. Racemic secondary amides **18** reacted with terminal olefins in combination with triethoxysilane under NiBr_2_/bis(oxazoline) (*R,R*)-**344** catalysis to provide the alkylated products **20** with good yields and enantioselectivities (Scheme 99a). In a representative example using (*Z*)-2-hexene as internal alkene, *n*-alkylation occurred by chain walking. In the case of tertiary bromides derived from β-lactams **136**, the corresponding products **345** bearing a quaternary stereocenter were generated with high yields and enantioselectivities under similar mild reaction conditions (Scheme 99b). The starting possible pathway is the Ni-catalyzed hydrosilylation of the olefin,^233^ and the resulting alkylsilane can serve as nucleophile in a Hiyama-type cross-coupling. However, this possibility was discarded because under these reaction conditions an alkylsilane was not cross-coupled with the alkyl bromide. In the proposed mechanism, the Ni(II) complex **I** reacts with trimethoxysilane to give a Ni–hydride complex **II**. After alkene complexation followed by migratory insertion, intermediate **III** results, which enters in the reaction cycle to undergo cross-coupling with the alkyl bromide. Other families of electrophiles, such as α-bromo esters, underwent very efficient cross-coupling with 1-hexene.

**Scheme 100:**
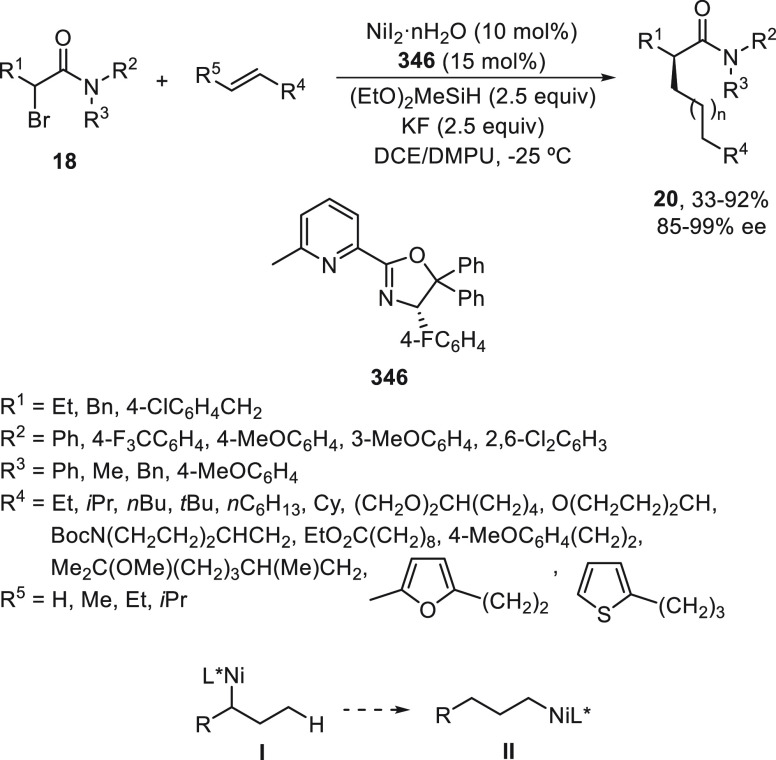
Enantioconvergent Ni-Catalyzed Hydroalkylation of Alkenes with α-Bromo Amides **18**

Independently, Zhu and co-workers^234^ reported a similar enantioconvergent hydroalkylation of internal alkenes with racemic α-bromo amides **18** under NiH/oxazoline **346** (Scheme 100). This reaction took place by alkene isomerization followed by the hydroalkylation process to provide products **20** in up to 92% yield and up to 99% ee. By using mixtures of octenes, only a single isomer was obtained with 91% yield and 97% ee. Several terminal alkenes have been used to give products **20** with excellent ee. In the proposed mechanism, after NiH addition to provide complex **I**, a chain-walking strategy gives the terminal alkylnickel complex **II**, which is followed by enantioconvergent oxidative addition of the secondary alkyl bromide.

**Scheme 101:**
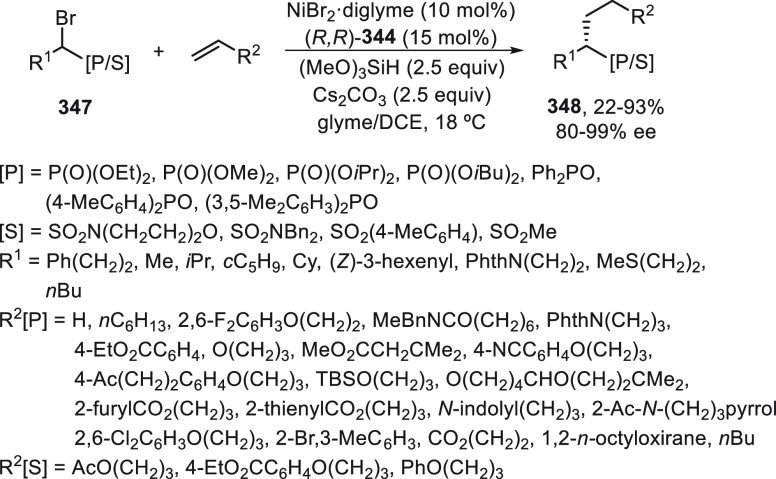
Enantioconvergent Ni-Catalyzed Hydroalkylation of Olefins with α-Heteroatom Phosphorus and Sulfur Alkyl Bromides **347**

When racemic α-heteroatom phosphorus or sulfur alkyl bromides **347** are used as electrophiles, and NiBr_2_/bis(oxazoline) (*R,R*)-**344** is used as catalyst, the hydroalkylation of terminal olefins takes place with good chemo-, regio-, and enantioselectivity to provide product **348** (Scheme 101).^235^ This alkyl–alkyl bond formation process was carried out under mild reaction conditions, a broad substrate scope, and good functional group compatibility. A radical-type enantioconvergent reaction mechanism has been proposed according to Fu’s proposal.^232^

**Scheme 102:**
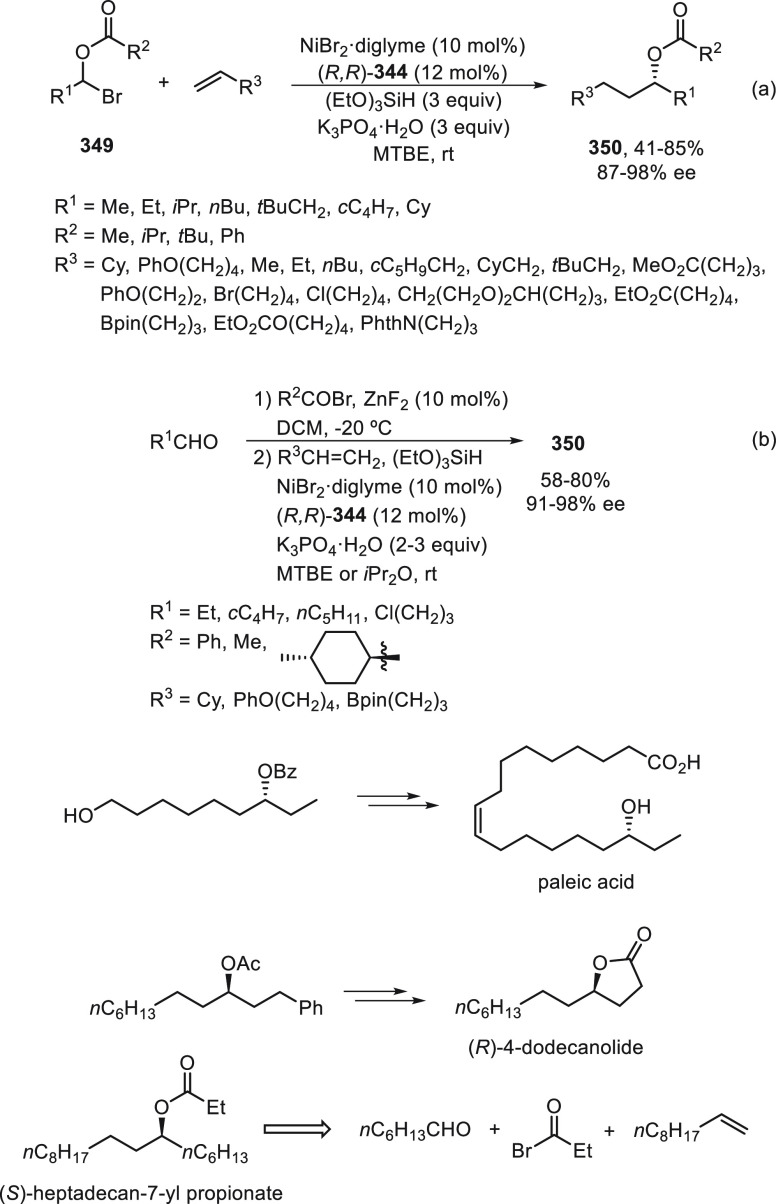
Enantioconvergent Ni-Catalyzed Hydroalkylation of Alkenes with α-Bromo *O*-Acyl Alcohols **349**

Enantioconvergent hydroalkylation of terminal alkenes with α-acyloxyalkyl bromides **349** was applied to the synthesis of enantioenriched *O*-arylated alcohols **350** by Yang and Fu^236^ (Scheme 102a). In this case, NiBr_2_/bis(oxazolidine) (*R,R*)-**344** was used as catalyst at room temperature to provide products **350** in up to 85% yield and up to 98% ee (Scheme 102b). This process can be also performed by generating *in situ* bromides **349** from aldehydes and acyl bromides and has been applied to the synthesis of key precursors of paleic acid, an antimicrobial; (*R*)-4-dodecanolide; and (*S*)-heptadecan-7-yl propionate, a component of a sex pheromone for the lichen moth.

**Scheme 103:**
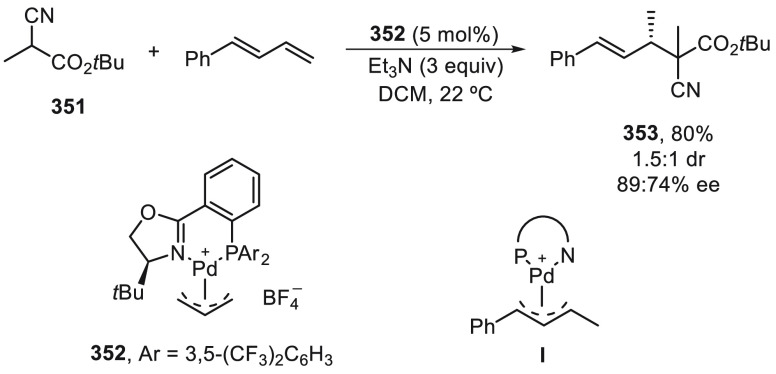
Enantioconvergent Pd-Catalyzed Hydroalkylation of (*E)*-1-Phenyl-1,3-butadiene with *t*-Butyl α-Cyanopropionate **351**

Malcolmson and co-workers^237^ reported the enantioselective Pd-catalyzed hydroalkylation of 1,3-dienes with racemic-activated C-nucleophiles, mainly β-diketones and malononitriles. In one particular example, this process is an enantioconvergent transformation, e.g., in the case of *t*-butyl α-cyanopropionate **351**. The reaction of this racemic nucleophile with (*E*)-1-phenyl-1,3-butadiene was carried out using chiral Pd-Phox complex **352** as catalyst and Et_3_N as base in CH_2_Cl_2_ at 22 °C to furnish product **353** in moderate diastereoselectivity (Scheme 103). This reaction occurs by nucleophilic attack of the corresponding enolate to the 1,3-disubstituted π-allyl intermediate **I**.

**Scheme 104:**
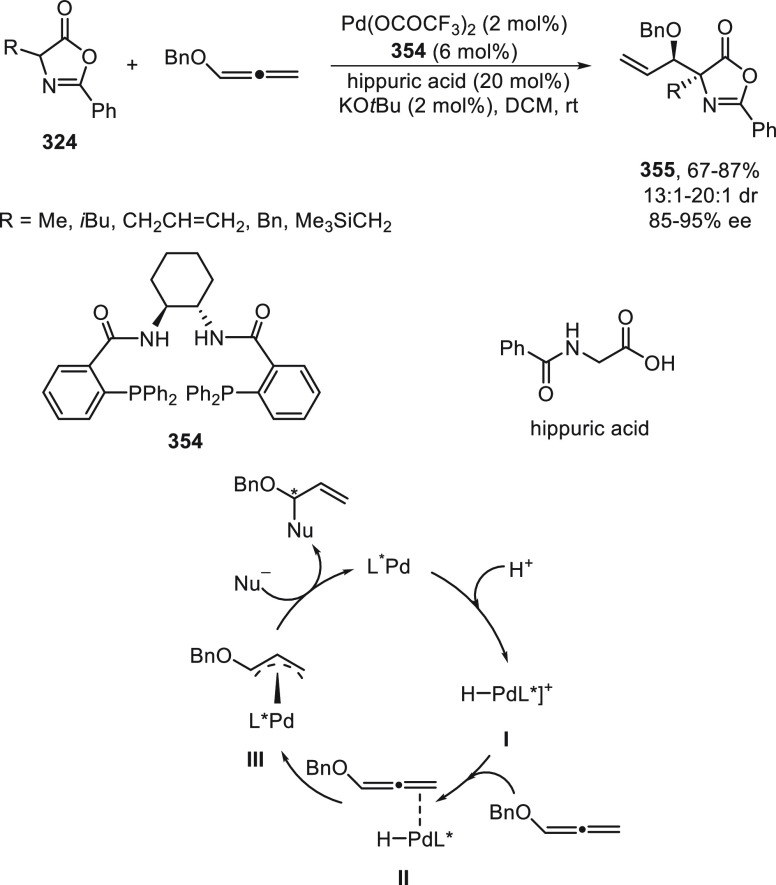
Enantioconvergent Pd-Catalyzed Hydroalkylation of 1-Benzyloxyallenes with Azlactones **324**

Trost and co-workers^238^ reported in 2003 the asymmetric addition of carbon nucleophiles to 1-benzyloxyallenes. Racemic azlactones **324** reacted enantioconvergently with 1-benzyloxyallenes under Pd/ligand **354** as catalyst by using hippuric acid and KO*t*Bu as buffered conditions to form products **355** with good yields and diastereo- and enantioselectivities (Scheme 104). In the proposed catalytic cycle, the catalyst reacts with NuH to give the hydride complex **I**, which coordinates the allene to give intermediate **II**. Subsequent formation of the cationic π-allylpalladium complex **III** followed by nucleophilic attack provides the product.

**Scheme 105:**
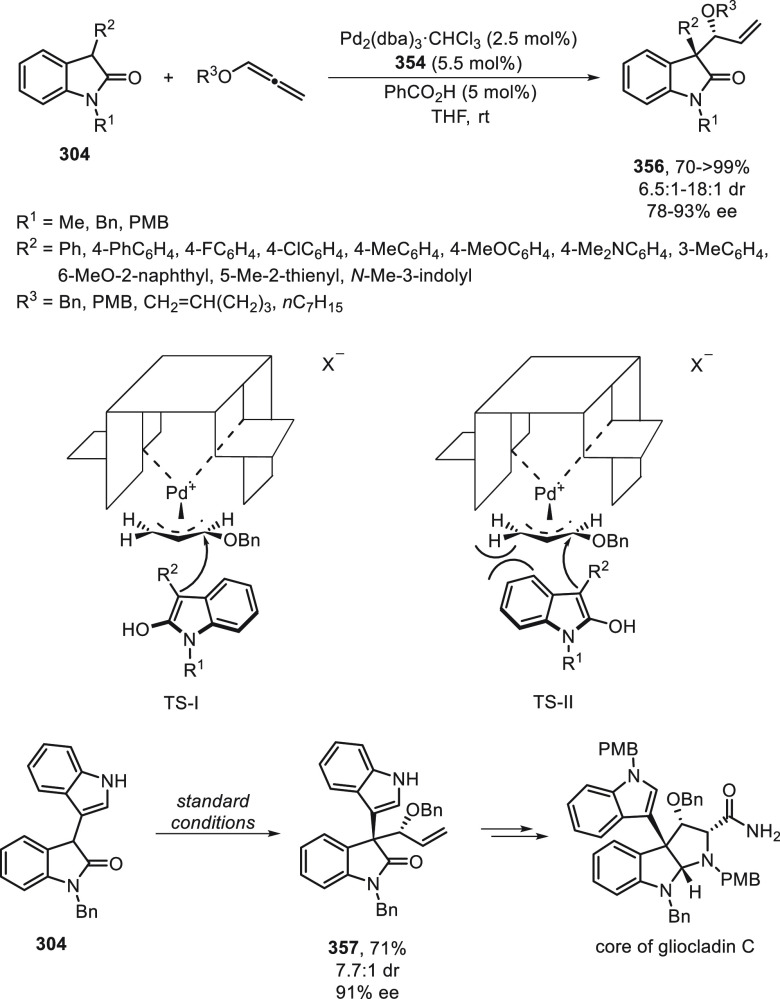
Enantioconvergent Pd-Catalyzed Hydroalkylation of 1-Alkoxyallenes with 3-Substituted Oxindoles **304**

The same group^239^ reported the enantioconvergent hydroalkylation of 1-alkoxyallenes with 3-substituted oxindoles **304** under Pd/**354** catalysis. In the presence of benzoic acid as cocatalyst, the corresponding enantioenriched oxindoles **356** with two vicinal stereocenters were obtained in excellent chemo-, regio-, diastereo-, and enantioselectivities with high chemical yields (Scheme 105). The authors proposed TS-I as the most favorable mechanism to explain the formation of (*R,R*)-**356** as the major stereoisomer toward TS-II. The 3-indolyl-substituted oxindole **304** was transformed into **357**, which was further transformed into the pyrrolidinoindoline core of the gliocladin natural products.

**Scheme 106:**
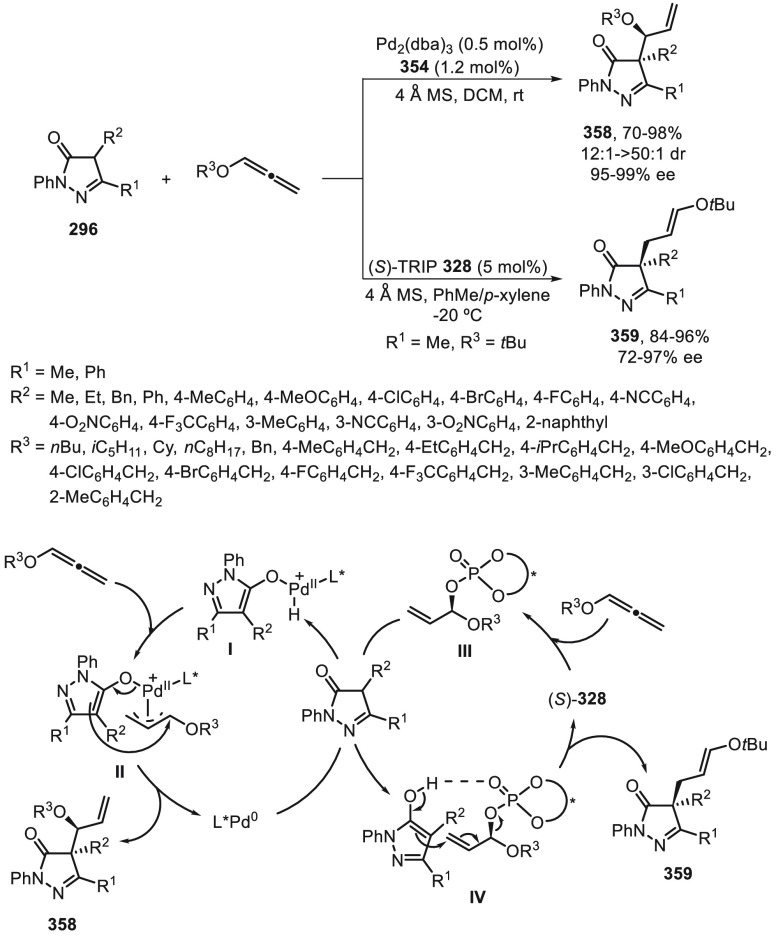
Enantioconvergent and Regiodivergent Pd- and CPA-Catalyzed Hydroalkylation of 1-Alkoxyallenes with Pyrazolones **296**

Jiang and co-workers^240^ reported an enantioselective regiodivergent^241−244^ hydroalkylation of 2-alkoxyallenes with pyrazolones **296** using either palladium or Brønsted acid catalysis. Under Trost’s reaction conditions and Pd/**354** as catalyst without activator, the branched products **358** were exclusively formed with high regio-, diastereo-, and enantioselectivities and also high yields (Scheme 106). However, using **328** as CPA (S)-TRIP, linear products **359** were formed with high yields and regio- and stereoselectivities. In the first case, it was postulated that the acidity of the H at the C4 in the pyrazolone generates the Pd(II) hydride intermediate **I**, which evolves to the π-allylpalladium intermediate **II** to give the branched product **338**. The CPA catalyst might enable the nucleophilic addition to form an alkyloxyallyl phosphate **III**, and after nucleophilic substitution through a hydrogen-bonding interaction in **IV**, it facilitates the resulted linear allylated pyrazolones **359**.

**Scheme 107:**
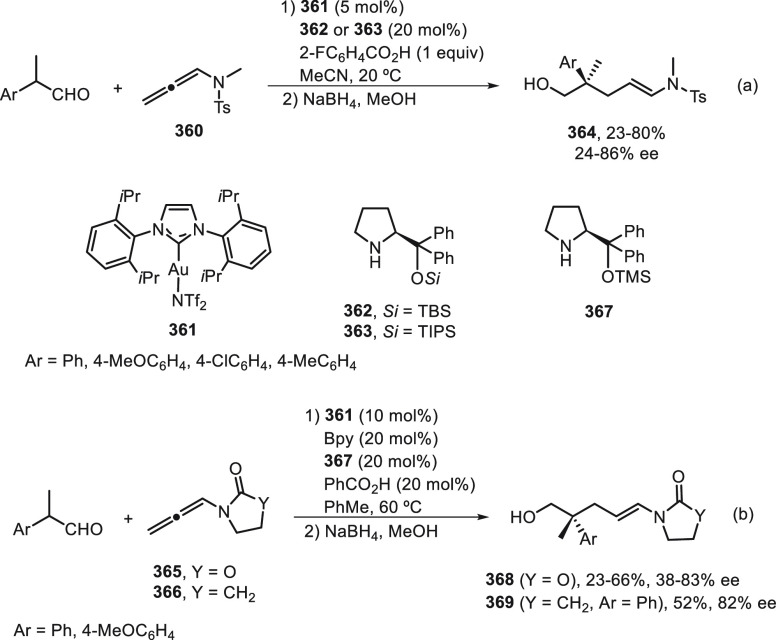
Enantioconvergent Au(I)- and Enamine-Catalyzed Hydroalkylation of Allenamides **360**, **365**, and **366** with Aldehydes

Two Spanish groups^245,246^ reported the enantioconvergent hydroalkylation of aldehydes with allenamides under gold and enamine synergistic catalysis. González and co-workers^245^ employed IPrAuNTf_2_**361**/prolinols **362** or **363** as catalysts in the presence of 2-fluorobenzoic acid in MeCN at room temperature for the hydroalkylation of allenamide **360** to give, after reduction with NaBH_4_, products **364** with moderate yields and enantioselectivities (Scheme 107a). Conversely, Mascareñas, López, and co-workers^246^ employed allenamides **365** and **366** and IPrAuNTf_2_**361**/prolinol **367** as catalysts in the presence of benzoic acid in toluene at 60 °C followed by reduction with NaBH_4_ to provide products **368** and **369** with moderate yields and enantioselectivities (Scheme 107b).

**Scheme 108:**
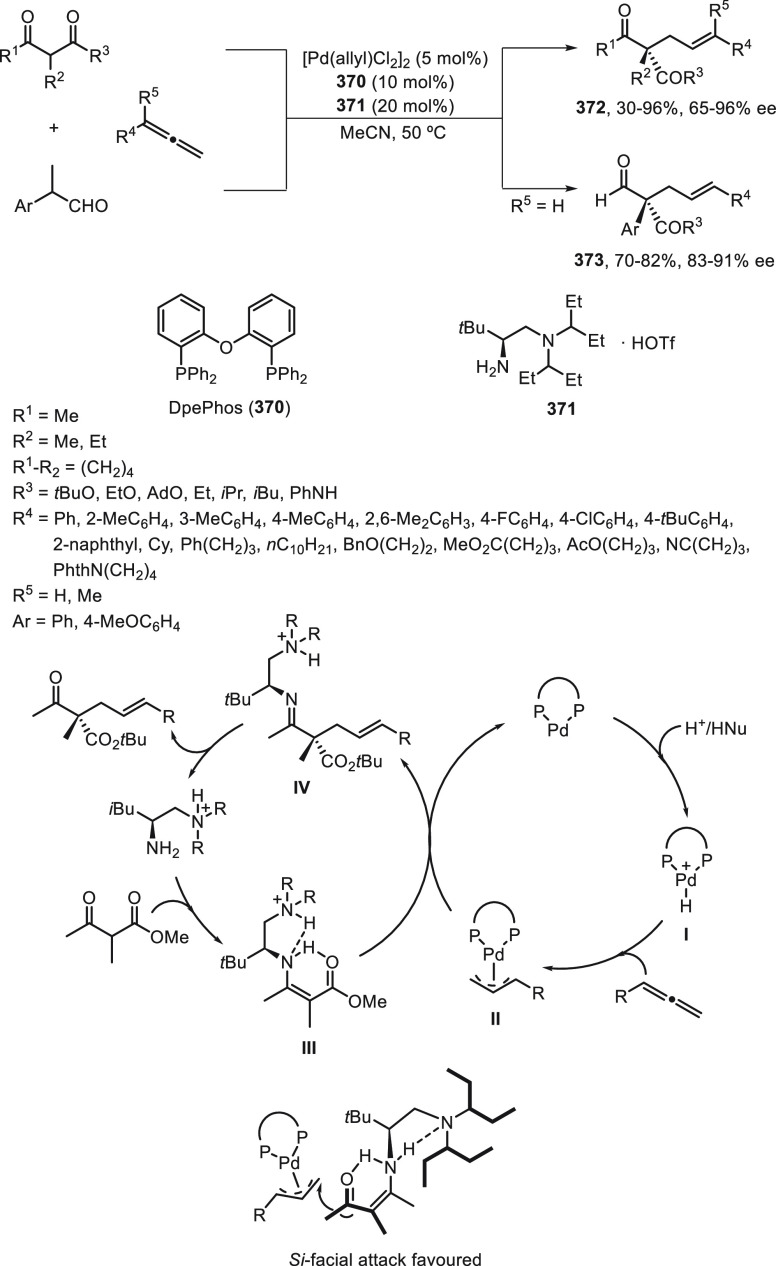
Enantioconvergent Pd- and Amine-Catalyzed Hydroalkylation of Allenes with β-Keto Carbonyl Compounds and Aldehydes

Enantioconvergent hydroalkylations of terminal allenes with β-keto carbonyl compounds and aldehydes have also been performed under dual Pd and amine catalysis by Luo and co-workers.^247^ These reactions were carried out with Pd and the phosphine DpePhos **370** as ligand for the hydrometalation step of the allene and with the chiral primary amine **371** as organocatalyst for the formation of intermediate enamines (Scheme 108). In both cases, the corresponding linear allylic systems **372** and **373** were obtained in up to 96% yield and 96% ee for compounds **372** and up to 82% yield and 91% ee for products **373**. In Scheme 108 are depicted the two catalytic cycles for β-keto carbonyl compounds by intermediacy of Pd complexes **I** and **II** and by enamine **III** and imine **IV**. DFT calculations showed that the bulky tertiary amino group in the enamine **III** blocks the *Re* face for the attack of the π-allylpalladium **II**. The steric effect may explain the exclusive linear selectivity in the allene addition step.

**Scheme 109:**
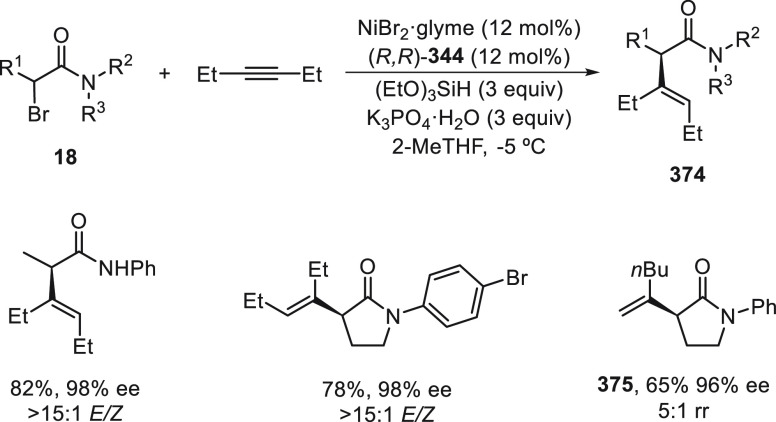
Enantioconvergent Ni-Catalyzed Hydroalkylation of Alkynes with α-Bromo Amides **18**

Fu and co-workers^232^ reported the enantioconvergent hydroalkylation of alkynes with racemic secondary bromides under Ni catalysis in combination with triethoxysilane, as previously mentioned for alkenes in Scheme 99. In this case, the corresponding α-vinyl-substituted amides **374** were obtained by reaction of α-bromo amides **18** with 3-hexyne in >15:1 *E/Z* ratio (Scheme 109). Terminal 1-hexyne gave mainly compound **375** in 5:1 regioisomeric ratio, 65% yield, and 96% ee.

**Scheme 110:**
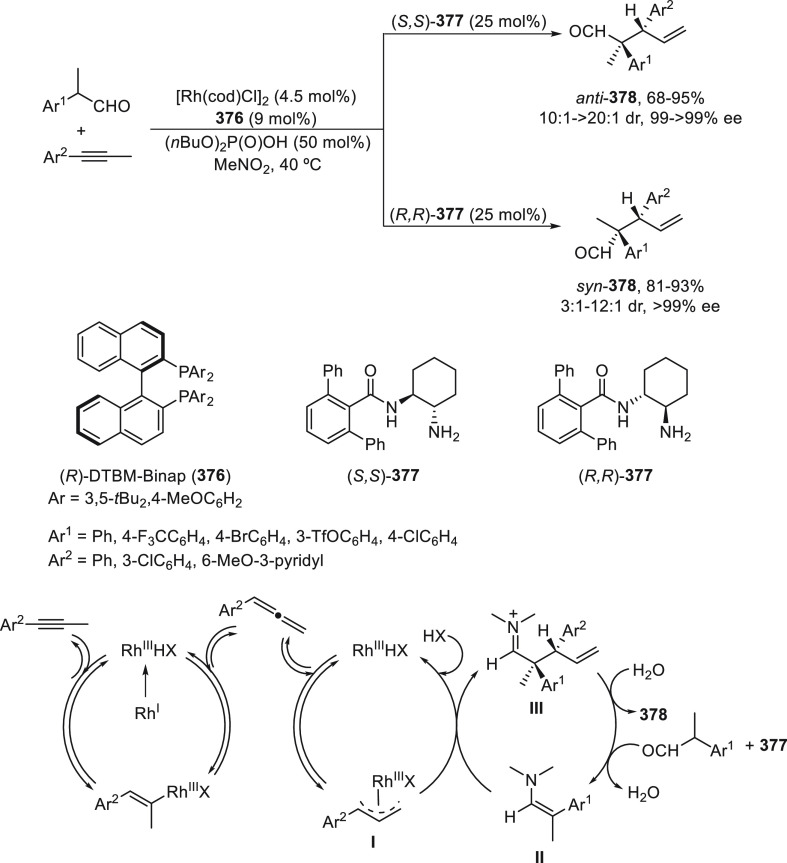
Enantioconvergent and Stereodivergent Rh- and Amine-Catalyzed Hydroalkylation of Alkynes with Aldehydes

For the enantioconvergent hydroalkylation of alkynes with aldehydes, Cruz and Dong^248^ employed a synergistic catalyst using Rh and Jacobsen’s amine. In this case, a chiral Rh-hydride generates the π-allyl species, and the amine generates the enamine of the racemic aldehyde. Chiral Rh complex (*R*)-DTBM-Binap **376** was used as ligand, and diamine **377** was used as chiral organocatalyst in the presence of di-*n*-butylphosphoric acid for the generation of the Rh–H catalyst. They also found a stereodivergent^249^ process by using enantiomeric amines (*R,R*)-**377**. Thus, (*S,S*)-**377** provided the *anti*-products **378**, whereas (*R,R*)-**377** afforded products *syn*-**378** in up to >20:1 regioselectivity (Scheme 110). According to Breit et al.,^250^ Rh-hydride catalysis can promote isomerization of alkynes to generate allenes, which undergo Rh–H insertion to provide electrophilic π-allylRh species **I**. In a third catalytic cycle, the formation of the enamine **II** takes place, and the attack to complex **I** gives the iminium **III** precursor of products **378**.

In summary, for the enantioconvergent functionalization of alkenes with racemic alkyl bromides, a Ni-catalyzed enantioselective reaction in the presence of (EtO)_3_SiH promotes the formation of Ni-hydride for addition to the carbon–carbon double bond for further cross-coupling with the radical formed from the electrophilic alkyl bromide. This process is a suitable alternative to the conventional cross-coupling of alkyl halides with organometallics or nucleophiles and allows the formation of C(sp^3^)–C(sp^3^) bonds. However, for the cross-coupling of racemic nucleophiles and allenes, palladium-catalyzed processes are mainly used. In this case, the formation of a Pd-hydride intermediate triggers the reaction with subsequent hydropalladation of the allene to form an electrophilic π-allylpalladium intermediate, which is attacked by the nucleophile. Alternatively, enantioconvergent hydroalkylation of alkynes has been achieved either under the Ni-catalyzed strategy, as in the case of alkenes, or under Rh-hydride isomerization to allenes and the formation of the π-allylrhodium catalyst followed by attack of the racemic nucleophile.

## 7 Enantioconvergent Hydrogen Autotransfer

**Scheme 111:**
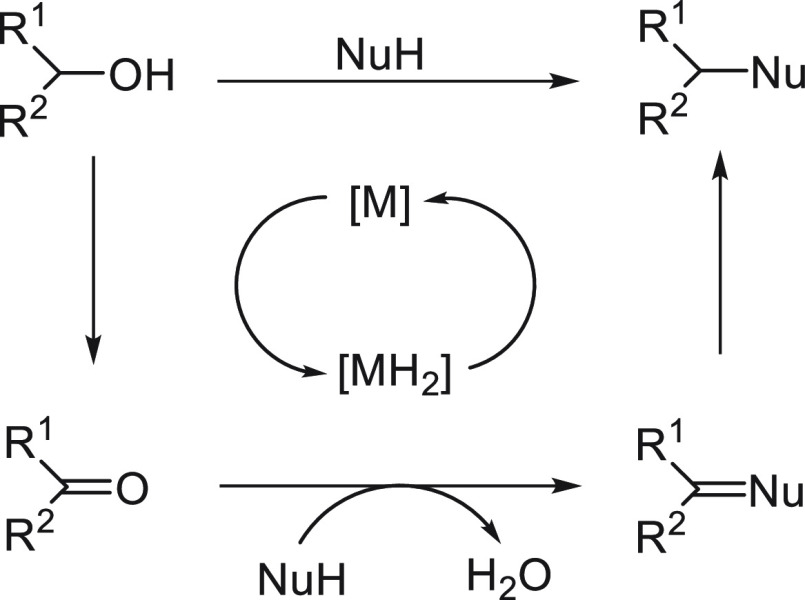
Hydrogen Autotransfer Catalysis

Borrowing hydrogen chemistry or hydrogen autotransfer^251−254^ is a division of hydrogenation reactions in which a catalyst, usually a transition metal [M], first oxidizes an alcohol to form a carbonyl compound and [MH_2_]. After condensation of the carbonyl compound with the nucleophile (usually an amine or enolate), the borrowed hydrogen is transferred to the intermediate (Scheme 111). Enantioconvergent hydrogen autotransfer converts racemic secondary alcohols into enantioenriched amines through C–N bond-forming reactions or ketones with a stereocenter at the β-position through C–C bond-forming processes. The enantiodetermining step is the hydrogen transfer to the intermediate R^1^R^2^C=Nu under transition metal catalysis. This reaction, mainly catalyzed by Ru and Ir complexes, has been used in combination with chiral phosphines and normally releases water as a byproduct, therefore making it an atom-efficient process.

### 7.1 C–N Bond-Forming Reactions

**Scheme 112:**
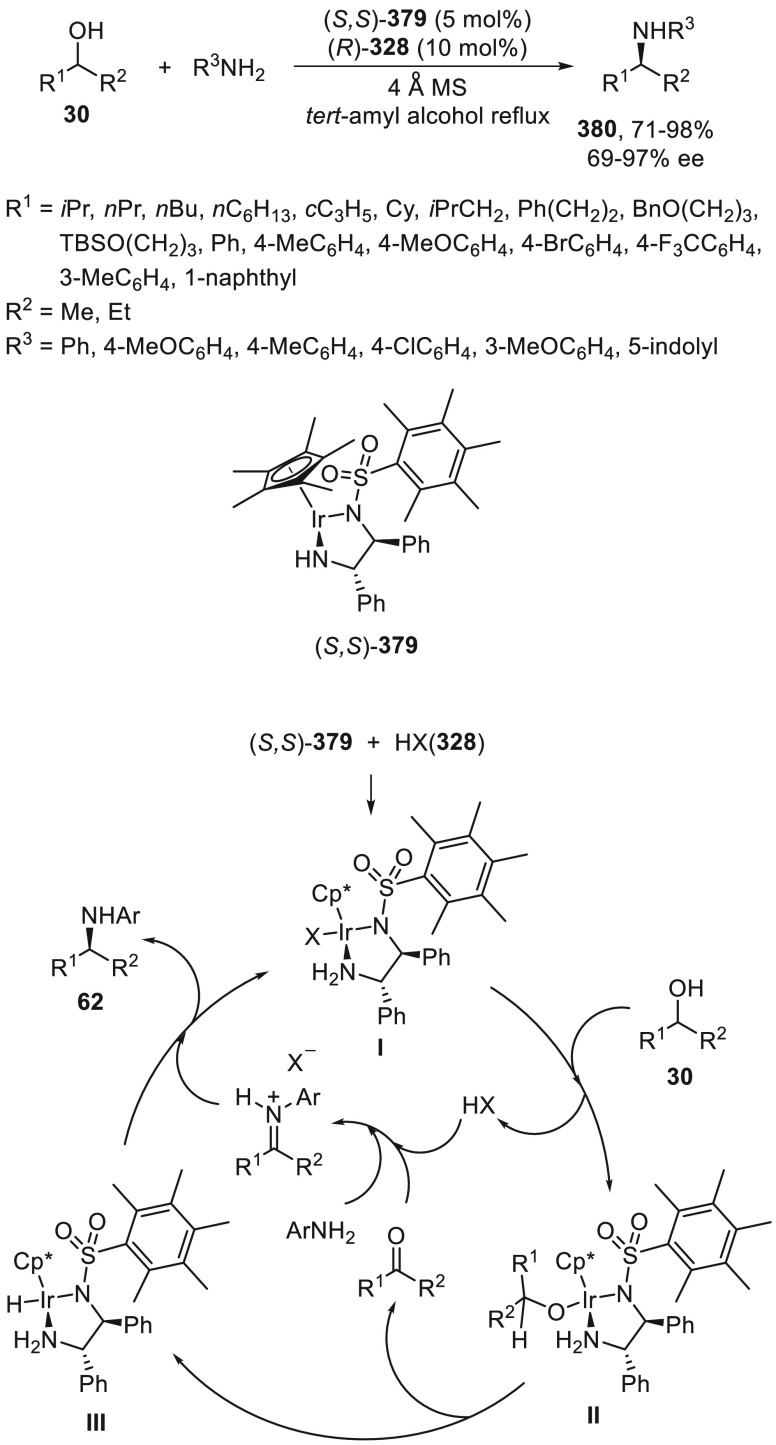
Enantioconvergent Ir- and CPA-Catalyzed Amination of Alcohols **30** by Hydrogen Autotransfer

Enantioconvergent amination of racemic alcohols **30** by hydrogen autotransfer was described first by Zhao and co-workers.^255^ Enantioenriched amines **380** were prepared by cooperative catalysis using the Ir complex (*S,S*)-**379** and CPA (*R*)-TRIP **328**, which promoted the condensation of ketones with anilines in the presence of a 4 Å MS under *tert*-amyl alcohol reflux (Scheme 112). In the proposed catalytic cycle, complex **I** is formed from the Ir complex and CPA and reacts with the alcohol to give intermediate **II**. After formation of the ketone and the iridium hydride **III**, this complex attacks the iminium species to produce the amine and the catalyst **I**.

**Scheme 113:**
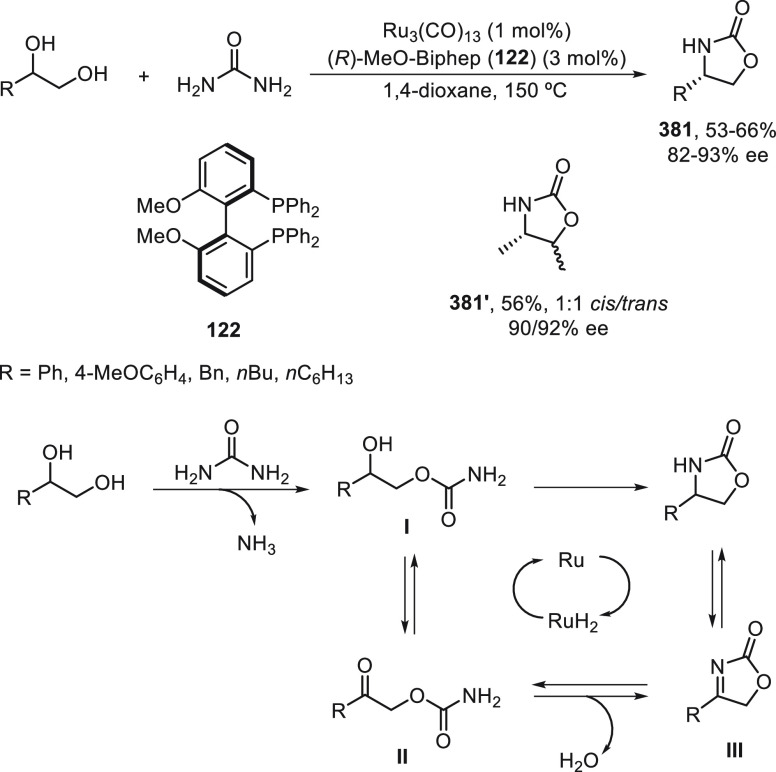
Enantioconvergent Ru-Catalyzed Amidation of 1,2-Diols by Hydrogen Autotransfer

Beller and co-workers^256^ described the enantioconvergent amidation of diols with urea under Ru_3_(CO)_13_/(*R*)-MeO-Biphep (**122**) catalysis for the synthesis of oxazolidin-2-ones **381** (Scheme 113). In this process, the sequential formation of C–O and C–N bonds takes place chemo- and regioselectively with good yields and enantioselectivities for terminal diols. The internal 2,3-butanediol gave a 1:1 mixture of *cis/trans* diastereomers **381′** in 56% yield and 90/92% ee, respectively. In the proposed catalytic cycle, initially the diol reacts with urea to give carbamate **I**, which would give ketone **II** by Ru-catalyzed dehydrogenation. Intramolecular condensation provides oxazoline **III**, which gives rise to the oxazolidin-2-one after reduction.

**Scheme 114:**
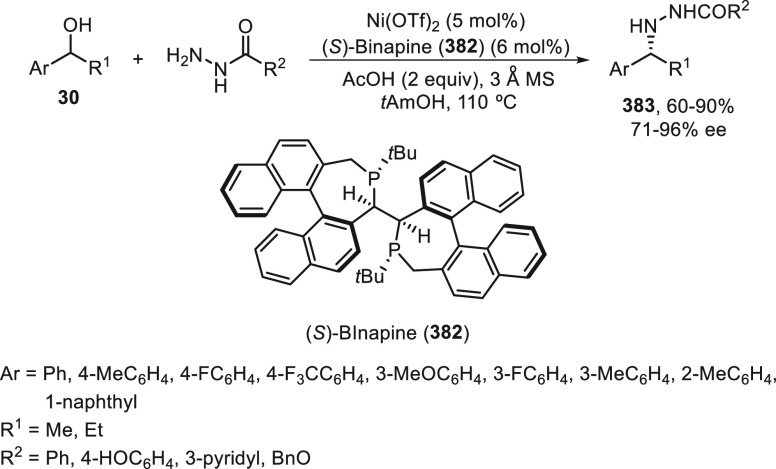
Enantioconvergent Ni-Catalyzed Amination of Alcohols **30** by Hydrogen Autotransfer

A Ni-catalyzed *N*-alkylation of hydrazides and anilines has been described by Tang, Zhou, and co-workers.^257^ The enantioconvergent process was carried out using Ni(OTf)_2_/(*S*)-Binapine (**382**) as catalyst for the amination of benzylic alcohols **30** with hydrazides to furnish enantioenriched products **383** in up to 96% ee (Scheme 114). The reaction was carried out in the presence of acetic acid and a 3 Å MS in *tert-*amyl alcohol at 110 °C. By further reduction of products **383** with SmI_2_ or Raney Ni, the N–N single bond can be cleaved to give benzyl amines.

**Scheme 115:**
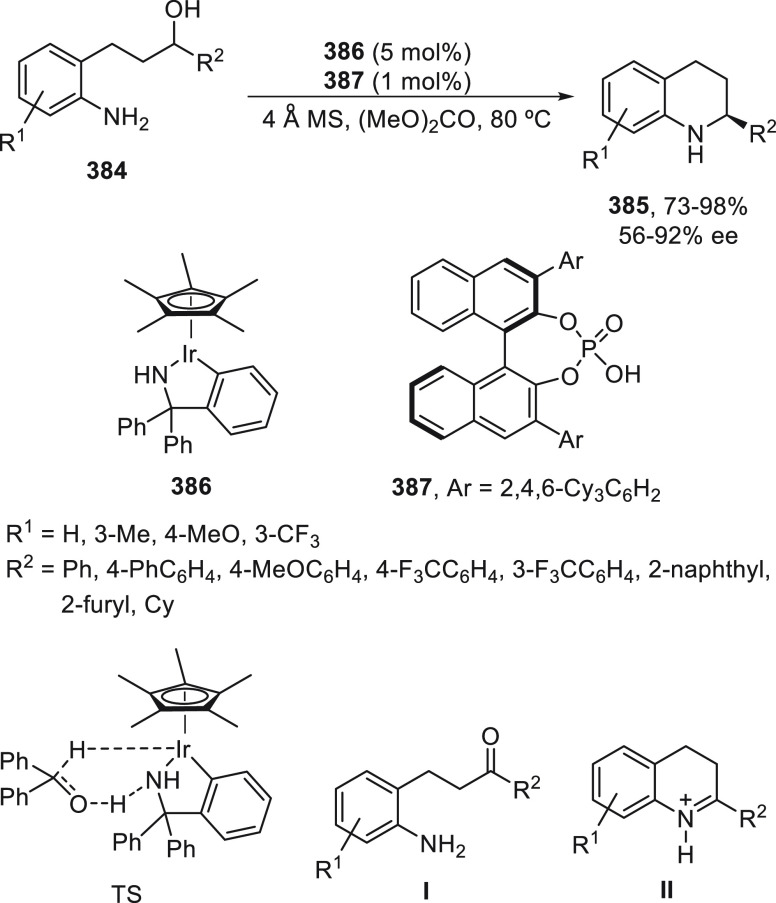
Enantioconvergent Ir- and CPA-Catalyzed Intramolecular Amination of Amino Alcohols **384** by Hydrogen Autotransfer

Zhao and co-workers^258^ applied the cooperative catalysis by an achiral iridacycle and a CPA to the enantioconvergent intramolecular amination of *ortho*-substituted anilines **384** to provide tetrahydroquinolines **385** (Scheme 115). This hydrogen autotransfer occurred under iridacycle **386** and CPA **387** catalysis and a 4 Å MS as additive in dimethyl carbonate at 80 °C to give products **385** with high yields and up to 92% ee. A similar catalytic cycle, as depicted in Scheme 112, was proposed. In this case, a concerted transition state (TS) can be formed, which evolves to ketone **I**, which is a precursor of the tetrahydroquinoline derivative, by intermediacy of the iminium species **II**.

**Scheme 116:**
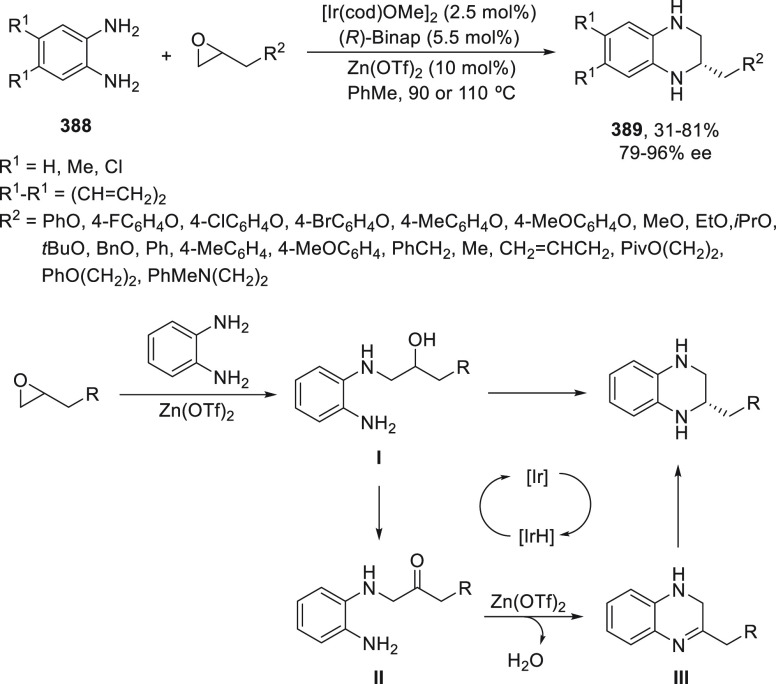
Enantioconvergent Iridium- and Zn(OTf)_2_-Catalyzed Epoxide Opening/Intramolecular Amination with *ortho-*Diamines **388** by Hydrogen Autotransfer

For the enantioconvergent synthesis of 1,2,3,4-tetrahydroquinoxalines **389**, Zhao’s group^259^ described the reaction of aromatic *ortho*-diamines **388** with epoxides (Scheme 116). This transformation was carried out under Ir/(*R*)-Binap catalysis with Zn(OTf)_2_ as Lewis acid in toluene at 90 or 110 °C. In this method, the Lewis acid catalyzes the epoxide ring opening to give the amino alcohol **I**, which undergoes Ir-catalyzed hydrogen autotransfer to ketone **II**. Intramolecular condensation promoted by Zn(OTf)_2_ gives imine **III**, and the enantiodetermining reduction of **III** forms the product.

**Scheme 117:**
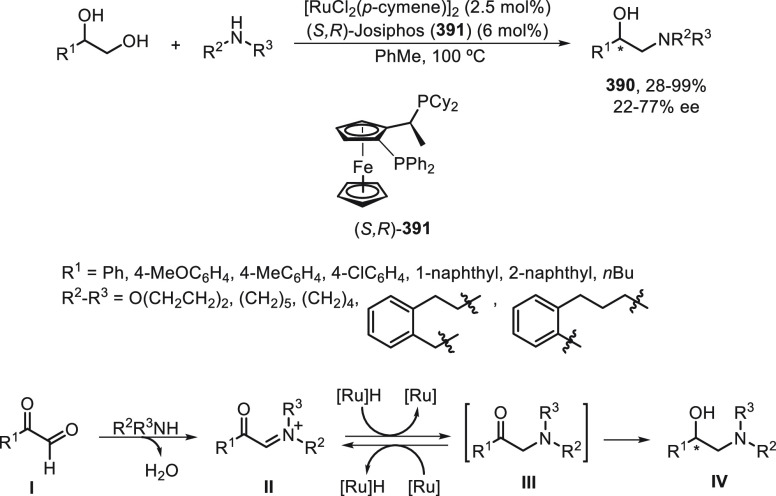
Enantioconvergent Ru-Catalyzed Monoamination of 1,2-Diols by Hydrogen Autotransfer

Monoamination of 1,2-diols **380** with secondary amines to give β-amino alcohols **390** was described in 2013 by Oe and co-workers.^260^ The enantioconvergent hydrogen autotransfer took place under [RuCl_2_(*p*-cymene)]_2_/(*S,R*)-Josiphos **391** catalysis in toluene at 100 °C to furnish products **390** in up to 99% yield and 77% ee (Scheme 117). According to mechanistic studies, it was proposed that the diol is converted into oxo aldehyde **I** by Ru-catalyzed double dehydrogenation. This oxo aldehyde **I** reacts with the amine to afford the iminium intermediate **II**, which is reduced successively to amino ketone **III** and amino alcohol **IV**. Absolute configuration of products **390** was not determined.

**Scheme 118:**
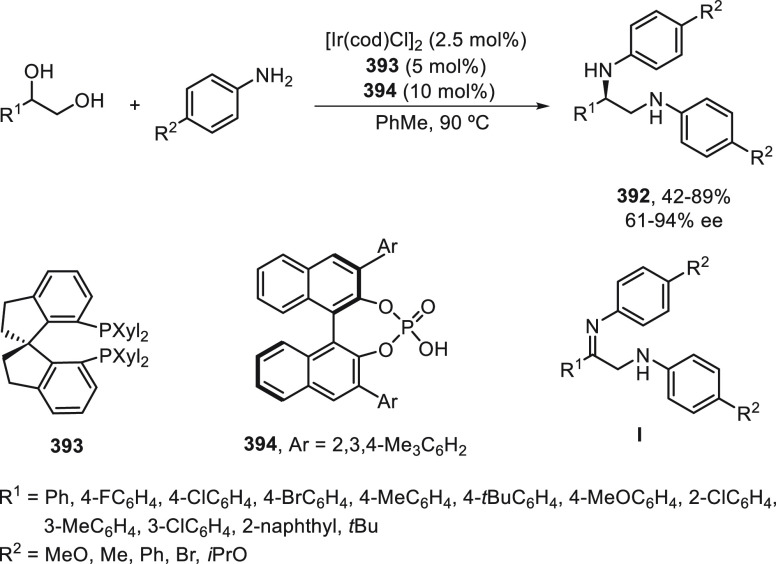
Enantioconvergent Ir- and CPA-Catalyzed Diamination of 1,2-Diols by Hydrogen Autotransfer

Diamination of 1,2-diols with anilines to provide 1,2-diamines **392** was recently performed by Zhao’s group.^261^ For this purpose [Ir(cod)Cl]_2_/bisphosphine **393** and CDA **394** were used as catalysts to provide diamines **392** in good yields and enantioselectivities (Scheme 118). In the proposed mechanism for this enantioconvergent diamination, the intermediacy of β-amino imine **I** would give, after reduction, the corresponding diamine. A DyKAT mechanism has been also proposed.

**Scheme 119:**
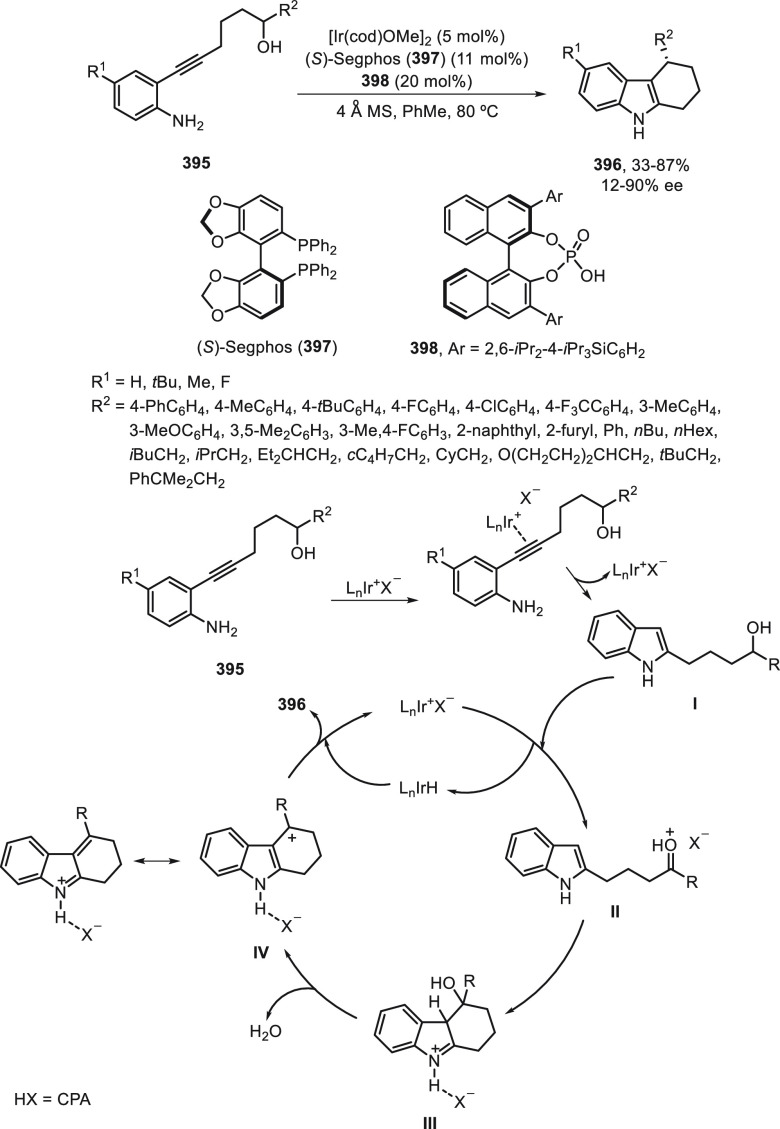
Enantioconvergent Ir- and CPA-Catalyzed Indolization/Substitution of Alkynyl Anilines **395**

Indolization/enantioconvergent substitution of alcohols via hydrogen autotransfer of alcohol-containing alkynyl anilines **395** provided enantioenriched 2,3-fused tricyclic indoles **396** (Scheme 119). This tandem transformation described by Zhao’s group^262^ was enabled by cooperative catalysis of Ir/(*S*)-Segphos (**397**) with a 4 Å MS as additive in toluene at 80 °C. A plausible mechanism has been proposed on the basis of experimental studies. First, starting product **395** cyclizes to indole intermediate **I** catalyzed by cationic iridium phosphate. This indole enters in the borrowing hydrogen catalytic cycle to form intermediate **II** by Ir-catalyzed dehydrogenation. Subsequent acid-catalyzed intramolecular Friedel–Crafts reaction gives intermediate **III** and, after dehydration, the α,β-unsaturated imine **IV**. Final reduction of **IV** yields the tricyclic indole product **396**.

Enantioconvergent C–N bond formation by alkylation of amines with racemic alcohols can be efficiently performed under asymmetric hydrogen autotransfer by a combination of iridium and phosphoric acid catalysis successfully developed by Zhao and co-workers.

### 7.2 C–C Bond-Forming Reactions

**Scheme 120:**
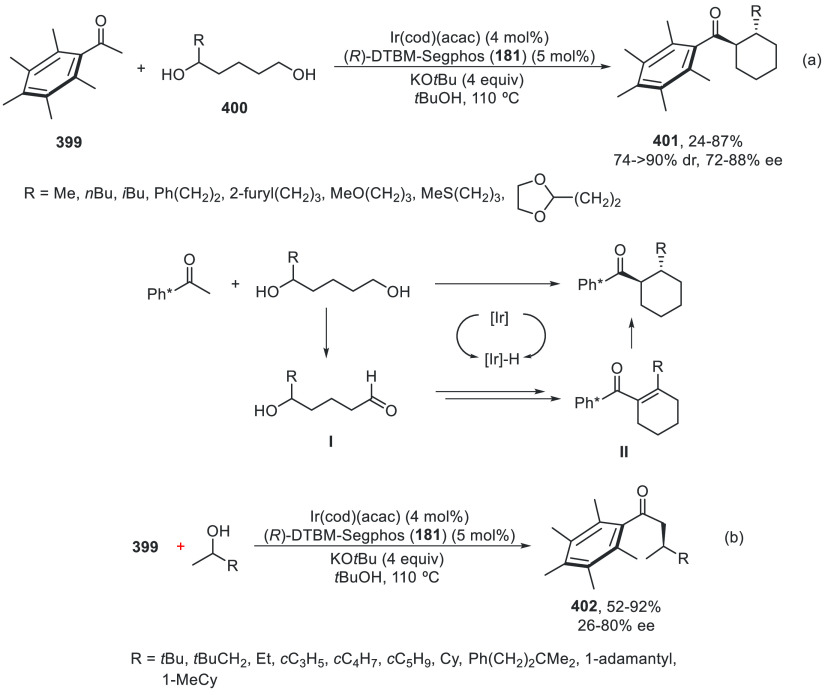
Enantioconvergent Ir-Catalyzed Alkylation of Ketones **399** with Alcohols by Hydrogen Autotransfer

Direct alkylation of enolates with unactivated alcohols using hydrogen autotransfer conditions allows the formation of C–C bonds.^251−254^ Donohoe and co-workers^263^ recently reported an enantioconvergent alkylation of pentamethylphenyl (Ph*) ketones **399** with racemic 1,5-diols **400** to give, through a (5 + 1) annulation process, enantioenriched cyclohexanes **401** with good yields and diastereo- and enantioselectivities (Scheme 120a). This annulation was performed under Ir(cod)(acac)/(*R*)-DTBM-Segphos (**181**) catalysis in the presence of KO*t*Bu and in *t*BuOH as solvent at 110 °C. The Ir catalyst oxidized the primary alcohol of **400** to aldehyde **I** followed by aldol condensation with the ketone **399** to give intermediate **II** after loss of water. The starting ketones **399** with the Ph* group play a key role by orthogonal orientation to the carbonyl group in avoiding competing reduction and homodimerization processes. Moreover, aryl Ph* derivatives can be transformed via an *ipso* substitution process into other functional groups.^264^ The same research group^265^ further described the intermolecular version of this alkylation of ketones **399** with racemic secondary alcohols to give enantioenriched β-alkylated ketones **402** with moderate enantioselectivities under the previously mentioned reaction conditions (Scheme 120b). The cleavage of the acyl Ph* group provided carboxylic acid esters, thioesters, amides, and alcohols.

**Scheme 121:**
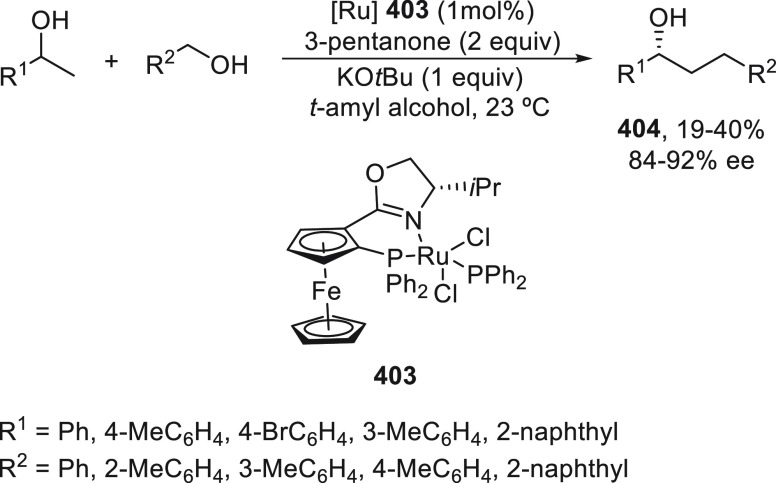
Enantioconvergent Ru-Catalyzed Guerbet Reaction of Secondary Alcohols with Primary Alcohols

The Guerbet reaction, which was described more than one century ago by Marcel Guerbet,^265,266^ is the coupling of two primary alcohols to give a new alcohol. However, only recently have two Chinese groups reported independently the asymmetric Guerbet reaction. Zhao and co-workers^267^ reported the asymmetric Guerbet reaction of racemic secondary alcohols with primary alcohols at room temperature using a chiral Ru complex **403** as catalyst (Scheme 121). The reaction must be carried out in the presence of 3-pentanone as hydrogen acceptor at the beginning of the reaction and KO*t*Bu as base in *tert*-amyl alcohol. Alcohols **404** were obtained with moderate yields because kinetic resolution of the starting secondary alcohols occurred simultaneously.

**Scheme 122:**
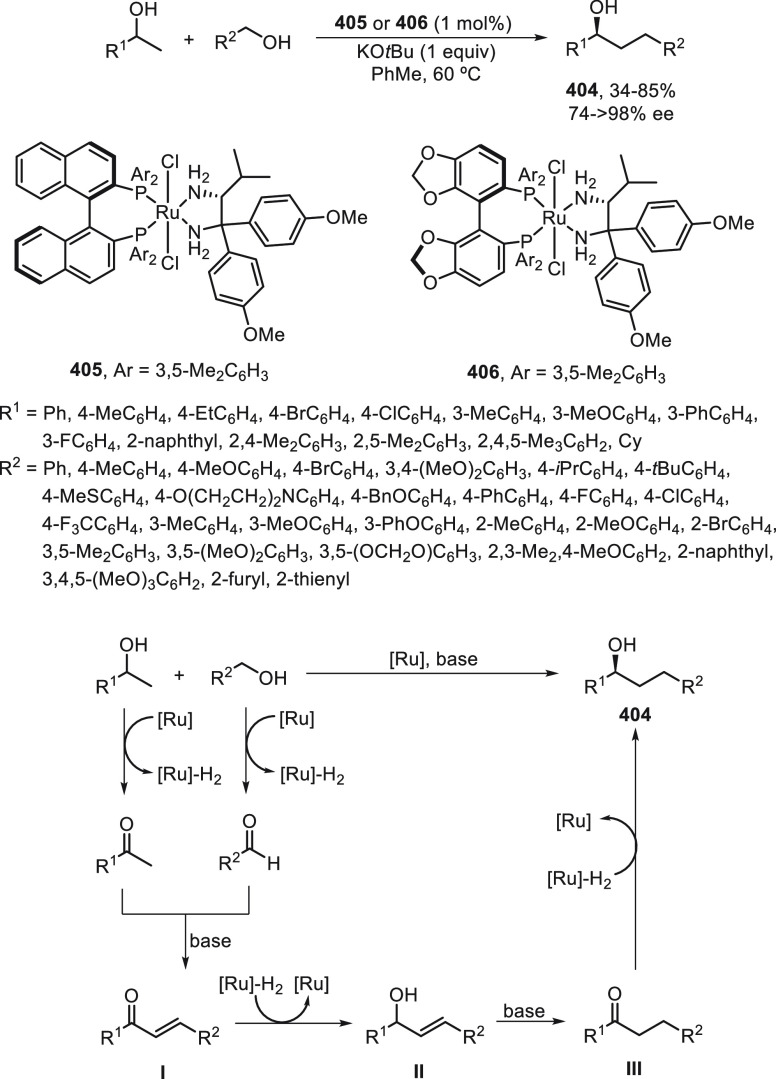
Enantioconvergent Ru-Catalyzed Guerbet Reaction of Secondary Alcohols with Primary Ones

Conversely, Wang and co-workers^268^ used commercially available classic Noyori Ru(II)-diamine-diphosphine **405** or **406** as catalysts and KO*t*Bu as base in toluene at 60 °C (Scheme 122). The resulting alcohols **404** were isolated with good yields and enantioselectivities and can be performed at the gram scale. Mechanistic studies support the catalytic cycle depicted in Scheme 122. The two starting alcohols are dehydrogenated to give Ru-hydride intermediates and the two carbonyl compounds. They gave, after aldol condensation, the α,β-unsaturated ketone **I**, which is then reduced to produce the allylic alcohol **II**. After a base-catalyzed isomerization, intermediate **II** affords a ketone **III**, which is finally reduced by a Ru-hydride to form the enantioenriched alcohol resembling the Noyori asymmetric hydrogenation.

In the case of C–C bond-forming reactions, intramolecular aldol condensation has been carried out under Ir/DTBM-Segphos catalysis of pentamethylphenyl ketones with diols to provide cyclohexane derivatives and with secondary alcohols to the corresponding β-alkylated ketones. Enantioconvergent Guerbet reactions have been efficiently performed using chiral Noyori-type Ru catalyst for secondary alcohols with primary alcohols to provide enantioenriched γ-alkylated secondary alcohols.

## 8 Other Metal-Catalyzed Enantioconvergent Reactions

In this Section, enantioconvergent transition-metal-catalyzed allylations of carbonyl compounds, aminations of 3-bromooxindoles, and other processes, such as addition of 3-bromooxindoles to silyl ketene imines, allenylation of carbonyl groups, and oxidation of β-keto esters, are considered.

### 8.1 Allylation of Carbonyl Compounds

**Scheme 123:**
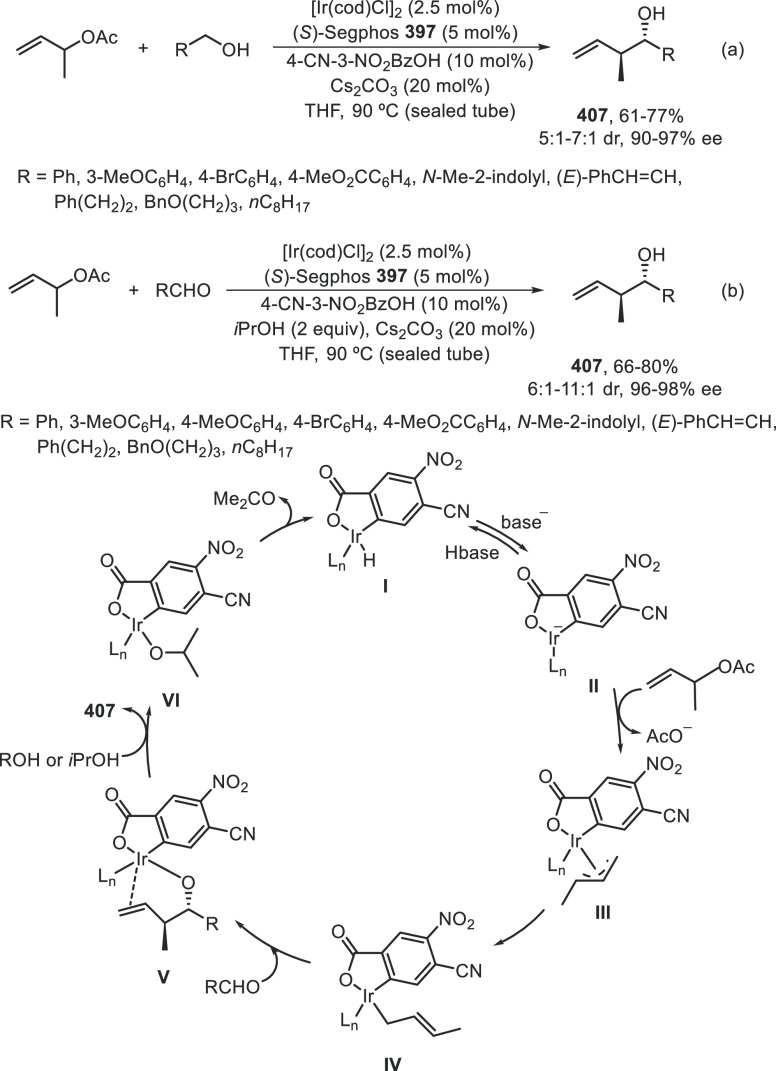
Enantioconvergent Ir-Catalyzed Diastereoselective Crotylation of Primary Alcohols or Aldehydes

Enantioconvergent allylation of carbonyl compounds starting from racemic allylic derivatives under transition metal catalysis affords diastereomeric addition products. Krische and co-workers^269^ described the crotylation of primary alcohols with 3-acetoxy-1-butene via transfer hydrogenative conditions. They employed an *ortho*-cyclometalated iridium catalyst **I** generated *in situ* from [Ir(cod)Cl]_2_, 4-cyano-3-nitrobenzoic acid, and (*S*)-Segphos (**397**) (Scheme 123). This carbonyl crotylation took place with total regioselectivity to provide products **407** with good *anti-*diastereo- and high enantioselectivity (Scheme 123a). Under the same reaction conditions, but using isopropanol as terminal reductant, the same alcohols **407** were obtained by crotylation of aldehydes (Scheme 123b). In a simplified catalytic mechanism, the cyclometalated iridium hydride **I** is deprotonated by Cs_2_CO_3_ to give the anionic iridacycle **II**. Oxidative addition of α-methyl allyl acetate forms the π-crotyliridium complex **III**. Addition of the (*E*)-α-crotyliridium complex **IV** to the aldehyde through a chairlike transition state delivers the *anti*-homoallyl iridium alkoxide **V**. Exchange of the homoallylic alcohol **407** by isopropanol or the primary alcohol gives intermediate **VI**, which undergoes β-hydride elimination to regenerate complex **I**. The same research group^270^ described an enhancement of the *anti*-diastereo- and enantioselectivity by using an isolable iridium complex **408** at lower temperature (60 °C) with K_3_PO_4_ as base and 5 equiv of H_2_O to give products **407** with up to 91% yield, >20:1 dr, and 99% ee.

**Scheme 124:**
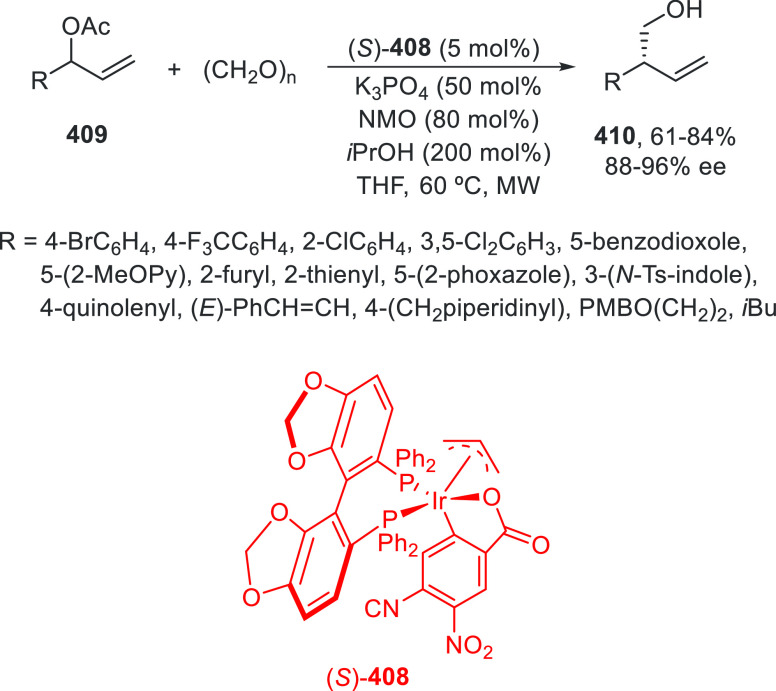
Enantioconvergent Ir-Catalyzed Reductive Coupling of Formaldehyde with Allylic Acetates **409**

When they used^271^ paraformaldehyde as an electrophile under similar reaction conditions, the enantioconvergent reductive coupling with branched allylic acetates **409** provided regioselectively primary homoallylic alcohols **410** in up to 96% ee (Scheme 124). This hydroxymethylation reaction was performed using the Segphos-derived iridacycle (*S*)-**408** in the presence of *N*-methylmorpholine oxide (NMO) as additive under microwave heating to avoid the formation of catalytic-inactive iridium carbonyl complexes. A similar mechanism as described in Scheme 123 was proposed with the same π-facial discrimination.

**Scheme 125:**
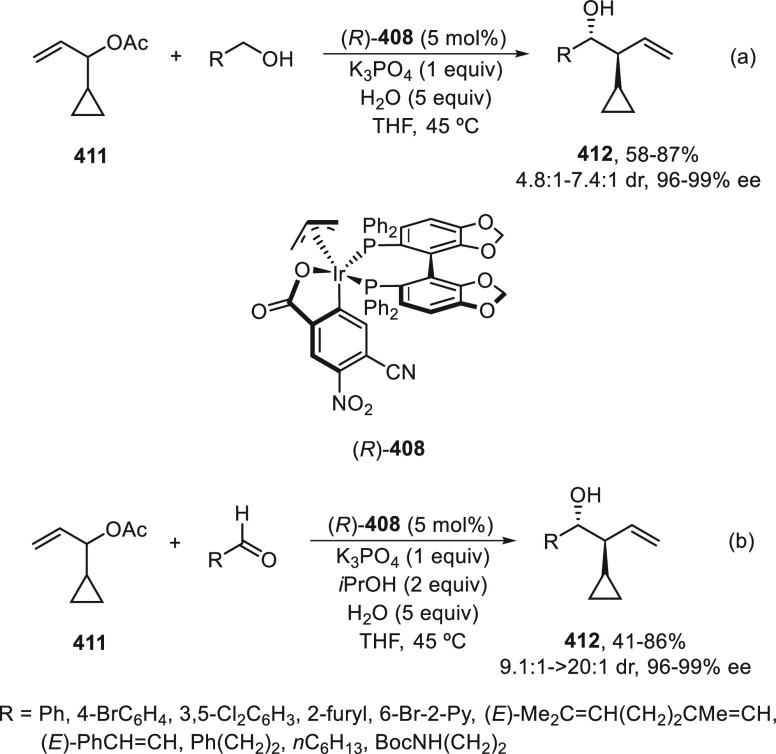
Enantioconvergent Ir-Catalyzed Diastereoselective (α-Cyclopropyl)allylation of Primary Alcohols or Aldehydes

Krische’s group^272^ described the iridium-catalyzed enantioconvergent allylation of carbonyl compounds or their alcohol precursors with α-cyclopropyl allyl acetate **411** (Scheme 125). Under the reaction conditions for iridacycle (*R*)-**408**-catalyzed transfer hydrogenation, the corresponding homoallylic alcohols **412** were obtained with total regioselectivity and high enantioselectivity. This (α-cyclopropyl)allylation can be performed with primary alcohols (a) or aldehydes (b) to give the corresponding products **412** with similar results, although with higher diastereoselectivities than in the case of aldehydes.

**Scheme 126:**
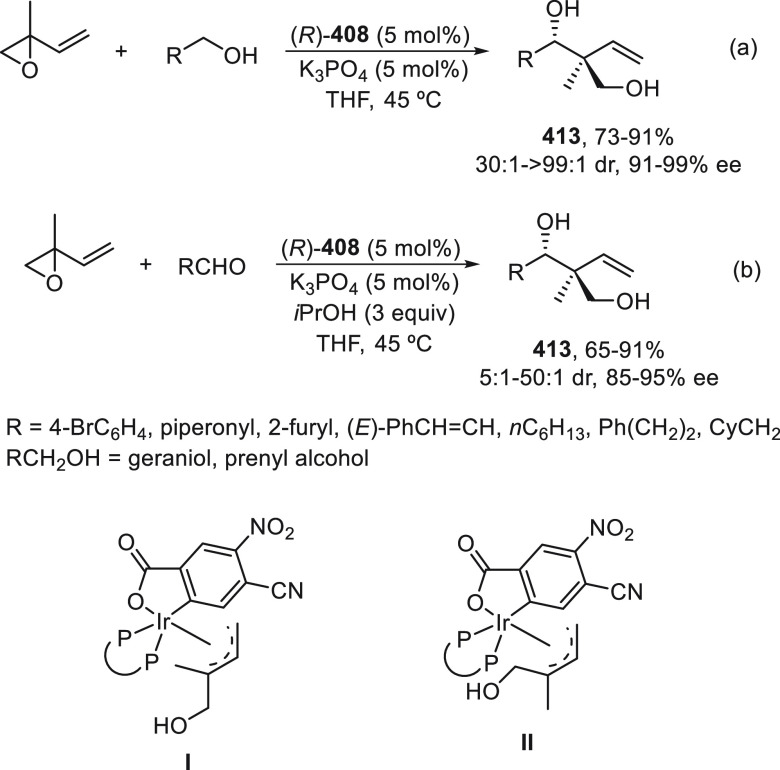
Enantioconvergent Ir-Catalyzed Diastereoselective *tert*-(Hydroxy)prenylation of Primary Alcohols or Aldehydes

Enantioconvergent redox-triggered C–C coupling of alcohols and vinyl epoxides has been developed by Kirsche’s group.^273^ Racemic isoprene oxide reacts with primary alcohols under (*R*)-**408** catalysis to furnish products **413** bearing a quaternary stereocenter with high levels of *anti-*diastereo- and enantioselectivity (Scheme 126a). 1,3-Butadiene epoxide and myrcene oxide reacted with 4-bromobenzyl alcohol to give product **413** with 63% yield, 5:1 dr, and 94% ee in the first case and with 94% yield, 40:1 dr, and 87% ee in the second example. When aldehydes were allowed to react with isoprene oxide in the presence of isopropanol as terminal reductant, prenylated products **413** were isolated with similar yields and diastereoselectivities (Scheme 126b). This *tert-*(hydroxy)prenylation probably takes place through the (*E*)-π-allyliridium intermediate **I**, which minimizes dipole–dipole interactions. Moreover, the reaction would be faster than by intermediacy of the (*Z*)-σ-allyliridium intermediate **II** because of the internal coordination of the hydroxy group to iridium. Compounds **413** were further transformed into enantioenriched 2,3,3-trisubstituted oxetanes and applied to the synthesis of an analogue of the antipsychotic agent haloperidol.^274^

**Scheme 127:**
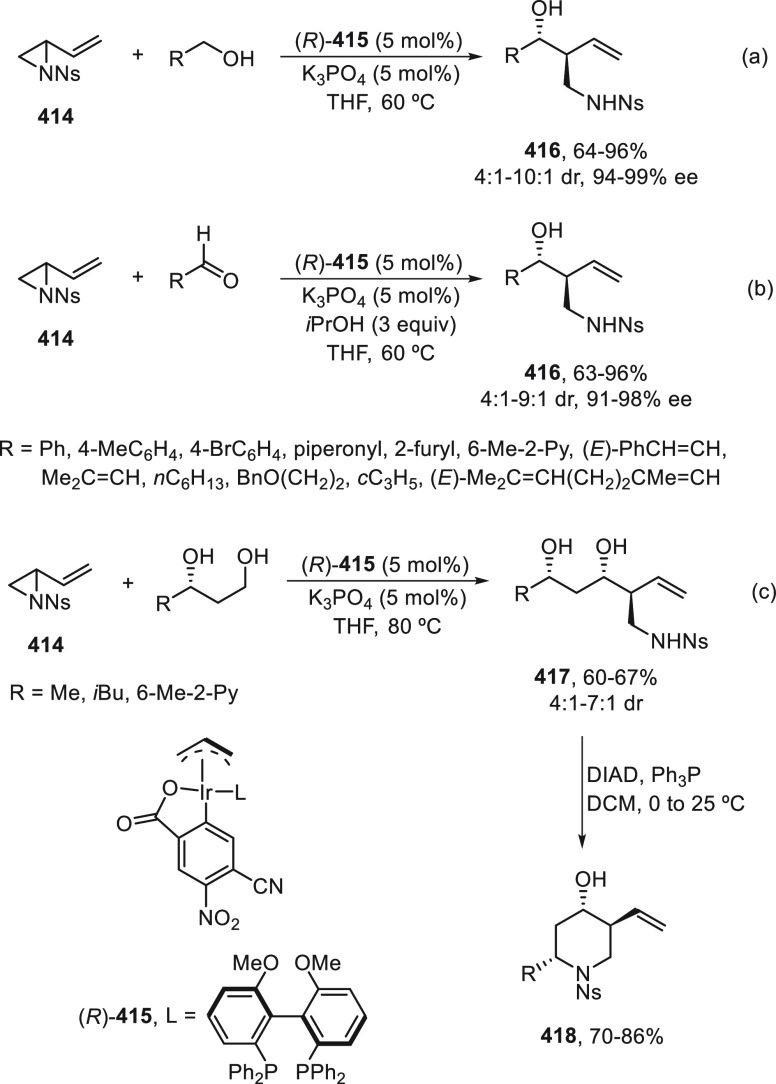
Enantioconvergent Ir-Catalyzed Diastereoselective Coupling of Vinyl Aziridine **414** with Alcohols, Aldehydes, or 1,3-Diols

Another application of this Ir-catalyzed C–C bond-forming transfer hydrogenation is the coupling of vinyl aziridines with alcohols and aldehydes.^275^*N*-(*p*-Nitrophenylsulfonyl) (Ns) vinyl aziridine **414** reacted with primary alcohols to give γ-amino alcohols **416** using iridacycle (*R*)-**415** as catalyst (Scheme 127a). Alternatively, aldehydes reacted in the presence of isopropanol as terminal reductant to provide branched products of (α-aminomethyl)allylation **416**, also with excellent levels of *anti*-diastereo- and enantioselectivity (Scheme 127b). Chiral 1,3-diols reacted with *N*-tosylvinyl aziridine under the same reaction conditions to give products **417** with good yields and stereoselectivities (Scheme 127c). These products **417** were converted into 2,4,5-trisubstituted piperidines **418** by intramolecular Mitsunobu reaction.

**Scheme 128:**
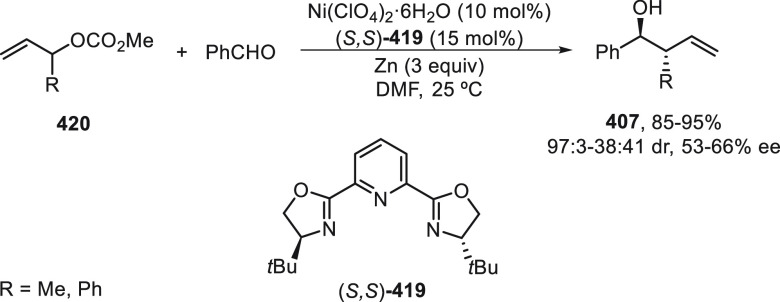
Enantioconvergent Ni-Catalyzed Diastereoselective Reductive Allylation of Benzaldehyde with Allylic Carbonates **420**

The asymmetric reductive allylation of aldehydes with allylic carbonates was performed under Ni(cod)_2_/bis(oxazoline) (*S,S*)-**419** as catalysts using Zn as the terminal reductant.^276^ In this study, some examples on enantioconvergent processes were included. Racemic carbonates **420** reacted with benzaldehyde under Ni(ClO_4_)_2_·6H_2_O/(*S,S*)-**419** catalysis at 25 °C in DMF to provide mainly *anti*-**407** in moderate ee, even at −25 °C (Scheme 128).

**Scheme 129:**
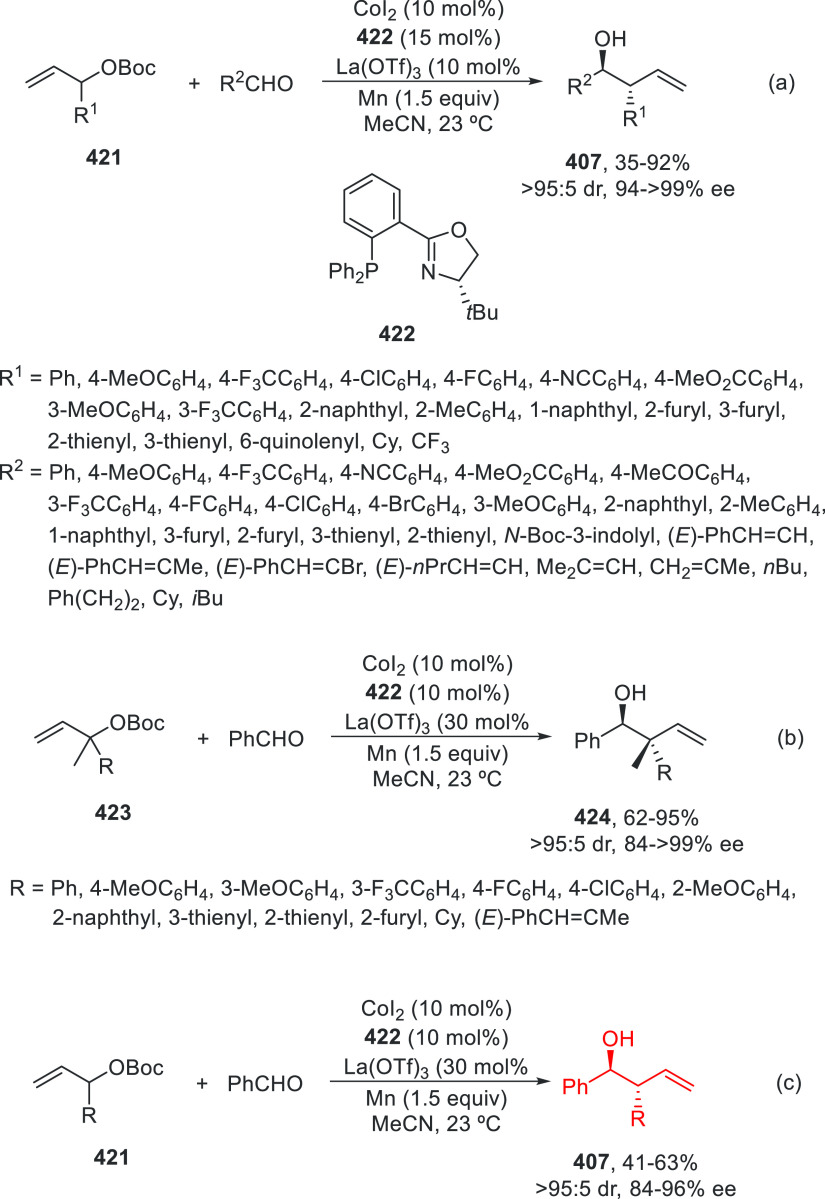
Enantioconvergent Co-Catalyzed Diastereoselective Reductive Allylation of Aldehydes with Allylic Alcohol Derivatives **421** and **423**

Recently, Chong, Meng, and co-workers^277^ reported a general enantioconvergent Co-catalyzed reductive allylation of aldehydes with allylic carbonates **421** bearing one or two substituents at the α-position. By means of CoI_2_/phosphine-oxazoline **422** catalysis, La(OTf)_3_ as Lewis acid, and Mn powder as the terminal reductant in MeCN at room temperature, the corresponding homoallylic alcohols **407** were obtained with good yields and diastereo- and enantioselectivities (Scheme 129a). In the case of branched allylic carbonates **423**, homoallylic alcohols **424** bearing a quaternary stereocenter resulted with good levels of stereoselectivity (Scheme 129b). Racemic allylic alcohols can directly participate in this process to give alcohols **407** with moderate yields and very good diastereo- and enantioselectivities (Scheme 129c). On the basis of experimental observations, the proposed catalytic cycle is depicted in Scheme 129. The Co(I) complex **II**, generated from the Co(II) complex **I** through a single-electron reduction by Mn, undergoes oxidative addition to afford the π-allylCo(III) complex **III** or a single-electron oxidative addition to provide an equilibrium of complexes **III** and **I** and the allyl radical **IV**. This radical **IV** is captured by complex **I**, which is followed by a single electron-reduction that delivers the σ-complex **V** in equilibrium with the π-allylCo complex **VI** and the more reactive complex **VII**. Addition of **VII** to the aldehyde provides the intermediate **VIII**, which evolves by a single-electron reduction with Mn to give the homoallylic alcoholate and complex **II**.

Diastereo- and regioselective enantioconvergent allylation of carbonyl compounds are efficiently carried out under Ir catalysis using transfer hydrogenation conditions to provide homoallylic alcohols with high yields and stereoselectivities. Apart from allylic esters, vinyl epoxides and aziridines can also be employed as allylating reagents. Alternatively, Ni and Co complexes work as catalysts under radical conditions.

### 8.2 Propargylation of Alcohols and Phenols

**Scheme 130:**
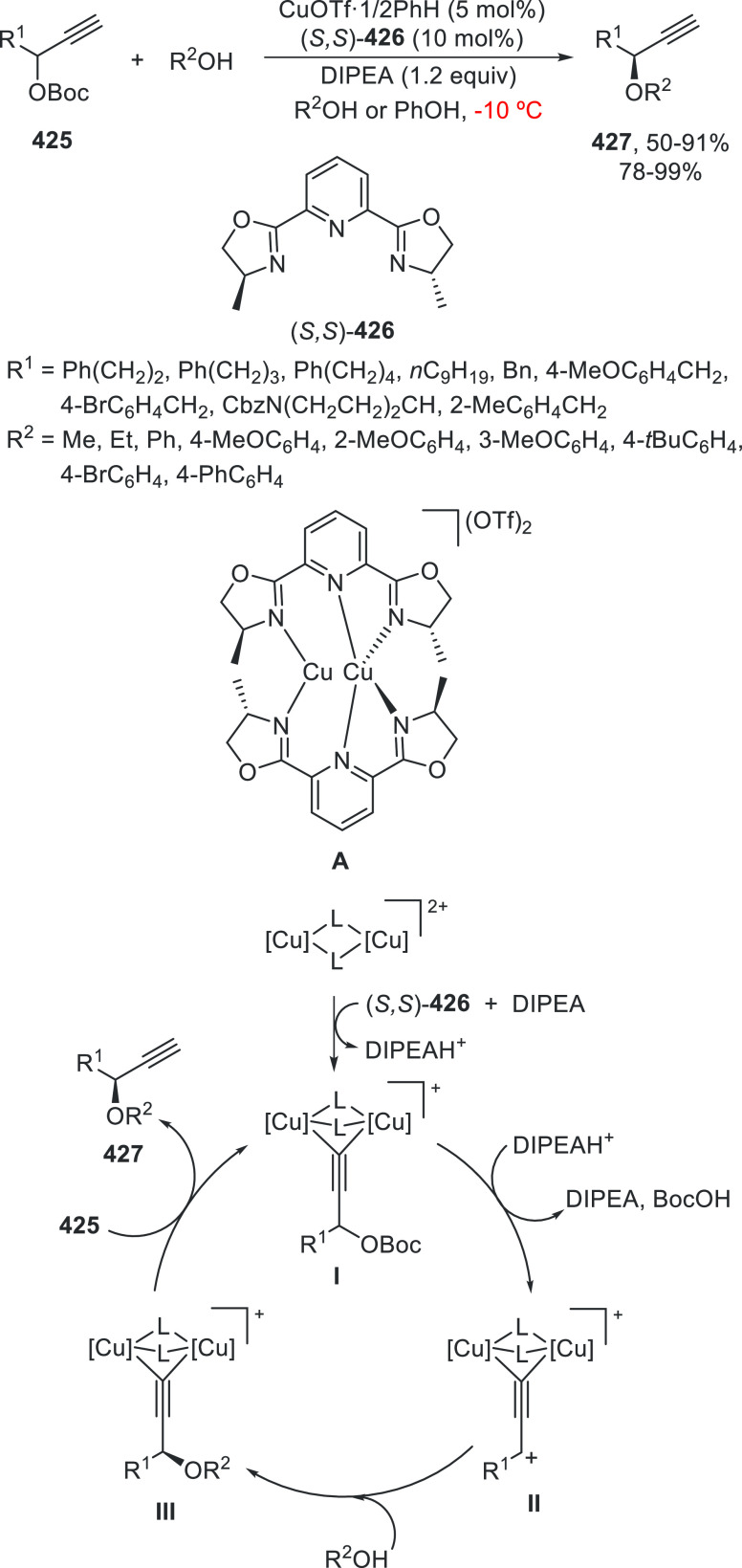
Enantioconvergent Cu-Catalyzed Etherification of Propargylic Carbonates **425** with Alcohols and Phenols

Transition metal catalysis is a powerful methodology for carbon–carbon bond formation between alcohols and alkyl electrophiles (see Section 2.2.2). Enantioconvergent substitution processes were initially described with propargyl derivatives. Nishibayashi and co-workers^278^ described the copper-catalyzed enantioconvergent etherification of propargylic carbonates **425** with alcohols and phenols using Py-bis(oxazoline) (*S,S*)-**426** as chiral ligand (Scheme 130). The resulting propargylic ethers **427** were obtained in up to 91% yield and with 78–99% ee. The isolated dicopper complex **A** explains the observed nonlinear effect. This complex reacts with propargyl carbonate **425** to give intermediate dicopper-acetylide **I**, which after elimination of a carbonate moiety can form a dicopper-allenylidene complex **II**. Alcohol attack to complex **II** at the *Si* face affords complex **III**, which evolves to liberate the propargyl ether by ligand exchange with another molecule of propargyl carbonate.

**Scheme 131:**
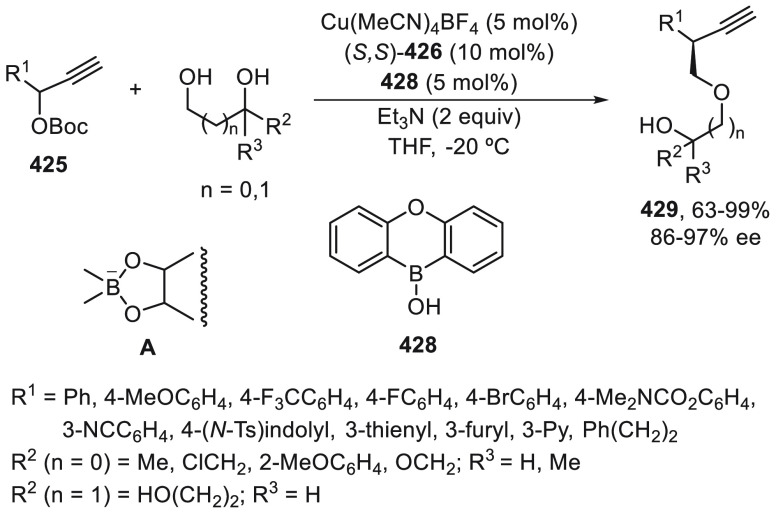
Enantioconvergent Cu/Borinic Acid-Catalyzed Etherification of Propargylic Carbonates **425** with Diols

Niu and co-workers^279^ employed Nishibayashi new conditions^280^ for benzyl alcohols in order to avoid the use of alcohols as solvents. Thus, polyols were propargylated with propargylic carbonates **425** under Cu(I)/borinic acid **428** dual catalysis and bis(oxazoline) PyBox (*S,S*)-**426** as chiral ligand (Scheme 131). Organoborinic acid **428** forms, according to Taylor’s studies,^281^ a boron “ate” complex **A**, thereby increasing the nucleophilicity of the diol. The resulting hydroxy ethers **429** were isolated in very good yields and with high enantioselectivity. A similar mechanism was proposed for this propargylation (see Scheme 130). This procedure was also applied to the desymmetrization of *meso* 1,2-diols.

**Scheme 132:**
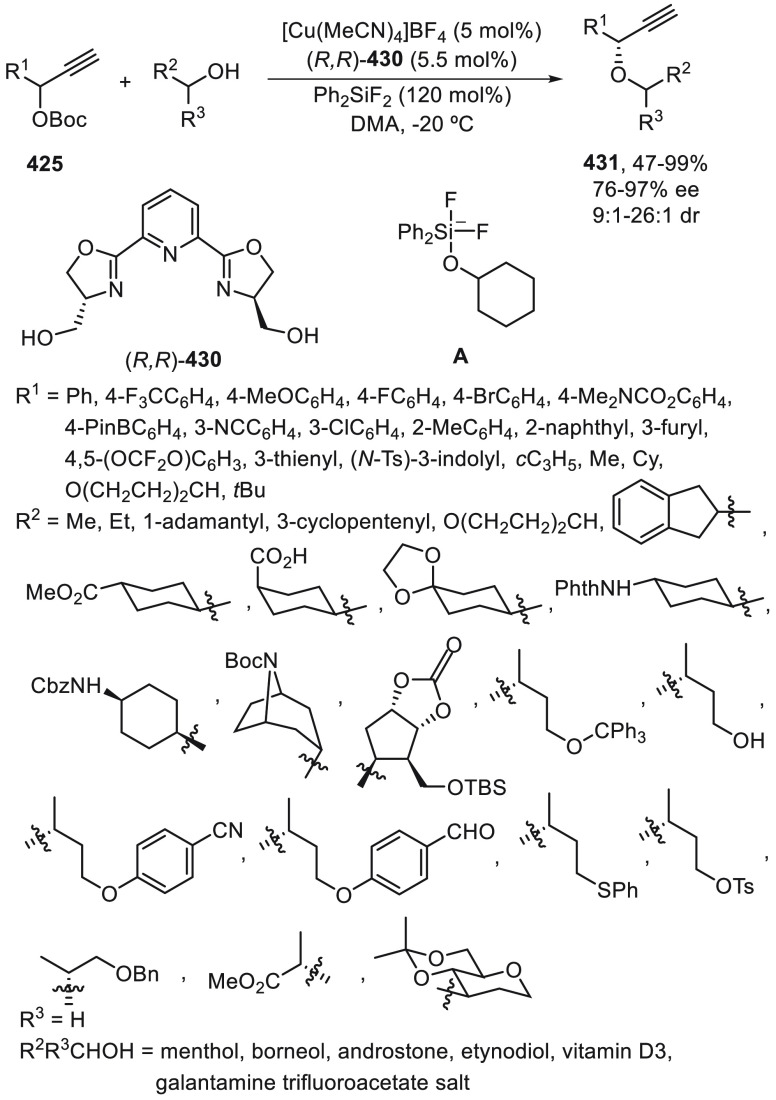
Enantioconvergent Cu-Catalyzed Etherification of Propargylic Carbonates **425** with Secondary Aliphatic Alcohols

The same group^282^ recently reported the enantioconvergent *O*-propargylation of secondary aliphatic alcohols. In this case, PyBox (*R,R*)-**430** was used as chiral ligand, and Ph_2_SiF_2_ was used as a mild Lewis acid additive to form the nucleophilic silicate **A**. Propargylic ethers **431** were isolated in good yields and with high enantio- and diastereoselectivity (Scheme 132). This method has been applied to derivatize bioactive (natural) products, such as androsterone, etynodiol, vitamin D_3_, and galantamine trifluoroacetate salt.

**Scheme 133:**
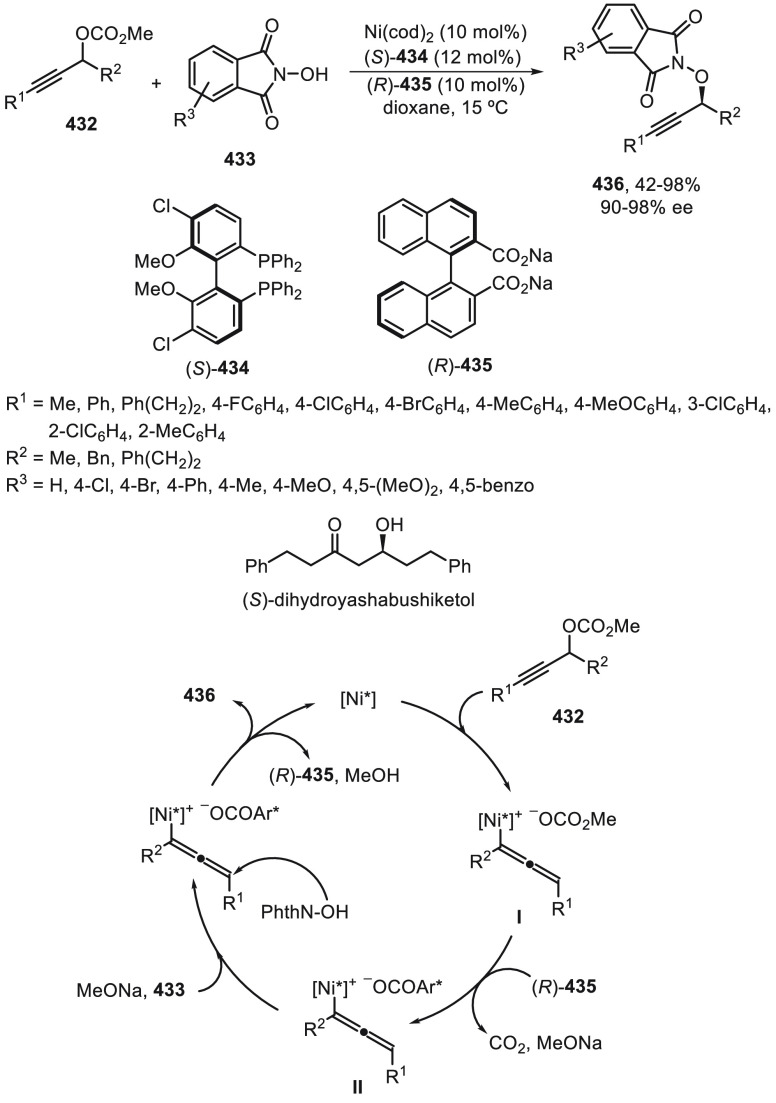
Enantioconvergent Ni/Sodium Carboxylate **435**-Catalyzed Propargylation of *N*-Hydroxyphthalimides **433** with Propargylic Carbonates **432**

An enantioconvergent nickel/chiral sodium carboxylate dual catalysis has been reported by Guo and co-workers^283^ for the *O*-propargylation reaction with hydroxylamines (Scheme 133). Propargylic carbonates **432** reacted with *N*-hydroxyphthalimides **433** using Ni(cod)_2_ as catalyst, diphosphine (*S*)-Cl–MeO-Bipep **434** as chiral ligand, and sodium dicarboxylate (*R*)-**435** as counteranion to provide products **436** in 42–98% yields with 90–98% ee. This strategy was applied, among others, to the total synthesis of (*S*)-dihydroyashabushiketol isolated initially from young shoots of *Alnus firma*(284) exhibiting 5α-reductase inhibition.^285^ From experimental studies, it was proposed that the Ni complex activates the propargylic carbonate to give an allenylnickel species **I**. After subsequent anion exchange, the nickel complex **II** is formed, which reacts with the *N*-hydroxyphthalimide to furnish the product and regenerate the catalyst.

Alcohols and phenols can be propargylated with propargylic carbonates using Cu(I)/bis(oxazoline) catalysts to the corresponding enantioenriched propargylic ethers. In the case of *N*-hydroxyphthalimides, the propargylation has been carried out under Ni/diphosphine catalysis with a sodium dicarboxylate as chiral counteranion.

### 8.3 Amination of 3-Bromooxindoles

**Scheme 134:**
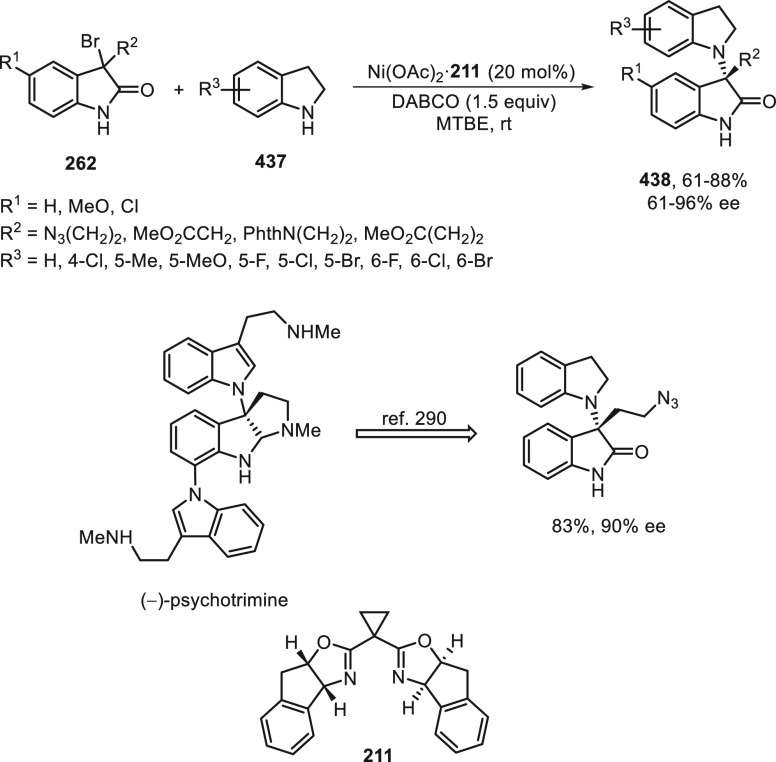
Enantioconvergent Ni-Catalyzed Amination of 3-Bromooxindoles **262** with Indolines **437**

The 3-substituted 3-aminooxidole unit is present in a large number of alkaloid natural products, drugs, and agrochemical compounds.^286−288^ Wang and co-workers^289^ reported an enantioconvergent amination of racemic 3-bromooxindoles **262** with indolines **437** under Ni(OAc)_2_/bis(oxazoline) **211** catalysis to afford 3-aminooxindoles **438** with good yields and enantioselectivities (Scheme 134). This method was applied to the formal synthesis of the cytotoxic alkaloid (+)-psychotrimine, which exhibits potent antitumor activity against colon and lung cancers^290^ and antibacterial activity against Gram-positive bacteria.^291^

**Scheme 135:**
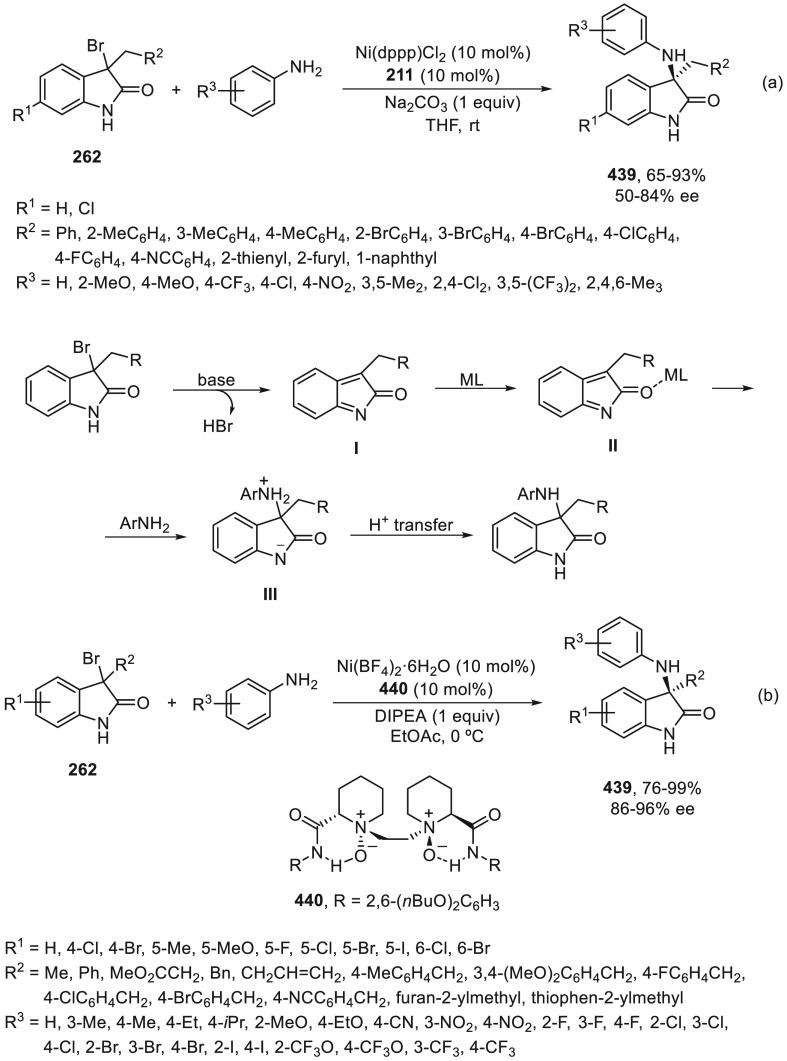
Enantioconvergent Ni-Catalyzed Amination of 3-Bromooxindoles **262** with Anilines

The same group^292^ reported the enantioconvergent amination of 3-bromooxindoles **262** with anilines under Ni(dppp)Cl_2_/bis(oxazoline) **211** catalysis to give 3-anilinooxindoles **439** with good yields and moderate enantioselectivities (Scheme 135a). In the proposed mechanism, the racemic 3-bromooxindole undergoes a base-mediated dehydrohalogenative process to provide *ortho*-azaxylylene **I**. Coordination of intermediate **I** with the chiral Ni(II) catalyst gives intermediate **II**, which suffers the aniline attack to form intermediate **III**. Final proton transfer and regeneration of the catalyst affords the product. Higher yields and enantioselectivities were obtained by Feng and co-workers^293^ using Ni(BF_4_)_2_/*N,N′-*dioxide **440** as catalyst (Scheme 135b). Working with DIPEA as base in EtOAc at 0 °C, compounds **439** were isolated in up to 99% yield and up to 96% ee.

The enantioconvergent amination of 3-bromooxindoles can be performed in the presence of a base and under Ni(II)/bis(oxazoline) catalysis by intermediacy of an *ortho*-azaxylylene formed by a dehydrohalogenative process.

### 8.4 Other Metal-Catalyzed Reactions

**Scheme 136:**
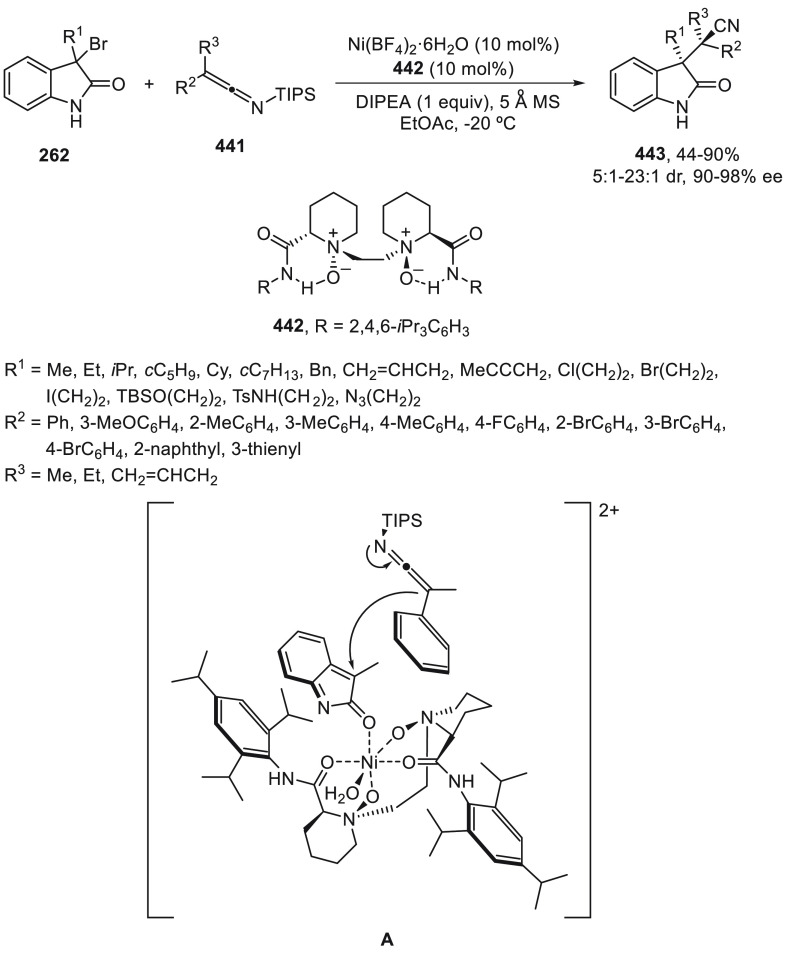
Enantioconvergent Ni-Catalyzed Reaction of 3-Bromooxindoles **262** with Silyl Ketene Imines **441**

Related with the previous Section 8.3 on reactivity of racemic 3-bromooxindoles **262** is the enantioconvergent Ni-catalyzed reaction of these compounds with silyl ketene imines **441** also reported by Feng and co-workers^294^ (Scheme 136). This reaction was carried out under Ni(BF_4_)_2_/*N,N′*-dioxide **442** catalysis at −20 °C to give products **443** by conjugate addition of silyl ketene imines **441** to the *in situ* generated indol-2-ones. The resulted enantioenriched β-alkyl nitriles **443** bearing vicinal all-carbon quaternary stereocenters were obtained in up to 90% yield with 23:1 dr and 98% ee. With the *N*-methyl-protected 3-bromooxindole, no desired product was obtained, which supports the participation of the indol-2-one. In the proposed stereochemical model **A**, the indol-2-one could be activated by the Ni complex with the *Si* face shielded by the 2,4,6-triisopropylphenyl group of the ligand. The resulting nitriles **443** have been transformed to enantioenriched pyrroloindoline frameworks and spirocyclohexane oxindole derivatives containing vicinal quaternary stereocenters.

**Scheme 137:**
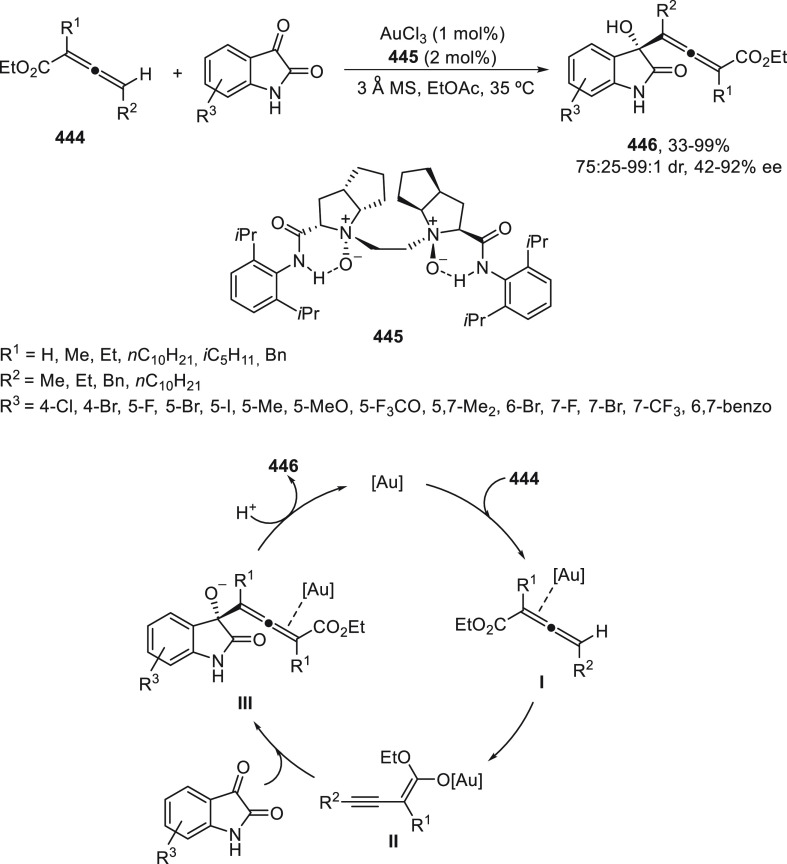
Enantioconvergent Au-Catalyzed Aldol Reaction of Allenolates **444** with Isatins

The same group reported^295^ an enantioconvergent alleno-aldol reaction catalyzed by AuCl_3_/*N,N′*-dioxide **445**. Racemic allenates **444** reacted with isatins regio-, diastereo-, and enantioselectively under mild reaction conditions to furnish carbinol allenolates **446** (Scheme 137). Complete γ-selectivity was observed to be controlled by the chiral catalyst. In the proposed catalytic cycle, the chiral gold complex [Au] coordinates the allenolate to form the π-adduct **I**. After γ-deprotonation and enolization to intermediate **II**, nucleophilic attack to isatin gives adduct **III**. Final protonation of **III** followed by elimination of the catalyst provides the product.

**Scheme 138:**
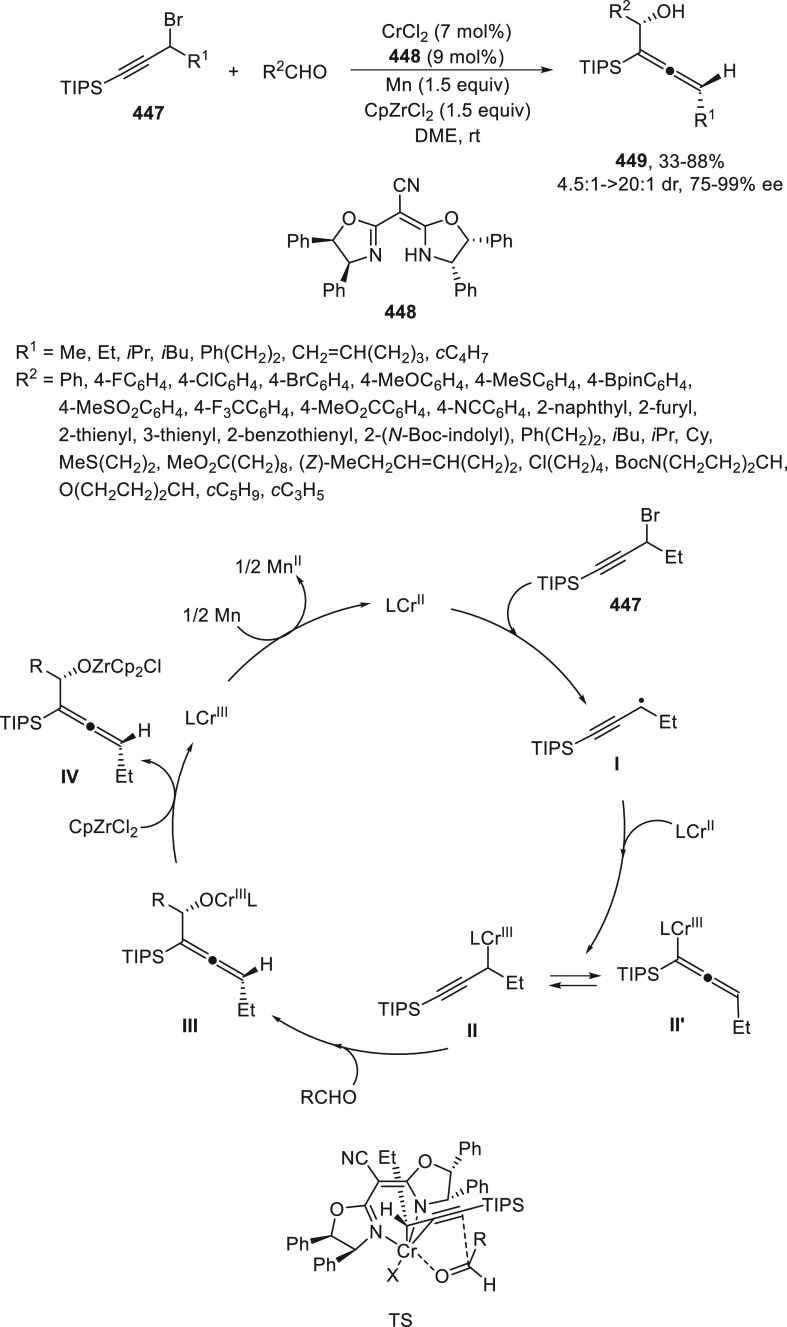
Enantioconvergent Cr-Catalyzed Allenylation of Aldehydes with Propargylic Bromides **447**

Recently, Wang and co-workers^296^ described the Cr-catalyzed allenylation of aldehydes with racemic propargyl bromides **447** (Scheme 138). In this case, CrCl_2_/bis(oxazoline) **448** was used as catalyst to furnish α-allenols **449** with adjacent axial and central chiralities. High regio-, diastereo-, and enantioselective control for products **449** were obtained with a broad substrate scope. Experimental studies suggested a radical reaction mechanism initiated by the single-electron reduction of the propargyl bromide by the Cr(II) complex to give the radical **I**. This radical is captured by the Cr(II) complex to form the propargylic and the allenylic Cr(III) intermediates **II** and **II′**, respectively. Subsequent reaction with the aldehyde provides intermediate **III**, which undergoes dissociation with Cp_2_ZrCl_2_ to give intermediate **IV**, a precursor of the α-allenol by hydrolysis. The Cr(III) complex is reduced to Cr(II) by manganese powder. Probably because of the steric repulsion between the TIPS group and the catalyst in the TS, the asymmetric allenylation, instead of the propargylation, is favored by the *Re* face attack.

**Scheme 139:**
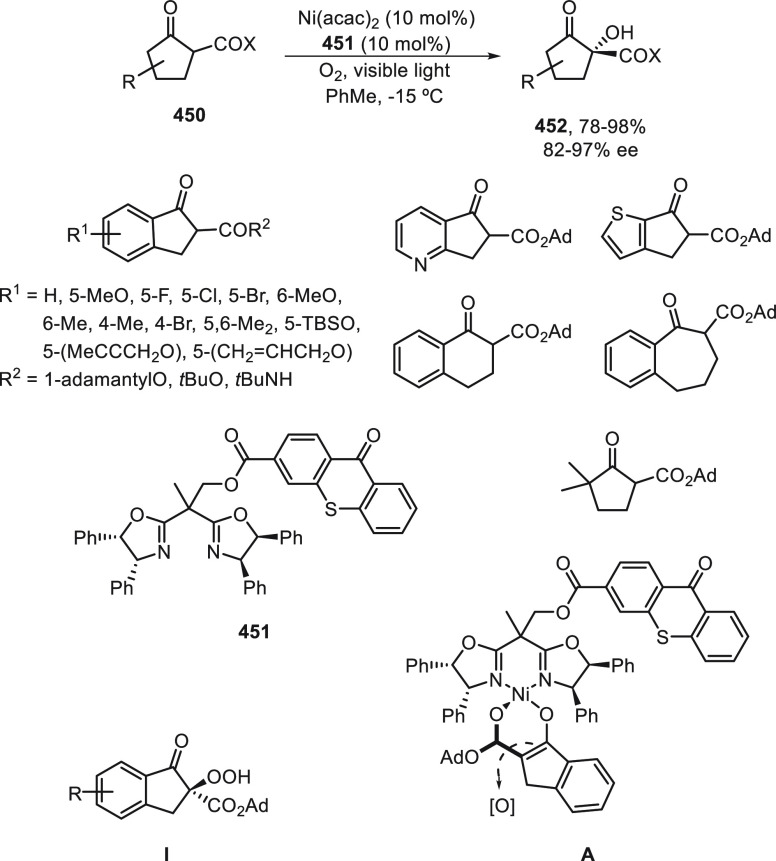
Enantioconvergent Photocatalyzed and Ni-Catalyzed Aerobic α-Hydroxylation of β-Keto Esters and Amides

Enantioconvergent aerobic oxidation of β-keto esters and amides **450** to α-hydroxy-β-dicarbonyl compounds **452** has been achieved by Xiao and co-workers^297^ under photocatalyzed conditions (Scheme 139). In this case, a chiral bis(oxazoline) ligand **451** was grafted with a photosensitive thioxanthone unit, and this ligand with Ni(acac)_2_ resulted as a powerful catalyst for the α-hydroxylation of compounds **450** using oxygen or air as a green oxidant under visible light. The thioxanthone motif acted as a triple-state sensitizer for the generation of singlet oxygen, and the Ni(II) cation acted as a Lewis acid and coordinated the indanone-derived esters and amides in the enolate form. A possible stereoinduction model **A** has been proposed in which the coordination enables the *Re* face attack of either the oxidant ^1^O_2_ or the peroxide **I**.

**Scheme 140:**
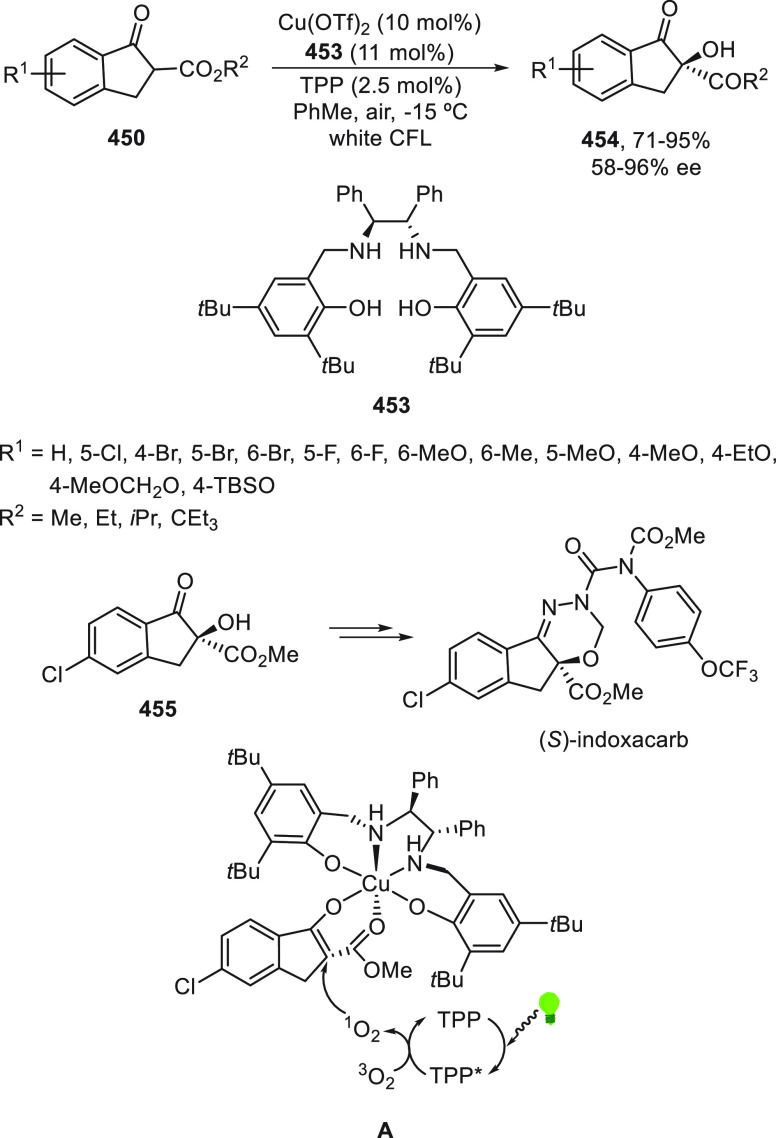
Enantioconvergent Photocatalyzed and Cu-Catalyzed α-Hydroxylation of β-Keto Esters **450**

Under visible light, Meng and co-workers^298^ performed the enantioconvergent α-hydroxylation of β-keto esters **450** under Cu(OTf)_2_/salan **453** catalysis. Cyclic substrates **450** were converted into products **454** with yields up to 95% and up to 96% ee by including methyl esters in the presence of air and tetraphenylporphyrin (TPP) as photosensitizer with a white compact fluorescent lamp (CFL) to produce singlet oxygen (Scheme 140). This protocol was applied to the synthesis of a key intermediate of the sodium channel blocker (*S*)-indoxacarb from compound **455**. According to experimental studies, it was suggested that reactive singlet oxygen may participate in this oxidation process, which attacks the enolate from the *Si* face in intermediate **A**.

Enantioconvergent addition of 3-bromooxindoles to silylketene imines takes place under Ni(II)/*N,N′*-dioxide catalysis through *in situ* generated indol-2-ones to provide β-alkyl nitriles. However, aldol reaction of allenoates occurs through an enantioconvergent gold-catalyzed reaction or under Cr-catalyzed addition of propargyl bromides to aldehydes. Enantioconvergent α-hydroxylation of β-keto esters and amides takes place by photo- and Ni- or Cu-catalyzed aerobic oxidations.

## Conclusions

Although in each Section a short conclusion sentence has been included, we enclose here a general conclusion. In Section 2, enantioconvergent cross-coupling of activated racemic alkyl electrophiles with aryl organometals can be performed under Ni catalysis by employing chiral bis(oxazoline) as ligands when Grignard and organozinc reagents are the nucleophiles. For organoboron reagents, chiral 1,2-diamines were the best ligands for the enantioconvergent cross-coupling of alkyl bromides and dichlorides with alkyl organoboranes. Bis(oxazolines) or diphosphines were used for aryl boronates and boronic acids under Ni, Co, Fe, and Cu catalysis. The formation of alkyl radicals as intermediates, which reacted with the chiral organonickel or Co, Fe, and Cu intermediates through an out-of-cage pathway, is the key step in these enantioselective transformations. Other organometals, such as organosilicon, organoindium, organozirconium, organoaluminum, and organotitanium reagents have been efficiently used for C(sp^3^)–C(sp^2^) bond-forming reactions under Ni(II)/bis(oxazoline) catalysis. Enantioconvergent amination and amidation reactions of racemic-activated alkyl bromides and chlorides can be carried out under photoinduced copper-catalyzed conditions using diphosphines as chiral ligands. Chiral tridentate anionic ligands promote the amination of α-chloro amides with aliphatic amines or ammonia under Cu(I) catalysis. Alcohols and phenols can be used as nucleophiles for the etherification of α-halo amides under Cu(I)/bis(oxazoline) catalysis. Benzylic and propargylic bromides undergo the Michaelis–Becker reaction with H-phosphonates under Cu/N,N-P ligand catalysis. Cyanation reactions require Cu(I)/bis(oxazoline) as catalyst. For the enantioconvergent alkynylation of alkyl bromides with acetylenes, Cu(I)/monophosphine or bis(oxazoline) is used as catalyst. However, tertiary α-chloro amides need chiral tridentate N,N,N-ligands for the alkynylation reaction and for secondary α-bromo amides to react under Cu/bis(oxazoline) catalysis. For the borylation of racemic benzylic bromides, Ni(II)/bis(oxazoline) or Cu(I)/diphosphine is employed as chiral ligand. Racemic alkylzinc reagents are cross-coupled with alkyl halides under Ni(II)/diamine or oxazoline catalysis. Enantioconvergent reductive cross-coupling of two electrophiles can be performed in the presence of a terminal reductant or under photoredox catalysis by intermediacy of stabilized alkyl radicals, which react with acyl chlorides, vinyl bromides, or aryl iodides, thereby avoiding the use of stoichiometric amounts of organometallic reagents. Photoredox decarboxylative enantioconvergent cross-coupling of α-amino acids with aryl bromides occurs mainly under Ni(II)/bis(oxazoline) catalysis. In the case of racemic-activated alkyl chlorides with aryl iodides, photoredox Ni(II)/bis(oxazoline) catalysis is employed. In Sections 3 and 4, racemic allylic and propargylic electrophiles are shown to undergo cross-coupling with organometals with complete regioselectivity and good enantioselectivity using Ni(II)/bis(oxazoline) and Cu(I)/phosphine catalysis. Enantioconvergent C(sp^2^)–H functionalizations with racemic electrophiles have been achieved under Cu(I)/phosphine or bis(oxazoline) catalysis by formation of radical intermediates. In the case of C(sp^3^)–H functionalizations, racemic compounds react at the α-, β-, or γ-position under Ni, Cu, or Co catalysis. Allylic alkylation was possible under Pd/phosphoramidite catalysis with racemic nucleophiles, such as 1,3-dicarbonyls, azlactones, aldehydes, pyrazolones, or thiazolones. Hydroalkylation of alkenes with racemic-activated alkyl electrophiles under Ni(II)/bis(oxazoline) catalysis in the presence of triethoxysilane promotes the formation of a Ni-hydride. In the case of racemic nucleophiles, the enantioconvergent hydroalkylation of dienes and allenes has been performed under Pd(II)/diphosphine catalysis. Racemic electrophiles have been employed in the enantioconvergent hydroalkylation of internal acetylenes under Ni or Rh catalysis. Enantioconvergent hydrogen autotransfer of racemic alcohols with nitrogen-containing nucleophiles was carried out mainly under Ir or Ru catalysis. In the case of alkylation of enolates, racemic alcohols react with primary alcohols either under Ir or Ru catalysis. Concerning other metal-catalyzed enantioconvergent reactions, racemic allylic systems have been added to carbonyl compounds or alcohols via transfer hydrogenative conditions, mainly under Ir(I)/diphosphine catalysis, with high regio-, diastereo-, and enantioselectivities. Alcohols and phenols can be propargylated with propargylic carbonates under Cu–bis(oxazoline) catalysis in the presence of borinic acids or diphenyldifluorosilane as alcohol activators. *N*-Hydroxyphthalimides are propargylated under Ni/diphosphine catalysis with a chiral carboxylate as counteranion. Amination of racemic 3-bromooxindoles were performed under Ni catalysis, other reactions involving allenylation of carbonyl compounds were performed under Au or Cr catalysis with racemic allenolates or propargylic bromides, and hydroxylation of racemic 1,3-dicarbonyl compounds under Ni or Cu catalysis were carried out. This review has attempted to clarify the terminology of enantioconvergent processes and to overview their recent developments and applications to the synthesis of enantioenriched molecules. It is worthy to highlight the impressive development of radical-based enantioconvergent reactions, which have evolved from simple bis(oxazolines) as chiral ligands under nickel catalysis to tridentate anionic ligands under copper catalysis able to facilitate the weak oxidative properties of alkyl halides. A broad array of enantioconvergent reactions have been described so far, but more studies should be carried out to explain the stereochemical results.

## Abbreviations

AAA: asymmetric allylic alkylation
Ac: acetyl
acac: acetoacetyl
Alloc: allyloxycarbonyl
AMG 837: 1-propyn-1-yl-4-{[4′-(trifluoromethyl)-1,1′-biphenyl]-3-yl-methoxy}benzenepropanoic acid
Ar: aryl
BARF: tetrakis[3,5-bis(trifluoromethyl)phenyl]borate
BBN: 9-borabicyclo[3.3.1]nonane
BenzP: 12-(*t*-butylmethylphosphorous)benzene
BHT: 2,6-di-*tert*-butyl-4-methylphenyl
Binap: 2,2′-bis(diphenylphosphino)-1,1′-binaphthyl
Bn: benzyl
BnPyBox: 2,6-bis[(4*S*)-benzyl-2-oxazolin-2-yl]pyridine
Boc: *tert-*butyloxycarbonyl
BOPA: bis(oxazoline)-phenylaniline
Bpin: bis(pinacolate)diboron
bpy: 2,2′-bipyridine
BTPP: *tert*-butylimino-tri(pyrrolidino)phosphane
Bz: benzyl
carb: carbazolide
cat: catalyst
Cbz: benzyloxycarbonyl
*c*C_5_H_9_: cyclopentyl
CFL: compact fluorescent lamp
cod: 1,5-cyclooctadiene
Cp: ferrocenyl
CPA: chiral phosphoric acid
CPME: cyclopentyl methyl ether
Cy: cyclohexyl
Cz: carbazolyl
4CzIPN: 2,4,5,6-tetracarbazolyl-1,3-denzene dinitriles
DABCO: 1,4-diazabicylo[2.2.2]octane
dba: dibenzylideneacetone
DCM: dichloromethane
DFB: 1,2-difluorobenzene
DFT: density functional theory
DHFR: dihydrolate reductase
DHP: dihydroxypyridine
DIAD: diisopropyl azodicarboxylate
diglyme: bis(2-methoxyethyl) ether
DIPEA: *N,N*-diisopropylethylamine
DKR: dynamic kinetic resolution
DMA: *N*,*N*-dimethylacetamide
DMAP: 4-dimethylaminopyridine
DMBA: dimethylbenzoic acid
DMBQ: dimethylbenzoquinone
DME: dimethoxyethane
DMF: dimethylformamide
DMI: 1,3-dimethyl-2-imidazolidinone
DMM-Box: 5-benzyl-2-[2-bis(3,5-dimethyl-4-methoxyphenyl)phosphino]oxazoline
DMPU: *N,N′*-dimethylpropylurea
DMSO: dimethyl sulfoxide
DpePhos: bis[(2-phenylphosphino)phenyl] ether
dppp: 1,3-bis(diphenylphosphino)propane
dr: diastereomeric ratio
DTBM-Binap: 2,2′-bis(3,5-di-*tert*-buty-4-methoxy)phenyl-1,1′-binaphthyl
DTBM-Segphos: 5,5′-bis[di(3,5-di-*tert*-butyl-4-methoxyphenyl)phosphino]-4,4′-dibenzodioxole
DyKAT: dynamic kinetic asymmetric transformations
ee: enantiomeric excess
EPR: electron paramagnetic resonance
equiv: equivalent(s)
ESI-MS: electrospray ionization-mass spectrometry
EZH2: enhancer of zeste homologue 2
Fmoc: fluorenylmethyloxycarbonyl
glyme: 1,2-dimethoxyethane
GPR 40: fatty acid receptor agonist
HEH: Hantzsch ester
Het: heterocycle
HFacac: hexafluoroacetoacetate
HP: high-power
HQ: hydroquinone
IPrAuNTf_2_: [1,3-bis(2,6-diisopropylphenyl)imidazol-2-ylene] [bis(trifluoromethanesulfonyl)imide] gold
iPrPyBox: 2,6-bis[(4-*S*)(−)-isopropyl-2-oxazolin-2-yl]pyridine
L: ligand
LED: light-emitting device
LUMO: lowest unoccupied molecular orbital
M: metal
M-H: Michaelis–Becker reaction
MeOBiphen: 2,2′-bis(diphenylphosphino)-6,6′-dimethoxy-1,1′-biphenyl
mGluR: metabotyropic glutamate receptor
mol: mole(s)
Ms: methylsulfonyl (mesyl)
MS: molecular sieve
MTBE: methyl *tert-*butyl ether
NHP: *N*-hydroxyphthalimide
NMP: *N*-methylpyrrolidone
NSAID: nonsteroidal anti-inflammatory drug
Nu: nucleophile
OFBA: 2-fluorobenzoic acid
PC: photocatalyst
Pc: phthaloccyanine
PG: protecting group
PhBox: 2 × 2-bis(4-phenyl-2-oxazolin-2-yl)propane
Phe: phenylalanine
Phen: 1,10-phenanthroline
Ph-PTZ: *N*-phenylphenothiazine
Phth: phthalimido
Piv: pivaloyl
PMB: *p*-methoxybenzyl
PMP: *p*-methoxyphenyl
Por: porphirine
ppy: 2-phenylpyridine
QuinoxP*: 2,3-bis(*tert*-butylmethylphosphino)quinoxaline
rac: racemic
RCC: reductive cross-coupling
red: reductant
rt: room temperature
RVC: reticulated vitreous carbon
Segphos: 5,5′-bis(diphenylphosphino)4,4′-bi-1,3-benzodioxole
SET: single-electron transfer
TADDOL: α α,α′,α′-tetraaryl-2,2-disubstituted 1,3-dioxolane-4,5-dimethanol
TBAF: tetrabutylammonium fluoride
TBAT: tetrabutylammonium triphenyldifluorosilicate
TBDPS: *tert*-butyldiphenylsilyl
TBS: *tert*-butyldimethylsilyl
TC: thiophene-2-carboxylate
TDAE: tetrakis(*N,N*-dimethylamino)ethylene
TEA: triethylamine
TES: triethylsilyl
Tf: trifluoromethylsulfonyl (triflyl)
TFA: trifluoroacetic acid
THF: tetrahydrofuran
THP: tetrahydropyranyl
TIPS: triisopropylsilyl
TMB: 1,3,5-trimethoxybenzene
TMEDA: tetramethylenediamine
TMS: trimethylsilyl
Tol: tolyl
TPP: tetraphenylprophyrine
TRIP: 3,3′-bis(2,4,6-triisopropylphenyl)-1,1′-binaphthyl-2,2′-diyl hydrogenophosphate
Ts: 4-methylbenzenesulfonyl (tosyl)
TS: transition state
UPC1172: 4-{3-[(2*S*)-4-2,4-diamino-6-ethylpyrimidin-5-yl]-5-methoxy}benzoic acid
